# Correction: Adverse pregnancy and birth outcomes associated with Mycoplasma hominis, Ureaplasma urealyticum and Ureaplasma parvum: a systematic review and meta-analysis

**DOI:** 10.1136/bmjopen-2022-062990corr1

**Published:** 2023-09-22

Jonduo ME, Vallely LM, Wand H, *et al*. Adverse pregnancy and birth outcomes associated with Mycoplasma hominis, Ureaplasma urealyticum and Ureaplasma parvum: a systematic review and meta-analysis. *BMJ Open* 2022;12:e062990. doi: 10.1136/bmjopen-2022-062990

This article has been corrected since it was first published. In this systematic review, some of the included data were based on interpretations of microbiological methods used to identify *Ureaplasma urealyticum*, which were not consistent with the methods reported by the primary study authors. We have revised our analyses of the organisms *U. urealyticum* and *U. parvum* and their associations with adverse pregnancy outcomes. We removed data from meta-analyses if identification of *U. urealyticum* was ambiguous in the original study report and we had erroneously extracted data based on our interpretation of the study, and we amended data from studies that identified biovars of *Ureaplasma* spp. but for which biovar 1 (*U. parvum*) was reported as *U. urealyticum*.

None of the revised analyses change our conclusion, that ‘The currently available literature does not allow conclusions about the role of mycoplasmas in adverse pregnancy and birth outcomes’.

The original systematic review included 57 articles. Of 14 articles found to have problems with microbiological methods, 10 articles remained eligible for inclusion in the review; 2 with re-categorised data^1 2^ and 8 with data removed from some meta-analyses.^3–10^ The other four articles were removed from the review because the methods reported did not distinguish between *Ureaplasma* spp. and there were no other eligible data.^11–14^ We also removed data from one included study^15^ from the meta-analysis of the association between *U. urealyticum* and preterm birth because the organism was not detected in any samples.

The corrected version of the systematic review includes 53 articles. Table A, table 2, figure 2 and figure 3 in the main text have been replaced with revised data. All affected data in the text have been replaced and the supplemental file has been revised. The revised analyses resulted in some changes to the results of meta-analyses. Table A, summarises the results presented in table 2 in the article with the first version alongside the results of the revised analyses of the primary outcome for both *U. urealyticum* and *U. parvum*, and of secondary outcomes for *U. urealyticum*. Secondary outcomes for associations with *U. parvum* were unaffected by the revised data. In addition to these changes, we replaced figure 1 in the main text because the date of publication in one study was incorrect, and made some grammatical corrections.

**Table A:** Original and revised summary estimates from random effects meta-analysis, by outcome for *U. urealyticum* and *U. parvum*

Adverse outcome Organism	Results in original article				Results of revised meta-analyses			
	No. of studies	Summary estimate OR (95% CI)	I^2^, %	Prediction interval	No. of studies	Summary estimate OR (95% CI)	I^2^ %	Prediction interval
Preterm birth								
*U. urealyticum*	27	1.84 (1.34, 2.55)	69.2	0.54, 6.36	16	1.96 (1.14, 3.39)	53.1	0.40, 9.73
*U. parvum*	11	1.60 (1.12, 2.30)	58.4	0.59, 4.36	13	1.79 (1.28, 2.52)	59.0	0.66, 4.85
Premature rupture of membranes								
*U. urealyticum*	11	4.27 (1.83, 9.98)	87.3	0.27, 68.07	4	9.87 (1.81, 53.72)	49.0	0.02, 5757.86
Low birth weight								
*U. urealyticum*	2	2.24 (1.16, 4.33)	0.0	NC	1	1.08 (0.08, 14.41)	NA	NA
Spontaneous abortion								
*U. urealyticum*	4	1.74 (1.02, 2.95)	0.0	0.54, 5.58	3	2.43 (1.21, 4.86)	0.0	0.03, 217.73
Perinatal death								
*U. urealyticum*	2	9.50 (2.99, 30.13)	0.0	NC	1	3.52 (0.14, 87.08)	NA	NA

NA, not applicable when only one study included; NC, could not be calculated because number of studies is too small.
