# Supplementary material for: Adverse pregnancy and birth outcomes associated with Mycoplasma hominis, Ureaplasma urealyticum and Ureaplasma parvum: a systematic review and meta-analysis
Source: BMJ Open. 2022 Aug 26;12(8):e062990. doi: 10.1136/bmjopen-2022-062990 (PMC9422885; doi:10.1136/bmjopen-2022-062990)
Supplement: Supplementary data [file bmjopen-2022-062990supp001.pdf]

ONLINE SUPPLEMENTAL FILE

Adverse pregnancy and birth outcomes associated with *M. hominis, U. urealyticum* and *U. parvum*: A systematic review and meta-analysis.

Marinjho Emely Jonduo,[1] Lisa Michelle Vallely,[1,2] Handan Wand,[3] Emma Louise Sweeney,[4] Dianne Egli-Gany,[5] John Martin Kaldor,[1] Andrew John Vallely[1,2] and Nicola Low,[5]

[1] Public Health Interventions Research Program, The Kirby Institute, University of New South Wales, Sydney, NSW 2052, Australia.

[2] Sexual and Reproductive Health Unit, Papua New Guinea Institute of Medical Research, Goroka, Eastern Highlands Province 441, Papua New Guinea.

[3] Biostatistics and Databases program, The Kirby Institute, University of New South Wales, Sydney, New South Wales, Australia.

[4] The University of Queensland Center for Clinical Research, Brisbane, Queensland 4029, Australia.

[5] Institute of Social and Preventive Medicine, University of Bern, CH-3012 Bern, Switzerland.

Jonduo ME, *et al. BMJ Open* 2023; 12:e062990. doi: 10.1136/bmjopen-2022-062990

ONLINE SUPPLEMENTAL FILE

Table of contents

A.1 Preferred reporting items for systematic review and meta-analysis (PRISMA) ... 3

A.2 Search strategy ... 6

Figure S1 Flow chart of identified and selected studies for inclusion ... 7

Table S1 Summary of characteristics of included studies, alphabetical order ... 8

Figure S3.1 Forest plot of association between *M. hominis* and premature rupture of membrane, random effects model ... 17

Figure S3.2 Forest plot of association between *M. hominis* and low birthweight, random effects model ... 18

Figure S3.3 Forest plot of association between *M. hominis* and perinatal death random effects model. ... 19

Figure S3.4 Forest plot of association between *M. hominis* and spontaneous abortion random effects model ... 20

Figure S3.5 Forest plot of association between *U. urealyticum* and premature rupture of membrane, random effects model. ... 21

Figure S3.6 Forest plot of association between *U. urealyticum* and spontaneous abortion, random effects model. ... 21

Figure S3.7 Forest plot of association between *U. parvum* and premature rupture of membrane, random effects model ... 22

Figure S3.8 Forest plot of association between *U. parvum* and spontaneous abortion random effects model. ... 22

Table S2.1 Descriptive tables: cohort studies (n=26), in alphabetical order ... 23

Table S2.2 Descriptive tables: case-control studies (n=22), in alphabetical order ... 26

Table S2.3 Descriptive tables: cross-sectional studies (n=5), in alphabetical order ... 28

Table S3.1 Summary description of studies reporting *M. hominis* (n=42) in alphabetical order, by country-level income status ... 29

Table S3.2 Summary description of studies reporting *U. urealyticum* (n=18) in alphabetical order, by country-level income status ... 33

Table S3.3 Summary description of studies reporting *U. parvum* (n=14) in alphabetical order ... 36

Table S4.1 Study setting and socio-demographics, cohort studies (n=26), in alphabetical order ... 38

Table S4.2 Study setting and socio-demographics, case-control studies (n=22), in alphabetical order ... 41

Table S4.3 Study setting and socio-demographics, cross-sectional studies (n=5), in alphabetical order ... 43

Table S5 Studies that reported on bacterial vaginosis and associations with adverse birth outcomes (n=10), in alphabetical order ... 44

Table S6.1 Risk of bias assessment, cohort studies (n=26) ... 47

Table S6.2 Risk of bias assessment, case-control studies (n=22) ... 53

Table S6.3 Risk of bias assessment, cross-sectional studies (n=5) ... 56

Table S7 Summary of assessment of regression analysis for small study effects, for outcomes reported in 10 or more studies ... 57

ONLINE SUPPLEMENTAL FILE

A.1 Preferred reporting items for systematic review and meta-analysis (PRISMA)

Section and Topic	Item #	Checklist item	Location where item is reported in published pdf
TITLE			
Title	1	Identify the report as a systematic review.	Page 1
ABSTRACT			
Abstract	2	See the PRISMA 2020 for Abstracts checklist.	Page 1
INTRODUCTION			
Rationale	3	Describe the rationale for the review in the context of existing knowledge.	Page 1
Objectives	4	Provide an explicit statement of the objective(s) or question(s) the review addresses.	Page 2
METHODS			
Eligibility criteria	5	Specify the inclusion and exclusion criteria for the review and how studies were grouped for the syntheses.	Page 2
Information sources	6	Specify all databases, registers, websites, organisations, reference lists and other sources searched or consulted to identify studies. Specify the date when each source was last searched or consulted.	Page 2
Search strategy	7	Present the full search strategies for all databases, registers, and websites, including any filters and limits used.	Appendix A.2
Selection process	8	Specify the methods used to decide whether a study met the inclusion criteria of the review, including how many reviewers screened each record and each report retrieved, whether they worked independently, and if applicable, details of automation tools used in the process.	Page 2
Data collection process	9	Specify the methods used to collect data from reports, including how many reviewers collected data from each report, whether they worked independently, any processes for obtaining or confirming data from study investigators, and if applicable, details of automation tools used in the process.	Page 2
Data items	10a	List and define all outcomes for which data were sought. Specify whether all results that were compatible with each outcome domain in each study were sought (e.g., for all measures, time points, analyses), and if not, the methods used to decide which results to collect.	Page 2
	10b	List and define all other variables for which data were sought (e.g., participant and intervention characteristics, funding sources). Describe any assumptions made about any missing or unclear information.	Page 2
Study risk of bias assessment	11	Specify the methods used to assess risk of bias in the included studies, including details of the tool(s) used, how many reviewers assessed each study and whether they worked independently, and if applicable, details of automation tools used in the process.	Page 2
Effect measures	12	Specify for each outcome the effect measure(s) (e.g., risk ratio, mean difference) used in the synthesis or presentation of results.	Page 2-3

ONLINE SUPPLEMENTAL FILE

Section and Topic	Item #	Checklist item	Location where item is reported in published pdf
Synthesis methods	13a	Describe the processes used to decide which studies were eligible for each synthesis (e.g., tabulating the study intervention characteristics and comparing against the planned groups for each synthesis (item #5)).	c
	13b	Describe any methods required to prepare the data for presentation or synthesis, such as handling of missing summary statistics, or data conversions.	Page 3
	13c	Describe any methods used to tabulate or visually display results of individual studies and syntheses.	Page 3
	13d	Describe any methods used to synthesize results and provide a rationale for the choice(s). If meta-analysis was performed, describe the model(s), method(s) to identify the presence and extent of statistical heterogeneity, and software package(s) used.	Page 3
	13e	Describe any methods used to explore possible causes of heterogeneity among study results (e.g., subgroup analysis, meta-regression).	Page 3
	13f	Describe any sensitivity analyses conducted to assess robustness of the synthesized results.	None
Reporting bias assessment	14	Describe any methods used to assess risk of bias due to missing results in a synthesis (arising from reporting biases).	Page 2
Certainty assessment	15	Describe any methods used to assess certainty (or confidence) in the body of evidence for an outcome.	None
RESULTS			
Study selection	16a	Describe the results of the search and selection process, from the number of records identified in the search to the number of studies included in the review, ideally using a flow diagram.	Page 3, figure S1
	16b	Cite studies that might appear to meet the inclusion criteria, but which were excluded, and explain why they were excluded.	Figure S1
Study characteristics	17	Cite each included study and present its characteristics.	Page 3, table S1, tables 2.1-2.3, 3.1-3.3, 4.1-4.3
Risk of bias in studies	18	Present assessments of risk of bias for each included study.	Page 3, tables 3.1-3.3, 6.1-6.3
Results of individual studies	19	For all outcomes, present, for each study: (a) summary statistics for each group (where appropriate) and (b) an effect estimates and its precision (e.g., confidence/credible interval), ideally using structured tables or plots.	Page 3-7, table 2, fig 1-3, figure S3.1-3.8
Results of syntheses	20a	For each synthesis, briefly summarise the characteristics and risk of bias among contributing studies.	Page 3
	20b	Present results of all statistical syntheses conducted. If meta-analysis was done, present for each the summary estimate and its precision (e.g., confidence/credible interval) and measures of statistical heterogeneity. If comparing groups, describe the direction of the effect.	Page 3-7, table 2, figure 1-3, figure S3.1-3.8
	20c	Present results of all investigations of possible causes of heterogeneity among study results.	None

ONLINE SUPPLEMENTAL FILE

Section and Topic	Item #	Checklist item	Location where item is reported in published pdf
	20d	Present results of all sensitivity analyses conducted to assess the robustness of the synthesized results.	None
Reporting biases	21	Present assessments of risk of bias due to missing results (arising from reporting biases) for each synthesis assessed.	Page 3, table S7
Certainty of evidence	22	Present assessments of certainty (or confidence) in the body of evidence for each outcome assessed.	None
DISCUSSION			
Discussion	23a	Provide a general interpretation of the results in the context of other evidence.	Page 8-10
	23b	Discuss any limitations of the evidence included in the review.	Page 8
	23c	Discuss any limitations of the review processes used.	Page 8
	23d	Discuss implications of the results for practice, policy, and future research.	Page 9-10
OTHER INFORMATION			
Registration and protocol	24a	Provide registration information for the review, including register name and registration number, or state that the review was not registered.	Page 1
	24b	Indicate where the review protocol can be accessed, or state that a protocol was not prepared.	Page 2, reference 22
	24c	Describe and explain any amendments to information provided at registration or in the protocol.	None
Support	25	Describe sources of financial or non-financial support for the review, and the role of the funders or sponsors in the review.	Page 10
Competing interests	26	Declare any competing interests of review authors.	Page 10
Availability of data, code and other materials	27	Report which of the following are publicly available and where they can be found; template data collection forms; data extracted from included studies; data used for all analyses; analytic code; any other materials used in the review.	Online supplemental file

ONLINE SUPPLEMENTAL FILE

A.2 Search strategy

1. Terms for population	"pregnancy" or "prenatal" or "antenatal"
2. Terms for exposure	"Mycoplasma hominis" or "M. hominis"; "Ureaplasma urealyticum" or "U. urealyticum"; "Ureaplasma parvum" or "U. parvum"
3. Terms for outcomes	"birth outcome" or "adverse birth outcome" or "adverse pregnancy outcome" or "perinatal morbidity" or "perinatal mortality" or "perinatal outcome" or "premature birth" or "premature delivery" or "very preterm birth" or "preterm birth" or "preterm delivery" or "premature labour" or "preterm labour" or "premature labor" or "preterm labor" or "premature rupture of membranes" or "preterm rupture of membranes" or "preterm premature rupture of membranes" or "low birth weight" or "intrauterine growth retardation" or "intrauterine growth restriction" or "small for gestational age" or "gestational age" or "stillbirth" or "perinatal mortality" or "perinatal morbidity" or "perinatal death" or "neonatal mortality" or "neonatal morbidity" or "neonatal death" or "fetal death" or "miscarriage" or "spontaneous abortion" or "chorioamnionitis"
4. Search = #1 + # 2 + # 3	

Free text terms in the search strategy will use truncated and wildcard forms e.g., pregn*
Explode function used for MeSH headings
Reference lists of retrieved articles searched

ONLINE SUPPLEMENTAL FILE

Figure S1 Flow chart of identified and selected studies for inclusion

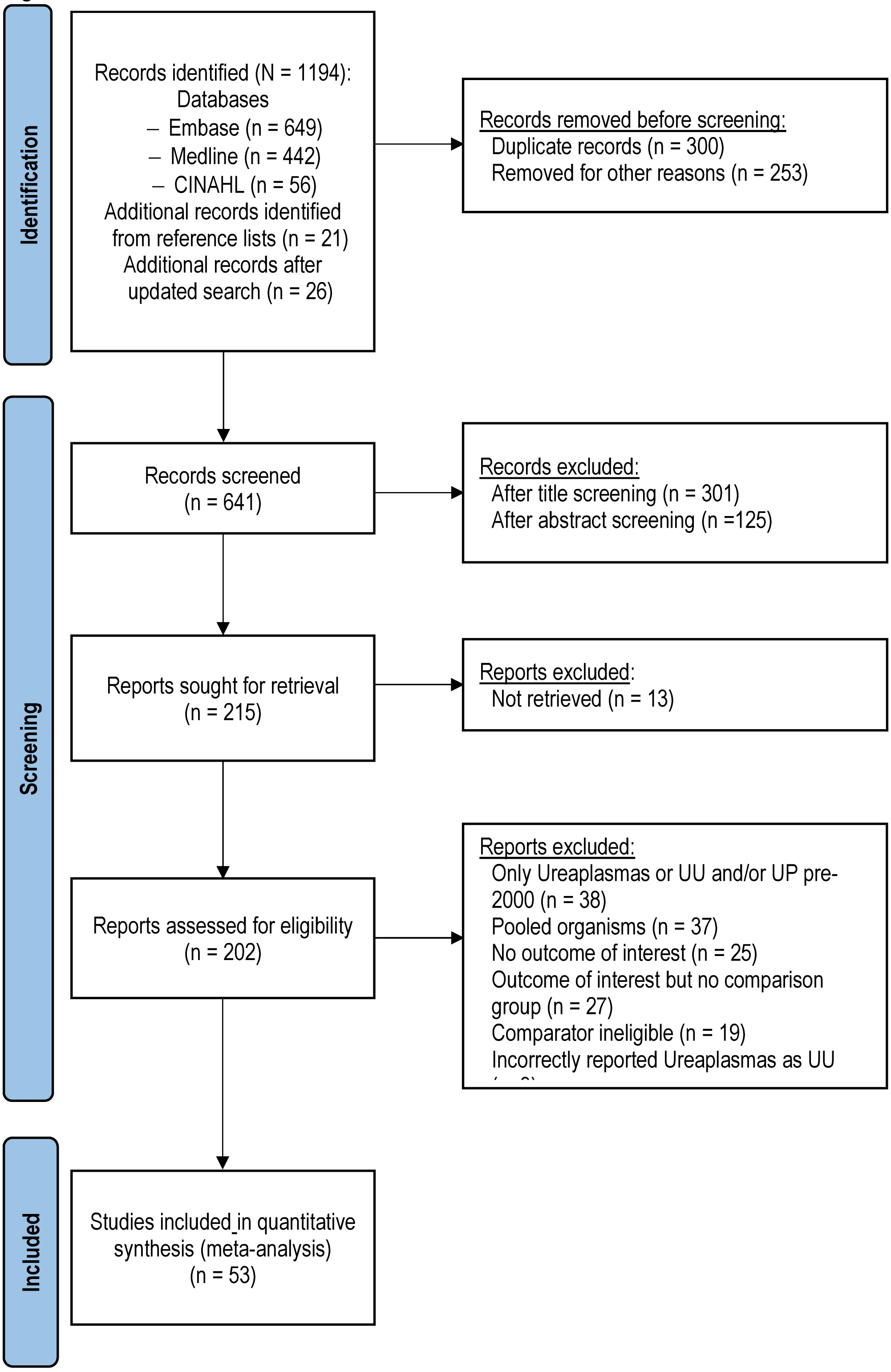

UP, *Ureaplasma parvum*; UU, *Ureaplasma urealyticum*

ONLINE SUPPLEMENTAL FILE

Table S1 Summary of characteristics of included studies, alphabetical order

First author, publication year, study reference*†	Country	Study design	Study population	Total no. of women	Outcomes measured	Specimen type; collection time	Diagnostic method	BV assessed
Agger, 2014[1]	USA	Cohort	Between 10-14 weeks gestation, initial prenatal visit, currently uncomplicated pregnancy	783	PTB	Endocervical swab; 1st, 2nd trimester	NAAT	NR
Ahmadi, 2014[9]	Iran	Case-control	10-20 weeks (cases), normal pregnancy 20-30 weeks (control)	218	SA	Endocervical swab; 1st, 2nd, 3rd trimester	NAAT	NR
Berman, 1987[34]	Mexico	Cohort	Women at their prenatal care visit, Oct 1980 - Oct 1983; single centre study	1204	LBW	Endocervical swab; 1st, 2nd, 3rd trimester	Culture	NR
Braun, 1971[35]	USA	Cohort	Entering antenatal clinic, Feb - Jul 1969; single centre study	688	LBW	Endocervical swab, urine; 1st, 3rd trimester	Culture	NR
Cassell, 1983[32]	USA	Case-control	Attending the amniocentesis for prenatal diagnosis; single centre study	61	PTB, PND	Amniotic fluid; 2nd trimester	Culture	NR
Chua, 1999[67]	Malaysia	Case-control	60 sequential mother who delivered and premature babies, Jan 1996-June 1997; single centre study	120	PTB	Endocervical swab; 2nd, 3rd trimester	Culture	NR
Daskalakis, 2009[65]	NR	Case-control	Singleton, normal pregnancy, >18 years old, mid-trimester amniocentesis, Feb 2006 - Sept 2007	613	PTB	Amniotic fluid; 2nd trimester	Culture	NR

ONLINE SUPPLEMENTAL FILE

First author, publication year, study reference*†	Country	Study design	Study population	Total no. of women	Outcomes measured	Specimen type; collection time	Diagnostic method	BV assessed
Donders, 2009[36]	Belgium	Cohort	Singleton, first antenatal visit between 9 -16 weeks with complete data available on *M. hominis* cultures, June 2000 - Dec 2001	759	PTB, SA	Vaginal swab; 1st, 2nd trimester	Culture	Yes
Embree, 1980[54]	Canada	Case-control	Single centre, deliveries between May 1977 - Jan 1978	554	SA, PND	Placenta; post-partum	Culture	NR
Farhadifar, 2016[11]	Iran	Case-control	Admitted into obstetrics and gynaecology wards, no antibiotics two weeks before sampling, no chronic disease, vaginal infection, Aug 2012-Jan 2013	218	SA	Endocervical swab; 1st, 2nd, 3rd trimester	NAAT	NR
Freitas, 2018[55]	Canada	Case-control	Spontaneous preterm birth and term deliveries, multicentre study	216	PTB	Vaginal swab; 2nd trimester	NAAT	NR
Gerber, 2003[37]	NR	Cohort	Transabdominal amniocentesis at 15-17 weeks gestation, singleton without complicated pregnancy	254	PTB, PROM	Amniotic fluid; 2nd trimester	NAAT	NR
Gonzàlez Bosquet, 2006[66]	NR	Case-control	Case: 24-34 weeks PTL, intact membranes; control: no history of preterm birth at same stage of delivery	250	PTB	Endocervical swab; NR	Culture	NR
Govender, 2009[38]	South Africa	Cohort	Low risk obstetric patients at first prenatal visit (16-23 weeks gestation)	199	PTB	Endocervical swab; 2nd trimester	NAAT	NR

ONLINE SUPPLEMENTAL FILE

First author, publication year, study reference*†	Country	Study design	Study population	Total no. of women	Outcomes measured	Specimen type; collection time	Diagnostic method	BV assessed
Grattard, 1995[71]	France	Cross-sectional	Women who delivered between Feb - May 1993 in obstetrical ward and their neonates; single centre study	208	PTB, LBW PROM,	Endocervical swab; post-partum	Culture	NR
Harada, 2008[56]	Japan	Case-control	Premature and term deliveries, Jan 2006 - July 2007	145	PTB	Endocervical swab; 2nd, 3rd trimester	NAAT, Culture	NR
Harrison, 1983[39]	USA	Cohort	Enrolled at their first prenatal visit; single centre study	860	SA	Endocervical swab; 1st, 2nd, 3rd trimester	Culture, ELISA	NR
Hillier, 1988[57]	USA	Case-control	Age >16 years, no antibiotics in previous 2 weeks, no known fetal anomaly, June 1984 - June 1985	112	PTB	Placenta; post-partum	Culture	Yes
Hillier, 1995[33]	USA	Cohort	> 16 years, singleton pregnancies at routine prenatal visits (23 to 26 weeks), between 1984 -1989	10 397	PTB	Endocervical swab; 2nd trimester	Culture	Yes
Holst, 1994[58]	Sweden	Case-control	Women presenting in PTL; controls were women with no pregnancy history	87	PTB	Endocervical swab; 3rd trimester	Culture	Yes
Jones, 2009[10]	United Kingdom	Case-control	Cases: <32 weeks gestation; control: >37 weeks gestation; single centre study	74	PTB, PROM	Placenta; post-partum	NAAT	NR
Kacerovsky, 2009[68]	NR	Case-control	Pregnancy with PPROM, Jan 2004 - Feb 2007; single centre study	450	PROM	Endocervical swab; 2nd, 3rd trimester	Culture	NR

Jonduo ME, *et al. BMJ Open* 2023; 12:e062990. doi: 10.1136/bmjopen-2022-062990

ONLINE SUPPLEMENTAL FILE

First author, publication year, study reference*†	Country	Study design	Study population	Total no. of women	Outcomes measured	Specimen type; collection time	Diagnostic method	BV assessed
Kataoka, 2006[40]	Japan	Cohort	Singleton pregnancies at <11 weeks of gestation, Jan - Dec 2002; single centre study	1040	PTB, PROM, SA, PND	Vaginal swab;1st trimester	NAAT	NR
Koucky, 2016[41]	Czech Republic	Cohort	Threatened premature deliveries, between Aug 2012 - Feb 2013	63	PTB	Vaginal swab; 2nd, 3rd trimester	NAAT	NR
Kumar, 2006[69]	India	Case-control	Women in spontaneous premature/ term labour with or without rupture of membrane; single centre study	120	PTB	Vaginal swab; 3rd trimester	Culture	Yes
Kundsin, 1984[72]	USA	Cross-sectional	Deliveries between Nov 1978 - Jun 1981; single centre study	801	PND	Placenta; post-partum	Culture	NR
Kwak, 2014[50]	South Korea	Cohort	Women with spontaneous premature labour or preterm PROM, Dec 2005 - Apr 2007; single centre study	179	PTB	Vaginal swab; 3rd trimester	Culture	NR
Lee, 2016[6]	South Korea	Cohort	Aged 15 - 47, delivered babies between Jun 2009 - May 2014; single centre study	1,035	PTB, PROM, SA	Vaginal swab; NR	Culture	NR
Luton, 1994[8]	Gabon	Cohort	Singleton pregnancy at <20 weeks gestation, Sept 1990 - Nov 1991	218	PTB, LBW, PND	Endocervical swab; 1st, 2nd trimester	Culture	NR
McCormack, 1975[73]	USA	Cross-sectional	Vaginal deliveries; single centre study	327	LBW, PND	Blood; post-partum	Culture	NR

ONLINE SUPPLEMENTAL FILE

First author, publication year, study reference*†	Country	Study design	Study population	Total no. of women	Outcomes measured	Specimen type; collection time	Diagnostic method	BV assessed
McDonald, 1992[59]	Australia	Case-control	Women who booked at one of 4 study centres, Oct 1986 - Dec 1988; multicentre study	2190	PTB, PROM	Endocervical swab; 2nd trimester	Culture	NR
McDonald, 1994[42]	Australia	Cohort	Patients attending the antenatal clinic, Oct 1986 - May 1990	560	PTB	Endocervical swab; 2nd, 3rd trimester	Culture	NR
Menard, 2010[43]	France	Cohort	Admitted preterm labour with no pregnancy related complications between July 2007 - July 2008	90	PTB	Vaginal swab; 2nd, 3rd trimester	NAAT	Yes
Minkoff, 1984[44]	USA	Cohort	Delivery between Mar - Sept 1982; single centre study	250	PTB, PROM	Vaginal swab; 1st, 2nd trimester	Culture	Yes
Mitsunari, 2005[60]	Japan	Case-control	Singleton pregnancy delivery, between Jan 2002 - Sept 2003	82	PTB, PROM	Endocervical swab; 2nd, 3rd trimester	NAAT	NR
Montenegro, 2019[70]	Colombia	Case-control	Pregnant women >18 years, no pregnancy related problems, non-smokers, no alcohol, no antibiotic	211	PTB, PROM	Placenta; post-partum	NAAT	NR
Munday, 1984[61]	United Kingdom	Case-control	Women admitted with vaginal bleeding before 28 weeks gestation and women attending one antenatal clinic at same hospital	241	SA	Endocervical swab; 2nd, 3rd trimester	Culture	NR

ONLINE SUPPLEMENTAL FILE

First author, publication year, study reference*†	Country	Study design	Study population	Total no. of women	Outcomes measured	Specimen type; collection time	Diagnostic method	BV assessed
Nasution, 2007[75]	NR	Cross-sectional	Women admitted with preterm PROM (<37weeks), normal vaginal deliveries at term, and women with post-partum fever	120	PROM	Placenta; post-partum	NAAT	NR
Nguyen, 2004[45]	Switzerland	Cohort	Women with transabdominal amniocentesis at 15 - 17 weeks gestation; single centre study	456	PTB, PROM, PND	Amniotic fluid; 2nd trimester	NAAT	NR
Odendaal, 2002[51]	South Africa	Cohort	Primigravid, first visit, 16 - 26 weeks with previous preterm labour or miscarriage, May - Dec 1996	395	PTB, SA, PND	Endocervical swab; 2nd trimester	Culture	Yes
Oliveira, 2020[7]	Brazil	Case-control	> 18 years old, cases: 8 - 20 weeks gestation; Control vaginal delivery at 38 - 40 weeks, Jul 2017 - Aug 2018	109	SA	Endocervical swab; 1st, 2nd, 3rd trimester	NAAT	NR
Payne, 2014[62]	China and Australia	Case-control	Singleton pregnancy referred for genetic amniocentesis	972	PTB	Amniotic fluid; 2nd trimester	NAAT	NR
Payne, 2016[46]	Australia	Cohort	Low risk singleton pregnancy, 18 -40 years old, at 1st or 2nd trimester when enrolled	191	PTB	Vaginal swab; 1st, 2nd, 3rd trimester	NAAT, Culture	NR
Payne, 2021[15]	Australia	Cohort	Nulliparous and multiparous, singleton pregnancy, ≥16 years between 12 - 23 weeks gestation	1000	PTB	Vaginal swab; 1st, 2nd trimester	NAAT	NR

Jonduo ME, *et al. BMJ Open* 2023; 12:e062990. doi: 10.1136/bmjopen-2022-062990

ONLINE SUPPLEMENTAL FILE

First author, publication year, study reference*†	Country	Study design	Study population	Total no. of women	Outcomes measured	Specimen type; collection time	Diagnostic method	BV assessed
Peretz, 2020[12]	Israel	Cohort	Women, 18 - 45 years, at any stage of labour and any mode of delivery, between Jun 2014 - Jan 2016	214	PTB, LBW	Vaginal swab; post-partum	NAAT	NR
Perni, 2004[52]	NR	Cohort	Singleton pregnancy: underwent transabdominal amniocentesis at 15-19 weeks with clear amniotic fluid	193	PTB, PROM	Amniotic fluid; 2nd trimester	NAAT	NR
Rittenschober-Böhm, 2018[47]	Austria	Cohort	Attending routine nuchal translucency screening between 12 - 14 weeks gestation; multicentre study	4330	PTB	Endocervical swab; 1st, 2nd trimester	NAAT	Yes
Schwab, 2015[53]	Indonesia	Cohort	2nd trimester, from Feb - Jun 2005; multicentre study	159	PTB	Vaginal swab; 2nd trimester	NAAT	Yes
Sperling, 1988[49]	USA	Cohort	Clinical diagnosis of intraamniotic infection, July 1979 - Dec 1986	409	LBW	Amniotic fluid; NR	Culture	NR
Sweeney, 2016[74]	USA	Cross-sectional	Term deliveries, no HIV infection, congenital infection, or fetal malformation, Jul 2010 - Apr 2013	535	PTB	Placenta; post-partum	NAAT, Culture	NR
Toth, 1992[63]	United Kingdom	Case-control	Admitted for delivery between Jan 1985 - Dec 1986	100	PTB	Endocervical swab; NR	Culture	NR
Usui, 2002[48]	Japan	Cohort	Singleton pregnancy attending first antenatal visit, Jan 1995 - Mar 1998	1958	PTB	Endocervical swab; 1st, 3rd trimester	Culture	NR

ONLINE SUPPLEMENTAL FILE

First author, publication year, study reference*†	Country	Study design	Study population	Total no. of women	Outcomes measured	Specimen type; collection time	Diagnostic method	BV assessed
Yoon, 2001[64]	South Korea	Case-control	Women who underwent mid-trimester amniocentesis	114	PTB, PROM	Amniotic fluid; 2nd trimester	NAAT	NR

LBW, infant low birth weight, defined as birth weight <2.5kg; NAAT, nucleic acid amplification tests; NR, not reported; PTB, preterm birth - defined as birth before 37 weeks gestation; PPROM, preterm premature rupture of membranes; PROM: premature rupture of membrane - defined as clinically confirmed rupture of membrane before 37weeks of gestation; PND, perinatal or neonatal death- defined as stillbirth (death after 20/40 gestation) or neonatal death (death <28 days since birth), unless otherwise defined by the study authors; SA: spontaneous abortion - defined as pregnancy loss at <20 weeks gestation or as defined by author; USA, United States of America.

* Study reference is the reference number cited in the main manuscript.

† Additional summary information about the characteristics of included studies in Tables S2.1-S2.3, S3.1-S3.3, S4.1-S4.3, S5

ONLINE SUPPLEMENTAL FILE

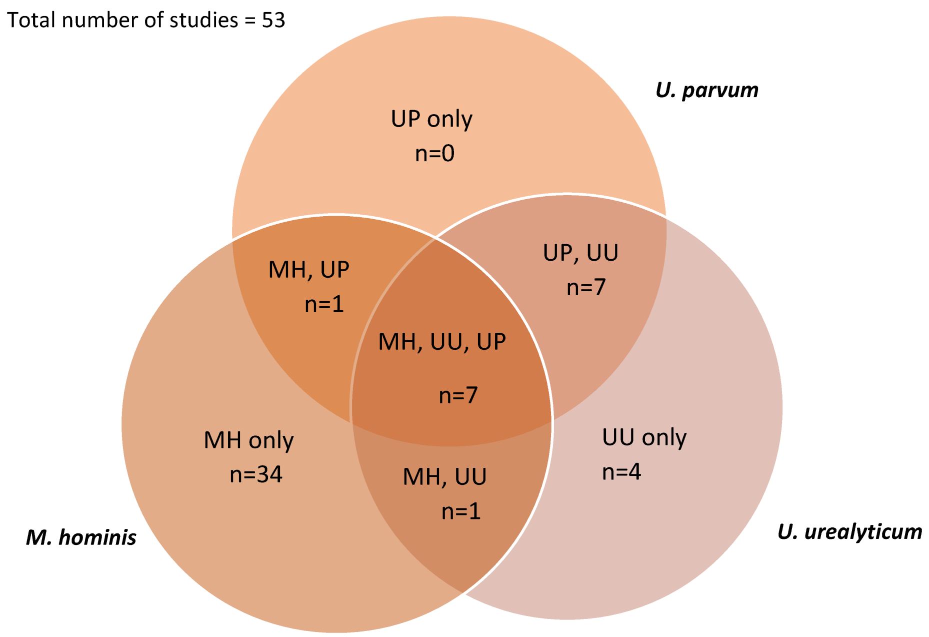

Figure S2 Venn diagram showing organisms reported in in the 53 articles included in the systematic review.

MH, *M. hominis*; UP, *U, parvum*; UU, *U. urealyticum*

Jonduo ME, *et al. BMJ Open* 2023; 12:e062990. doi: 10.1136/bmjopen-2022-062990

ONLINE SUPPLEMENTAL FILE

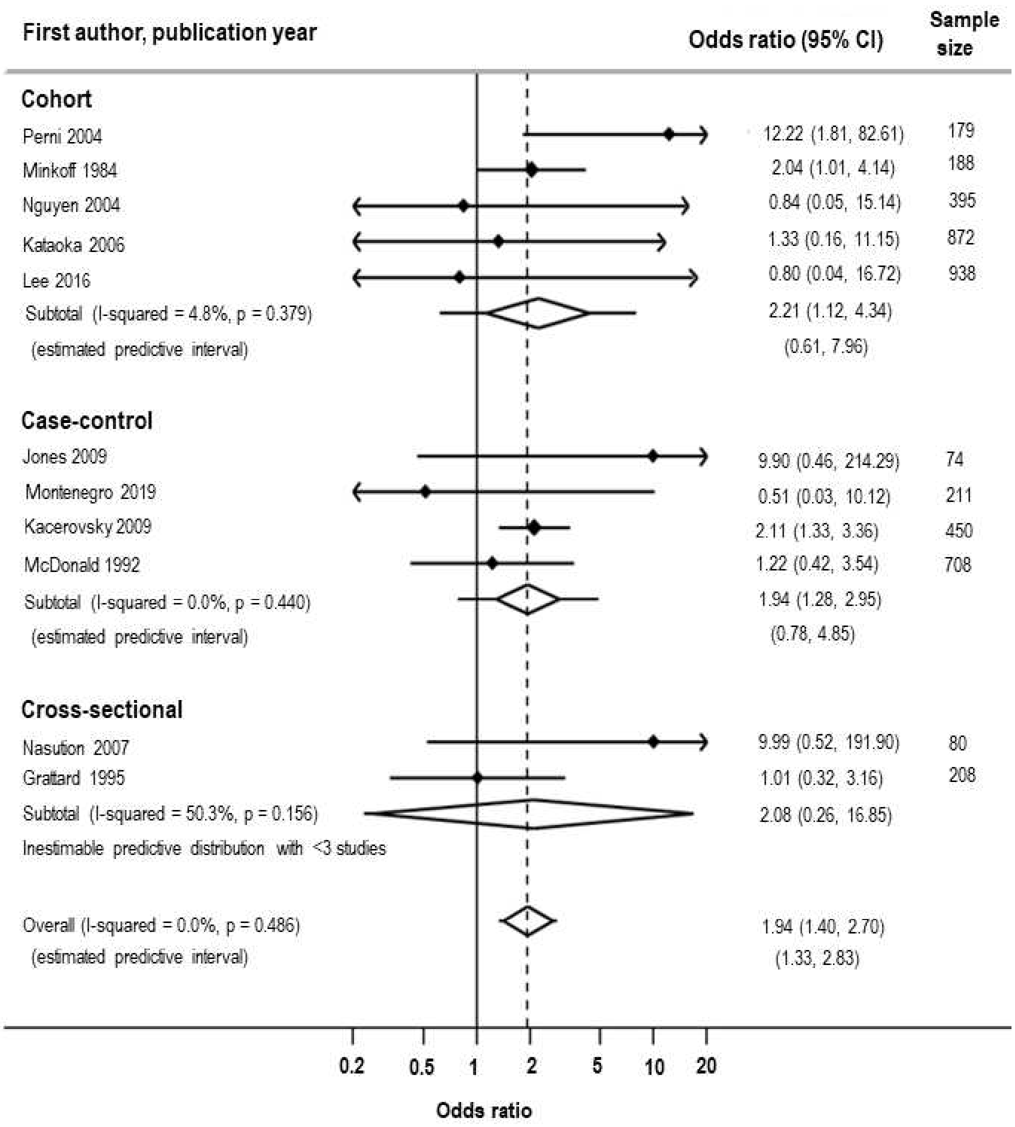

Figure S3.1 Forest plot of association between *M. hominis* and premature rupture of membrane, random effects model.

Solid diamond shows the point estimate and study weight. Solid line shows 95% confidence intervals (CI) for each study. The open diamond shows the point estimate and 95% CI of the summary estimate. Solid lines extending from the diamond show the prediction interval.

Jonduo ME, *et al. BMJ Open* 2023; 12:e062990. doi: 10.1136/bmjopen-2022-062990

ONLINE SUPPLEMENTAL FILE

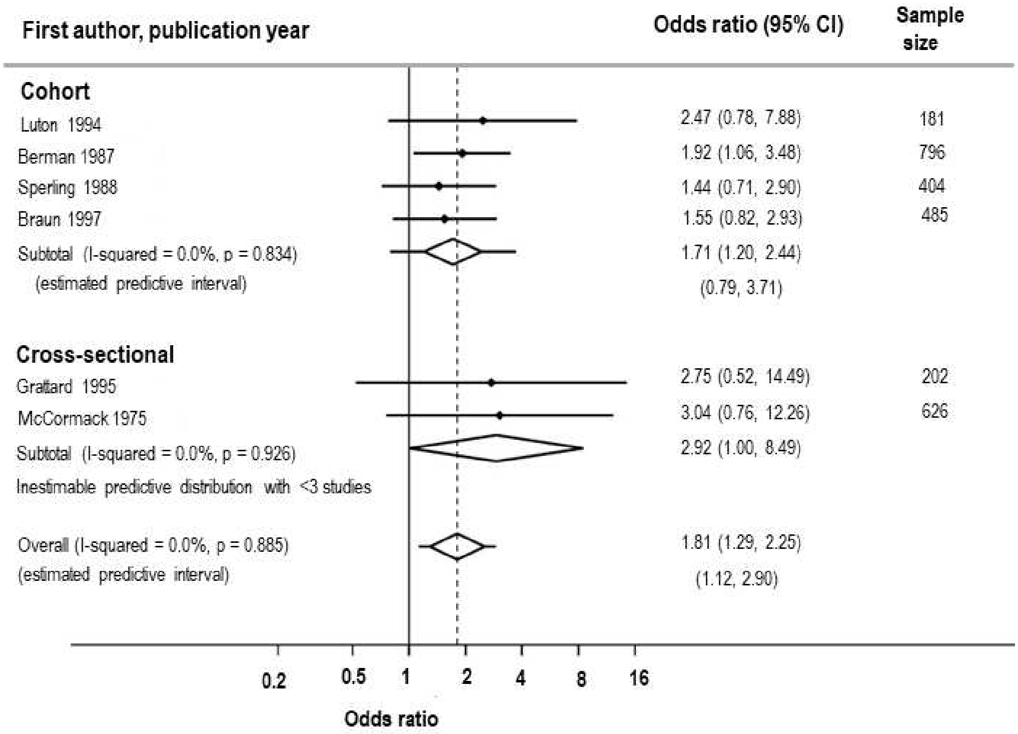

Figure S3.2 Forest plot of association between *M. hominis* and low birthweight, random effects model. Solid diamond shows the point estimate and study weight. Solid line shows 95% confidence intervals (CI) for each study. The open diamond shows the point estimate and 95% CI of the summary estimate. Solid lines extending from the diamond show the prediction interval.

ONLINE SUPPLEMENTAL FILE

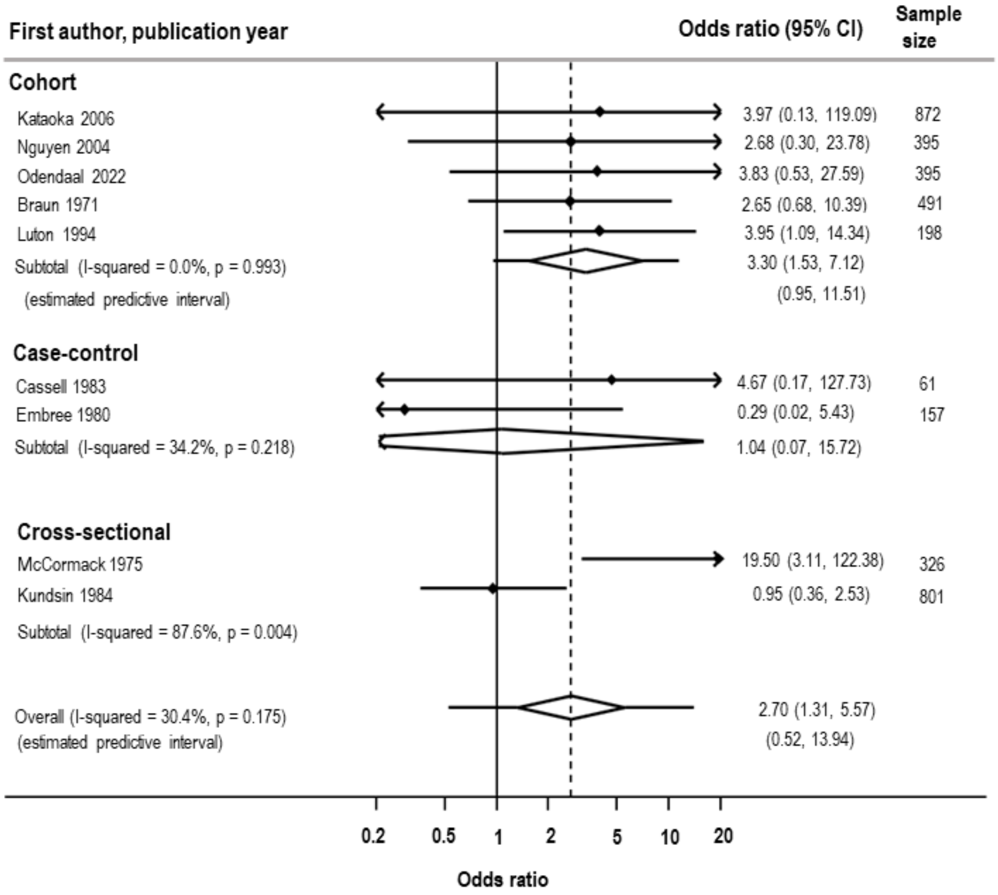

Figure S3.3 Forest plot of association between *M. hominis* and perinatal death random effects model. Solid diamond shows the point estimate and study weight. Solid line shows 95% confidence intervals (CI) for each study. The open diamond shows the point estimate and 95% CI of the summary estimate. Solid lines extending from the diamond show the prediction interval.

ONLINE SUPPLEMENTAL FILE

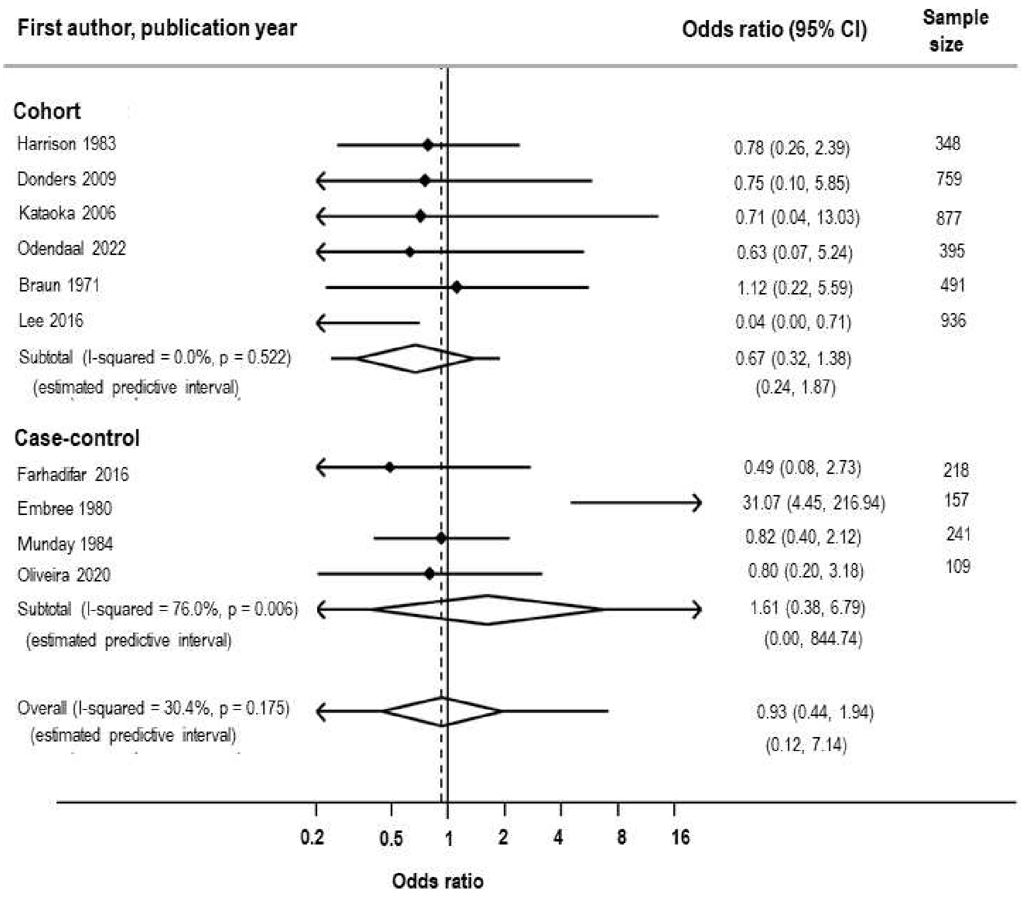

Figure S3.4 Forest plot of association between *M. hominis* and spontaneous abortion random effects model. Solid diamond shows the point estimate and study weight. Solid line shows 95% confidence intervals (CI) for each study. The open diamond shows the point estimate and 95% CI of the summary estimate. Solid lines extending from the diamond show the prediction interval.

ONLINE SUPPLEMENTAL FILE

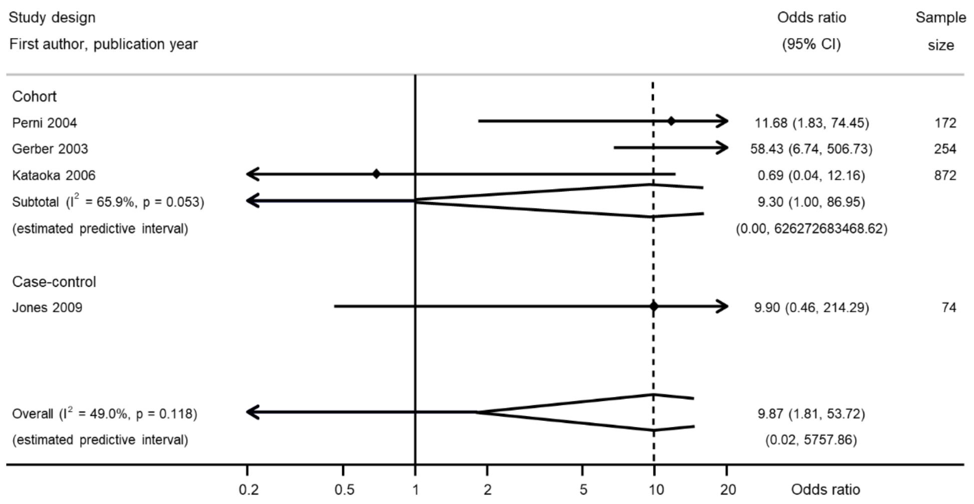

Figure S3.5 Forest plot of association between *U. urealyticum* and premature rupture of membrane, random effects model.
Solid diamond shows the point estimate and study weight. Solid line shows 95% confidence intervals (CI) for each study. The open diamond shows the point estimate and 95% CI of the summary estimate. Solid lines extending from the diamond show the prediction interval.

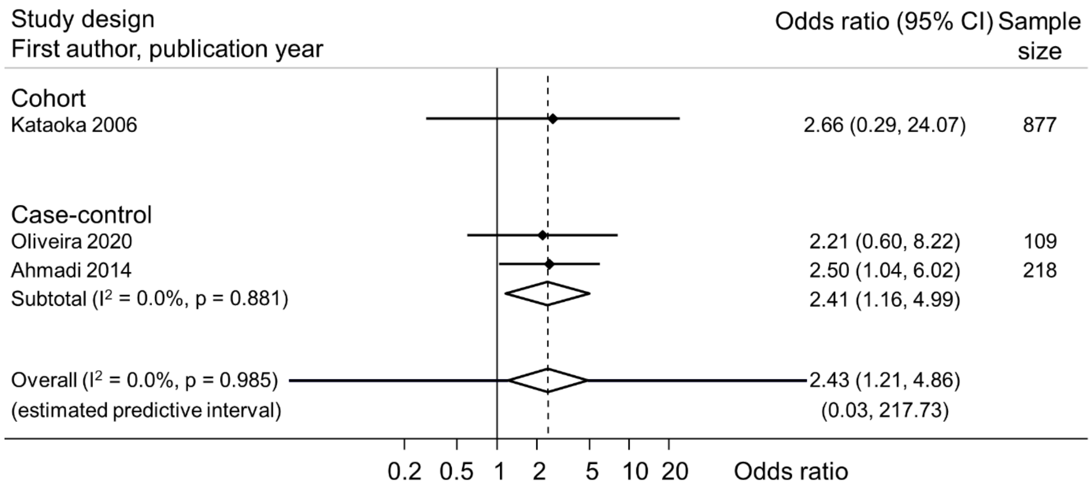

Figure S3.6 Forest plot of association between *U. urealyticum* and spontaneous abortion, random effects model.
Solid diamond shows the point estimate and study weight. Solid line shows 95% confidence intervals (CI) for each study. The open diamond shows the point estimate and 95% CI of the summary estimate. Solid lines extending from the diamond show the prediction interval.

ONLINE SUPPLEMENTAL FILE

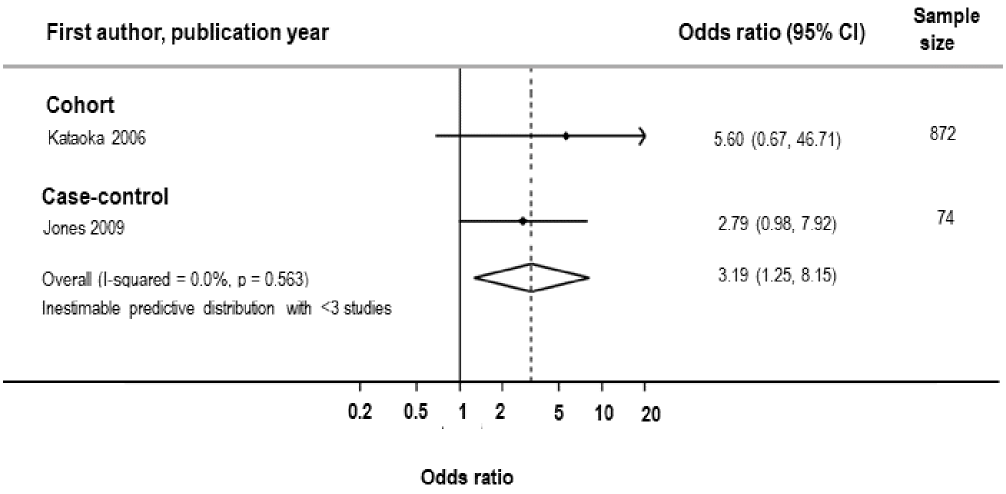

Figure S3.7 Forest plot of association between *U. parvum* and premature rupture of membrane, random effects model.
Solid diamond shows the point estimate and study weight. Solid line shows 95% confidence intervals (CI) for each study. The open diamond shows the point estimate and 95% CI of the summary estimate. Solid lines extending from the diamond show the prediction interval.

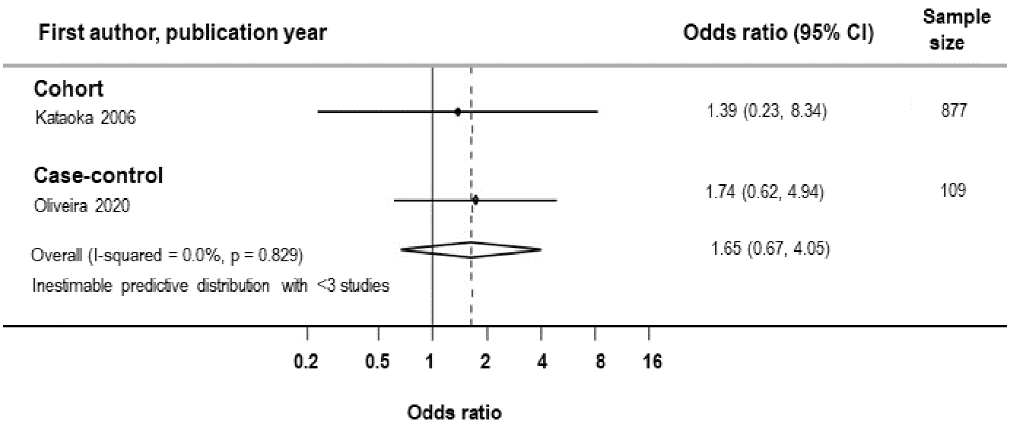

Figure S3.8 Forest plot of association between *U. parvum* and spontaneous abortion random effects model.
Solid diamond shows the point estimate and study weight. Solid line shows 95% confidence intervals (CI) for each study. The open diamond shows the point estimate and 95% CI of the summary estimate. Solid lines extending from the diamond show the prediction interval.

ONLINE SUPPLEMENTAL FILE

Table S2.1 Descriptive tables: cohort studies (n=26), in alphabetical order

First author, publication year, study reference	Organism reported	Outcomes	Definition provided	Effect size reported by study authors, OR or RR (95% CI)		
				Organism_ outcome	Unadjusted OR/RR	Adjusted OR, aOR
Agger, 2014[1]	MH<br>UU<br>UP	PTB	Born < 37 weeks	MH_PTB<br>UU_PTB<br>UP_PTB	OR 1.72 (0.91, 3.28)<br>OR 1.64 (0.67, 4.05)<br>OR 1.23 (0.7, 2.15)	'Final model… factors… from preliminary models with p>0.15.'<br>No organism in final multivariable model for PTB <37 weeks.<br>MH in final model PTB< 35 weeks, aOR 3.6 (1.4 - 9.7)
Berman, 1987[34]	MH	LBW	< 2.5kg	MH_LBW	RR 1.8 (1.0, 3.1)	Birth weight as continuous variable, p=0.01; adjusted for parity, maternal height, weight, marital status, age, enrolment, gestation, *C. trachomatis*
Braun, 1971[35]	MH	LBW<br>SA<br>PND	< 2.5kg<br>Not defined<br>Not defined	NR		No multivariable analysis
Donders, 2009[36]	MH	PTB<br>SA	Born < 37 weeks<br>*	MH_PTB	OR 8.5 (2.8, 25.5)	No multivariable analysis
Gerber, 2003[37]	UU	PTB<br>PROM	Born < 37 weeks<br>†	NR		No multivariable analysis
Govender, 2009[38]	MH, UU, UP	PTB	Born < 37 weeks	NR		No multivariable analysis
Harrison, 1983[39]	MH	SA	*	NR		No multivariable analysis
Hillier, 1995[33]	MH	PTB	Born < 37 weeks	MH_PTB		No multivariable analysis
Kataoka, 2006[40]	MH, UU, UP	PTB<br>PROM<br>SA<br>PND	Born < 37 weeks<br>Not defined<br>*<br>‡	NR		No multivariable analysis
Koucky, 2016[41]	UU	PTB	Born < 37 weeks	NR		aOR 3.4 (1.3, 5.5); adjusted for progesterone treatment, other factors not reported 5.46 (1.80, 16.62)
Kwak, 2014[50]	MH	PTB	Born < 37 weeks	NR		No multivariable analysis

ONLINE SUPPLEMENTAL FILE

First author, publication year, study reference	Organism reported	Outcomes	Definition provided	Effect size reported by study authors, OR or RR (95% CI)		
				Organism_ outcome	Unadjusted OR/RR	Adjusted OR, aOR
Lee, 2016[6]	MH	PTB<br>PROM<br>SA	Born < 37 weeks<br>Not defined<br>*	NR		No multivariable analysis
Luton, 1994[8]	MH	PTB<br>LBW<br>PND	Born < 37 weeks<br>< 2.5kg<br>‡	NR		No multivariable analysis
McDonald, 1994[42]	MH	PTB	Born < 37 weeks	NR		No multivariable analysis
Menard, 2010[43]	MH	PTB	Born < 37 weeks	NR		No multivariable analysis
Minkoff, 1984[44]	MH	PTB<br>PROM	Born < 37 weeks<br>†	NR		Stepwise multiple logistic regression. Results for MH not reported for either outcome
Nguyen, 2004[45]	MH	PTB<br>PROM<br>PND	Born < 37 weeks<br>†<br>Not defined	MH_PTB	RR 4.6 (1.7, 12.8)	No multivariable analysis
Odendaal, 2002[51]	MH	PTB<br>SA<br>PND	Born < 37 weeks<br>*<br>Not defined	NR		No multivariable analysis
Payne, 2016[46]	MH, UU, UP	PTB	Born < 37 weeks	NR		No multivariable analysis
Payne, 2021[15]	MH, UU, UP	PTB	Born < 37 weeks	NR		No multivariable analysis
Peretz, 2020[12]	UU, UP	PTB<br>LBW	Born < 37 weeks<br>< 2.5kg	NR		No multivariable analysis
Perni, 2004[52]	MH, UU	PTB<br>PROM	Born < 37 weeks<br>†	NR		No multivariable analysis

ONLINE SUPPLEMENTAL FILE

First author, publication year, study reference	Organism reported	Outcomes	Definition provided	Effect size reported by study authors, OR or RR (95% CI)		
				Organism_ outcome	Unadjusted OR/RR	Adjusted OR, aOR
Rittenschober-Böhm, 2018[47]	UU<br>UP	PTB	Born < 37 weeks	UU_PTB<br>UP_PTB	OR 1.4 (0.9, 2.3)<br>OR 1.7 (1.3, 2.2)	aOR 1.4 (0.8, 2.2)<br>aOR 1.6 (1.2, 2.1)<br>Adjusted for age, smoking, history of PTB, BV, smoking UU or UP
Schwab, 2015[53]	MH	PTB	Born < 37 weeks	MH_PTB	OR 0.26 (0.03, 1.13)	No multivariable analysis
Sperling, 1988[49]	MH	LBW	< 2.5kg	NR		No multivariable analysis
Usui, 2002[48]	MH	PTB	Born < 37 weeks	NR	OR not reported by study authors	aOR 1.5 (0.8, 2.7)<br>Adjusted for *Lactobacillus* spp., *E. coli*, glucose non-fermenting Gram-negative rods

LBW: low birth weight, defined as birth weight <2.5kg; NAAT, nucleic acid amplification tests; NR, not reported; MH, *M. hominis*; PND, perinatal death; PROM, premature rupture of membrane; PTB, preterm birth; SA: spontaneous abortion; UP, *U. parvum*; UU, *U. urealyticum*.

Study reference is the reference number cited in the main manuscript.

*SA defined as pregnancy loss at <20 weeks gestation or as defined by author;

†PROM defined as rupture of membrane before 37 completed weeks or as defined by author;

‡ Perinatal death, defined as birth after 20 weeks gestation (stillbirth) or death within 28 days after birth (neonatal death) unless otherwise defined by the study authors.

ONLINE SUPPLEMENTAL FILE

Table S2.2 Descriptive tables: case-control studies (n=22), in alphabetical order

First author, publication year, study reference	Organism reported	Outcome	Definition Provided	OR/ RR (95% CI) reported by study authors		
				Organism_ outcome	Unadjusted, OR	Adjusted, aOR
Ahmadi, 2014[9]	UU	SA	*	NR		No multivariable analysis
Cassell, 1983[32]	MH	PTB<br>PND	Born < 37 weeks<br>‡	NR		No multivariable analysis
Chua, 1999[67]	MH	PTB	Born < 37 weeks	NR		No multivariable analysis
Daskalakis, 2009,[65]	MH	PTB	Born < 37 weeks	NR		No multivariable analysis
Embree,1980[54]	MH	SA<br>PND	Not defined<br>Partially defined	NR		No multivariable analysis
Farhadifar, 2017[11]	MH	SA	*	MH_SA	OR 0.49 (0.08, 2.73)	No multivariable analysis
Freitas, 2018[55]	UU, UP	PTB	Born < 37 weeks	NR		No multivariable analysis
Gonzàlez-Bosquet, 2006[66]	MH	PTB	Born < 37 weeks	NR		No multivariable analysis
Harada, 2008[56]	UU,<br>UP	PTB	Not defined	UU_PTB<br>UP_PTB		No multivariable analysis
Hillier, 1988[57]	MH	PTB	Born < 37 weeks	NR		No multivariable analysis
Holst, 1994[58]	MH	PTB	Born < 37 weeks	NR		No multivariable analysis
Jones, 2009[10]	MH, UU, UP	PTB<br>PROM	Born < 37 weeks<br>†	NR		No multivariable analysis
Kacerovsky, 2009[68]	MH	PROM	†	NR		No multivariable analysis
Kumar, 2006[69]	MH	PTB	Born < 37 weeks			No multivariable analysis

ONLINE SUPPLEMENTAL FILE

First author, publication year, study reference	Organism reported	Outcome	Definition Provided	OR/ RR (95% CI) reported by study authors		
				Organism_ outcome	Unadjusted, OR	Adjusted, aOR
McDonald, 1992[59]	MH	PTB<br>PROM	Born < 37 weeks<br>Not defined	MH_PTB<br>MH_PROM	OR 1.7 (0.9, 3.5)<br>OR 1.5 (0.5, 4.3)	aOR 1.1 (0.5, 2.5)<br>aOR 1.1 (0.3, 3.7)<br>Adjusted for 'confounding demographic and obstetric variables'
Mitsunari, 2005[60]	UU,<br>UP	PTB	Not defined	UU_PTB<br>UP_PTB		No multivariable analysis
Montenegro, 2019[70]	MH	PTB<br>PROM	Born < 37 weeks<br>Not defined	NR		No multivariable analysis
Munday, 1984[61]	MH	SA	Not defined	NR		No multivariable analysis
Oliveira, 2020[7]	MH<br>UU<br>UP	SA	*	MH_SA<br>UU_SA<br>UP_SA	OR 0.08 (0.2, 3.17)<br>OR 2.21 (0.6, 8.22)<br>OR 1.74 (0.61, 4.93)	No multivariable analysis
Payne, 2014[62]	UP	PTB	Born < 37 weeks			NR
Toth, 1992[63]	MH	PTB	Born < 37 weeks	NR		No multivariable analysis
Yoon, 2001[64]	UU	PTB<br>PROM	Born < 37 weeks<br>Not defined	NR		No multivariable analysis

LBW: low birth weight defined as birth weight <2.5kg; NAAT, nucleic acid amplification tests; NR, not reported; MH, *M. hominis*; PND, perinatal death; PROM, premature rupture of membrane; PTB, preterm birth; SA: spontaneous abortion; UP, *U. parvum*; UU, *U. urealyticum*.

Study reference is the reference number cited in the main manuscript.

*SA defined as pregnancy loss at <20 weeks gestation or as defined by author;

†PROM defined as rupture of membrane before 37 completed weeks or as defined by author;

‡ Perinatal death, defined as birth after 20 weeks gestation (stillbirth) or death within 28 days after birth (neonatal death) unless otherwise defined by the study authors.

ONLINE SUPPLEMENTAL FILE

Table S2.3 Descriptive tables: cross-sectional studies (n=5), in alphabetical order

First author, publication year, study reference*	Organism reported	Total enrolled	Outcome	Definition Provided	OR/ RR (95% CI) reported by study authors		
					Organism_ outcome	Unadjusted, OR	Adjusted, aOR
Grattard, 1995[71]	MH	208	PTB,<br>PROM<br>LBW	Born < 37 weeks<br>†<br>< 2.5kg	NR		No multivariable analysis
Kundsin, 1984[72]	MH	801	PND	‡	NR		No multivariable analysis
McCormack, 1975[73]	MH	327	LBW<br>PND	< 2.5kg<br>Not defined	NR		No multivariable analysis
Nasution, 2007[75]	MH	120	PROM	Not defined	NR		No multivariable analysis
Sweeney, 2016[74]	UU, UP	535	PTB	Born < 37 weeks	NR		No multivariable analysis

LBW: low birth weight defined as birth weight <2.5kg; NAAT, nucleic acid amplification tests; NR, not reported; MH, *M. hominis*; PND, perinatal death; PROM, premature rupture of membrane; PTB, preterm birth; SA: spontaneous abortion; UP, *U. parvum*; UU, *U. urealyticum.*

* Study reference is the reference number cited in the main manuscript;

†PROM defined as rupture of membrane before 37 completed weeks or as defined by author;

‡ Perinatal death, defined as birth after 20 weeks gestation (stillbirth) or death within 28 days after birth (neonatal death) unless otherwise defined by the study authors.

ONLINE SUPPLEMENTAL FILE

Table S3.1 Summary description of studies reporting *M. hominis* (n=42) in alphabetical order, by country-level income status

First author, publication year, country, study reference	Study design	Gestational age at assessment	Sample size for outcome of interest<br>Number of adverse outcomes in women with *M. hominis*/ total number of women with adverse outcome (%)					NICE checklist criteria fulfilled*
			PTB	LBW	PROM	SA	PND	
**High-income country†**								
Agger, 2014,[1] USA	Cohort	NR	676<br>14/54 (26)					+/+
Braun, 1971,[35] USA	Cohort	LMP		485<br>24/42 (57)		491<br>3/6 (50)	491<br>7/10 (70)	+/-
Cassell, 1983,[32] USA	Case-control	US	61<br>1/10 (10)				61<br>0/3 (0)	+/-
Donders, 2009,[36] Belgium	Cohort	US	744<br>5/50 (10)			759<br>1/15 (7)		+/+
Embree, 1980,[54] Canada	Case-control	LMP, NN assessment				157<br>3/10 (30)	157<br>0/39 (0)	-/-
Grattard, 1995,[71] France	Cross-sectional	NR	193<br>3/8 (38)	202<br>2/8 (25)	208<br>4/36 (11)			-/+
Harrison, 1983,[39] USA	Cohort	NR				348<br>4/22 (18)		-/-
Hillier, 1988,[57] USA	Case-control	US, FH, LMP	112<br>3/38 (8)					+/+
Hillier, 1995,[33] USA	Cohort	LMP	9105<br>161/423 (38)					
Holst, 1994,[58] Sweden	Case-control	US, LMP	87<br>10/22 (45)					++/+
Jones, 2009,[10] United Kingdom	Case-control	NR	74<br>2/53 (4)		74<br>2/26 (8)			-/-
Kataoka, 2006,[40] Japan	Cohort	US, LMP	872<br>4/16 (25)		872<br>1/7 (14)	877<br>0/5 (0)	872<br>0/1 (0)	+/+

ONLINE SUPPLEMENTAL FILE

First author, publication year, country, study reference	Study design	Gestational age at assessment	Sample size for outcome of interest<br>Number of adverse outcomes in women with *M. hominis*/ total number of women with adverse outcome (%)					NICE checklist criteria fulfilled*
			PTB	LBW	PROM	SA	PND	
Kundsin, 1984,[72] USA	Cross-sectional	NR					801<br>5/29 (17)	-/+
Kwak, 2014,[50] South Korea	Cohort	NR	112<br>13/86 (15)					+/+
Lee, 2016,[6] South Korea	Cohort	NR	466<br>1/141 (<1)		466<br>0/187 (0)	466<br>0/11 (0)		-/-
McCormack, 1975,[73] USA	Cross-sectional	NR		326<br>3/42 (7)			326<br>2/6 (33)	+/-
McDonald, 1992,[59] Australia	Case-control	LMP, US	786<br>11/135 (8)		708<br>4/57 (8)			-/-
McDonald, 1994,[42] Australia	Cohort	US, LMP	337<br>7/45 (16)					-/-
Menard, 2010,[43] France	Cohort	US, LMP	90<br>6/36 (17)					-/-
Minkoff, 1984,[44] USA	Cohort	NR	201<br>10/18 (56)		188<br>21/40 (53)			-/-
Munday, 1984,[61] United Kingdom	Case-control	NR				241<br>9/76 (12)		+/-
Nguyen, 2004,[45] Switzerland	Cohort	NR	395<br>3/10 (30)		365<br>0/7 (0%)		395<br>1/6 (17)	+/+
Payne, 2016,[46] Australia	Cohort	NR	187<br>2/13 (15)					+/+
Payne, 2021,[15] Australia	Cohort	NR	1000<br>9/118 (8)					+/+
Sperling, 1988,49 USA	Cohort	NR		404<br>14/37 (38)				-/-
Toth, 1992,[63] United Kingdom	Case-control	NR	80<br>3/39 (8)					-/-

ONLINE SUPPLEMENTAL FILE

First author, publication year, country, study reference	Study design	Gestational age at assessment	Sample size for outcome of interest<br>Number of adverse outcomes in women with *M. hominis*/ total number of women with adverse outcome (%)					NICE checklist criteria fulfilled*
			PTB	LBW	PROM	SA	PND	
Usui, 2002,[48] Japan	Cohort	LMP	1958<br>15/342 (4)					+/-
**Upper-middle income†**								
Berman, 1987,[34] Mexico	Cohort	NR		796<br>28/48 (58)				-/+
Chua, 1999,[67] Malaysia	Case-control	LMP, NN assessment	120<br>9/60 (15)					+/+
Farhadifar, 2016,[11] Iran	Case-control	US/LMP				218<br>2/109 (2)		+/+
Govender, 2009,[38] South Africa	Cohort	NR	199<br>11/20 (55)					-/-
Luton, 1994,[8] Gabon	Cohort	US, LMP	181<br>11/20 (55)	181<br>8/13 (62)			198<br>5/10 (50)	-/-
Montenegro,[70] 2019, Colombia	Case-control	NR	211<br>1/84 (1)		211<br>0/3 (0)			+/+
Odendaal, 2002,[51] South Africa	Cohort	US	395<br>33/119 (28)			395<br>1/7 (14)	395<br>2/4 (50)	+/-
Oliveria, 2020,[7] Brazil	Case-control	NR				109<br>11/89 (12)		+/+
**Lower-middle/low income†**								
Schwab,2015,[53] Indonesia	Cohort	LMP	62<br>2/23 (9)					-/-
Kumar, 2006,[69] India	Case-control	NR	120<br>4/60 (7)					+/+
**Country not reported**								
Gonzàlez Bosquet, 2006[66]	Case-control	US	120					+/+

ONLINE SUPPLEMENTAL FILE

First author, publication year, country, study reference	Study design	Gestational age at assessment	Sample size for outcome of interest<br>Number of adverse outcomes in women with *M. hominis*/ total number of women with adverse outcome (%)					NICE checklist criteria fulfilled*
			PTB	LBW	PROM	SA	PND	
			0/70 (0)					
Daskalakis, 2009[65]	Case-control	US, LMP	37<br>8/25 (32)					+/+
Kacerovsky, 2009[68]	Case-control	NR			450<br>63/225 (28)			-/-
Nasution, 2007[75]	Cross-sectional	NR			80<br>4/40 (10)			-/-
Perni, 2004[52]	Cohort	NR	179<br>0/10 (0)		179<br>2/5 (40)			+/+

MH, *Mycoplasma hominis*; UU, *Ureaplasma urealyticum*; UP, *Ureaplasma parvum*; NR, not reported; USA, United States of America.

LBW: low birth weight, defined as birth weight <2.5kg ; PTB: preterm birth, defined as delivery before 37weeks gestation; PROM: premature rupture of membranes, - defined as clinically confirmed rupture of membrane before 37weeks gestation; PND, perinatal death, defined as stillbirth (death after 20/40 gestation) or neonatal death (death <28 day since birth), unless otherwise defined by the study authors; SA: spontaneous abortion- defined as pregnancy loss at <20/40 or as defined by author.

Study reference is the reference number cited in the main manuscript.

* UK National Institute of Health and Care Excellence (NICE) checklist, summary of criteria for internal/external validity: +/+ Some checklist criteria fulfilled; -/-Few or no checklist criteria fulfilled. Detailed assessment reported by study design in Tables S6.1-S6.3.

† High-income ($12,376 or more); Upper-middle income ($3,996 to $1,2375); Lower-middle-income ($1,025 to $3,995); Low-income ($1,025 or less) (Source: World Bank, Gross national income per capita, 2019-2020 https://datatopics.worldbank.org/world-development-indicators/the-world-by-income-and-region.html).

ONLINE SUPPLEMENTAL FILE

Table S3.2 Summary description of studies reporting *U. urealyticum* (n=18) in alphabetical order, by country-level income status

First author, publication year, country, study reference	Study design	Gestational age at assessment	Sample size for outcome of interest<br>Number of adverse outcomes in women with *U. urealyticum*/ total number of women with adverse outcome (%)					NICE checklist criteria fulfilled*
			PTB	LBW	PROM	SA	PND	
**High-income country**†								
Agger, 2014,[1] USA	Cohort	NR	676<br>4/54 (11)					+/+
Freitas, 2018,[55] Canada	Case-control	NR	216<br>0/46 (0)					+/+
Harada, 2008,[56] Japan	Case-control	NR	145<br>0/45 (0)					+/+
Jones, 2009,[10] United Kingdom	Case-control	NR	74<br>2/53 (4)		74<br>2/26 (8)			-/-
Kataoka, 2006,[40] Japan	Cohort	US, LMP	872<br>0/16 (0)		872<br>0/7 (0)	877<br>1/5 (20)	872<br>0/1 (0)	+/+
Koucky, 2016,[41] Czech Republic	Cohort	US, LMP	63<br>17/29 (59)					+/+
Mitsunari, 2005,[60] Japan	Case-control	NR	82<br>2/21 (10)					+/+
Payne, 2016,[46] Australia	Cohort	NR	187<br>2/13 (15)					+/+
Payne, 2021,[15] Australia	Cohort	NR	1000<br>14/118 (12)					+/+
Peretz, 2020,[12] Israel	Cohort	NR	214<br>3/5 (60)	214<br>1/3 (33)				-/-

ONLINE SUPPLEMENTAL FILE

First author, publication year, country, study reference	Study design	Gestational age at assessment	Sample size for outcome of interest Number of adverse outcomes in women with *U. urealyticum*/ total number of women with adverse outcome (%)					NICE checklist criteria fulfilled*
			PTB	LBW	PROM	SA	PND	
Rittenschober-Böhm, 2018,[47] Austria	Cohort	US	2183<br>19/146 (13)					+/+
Sweeney, 2016,[74] USA	Cross-sectional	NR	535<br>6/443 (1)					+/-
Yoon, 2001,[64] South Korea	Case-control	NR	114<br>3/19 (16)		Missing data<br>2/9 (22)**			+/+
**Upper-middle income†**								
Ahmadi, 2014,[9] Iran	Cohort	US, LMP				218<br>18/109 (17)		+/+
Govender, 2009,[38] South Africa	Cohort	NR	199<br>5/20 (25)					-/-
Oliveira, 2020,[7] Brazil	Case-control	NR				109<br>25/89 (28)		+/+
**Country not reported**								
Gerber, 2003,[37]	Cohort	NR	254<br>9/10 (90)		254<br>6/7 (86)			+/-
Perni, 2004[52]	Cohort	Cohort	172<br>0/10 (0)		172<br>3/5 (60)			+/+

MH, *Mycoplasma hominis*; UU, *Ureaplasma urealyticum*; UP, *Ureaplasma parvum*; NR, not reported; USA, United States of America.

LBW: low birth weight, defined as birth weight <2.5kg ; PTB: preterm birth, defined as delivery before 37weeks gestation; PROM: premature rupture of membranes, - defined as clinically confirmed rupture of membrane before 37weeks gestation; PND, perinatal death, defined as stillbirth (death after 20/40 gestation) or neonatal death (death <28 day since birth), unless otherwise defined by the study authors; SA: spontaneous abortion- defined as pregnancy loss at <20/40 or as defined by author.

Study reference is the reference number cited in the main manuscript.

ONLINE SUPPLEMENTAL FILE

* UK National Institute of Health and Care Excellence (NICE) checklist, summary of criteria for internal/external validity: +/+ Some checklist criteria fulfilled; -/-Few or no checklist criteria fulfilled. Detailed assessment reported by study design in Tables S6.1-S6.3.

† High-income ($12,376 or more); Upper-middle income ($3,996 to $1,2375); Lower-middle-income ($1,025 to $3,995); Low-income ($1,025 or less) (Source: World Bank, Gross national income per capita, 2019-2020 https://datatopics.worldbank.org/world-development-indicators/the-world-by-income-and-region.html).

ONLINE SUPPLEMENTAL FILE

Table S3.3 Summary description of studies reporting *U. parvum* (n=14) in alphabetical order

First author, publication year, country, study reference	Study design	Gestational age at assessment	Sample size for outcome of interest<br>Number of adverse outcomes in women with *U. parvum*/ total number of women with adverse outcome (%)					NICE checklist criteria fulfilled*
			**PTB**	**LBW**	**PROM**	**SA**	**PND**	
**Upper-middle and high-income country†**								
Agger, 2014,[1]<br>USA	Cohort	NR	676<br>29/54 (54)					+/+
Freitas, 2018,[55]<br>Canada	Case-control	NR	216<br>14/46 (30)					+/-
Govender, 2009,[38]<br>South Africa	Cohort	NR	199<br>10/20 (50)					-/-
Harada, 2008,[56]<br>Japan	Case-control	NR	145<br>23/45 (51)					+/+
Jones, 2009,[10]<br>United Kingdom	Case-control	NR	74<br>19/53 (36)		74<br>11/26 (42)			-/-
Kataoka, 2006,[40]<br>Japan	Cohort	US, LMP	872<br>4/16 (25)		872<br>6/7 (86)	877<br>3/5 (60)	872<br>1/1 (100)	+/+
Mitsunari, 2005,[60]<br>Japan	Case-control	NR	82<br>16/21 (76)					+/+
Oliveira, 2020,[7]<br>Brazil	Case-control	NR				109<br>68/89 (76)		+/+
Payne, 2014,[62]<br>China & Australia	Case-control	NR	972<br>2/115 (2)					+/+
Payne, 2016,[46]<br>Australia	Cohort	NR	187<br>10/13 (77)					+/+

ONLINE SUPPLEMENTAL FILE

Payne, 2021,[15] Australia	Cohort	NR	1000<br>56/118 (48)		+/+
Peretz, 2020,[12]<br>Israel	Cohort	NR	214<br>1/5 (20)	214<br>1/3 (33)	-/-
Rittenschober-Böhm, 2018,[47] Austria	Cohort	US	3316<br>140/267 (52)		+/+
Sweeney, 2016,[74]<br>USA	Cross-sectional	NR	535<br>27/443 (4)		+/-

MH, *Mycoplasma hominis*; UU, *Ureaplasma urealyticum*; UP, *Ureaplasma parvum*; NR, not reported; UK, United Kingdom; USA, United States of America.

LBW: low birth weight, defined as birth weight <2.5kg ; PTB: preterm birth, defined as delivery before 37weeks gestation; PROM: premature rupture of membranes, - defined as clinically confirmed rupture of membrane before 37weeks gestation; PND, perinatal death, defined as stillbirth (death after 20/40 gestation) or neonatal death (death <28 day since birth), unless otherwise defined by the study authors; SA: spontaneous abortion- defined as pregnancy loss at <20/40 or as defined by author.

Study reference is the reference number cited in the main manuscript.

* UK National Institute of Health and Care Excellence (NICE) checklist, summary of criteria for internal/external validity: +/+ Some checklist criteria fulfilled; -/-Few or no checklist criteria fulfilled. Detailed assessment reported by study design in Tables S6.1-S6.3.

† High-income ($12,376 or more); Upper-middle income ($3,996 to $1,2375) (Source: World Bank, Gross national income per capita, 2019-2020 https://datatopics.worldbank.org/world-development-indicators/the-world-by-income-and-region.html).

Jonduo ME, *et al. BMJ Open* 2023; 12:e062990. doi: 10.1136/bmjopen-2022-062990

ONLINE SUPPLEMENTAL FILE

Table S4.1 Study setting and socio-demographics, cohort studies (n=26), in alphabetical order

First author, publication year, study reference*	Study location	Study setting	Urban/ rural location	Mean†/ median age years (range)	Ethnicity	Other infections included/(excluded)	Smokers included (%)	Multiple pregnancies
Agger, 2014[1]	USA	NR/unclear;	Mixed	NR	Mixed	CT, NG, MG, syphilis, HPV, herpes,	NR	Yes
Berman, 1987[34]	Mexico	Health facility	NR/ unclear	NR	NR	CT	NR	No
Braun, 1971[35]	USA	Health facility	Urban	NR	Mixed	NR	NR	NR
Donders, 2009[36]	Belgium	Health facility	Urban	29†	Mixed	BV (CT, NG, TV, syphilis)	120/761 (15.8%)	No
Gerber, 2003[37]	NR	Health facility	NR/ unclear	(19 – 42)	White	NR	NR	Yes
Govender, 2009[38]	South Africa	Health facility	Urban	NA	NR	CT, MG, HIV	NR	NR
Harrison, 1983[39]	USA	Health facility	Urban	NR	Mixed	CT	NR	Yes
Hillier, 1995[33]	USA	Health facility	Urban	NR	Mixed	CT, NG, TV, BV	Yes but number/% NR	No
Kataoka, 2006[40]	Japan	Health facility	Urban	28.9†	NR	CT, NG, MG	NR	No
Koucky, 2016[41]	Czech Republic	Health facility	Urban	31	NR	NR	NR	No
Kwak, 2014[50]	South Korea	Health facility	Urban	30.7	NR	NR	NR	No
Lee, 2016[6]	South Korea	Health facility	Urban	31 (15 - 47)	NR	NR	NR	NR

Jonduo ME, et al. BMJ Open 2023; 12:e062990. doi: 10.1136/bmjopen-2022-062990

ONLINE SUPPLEMENTAL FILE

First author, publication year, study reference*	Study location	Study setting	Urban/ rural location	Mean†/ median age years (range)	Ethnicity	Other infections included/(excluded)	Smokers included (%)	Multiple pregnancies
Luton, 1994[8]	Gabon	Health facility	NR/ unclear	NR	NR	CT, NG, TV, syphilis, HIV	NR	No
McDonald, 1994[42]	Australia	Health facility	NR	NR	NR	CT, TV	NR	NR
Menard, 2010[43]	France	Health facility	Urban	NR	NR	BV	NR	No
Minkoff, 1984[44]	USA	Health facility	NR	27† (17 - 39)	Mixed	CT, TV, HSV, nonspecific vaginitis/BV	NR	Yes
Nguyen, 2004[45]	Switzerland	Health facility	Urban	19 - 42	NR	NR	NR	No
Odendaal, 2002[51]	South Africa	Health facility	Urban	NR	NR	CT, NG, BV	161/395 (40.8%)	No
Payne, 2016[46]	Australia	Health facility	Urban	30 (18 - 43)	Mixed	NR	21/191 (11%)	No
Payne, 2021[15]	Australia	Health facility	Urban	NR	Mixed	MG, (HIV)	135/1000 (13.5%)	No
Peretz, 2020[12]	Israel	Health facility	Urban	29.8†	Mixed	MG	NR	Yes
Perni, 2004[52]	NR	Health facility	NR/ unclear	18 - 44	Mixed	NR	NR	No
Rittenschober-Böhm, 2018[47]	Austria	Health facility	Urban	30.3†	NR	BV	670/3643 (18.4%)	No

ONLINE SUPPLEMENTAL FILE

First author, publication year, study reference*	Study location	Study setting	Urban/ rural location	Mean†/ median age years (range)	Ethnicity	Other infections included/(excluded)	Smokers included (%)	Multiple pregnancies
Schwab, 2015[53]	Indonesia	Health facility	Urban	26.6† (17- 42)	NR	CT, NG, BV	NR	NR
Sperling, 1988[49]	USA	Health facility	Urban	NR	Mixed	NR	NR	NR
Usui, 2002[48]	Japan	Health facility	Urban	NR	Asian	CT	NR	No

BV, bacterial vaginosis; CT, *Chlamydia trachomatis*; HIV, human immunodeficiency virus; HPV, human papilloma virus; HSV, herpes simples virus; MG, *Mycoplasma genitalium*; NG, *Neisseria gonorrhoeae*; NR, not reported; No, did not include multiple pregnancy; Yes, included multiple pregnancies; TV, *Trichomonas vaginalis*; USA, United States of America.

NR/ unclear, it was not clearly reported where the study setting was located.

* Study reference is the reference number cited in the main manuscript

†Reported mean age.

Jonduo ME, *et al. BMJ Open* 2023; 12:e062990. doi: 10.1136/bmjopen-2022-062990

ONLINE SUPPLEMENTAL FILE

Table S4.2 Study setting and socio-demographics, case-control studies (n=22), in alphabetical order

First author, Publication year, study reference*	Study location	Study setting	Urban /rural location	Mean†/ median age years (range)	Ethnicity	Other infections included/(excluded)	Smokers included (%)	Multiple pregnancies
Ahmadi, 2014[9]	Iran	Health facility	Urban	19 - 43	NR	NR	3/218 (1.4)	NR
Cassell, 1983[32]	USA	Health facility	Urban	NR	White, Black	NR	NR	NR
Chua, 1999[67]	Malaysia	Health facility	Urban	NR	NR	NR	NR	No
Daskalakis, 2009,[65]	NR	Health facility	Urban	NR	NR	NR	36/144 (25)	No
Embree,1980[54]	Canada	Health facility	Urban	14-45	NR	NR	NR	Yes
Farhadifar, 2017[11]	Iran	Health facility	Urban	25 (19 - 43)	NR	NR	NR	NR
Freitas, 2018[55]	Canada	Health facility	Urban	33† (21 - 45)	Mixed	NR	4/216 (2.3%)	NR
Gonzàlez- Bosquet, 2006[66]	NR	Health facility	NR/ unclear	NR	NR	TV, BV-associated bacteria, CA, *E. coli*, GBS,	NR	No
Harada, 2008[56]	Japan	Health facility	Urban	NR	NR	NR	NR	No
Hillier, 1988[57]	USA	Health facility	Urban	NR	NR	CT, TV, BV	NR	No
Holst, 1994[58]	Sweden	Health facility	Urban	NR	NR	CT, NG, BV	20/49 (40.8)	No
Jones, 2009[10]	UK	Health facility	Urban	NR	NR	NR	NR	No
Kacerovsky, 2009[68]	NR	Health facility	NR/ unclear	26 (19-38)	NR	NR	NR	No
Kumar, 2006[69]	India	Health facility	Urban	NR	NR	BV	NR	NR

ONLINE SUPPLEMENTAL FILE

First author, Publication year, study reference*	Study location	Study setting	Urban /rural location	Mean†/ median age years (range)	Ethnicity	Other infections included/(excluded)	Smokers included (%)	Multiple pregnancies
McDonald, 1992[59]	Australia	Health facility	Urban	NR	NR	NR	839/ 2190 (39.8%)	NR
Mitsunari, 2005[60]	Japan	Health facility	Urban	NR	Asian	(CT)	NR	No
Montenegro, 2019[70]	Colombia	Health facility	Urban	NR	NR	NR	NR	NR
Munday, 1984[61]	UK	Health facility	Urban	NR	Mixed	CT	NR	NR
Oliveira, 2020[7]	Brazil	Health facility	Urban	27.3	Mixed	NG, MG	5/109 (4.6)	NR
Payne, 2014[62]	China, Australia	Health facility	NR/unclear	17- 49	Mixed	NR	69/972 (7.1%)	No
Toth, 1992[63]	UK	Health facility	Urban	NR	NR	CT, TV	NR	NR
Yoon, 2001[64]	South Korea	Health facility	Urban	NR	NR	NR	NR	No

BV, bacterial vaginosis; CA, *Candida albicans*; CT, *Chlamydia trachomatis*; HIV, human immunodeficiency virus; NG, *Neisseria gonorrhoeae*; NR, not reported; No, did not include multiple pregnancy; Yes, included multiple pregnancies; TV, *Trichomonas vaginalis*; UK, United Kingdom; USA, United States of America.

* Study reference is the reference number cited in the main manuscript;

† Reported mean age.

ONLINE SUPPLEMENTAL FILE

Table S4.3 Study setting and socio-demographics, cross-sectional studies (n=5), in alphabetical order

First author, Publication year, study reference*	Study location	Study setting	Urban /rural location	Mean†/ median age years (range)	Ethnicity	Other infections included/(excluded)	Smokers included (%)	Multiple pregnancies
Grattard, 1995[71]	France	Health facility	Urban	NR	NR	NR	NR	NR
Kundsin, 1984[72]	USA	Health facility	Urban	NR	Mixed	NR	105/801 (31.4%)	Yes
McCormack, 1975[73]	USA	Health facility	Urban	23.6†	Mixed	NR	NR	Yes
Nasution, 2007[75]	NR	Health facility	NR/ unclear	24 - 38	Asian	CT, NG	NR	NR
Sweeney, 2016[74]	USA	Health facility	Urban	NR	Mixed	NR	NR	Yes

BV, bacterial vaginosis; CT, *Chlamydia trachomatis*; HIV, human immunodeficiency virus; NG, *Neisseria gonorrhoeae*; No, did not include multiple pregnancy; Yes, included multiple pregnancies; NR, not reported; TV, *Trichomonas vaginalis*; UK, United Kingdom; USA, United States of America

* Study reference is the reference number cited in the main manuscript

† Reported mean age

ONLINE SUPPLEMENTAL FILE

Table S5 Studies that reported on bacterial vaginosis and associations with adverse birth outcomes (n=10), in alphabetical order

First author, publication year, study reference*	Study population	Organism/ outcome	OR (95% CI)	Reported associations with genital mycoplasmas
Donders, 2009[36]	Cohort study: 759 women; 55 PTB; 64 BV; 14 *M. hominis*	BV/PTB	2.43 (1.1, 4.7)	Association between lactobacilli and PTB, and between BV and PTB reported as primary analysis. Proportion of women with *M. hominis* but no BV reported (0.5% of 759), but association between *M. hominis* and PTB in absence of BV could not be calculated from data presented. Discussion includes, "In the literature, the presence of *M. hominis* has generally been related to an increased risk of miscarriage, and premature delivery if found in combination with bacterial vaginosis."
Hillier, 1988[57]	Case-control study: 94 women; 38 PTB; 28 BV; 29 *U. urealyticum*; 5 *M. hominis*	BV/PTB	3.31 (1.20, 9.24)	Association between organisms in chorioamnion and PTB reported as primary analysis. BV measured in vaginal smears. Association between genital mycoplasmas and PTB in absence of BV could not be calculated from data presented.
Hillier, 1995[33]	Cohort study: 9105 women; 423 PTB; 1392 BV; 2805 *M. hominis*	BV/ PTB<br>MH+, BV+/ PTB<br>MH+, BV-/PTB	1.60 (1.25, 2.03)<br>1.58 (0.94, 2.77)<br>1.18 (0.91, 1.52)	Association between BV and PTB of low birthweight infants reported as primary analysis. Raw data not available for association between *M. hominis* and PTB but reported in text and can be extracted from bar chart of ORs for PTB, stratified by *M. hominis*, bacteroides and BV. OR for BV and BV with *M. hominis* similar, and stronger than association for *M. hominis* alone.
Holst, 1994[58]	Case-control: 87 women; 22 PTB; 16 BV, 20 *M. hominis;*	BV/PTB<br>MH+, BV+/ PTB<br>MH+, BV-/PTB	5.73 (1.54, 21.45)<br>2.62 (0.19, 42.24)<br>2.6 (0.36, 14.63)	Association between BV and PTB reported Relative risk (< 36 weeks, n=22: RR 2.10, 95% CI 1.2, 3.7 and <34 weeks, n=15: RR 2.05; 95% CI 0.9,4.6) as primary analysis. Associations with *M. hominis* stratified by BV status was calculated from data presented in tables 3 and 5 of the study.

BMJ Publishing Group Limited (BMJ) disclaims all liability and responsibility arising from any reliance placed on this supplemental material which has been supplied by the author(s)

ONLINE SUPPLEMENTAL FILE

First author, publication year, study reference*	Study population	Organism/ outcome	OR (95% CI)	Reported associations with genital mycoplasmas
Kumar, 2006[69]	Case-control study: 120 women; 60 PTB; 31 BV; 6 *M. hominis*	BV/PTB	5.05 (1.97, 12.95)	Association between BV and PTB reported as primary analysis. Association between *M. hominis* and PTB in absence of BV could not be calculated from data presented. Discussion does not mention *M. hominis.*
Menard, 2010[43]	Cohort study: 90 women; 36 PTB; 2 BV; 10 *M. hominis*			Association between quantities of BV-associated bacteria and PTB reported as primary analysis. Association between *M. hominis* and PTB in absence of BV could not be calculated from data presented.
Minkoff, 1984[44]	Cohort study; 188 women with PROM; 74 BV†; *T. vaginalis* 30; 40 PROM (21 *M. hominis;* 16 BV; *T. vaginalis* 11), 148 not PROM (52 *M. hominis*; 42 BV; *T. vaginalis* 19)	BV/PROM<br>TV/ PROM	1.68 (0.75, 3.68)<br>2.57 (0.99, 6.41)	Association between quantities of BV-associated bacteria and PROM, and *T. vaginalis* and PROM were reported as primary analysis, where TV/BV was significant (P-value=0.03). Association between PROM and BV, and PROM and *T. vaginalis* could be calculated from data presented. Association between *M. hominis* and PROM in absence of BV and/ or *T. vaginalis* could not be calculated from data presented. Discussion regarding PROM includes “in stepwise logistic regression, *M. hominis* was no longer statistically significant when effects of *T. vaginalis* and *S. epidermidis* were taken into account.”
Odendaal, 2002[51]	Cohort study as sub-study of a randomised controlled trial: 395 women; 119 PTB; 132 BV; 83 *M. hominis*	MH/BV	10.21 (5.63, 18.65)	Association between *M. hominis* and PTB reported as primary analysis. Association between *M. hominis* and BV reported, but not association between BV and PTB. Discussion includes, “It is also possible that the BV is not directly involved in the causation of premature labour but that it is only a marker of a more important underlying condition such as *M. hominis* infection,”

ONLINE SUPPLEMENTAL FILE

First author, publication year, study reference*	Study population	Organism/ outcome	OR (95% CI)	Reported associations with genital mycoplasmas
Rittenschober-Böhm, 2018[47]	Cohort study: 3643 women; 292 PTB; 279 BV; 1347 *U. parvum*; 214 *U. urealyticum*	BV/PTB<br>UP-,BV-/PTB<br>UP+,BV-/PTB<br>UP-,BV+/PTB<br>UP+,BV+/PTB	Crude 1.7 (1.3, 2.2)<br>Adjusted 1.6 (1.1, 2.4)<br>Adjusted 1.6 (1.2, 2.1)<br>Adjusted 1.6 (1.1, 2.3)<br>Adjusted 2.6 (1.7, 4.0)	Associations between *Ureaplasma* spp. and PTB reported as primary analysis. Associations with *U. parvum*, stratified by BV status and adjusted for maternal age, diagnosis of vaginal candida, smoking and history of previous PTB. Stratified associations with *U. urealyticum* not reported on basis of univariable analysis (OR 1.4, 95% CI 0.8, 2.2). Discussion does not mention potential associations between both BV and *Ureaplasma* spp.
Schwab, 2015[53]	Cohort study: 62 women; 23 PTB; 13 BV; 13 *M. hominis*;	None reported		Descriptive study of infections in pregnancy. Association between *M. hominis* and PTB reported, but not association between BV and PTB.

BV, bacterial vaginosis; CI, confidence interval; MH, *Mycoplasma hominis*; OR, odds ratio; PTB, premature birth; UP, *Ureaplasma parvum*; UU, *Ureaplasma urealyticum.*

* Study reference is the reference number cited in the main manuscript;

† reported by authors as non-specific vaginitis

Jonduo ME, *et al. BMJ Open* 2023; 12:e062990. doi: 10.1136/bmjopen-2022-062990

ONLINE SUPPLEMENTAL FILE

Table S6.1 Risk of bias assessment, cohort studies (n=26)

Questions	Agger, 2014[1]	Berman, 1987[34]	Braun, 1971[35]	Donders, 2009[36]	Gerber, 2003[37]
1) The method of allocation to exposure groups was unrelated to potential confounding factors	NA	NA	NA	NA	NA
2) Attempts made within design or analysis to balance both groups for potential confounders.	Yes	Unclear	Unclear	Unclear	Unclear
3) The groups were comparable at baseline, including all major confounding factors.	No	Yes	Unclear	Yes	Unclear
4) Based on above answers, was selection bias present?	No	Low	Unclear	Unclear	Unclear
5) If so, what is the likely direction of its effect?	Unclear	Unclear	Unclear	Unclear	Unclear
6) The comparison groups received the same care and support apart from the exposure(s) studied.	Yes	Unclear	Unclear	Yes	Unclear
7) Participants receiving care and support were kept 'blind' to intervention allocation.	NA	NA	NA	NA	NA
8) Individuals administering care, support were kept 'blind' to intervention allocation.	NA	NA	NA	NA	NA
9) Based on above answers, was performance bias present?	Unclear	Unclear	Unclear	Unclear	Unclear
10) If so, what is the likely direction of its effect?	Unclear	Unclear	Unclear	Unclear	Unclear
11) All groups followed up for an equal length of time?	Low	Low	Low	Low	Low
12) Number of participants did not complete the intervention in each group?	NA	NA	NA	NA	NA
13) The groups were comparable for intervention completion.	NA	NA	Unclear	NA	NA
14) For how many participants were no outcome data available?‡	107/783, (13.7%)	104/1204 (8.6%)	203/688 (30%)	42/801 (5.2%)	63/317 (19.9%)
15) Were groups comparable for outcome data?	Unclear	Unclear	Unclear	Yes	Unclear
16) Based on above answers, was attrition bias present?	Unclear	Unclear	Unclear	Unclear	Unclear
17) If so, what is the likely direction of its effect?	Unclear	Unclear	Unclear	Unclear	Unclear
18) The study had an appropriate length of follow-up.	Yes	Yes	Yes	Yes	Yes
19) The study used a precise definition of outcome.	Yes	Unclear	No	Yes	Yes
20) A valid, reliable method used to determine the outcome?	Unclear	Unclear	No	Yes	Yes
21) Investigators were kept 'blind' to participants' exposure to the intervention.	NA	NA	NA	NA	NA
22) Investigators were kept 'blind' to other important confounding factors.	Unclear	Unclear	Unclear	Unclear	Unclear
23) Based on above answers, was detection bias present?	Unclear	Unclear	Unclear	No	Unclear
24) If so, what is the likely direction of its effect?	Unclear	Unclear	Unclear	NA	Unclear
**25) Overall assessment of internal validity[a]**	**+**	**-**	**+**	**+**	**+**
**26) Overall assessment of external validity[a]**	**+**	**+**	**-**	**+**	**-**

ONLINE SUPPLEMENTAL FILE

Questions	Govender, 2009[38]	Harrison, 1983[39]	Hillier, 1995[33]	Kataoka, 2006[40]	Koucky, 2016[41]
1) The method of allocation to exposure groups was unrelated to potential confounding factors	NA	NA	NA	NA	NA
2) Attempts made within design or analysis to balance both groups for potential confounders.	Unclear	Unclear	Unclear	Yes	Unclear
3) The groups were comparable at baseline, including all major confounding factors.	Unclear	Unclear	Unclear	No	Unclear
4) Based on above answers, was selection bias present?	Unclear	Unclear	Unclear	Unclear	Unclear
5) If so, what is the likely direction of its effect?	Unclear	Unclear	Unclear	Unclear	Unclear
6) The comparison groups received the same care and support apart from the exposure(s) studied.	Unclear	Yes	Yes	Yes	Yes
7) Participants receiving care and support were kept 'blind' to intervention allocation.	NA	NA	NA	NA	NA
8) Individuals administering care and support were kept 'blind' to intervention allocation.	NA	NA	NA	NA	NA
9) Based on above answers, was performance bias present?	Unclear	Unclear	Unclear	Unclear	Unclear
10) If so, what is the likely direction of its effect?	Unclear	Unclear	Unclear	Unclear	Unclear
11) All groups followed up for an equal length of time?	Low	Low	Low	Low	Low
12) Number of participants did not complete the intervention in each group?	NA	NA	Na	NA	NA
13) The groups were comparable for intervention completion.	NA	NA	NA	NA	NA
14) For how many participants were no outcome data available?‡	0/199 (0%)	SA, 13/361 (3.6%); PND, 0/467 (0%)	1292/10397 (12.4%)	163/1040 (15.7%)	0/36 (0%)
15) Were groups comparable for outcome data?	Unclear	Unclear	No	Unclear	Yes
16) Based on above answers, was attrition bias present?	Unclear	Unclear	Unclear	Unclear	Unclear
17) If so, what is the likely direction of its effect?	Unclear	Unclear	Unclear	Unclear	Unclear
18) The study had an appropriate length of follow-up.	Yes	Yes	Yes	Yes	Yes
19) The study used a precise definition of outcome.	Yes	No	Yes	Yes	Yes
20) A valid, reliable method used to determine the outcome?	Unclear	Unclear	No	Yes	Yes
21) Investigators were kept 'blind' to participants' exposure to the intervention.	NA	NA	NA	NA	NA
22) Investigators were kept 'blind' to other important confounding factors.	Unclear	Unclear	Unclear	Unclear	Unclear
23) Based on above answers, was detection bias present?	Unclear	Unclear	Unclear	No	Unclear
24) If so, what is the likely direction of its effect?	Unclear	Unclear	Unclear	NA	Unclear
**25) Overall assessment of internal validity[a]**	**-**	**-**	**-**	**+**	**+**
**26) Overall assessment of external validity[a]**	**-**	**-**	**-**	**+**	**+**

ONLINE SUPPLEMENTAL FILE

Questions	Kwak, 2014[50]	Lee, 2016[6]	Luton, 1994[8]	McDonald, 1994[42]	Menard, 2010[43]
1) The method of allocation to exposure groups was unrelated to potential confounding factors	NA	NA	NA	NA	NA
2) Attempts made within design or analysis to balance both groups for potential confounders.	Unclear	Unclear	Yes	Yes	Unclear
3) The groups were comparable at baseline, including all major confounding factors.	Unclear	Unclear	Yes	Unclear	Unclear
4) Based on above answers, was selection bias present?	Unclear	Unclear	Low	Unclear	Unclear
5) If so, what is the likely direction of its effect?	Unclear	Unclear	Unclear	Unclear	Unclear
6) The comparison groups received the same care and support apart from the exposure(s) studied.	Unclear	Unclear	Yes	Unclear	Yes
7) Participants receiving care and support were kept 'blind' to intervention allocation.	NA	NA	NA	NA	NA
8) Individuals administering care and support were kept 'blind' to intervention allocation.	NA	NA	NA	NA	NA
9) Based on above answers, was performance bias present?	Unclear	Unclear	Unclear	Unclear	Unclear
10) If so, what is the likely direction of its effect?	Unclear	Unclear	Unclear	Unclear	Unclear
11) All groups followed up for an equal length of time?	Low	Low	Low	Low	Low
12) Number of participants did not complete the intervention in each group?	NA	NA	NA	NA	NA
13) The groups were comparable for intervention completion.	NA	NA	NA	NA	NA
14) For how many participants were no outcome data available?‡	0/179 (0%)	0/1035 (0%)	37/218 (17%)	Control, 182/649, (28%); Cases, 42/135 (31%)	0/90 (0%)
15) Were groups comparable for outcome data?	Yes	Yes	No	Unclear	Yes
16) Based on above answers, was attrition bias present?	Low	Low	Unclear	Unclear	Unclear
17) If so, what is the likely direction of its effect?	Unclear	Unclear	Unclear	Unclear	Unclear
18) The study had an appropriate length of follow-up.	Yes	Yes	Yes	Yes	Yes
19) The study used a precise definition of outcome.	Yes	No	Yes	Yes	Yes
20) A valid, reliable method used to determine the outcome?	Yes	No	Yes	No	Yes
21) Investigators were kept 'blind' to participants' exposure to the intervention.	NA	NA	NA	Unclear	NA
22) Investigators were kept 'blind' to other important confounding factors.	Unclear	Unclear	Unclear	Unclear	NA
23) Based on above answers, was detection bias present?	No	Yes	Unclear	Unclear	No
24) If so, what is the likely direction of its effect?	NA	Unclear	Unclear	Unclear	NA
**25) Overall assessment of internal validity[a]**	**+**	**-**	**-**	**-**	**-**
**26) Overall assessment of external validity[a]**	**+**	**-**	**-**	**-**	**-**

ONLINE SUPPLEMENTAL FILE

Questions	Minkoff, 1984[44]	Nguyen, 2004[45]	Odendaal, 2002[51]	Payne, 2016[46]	Payne, 2021[15]
1) The method of allocation to exposure groups was unrelated to potential confounding factors	NA	NA	NA	NA	NA
2) Attempts made within design or analysis to balance both groups for potential confounders.	Unclear	Unclear	Unclear	Unclear	No
3) The groups were comparable at baseline, including all major confounding factors.	Unclear	Unclear	Unclear	Unclear	Unclear
4) Based on above answers, was selection bias present?	Unclear	Unclear	Unclear	Unclear	Low
5) If so, what is the likely direction of its effect?	Unclear	Unclear	Unclear	Unclear	Unclear
6) The comparison groups received the same care and support apart from the exposure(s) studied.	Unclear	Unclear	Yes	Unclear	Yes
7) Participants receiving care and support were kept 'blind' to intervention allocation.	NA	NA	NA	NA	NA
8) Individuals administering care and support were kept 'blind' to intervention allocation.	NA	NA	NA	NA	NA
9) Based on above answers, was performance bias present?	Unclear	Unclear	Unclear	Unclear	Unclear
10) If so, what is the likely direction of its effect?	Unclear	Unclear	Unclear	Unclear	Unclear
11) All groups followed up for an equal length of time?	Low	Low	Low	Low	Low
12) Number of participants did not complete the intervention in each group?	NA	NA	NA	NA	NA
13) The groups were comparable for intervention completion.	NA	NA	NA	NA	NA
14) For how many participants were no outcome data available?‡	PROM 45/233 (19.3%); PTB 15/233 (6.4%)	61/456 (13.4%)	31/426 (7.3%)	15/206 (7.3%)	6.4% (64/1000)
15) Were groups comparable for outcome data?	Unclear	Yes	Yes	Yes	Unclear
16) Based on above answers, was attrition bias present?	Unclear	Low	Unclear	Unclear	Low
17) If so, what is the likely direction of its effect?	Unclear	Unclear	Unclear	Unclear	Unclear
18) The study had an appropriate length of follow-up.	Yes	Yes	Yes	Yes	Yes
19) The study used a precise definition of outcome.	Yes	Yes	Yes	Yes	Yes
20) A valid, reliable method used to determine the outcome?	Yes	Unclear	Yes	Unclear	Unclear
21) Investigators were kept 'blind' to participants' exposure to the intervention.	NA	NA	NA	NA	NA
22) Investigators were kept 'blind' to other important confounding factors.	Unclear	Unclear	Unclear	Unclear	Unclear
23) Based on above answers, was detection bias present?	No	Unclear	Unclear	Unclear	No
24) If so, what is the likely direction of its effect?	NA	Unclear	Unclear	Unclear	NA
**25) Overall assessment of internal validity[a]**	**-**	**+**	**+**	**+**	**+**
**26) Overall assessment of external validity[a]**	**-**	**+**	**-**	**+**	**+**

ONLINE SUPPLEMENTAL FILE

Questions	Peretz, 2020[12]	Perni, 2004[52]	Rittenschober-Böhm, 2018[47]	Schwab, 2015[53]	Sperling, 1988[49]	Usui, 2002[48]
1) The method of allocation to exposure groups was unrelated to potential confounding factors	NA	NA	NA	NA	NA	NA
2) Attempts made within design or analysis to balance both groups for potential confounders.	Yes	Unclear	Yes	No	Unclear	Unclear
3) The groups were comparable at baseline, including all major confounding factors.	Unclear	Unclear	Unclear	Unclear	Unclear	Unclear
4) Based on above answers, was selection bias present?	Unclear	Unclear	Low	High	Unclear	Unclear
5) If so, what is the likely direction of its effect?	Unclear	Unclear	Unclear	Unclear	Unclear	Unclear
6) The comparison groups received the same care and support apart from the exposure(s) studied.	Yes	Unclear	Unclear	Unclear	Yes	Yes
7) Participants receiving care and support were kept 'blind' to intervention allocation.	NA	NA	NA	NA	NA	NA
8) Individuals administering care and support were kept 'blind' to intervention allocation.	NA	NA	NA	NA	NA	NA
9) Based on above answers, was performance bias present?	Unclear	Unclear	Unclear	Unclear	Unclear	Low
10) If so, what is the likely direction of its effect?	Unclear	Unclear	Unclear	Unclear	Unclear	NA
11) All groups followed up for an equal length of time?	Low	Low	Low	Low	Low	Low
12) Number of participants did not complete the intervention in each group?	NA	NA	NA	NA	NA	NA
13) The groups were comparable for intervention completion.	NA	NA	NA	NA	NA	NA
14) For how many participants were no outcome data available?‡	PTB, 195/214 (91%); LBW, 192/214 (90%)	14/193 (7.3%)	687/4330 (15.9%)	97/159 (61.0%)	5/409 (1.2%)	0/1958 (0%)
15) Were groups comparable for outcome data?	No	Unclear	Unclear	Unclear	Yes	Yes
16) Based on above answers, was attrition bias present?	High	Unclear	Unclear	High	Low	Low
17) If so, what is the likely direction of its effect?	Unclear	Unclear	Unclear	Unclear	NA	NA
18) The study had an appropriate length of follow-up.	Yes	Yes	Yes	Yes	Yes	Yes
19) The study used a precise definition of outcome.	Yes	Yes	Yes	Yes	Yes	Yes
20) A valid, reliable method used to determine the outcome?	Yes	Unclear	Yes	No	No	Yes
21) Investigators were kept 'blind' to participants' exposure to the intervention.	NA	NA	NA	NA	NA	NA
22) Investigators were kept 'blind' to other important confounding factors.	NA	Unclear	Unclear	Unclear	Unclear	Unclear
23) Based on above answers, was detection bias present?	No	Unclear	No	Yes	Unclear	Unclear

ONLINE SUPPLEMENTAL FILE

Questions	Peretz, 2020[12]	Perni, 2004[52]	Rittenschober-Böhm, 2018[47]	Schwab, 2015[53]	Sperling, 1988[49]	Usui, 2002[48]
24) If so, what is the likely direction of its effect?	NA	Unclear	NA	Unclear	Unclear	Unclear
**25) Overall assessment of internal validity[a]**	**-**	**+**	**+**	**-**	**-**	**+**
**26) Overall assessment of external validity[a]**	**-**	**+**	**+**	**-**	**-**	**-**

High, high risk of bias; Low, low risk of bias; LBW, low birth weight; NA, not applicable; NK, not known; PTB, preterm birth; Unclear, unclear of risk of bias. ‡Both groups combined unless stated.
Validity [a]: ++ all or most of checklist criteria fulfilled; + some of checklist criteria fulfilled; – few or no checklist criteria fulfilled.

Study reference number is the reference number cited in the main manuscript

ONLINE SUPPLEMENTAL FILE

Table S6.2 Risk of bias assessment, case-control studies (n=22)

Questions	Ahmadi, 2014[9]	Cassell, 1993[32]	Chua, 1999[67]	Daskalakis, 2009[65]	Embree, 1980[54]	Farhadifar, 2016[11]
1) Appropriate and clearly focused question.	WC	WC	WC	WC	AA	WC
2) The cases and controls are taken from comparable populations.	AA	WC	WC	AA	PA	AA
3) The same exclusion criteria are used for both cases and controls.	WC	NA	PA	WC	NAd	AA
4) What was the participation rate (%) for each group (cases)?	Unclear	29/33 (87.9%)	Unclear	Unclear	100% (n=446)	Unclear
5) What was the participation rate (%) for each group (controls)?	Unclear	100% (n=28)	Unclear	Unclear	100% (n=108)	Unclear
6) Both groups compared to establish their similarities or differences.	NAd	NAd	NAd	NAd	NAd	NAd
7) Cases are clearly defined and differentiated from controls.	WC	AA	AA	WC	AA	WC
8) It is clearly established that controls are not cases.	WC	AA	AA	WC	AA	WC
9) Measures taken to prevent knowledge of primary exposure from influencing case ascertainment.	NA	NA	NA	NA	NA	NA
10) Exposure status is measured in a standard, valid and reliable way.	WC	AA	AA	WC	AA	AA
11) Main potential confounders are accounted for in design/analysis	AA	PA	NR	AA	PA	AA
12) Confidence intervals provided?	No	No	No	No	No	Yes
**13) Study results internally valid[a]**	**+**	**+**	**+**	**+**	**-**	**+**
**14) Study results externally valid[a]**	**+**	**-**	**+**	**+**	**-**	**+**

Questions	Freitas, 2018[55]	Gonzàlez Bosquet, 2006[66]	Harada, 2008[56]	Hillier, 1988[57]	Holst, 1994[58]	Jones, 2009[10]
1) Appropriate and clearly focused question.	AA	WC	WC	WC	WC	WC
2) The cases and controls are taken from comparable populations.	PA	AA	AA	AA	PA	PA
3) The same exclusion criteria are used for both cases and controls.	NAd	AA	AA	PA	AA	NAd
4) What was the participation rate for each group (cases)? %	100% (n=46)	Unclear	Unclear	99/107 (92.5%)	49/120 (40.8%)	Unclear
5) What was the participation rate for each group (controls)? %	100% (n=170)	Unclear	Unclear	68/140 (48.6%)	38/38 (100%)	Unclear
6) Both groups compared to establish their similarities or differences.	AA	NAd	NAd	NAd	NAd	NAd
7) Cases are clearly defined and differentiated from controls.	AA	WC	AA	WC	WC	AA
8) It is clearly established that controls are not cases.	AA	WC	AA	WC	WC	AA

Jonduo ME, *et al. BMJ Open* 2023; 12:e062990. doi: 10.1136/bmjopen-2022-062990

ONLINE SUPPLEMENTAL FILE

Questions	Freitas, 2018[55]	Gonzàlez Bosquet, 2006[66]	Harada, 2008[56]	Hillier, 1988[57]	Holst, 1994[58]	Jones, 2009[10]
9) Measures taken to prevent knowledge of primary exposure from influencing case ascertainment.	NA	NA	NA	NA	NA	NA
10) Exposure status is measured in a standard, valid, and reliable way.	WC	AA	WC	AA	AA	WC
11) Main potential confounders are accounted for in design/analysis	PA	PA	PA	WC	AA	NAd
12) Confidence intervals provided?	No	No	Yes	Yes	No	No
**13) Study results internally valid[a]**	+	+	+	+	++	-
**14) Study results externally valid[a]**	-	+	+	+	+	-

Questions	Kacerovsky, 2009[68]	Kumar, 2006[69]	McDonald, 1992[59]	Mitsunari, 2005[60]	Montenegro, 2019[70]
1) Appropriate and clearly focused question.	WC	AA	AA	WC	WC
2) The cases and controls are taken from comparable populations.	PA	PA	AA	WC	AA
3) The same exclusion criteria are used for both cases and controls.	PA	NAd	PA	WC	AA
4) What was the participation rate for each group (cases)?	Unclear	100% (n=60)	Unclear	23/40 (57.5%)	84 (100%)
5) What was the participation rate for each group (controls)?	Unclear	100% (n=60)	Unclear	59/97 (60.8%)	127 (1000%)
6) Both groups compared to establish their similarities or differences.	NAd	NAd	AA	NA	NA
7) Cases are clearly defined and differentiated from controls.	WC	NAd	AA	AA	AA
8) It is clearly established that controls are not cases.	PA	AA	AA	AA	AA
9) Measures taken to prevent knowledge of primary exposure from influencing case ascertainment.	NA	NA	NA	NA	NA
10) Exposure status is measured in a standard, valid, and reliable way.	AA	PA	PA	WC	WC
11) Main potential confounders are accounted for in design/analysis	PA	NAd	AA	PA	PA
12) Confidence intervals provided?	No	No	Yes	No	No
**13) Study results internally valid[a]**	-	-	+	+	+
**14) Study results externally valid[a]**	-	-	+	+	+

Jonduo ME, *et al. BMJ Open* 2023; 12:e062990. doi: 10.1136/bmjopen-2022-062990

ONLINE SUPPLEMENTAL FILE

Questions	Munday, 1984[61]	Oliveira, 2020[7]	Payne, 2014[62]	Toth, 1992[63]	Yoon, 2001[64]
1) Appropriate and clearly focused question.	WC	AA	WC	WC	WC
2) The cases and controls are taken from comparable populations.	AA	PA	WC	AA	AA
3) The same exclusion criteria are used for both cases and controls.	NAd	PA	AA	PA	PA
4) What was the participation rate for each group (cases)?	Unclear	100% (n=unclear)	100% (n=unclear)	Unclear	Unclear
5) What was the participation rate for each group (controls)?	Unclear	100% (n=unclear)	100% (n=unclear)	Unclear	Unclear
6) Both groups compared to establish their similarities or differences.	NA	AA	NAd	NA	NA
7) Cases are clearly defined and differentiated from controls.	PA	WC	AA	PA	WC
8) It is clearly established that controls are not cases.	PA	WC	PA	PA	WC
9) Measures taken to prevent knowledge of primary exposure from influencing case ascertainment.	NA	NA	NAd	NA	NA
10) Exposure status is measured in a standard, valid, and reliable way.	AA	AA	AA	PA	WC
11) Main potential confounders are accounted for in design/analysis	PA	AA	NAd	PA	AA
12) Confidence intervals provided?	No	Yes	Yes	No	No
**13) Study results internally valid[a]**	**+**	**+**	**+**	**-**	**+**
**14) Study results externally valid[a]**	**-**	**+**	**+**	**-**	**+**

AA, adequately addressed; NAd, not addressed; NA, not applicable; PA, poorly addressed; Unclear, does not have data on how many were excluded or declined to participate but only present numbers; WC, well covered.

Validity [a]: ++, all or most of checklist criteria fulfilled; + some of checklist criteria fulfilled; – few or no checklist criteria fulfilled.

Study reference number is the reference number cited in the main manuscript.

Jonduo ME, *et al. BMJ Open* 2023; 12:e062990. doi: 10.1136/bmjopen-2022-062990

ONLINE SUPPLEMENTAL FILE

Table S6.3 Risk of bias assessment, cross-sectional studies (n=5)

Questions	Grattard, 1995[71]	Kundsin, 1984[72]	McCormack, 1975[73]	Nasution, 2007[75]	Sweeney, 2016[74]
1) Is the source population, source area well described?	+	NR	-	NR	+
2) Is the eligible population or area representative of the source population?	+	NR	-	NR	-
3) Do the selected participants or areas represent the eligible population or area?	NR	-	-	NR	-
4) Selection of exposure (and comparison) group. How was selection bias minimised?	NR	NR	NR	NR	NR
5) Was the selection of explanatory variables based on a sound theoretical basis?	-	+	-	+	+
6) Was the contamination acceptably low?	NA	NA	NA	NR	NA
7) How well were likely confounding factors identified and controlled?	NR	NR	NR	NR	-
8) Outcome measures and procedures reliable?	-	+	-	+	-
9) Were the outcome measurements complete?	+	+	+	++	+
10) Were all the important outcomes assessed?	+	+	+	+	++
11) Was there a similar follow-up time in exposure and comparison groups?	-	++	+	++	+
12) Was follow-up time meaningful?	+	++	+	++	+
13) Was the study sufficiently powered to detect an exposure effect (if one exists)	NA	NA	NA	NR	NA
14) Were multiple explanatory variables considered in analyses?	NR	NR	NR	NR	NR
15) Were the analytical methods appropriate?	-	-	+	-	+
16) Was the precision of association given or calculable?	+	+	+	+	+
**17) Overall assessment of internal validity[a]**	**-**	**-**	**+**	**-**	**+**
**18) Overall assessment of external validity[a]**	**+**	**+**	**-**	**-**	**-**

++, yes; +, mostly; -, no; NR, not reported; NA, not applicable. Validity [a]: ++, all or most of checklist criteria fulfilled; + some of checklist criteria fulfilled; – few or no checklist criteria fulfilled.

Study reference number is the reference number cited in the main manuscript.

ONLINE SUPPLEMENTAL FILE

Table S7 Summary of assessment of regression analysis for small study effects, for outcomes reported in 10 or more studies

Organism	Outcome	Number of studies	Egger test coefficient (95% CI)	P value
M. hominis	Preterm birth	30	0.56 (-0.08, 1.2)	0.09
	PROM	11	0.05 (-1.07, 1.17)	0.92
	Spontaneous abortion	10	-0.28 (-3.20, 2.64)	0.83
U. urealyticum	Preterm birth	16	0.24 (-0.57, 1.06)	0.42
U. parvum	Preterm birth	13	0.25 (-0.24, 0.75)	0.25

CI, confidence interval; PROM: premature rupture of membrane.

Jonduo ME, *et al. BMJ Open* 2023; 12:e062990. doi: 10.1136/bmjopen-2022-062990
