# Supplementary material for: Adverse pregnancy and birth outcomes associated with Mycoplasma hominis, Ureaplasma urealyticum and Ureaplasma parvum: a systematic review and meta-analysis
Source: BMJ Open. 2022 Aug 26;12(8):e062990. doi: 10.1136/bmjopen-2022-062990 (PMC9422885; doi:10.1136/bmjopen-2022-062990)
Supplement: Author's manuscript [file bmjopen-2022-062990.draft_revisions.pdf]

BMJ Open

BMJ Open is committed to open peer review. As part of this commitment we make the peer review history of every article we publish publicly available.

When an article is published we post the peer reviewers' comments and the authors' responses online. We also post the versions of the paper that were used during peer review. These are the versions that the peer review comments apply to.

The versions of the paper that follow are the versions that were submitted during the peer review process. They are not the versions of record or the final published versions. They should not be cited or distributed as the published version of this manuscript.

BMJ Open is an open access journal and the full, final, typeset and author-corrected version of record of the manuscript is available on our site with no access controls, subscription charges or pay-per-view fees (http://bmjopen.bmj.com).

If you have any questions on BMJ Open's open peer review process please email info.bmjopen@bmj.com

BMJ Open

Adverse pregnancy and birth outcomes associated with Mycoplasma hominis, Ureaplasma urealyticum and Ureaplasma parvum: A systematic review and meta-analysis

Journal:	*BMJ Open*
Manuscript ID	bmjopen-2022-062990
Article Type:	Original research
Date Submitted by the Author:	21-Mar-2022
Complete List of Authors:	Jonduo, Marinjho; University of New South Wales Faculty of Medicine, Kirby Institute<br>Vallely, Lisa; University of New South Wales Faculty of Medicine, Kirby Institute<br>Wand, Handan; University of New South Wales Faculty of Medicine, Kirby Institute<br>Sweeney, Emma; The University of Queensland Centre for Clinical Research<br>Egli-Gany, Dianne; Universitat Bern Institut fur Sozial- und Praventivmedizin,<br>Kaldor, John; University of New South Wales Faculty of Medicine, Kirby Institute<br>Vallely, Andrew; University of New South Wales Faculty of Medicine, Kirby Institute,; Papua New Guinea Institute of Medical Research, Sexual and Reproductive Health Unit<br>Low, Nicola; University of Bern, Institute of Social and Preventive Medicine
Keywords:	GYNAECOLOGY, MICROBIOLOGY, OBSTETRICS, EPIDEMIOLOGY, Epidemiology < INFECTIOUS DISEASES

SCHOLARONE™
Manuscripts

I, the Submitting Author has the right to grant and does grant on behalf of all authors of the Work (as defined in the below author licence), an exclusive licence and/or a non-exclusive licence for contributions from authors who are: i) UK Crown employees; ii) where BMJ has agreed a CC-BY licence shall apply, and/or iii) in accordance with the terms applicable for US Federal Government officers or employees acting as part of their official duties; on a worldwide, perpetual, irrevocable, royalty-free basis to BMJ Publishing Group Ltd ("BMJ") its licensees and where the relevant Journal is co-owned by BMJ to the co-owners of the Journal, to publish the Work in this journal and any other BMJ products and to exploit all rights, as set out in our licence.

The Submitting Author accepts and understands that any supply made under these terms is made by BMJ to the Submitting Author unless you are acting as an employee on behalf of your employer or a postgraduate student of an affiliated institution which is paying any applicable article publishing charge ("APC") for Open Access articles. Where the Submitting Author wishes to make the Work available on an Open Access basis (and intends to pay the relevant APC), the terms of reuse of such Open Access shall be governed by a Creative Commons licence – details of these licences and which Creative Commons licence will apply to this Work are set out in our licence referred to above.

Other than as permitted in any relevant BMJ Author's Self Archiving Policies, I confirm this Work has not been accepted for publication elsewhere, is not being considered for publication elsewhere and does not duplicate material already published. I confirm all authors consent to publication of this Work and authorise the granting of this licence.

For peer review only

Adverse pregnancy and birth outcomes associated with *Mycoplasma hominis, Ureaplasma urealyticum* and *Ureaplasma parvum*: A systematic review and meta-analysis

Corresponding author: Prof. N Low, MD MSc FFPH, Institute of Social and Preventive Medicine, Mittelstrasse 43, University of Bern, CH-3012 Bern, Switzerland. Tel: +41 30 684 30 92; Email: nicola.low@ispm.unibe.ch. ORCiD 0000-0003-4817-8986

Marinjho Emely Jonduo,[1] Lisa Michelle Vallely,[1,2] Handan Wand,[3] Emma Louise Sweeney,[4] Dianne Egli-Gany,[5] John Martin Kaldor,[1] Andrew John Vallely[1,2] and Nicola Low,[5]

[1] Public Health Interventions Research Program, The Kirby Institute, University of New South Wales, Sydney, NSW 2052, Australia.

[2] Sexual and Reproductive Health Unit, Papua New Guinea Institute of Medical Research, Goroka, Eastern Highlands Province 441, Papua New Guinea.

[3] Biostatistics and Databases program, The Kirby Institute, University of New South Wales, Sydney, New South Wales, Australia.

[4] The University of Queensland Center for Clinical Research, Brisbane, Queensland 4029, Australia.

[5] Institute of Social and Preventive Medicine, University of Bern, CH-3012 Bern, Switzerland.

Word count: 4000 (max 4000)

Conflict of interest: The authors report no conflict of interest.

Study funding: Australian National Health & Medical Research Council (NHMRC); DFID/MRC/Wellcome Trust Joint Global Health Trials; Swiss National Science Foundation.

For peer review only

ABSTRACT

Objectives

Mycoplasma hominis, Ureaplasma urealyticum and *Ureaplasma parvum* (genital mycoplasmas) commonly colonise the urogenital tract in pregnant women. This systematic review aims to investigate the role of genital mycoplasmas in adverse pregnancy and birth outcomes, alone or in combination with bacterial vaginosis.

Data sources and eligibility: We searched Embase, Medline and CINAHL databases from January 1971 to February 2021. Eligible studies tested for any of the three genital mycoplasmas during pregnancy and reported on preterm birth (PTB), low birth weight (LBW), premature rupture of membranes (PROM), spontaneous abortion (SA) and/or perinatal death PND, and were cohort, cross-sectional or case-control studies, or randomised controlled trials.

Study appraisal and synthesis: Two reviewers independently screened titles and abstract, read potentially eligible full texts and extracted data. Two reviewers independently assessed risks of bias using published checklists. Random effects meta-analysis was used to estimate summary odds ratios (OR, with 95% confidence intervals, and prediction intervals). Multivariable and stratified analyses were synthesised descriptively.

Results

Of 57/1194 included studies, 39 were from high-income countries. In meta-analysis of unadjusted ORs, *M. hominis* was associated with PTB, PROM, LBW and PNM, but not SA. *U. urealyticum* was associated with PTB, PROM, SA and PNM. *U. parvum* was associated with PTB, PROM and SA. Nine of 57 studies reported any multivariable analysis. In two studies, analyses stratified by BV status showed that *M. hominis* and *U. parvum* were more strongly associated with PTB in the presence than in the absence of BV. The most frequent source of bias was a failure to control for confounding.

Conclusions

For peer review only

The currently available literature does not allow conclusions about the role of mycoplasmas in adverse pregnancy and birth outcomes, alone or with co-existing bacterial vaginosis. Future studies that consider genital mycoplasmas in the context of the vaginal microbiome are needed.

PROSPERO published date: 01 Nov 2018; **registration number:** CRD42016050962

Strengths and limitations

- We followed a published protocol with predefined outcomes and statistical analysis plan
- Two reviewers independently selected the studies, extracted data and performed risk of bias assessment
- Evidence for heterogeneity was examined and described both visually and statistically
- We triangulated findings across study designs
- Restriction to studies in English and German might have missed eligible articles.

For peer review only

INTRODUCTION

Mycoplasma hominis, Ureaplasma parvum and *Ureaplasma urealyticum*, referred to together as genital mycoplasmas, commonly colonise the urogenital tract in women, and are often found together.[1, 2] These species do not appear to cause symptoms or harmful effects in nonpregnant women.[2, 3] Plummer et al. found that *M. hominis* was associated with abnormal vaginal discharge only in nonpregnant women who also had BV.[2] Colonisation with a genital mycoplasma has, however, been reported in many studies to be associated with several adverse pregnancy outcomes[4, 5] including preterm birth (PTB); low birth weight (LBW); premature rupture of membranes (PROM) and preterm premature rupture of the membranes (PPROM), spontaneous abortion (SA), and perinatal death (PND).[1, 6-12] Several research groups have suggested that *M. hominis*, whilst considered a part of the normal vaginal microbiota, might only be pathogenic in the presence of bacterial vaginosis (BV) as part of a disturbed vaginal microbiota.[4, 5, 13] There are, however, inconsistencies across studies, uncertainty about the interplay between specific organisms and the vaginal microbiota in general,[14-16] and differences in recommendations for testing and treatment.[13, 17]

Technological advances in the molecular detection of multiple vaginal and endocervical organisms in the same assay[18, 19] should make it easier to study the role of genital mycoplasmas in adverse pregnancy outcomes. Methods to distinguish between *U. urealyticum* and *U. parvum* were not widely available before 2000,[20, 21] and unspeciated *Ureaplasma* spp. detected by culture were reported together as *U. urealyticum*.[18] Narrative reviews have not fully elucidated whether the apparent pathogenicity of genital mycoplasmas in pregnancy is associated with a particular organism, concurrent infection with multiple genital mycoplasmas and other lower genital tract organisms, or confounding by other demographic, clinical and behavioural factors.[4, 5, 13] A systematic and quantitative assessment of these questions is therefore timely.

For peer review only

OBJECTIVES

The primary objective of this study was to investigate the associations between *M. hominis*, *U. urealyticum* and/or *U. parvum* and the risk of PTB, alone and in combination with BV. Secondary objectives were to investigate associations between each genital mycoplasma and LBW, PROM, SA and PND.

METHODS

This systematic review followed a registered protocol (PROSPERO CRD42016050962),[22] which covers multiple organisms, for which findings are reported elsewhere, including *Neisseria gonorrhoeae*[23] and *M. genitalium.*[24] We report our findings using the Preferred Reporting Items for Systematic Reviews and Meta-Analyses (PRISMA 2020) (A.1)[25] and methodological guidance about systematic reviews of observational studies (MOOSE) (A.2).[26] Patients or the public were not involved in the design, or conduct, or reporting, or dissemination plans of our research.

For peer review only

Eligibility criteria, information sources and search strategy

Studies were eligible if they reported on pregnant women with and without *M. hominis*, *U. urealyticum* and/or *U. parvum* and included one or more of the outcomes: PTB, LBW, PROM (preterm or term), SA and PND. Standard definitions were used for all outcomes (PTB, delivery at <37 weeks gestation; LBW, birthweight <2.5kg; PROM, rupture of membranes prior to onset of labour; PPROM, premature rupture at <37 weeks gestation; SA, delivery at <20 weeks gestation; stillbirth (death after >20 weeks in utero); perinatal or neonatal death (PND, stillbirths and death <28 days after birth), but we used author's definitions if necessary.[22] We excluded articles published before 2000 if they reported unspeciated *U. urealyticum* alone. If they reported on *M. hominis* and *U. urealyticum* we included the study but did not extract results about

U. urealyticum. We included cohort, cross-sectional and case-control studies, and randomised controlled trials.

We searched Medline, Embase, Cumulative Index to Nursing and Allied Health Literature (CINAHL) for literature published from January 1971 to February 2021. We searched reference lists of included studies for additional potentially eligible studies but did not search grey literature sources. The searches did not include language restrictions, but we only read the full-text of articles in English and German (languages spoken by the review team). The full search strategy is in the online supporting information (A.3). We used Endnote (V7, Thomson Reuters) to import, de-duplicate and manage retrieved records.

Study selection and data extraction

Two reviewers (MJ, LV) independently screened titles and abstracts, and read the full text of potentially eligible papers. Disparities were resolved by discussion or by a third reviewer (DEG). Where multiple reports presented data from the same study population, we identified a primary record with the most detailed information but included data from other publications. Two reviewers (MJ, LV) extracted data independently into an online database (Research Electronic Data Capture, REDCap, Vanderbilt University, Tennessee). Disparities were resolved by discussion or by a third reviewer (DEG, NL or ES).

Data extraction

Each reviewer extracted data about the study design, study setting and sociodemographic characteristics, specimen type and timing, laboratory tests, organisms tested for, outcomes reported, raw numbers of participants with and without each outcome and organism, where available, or author reported effect size and 95% confidence intervals (CI). They extracted the adjusted odds ratio (aOR, 95% CI) and recorded variables included in multivariable models, where possible. If results were described for more than one anatomical site, we used the

For peer review only

following order of preference: vaginal or cervical swabs, amniotic fluid, placenta, urine, blood. Where more than one diagnostic method, we used data from nucleic acid amplification test (NAAT), then bacterial culture, followed by ELISA. The data underlying this article are available in the article and in its online supplementary material.

Risk of bias assessments

Two reviewers (MJ, LV) appraised each article independently, using checklists published by the UK National Institute for Health and Care Excellence (NICE).[27, 28] A qualitative judgement about internal and external validity was summarised as: all or most checklist criteria fulfilled (++), some criteria fulfilled (+), or few or no criteria fulfilled (-). We used funnel plots and the Egger test[29] to investigate evidence for publication or small study biases across studies for outcomes reported by more than nine studies.

Data synthesis

We used Stata 14.0 (StataCorp, College Station, TX) for all analyses. We used the OR, with 95% CI as the measure of association for all study designs, since the OR and risk ratio are similar for rare outcomes, as is the case for most of the outcomes of interest. This allowed us to analyse findings from different study designs together, where appropriate.[30] We constructed 2x2 tables to calculate of the OR or used the authors' calculation when raw data were unavailable. We added 0.5 to each cell in the table if there were zero observations in any cell. For each exposure-outcome pair, we examined forest plots of univariable associations visually, displaying the OR (with 95% CI) and the I^2 statistic, to examine between study heterogeneity. We used a random effects model to estimate a summary OR (95% CI), which is the average effect across all included studies,[31] allowing for the differences in study designs, populations and settings. We stratified studies by study design in forest plots and, where the stratified estimates were compatible, we estimated the overall estimated OR with its prediction interval, to show the range of effect sizes across all settings of included studies.[31] We then examined evidence for from

For peer review only

studies that also reported on BV. We described findings from analyses that were stratified by BV status, or in studies with a multivariable analysis, we reported the aOR, controlling for BV and other measured confounding variables.[26]

RESULTS

Study selection

Our searches identified 1194 records and we screened 641, after exclusion of duplicates (Figure S1). Of 215 full-text articles, we included 57 studies. Articles excluded based on title and abstract mostly concerned neonatal respiratory outcomes, chorioamnionitis and infertility. Exclusion of full-text articles had various reasons (Figure S1).

Study characteristics

Of the 52 studies, we identified 42 reporting on *M. hominis* (proportion detected <1-70%), 31 reporting on *U. urealyticum* (proportion detected 0-91%) and 12 reporting on *U. parvum* (2–100%) and median sample size 250, interquartile range, IQR 145-613, range 37 [32] to 9105 [33] (Table 1, Supporting information Table S1). There were 26 cohort studies (Table S3.1),[1, 6, 8, 12, 15, 33-53] 25 case-control studies (Table S3.2)[7, 9-11, 32, 54-73] and six cross-sectional studies (Table S3.3).[74-79] Most studies were from high-income settings (39/57) (Table S4.1, S4.2, S4.3); ethnicity was reported in 24 studies, and maternal smoking in 12 (Table S5.1, S5.3, S5.3). Most studies (54/57) stated the timing of specimen collection, and all described the laboratory tests used (Table S1): 29/57 bacterial culture only; 24/57 NAAT only (Table 1, Table S1). Three studies reported on antimicrobial susceptibilities[8, 50] with *M. hominis* resistant to erythromycin, clarithromycin, tetracycline and *U. urealyticum* resistant to ciprofloxacin, tetracycline and erythromycin.[6, 50]

For peer review only

Table 1. Summary of characteristics of studies included in the systematic review

Characteristic	Total	*M. hominis*	*U. urealyticum*	*U. parvum*
Number of studies, n*	57	42	31	12
Study design, n				
Cohort	26	23	16	9
Case-control	25	13	12	1
Cross-sectional	6	6	3	2
Number of women, total	36,992	28,697	16,609	9,663
(median; IQR)	(250; 145-613)	(250; 159-759)	(216; 145-613)	(376; 195-986)
Study setting, income category, n				
High income	38	27	20	10
Upper-middle income	9	8	4	2
Lower middle-income or low	3	2	1	0
Not reported	11	5	6	0
Outcomes reported, n				
Preterm birth	43	29	27	11
Low birth weight	8	6	2	1
Premature ruptures of membrane	15	11	11	2
Spontaneous abortion	11	10	4	2
Perinatal death	11	10	2	1
Specimen type, n†				
Endocervical swab	24	18	12	4

For peer review only

Vaginal swab	15	10	11	5
Urine	1	1	0	0
Amnotic fluid	9	6	5	2
Placental membrane	8	7	3	2
Diagnostic method*				
NAAT	24	13	20	10
Culture	29	27	7	0
Culture and NAAT	3	1	3	2
Other**	1	1	0	0
Bacterial vaginosis assessed, n	10	8	3	1
Reported presence of STI, n	20	14	8	3
Reported on smoking status, n	13	7	6	4
Reported on Multiple pregnancy, n*‡				
Excluded	26	18	15	6
Included	8	5	4	3

Abbreviations: IQR, interquartile range; STI, sexually transmitted infection

* The total number of studies included is 57. The totals for each organism sum to more than 57 because one study might have reported on more than one organism;

† One study used both urine and endocervical swab;

**ELISA (with NAAT/ Culture)

‡ 22 studies included women with multiple pregnancy

For peer review only

Of the 57 studies, 37 reported on a single microorganism (*M. hominis*, n=27; *U. urealyticum*, n=10); 13 included two genital mycoplasmas (*M. hominis* and *U. urealyticum*, n=9; ureaplasmas, n=4) and seven reported on all three organisms (Figure S2). Only two studies presented findings for combinations of more than one genital mycoplasma;[6, 47] the rest presented data separately, even if they had tested for more than one organism. Ten studies reported on the presence of BV;[33, 36, 43, 47, 51, 53, 58, 59, 65, 72] we report the findings of these studies in the relevant section of the results for each genital mycoplasma. Twenty-three studies reported on other sexually transmitted infections (Table S5.1, S5.3, S5.3), including 2/23 reporting on syphilis, 5/23 gonorrhoea, 14/23 chlamydia, 5/23 *M. genitalium*, 5/23 trichomonas, and 2/23 HIV.

Table 2 summarises the meta-analyses of each exposure-outcome pair and information about genital mycoplasmas in the presence or absence of BV. In most meta-analyses, heterogeneity was low or moderate. Summary findings from different study designs were compatible, so we present summary measures across all study designs (Figures 1, 2, and S3.1-S5.3).

For peer review only

Table 2 Summary estimates, by outcome and organism, from random effects meta-analysis of unadjusted odds ratios, for associations between genital mycoplasmas and adverse birth outcomes, and summary of multivariable and analyses that stratify the main association by BV status

**Adverse outcome** ***Organism***	**No. of studies**	**Summary estimate* OR (95% CI)**	**I^2, %**	**Prediction interval**	**Any multivariable analysis†**	**Analyses of genital mycoplasmas and adverse birth outcomes in presence and absence of BV‡**
**Preterm birth**						
M. hominis	30	1.87 (1.49, 2.34)	29.2	0.98, 3.55	5 studies[1, 44, 45, 48, 61]	MH+,BV+/PTB OR 1.58 (95% CI 0.94, 2.77); MH+,BV-/PTB 1.18 (0.91, 1.52)[33]
U. urealyticum	27	1.84 (1.33, 2.54)	69.2	0.53, 6.36	5 studies[1, 41, 47, 65, 74]	UU+,BV+/PTB 0.47 (0.09, 3.31); UU+,BV-/PTB 1.15 (0.67, 1.98)[65]
U. parvum	11	1.59 (1.12, 2.27)	57.4	0.60, 4.26	2 studies[1, 47]	UP-,BV-/PTB; UP+,BV-/PTB Adjusted 1.6 (1.2, 2.1);<br>UP-,BV+/PTB aOR 1.6 (1.1, 2.3); UP+,BV+/PTB aOR 2.6 (1.7, 4.0)[47]
**Premature rupture of membrane**						
M. hominis	11	1.99 (1.43, 2.79)	0.0	1.36, 2.90	1 study[61]	None reported
U. urealyticum	11	4.27 (1.83, 9.98)	87.3	0.27, 68.07	0 studies	
U. parvum	2	3.19 (1.25, 8.15)	0.0	NC	0 studies	
**Low birth weight**						None reported
M. hominis	6	1.81 (1.29, 2.52)	0.0	1.12, 2.90	1 study[34]	
U. urealyticum	2	2.24 (1.16, 4.33)	0.0	NC	0 studies	
U. parvum	0	NA	NA	NA	0 studies	
**Spontaneous abortion**						None reported
M. hominis	10	1.06 (0.49, 2.30)	54.4	0.12, 9.68	0 studies	

For peer review only

U. urealyticum	4	1.74 (1.02, 2.95)	0.0	0.54, 5.58	0 studies	
U. parvum	2	1.65 (0.67, 4.05)	0.0	NC	0 studies	
**Perinatal death**						None reported
M. hominis	9	2.70 (1.31, 5.57)	30.4	1.31, 13.94	0 studies	
U. urealyticum	2	9.50 (2.99, 30.13)	0.0	NC	0 studies	
U. parvum	1	NA			0 studies	

Abbreviations: aOR, adjusted odds ratio; BV, bacterial vaginosis; CI, confidence interval; I^2, heterogeneity; MH, *Mycoplasma hominis*; NA, not applicable; NC, could not be calculated; OR, odds ratio; UP, *Ureaplasma parvum*; UU, *Ureaplasma urealyticum*

* Meta-analysis of unadjusted ORs, using random effects model

† Details for individual studies reported in Tables S5.1-5.3

‡ Further details of analyses based on exclusion of other infections, stratification, or multivariable analyses in Table S7

For peer review only

Risk of bias within and across studies

Based on the NICE checklists,[27, 28] none of the 57 studies met all or most (++/++) checklist criteria for internal and external validity, 29 studies met some (+/+)[7, 9, 11, 15, 32, 33, 36, 40, 41, 45-47, 50, 52, 56-58, 60, 62, 64, 65, 67-70, 72-74] and 17 met few or no checklist criteria (-/-)[6, 8, 10, 12, 38, 39, 42-44, 49, 53, 55, 61, 66, 71, 79] (Tables S7.1-7.3). Poor reporting of study methods meant that many items could not be assessed. In all study designs, control of confounding in most studies was poorly addressed or not addressed. Funnel plots for *M. hominis* (PTB, PROM, SA and PND), *U. urealyticum* (PTB, PROM) and *U. parvum* (PTB) did not show evidence of asymmetry (Table S2).

Associations between *M. hominis* and adverse pregnancy outcomes

There were 42 studies with data about *M. hominis*, reporting on 66 outcomes (Tables S3.1-S3.1). Of these, 30 included data about PTB.[1, 6, 8, 10, 15, 32, 33, 36, 38, 40, 42-46, 48, 50-54, 58, 59, 61, 66, 68, 69, 72, 73, 75] *M. hominis* was associated with PTB in meta-analysis of unadjusted ORs (19,576 women, summary OR 1.87, 95% CI 1.49, 2.34; I^2 29.2%; prediction interval 0.98, 3.55) (Figure 1). Five studies reporting a univariable association between *M. hominis* and PTB conducted multivariable analyses.[1, 44, 45, 48, 61] The association was attenuated in one (aOR 1.1, 95% CI 0.5, 2.5), after controlling for obstetric factors (previous PTB, miscarriage, multiple pregnancy and cervical incompetence).[61] In two others, authors reported no association with PTB <37 weeks, but subgroup analyses showed associations with PTB <35 [1] or <33[48] weeks. In two studies, no numerical results were reported (Table S3.1). In seven studies, authors also reported on BV.[33, 36, 43, 51, 58, 59, 72] In one study, the associations between *M. hominis*, BV and PTB could be examined in detail.[33] *M. hominis*, in the absence of BV, was less strongly associated with PTB (OR 1.18, 95% CI 0.91, 1.52) than in the presence of BV (OR 1.58, 95% CI 0.94, 2.77).

For peer review only

[Figure 1]

Eleven studies included data about PROM.[6, 10, 40, 44, 45, 52, 61, 71, 73, 75, 79] *M. hominis* was associated with PROM in meta-analysis of unadjusted ORs (4,303 women, summary OR 1.99, 95% CI 1.43, 2.75; I^2 0.0 %; predictive interval 1.36, 2.90) (Figure S3.1). In one study with a multivariable analysis, the association was attenuated (aOR 1.1, 95% CI 0.3, 3.7)[61]. Six studies included data about LBW.[8, 34, 35, 49, 75, 77] *M. hominis* was associated with LBW in meta-analysis of unadjusted ORs (2,394 new-borns, summary OR 1.81, 95% CI 1.29, 2.52; I^2 0.0 %; predictive interval 1.12, 2.90) (Figure S3.2). In one study, *M hominis* was associated with LBW in multivariable analysis, when considered as a continuous variable (reported p=0.01).[34] In 10 studies with data about PND,[8, 35, 40, 45, 51, 54, 55, 76, 77] meta-analysis of unadjusted ORs found an association with *M. hominis* (3,696 women, summary OR 2.09, 95% CI 1.00, 4.37; I^2 44.6%; predictive interval 0.30, 14.62) (Figure S3.3). In 10 studies with data about SA,[6, 7, 11, 35, 36, 39, 40, 51, 55, 63] there was no association with *M. hominis* in meta-analysis of unadjusted ORs (4,531 women, summary OR 1.06, 95% CI 0.49, 2.30 ; I^2 54.4%; predictive interval 0.12, 9.68) (Figure S3.4). No results of multivariable analyses were reported for PND or SA.

Associations between *U. urealyticum* and adverse pregnancy outcomes

Thirty-one studies included data about *U. urealyticum* and 46 outcomes. There were 27 studies with data about PTB.[1, 6, 10, 12, 15, 32, 38, 40, 41, 46-48, 50, 52, 53, 56, 57, 60, 62, 64, 65, 67, 70, 73-75, 78] In meta-analysis of unadjusted ORs, *U. urealyticum* was associated with PTB (12,234 women, summary OR 1.84, 95% CI 1.33, 2.54; I^2 69.2%; predictive interval 0.53, 6.36) (Figure 2). Five studies reported multivariable analyses.[1, 41, 47, 65, 74] In one, multivariable and univariable associations were similar (aOR 1.4, 95% CI 0.8, 2.2).[47] In four, no numerical results were reported.[1] In one study with information about

For peer review only

BV, there was no strong evidence of an association between *U. urealyticum* and PTB in the presence (OR 0.47, 95% CI 0.09, 3.31) or absence of BV (OR 1.15, 95% CI 0.67, 1.98).[65]

[Figure 2]

For all other outcomes, data were only available for meta-analysis of unadjusted ORs. *U. urealyticum* was associated with: PROM in 12 studies[6, 10, 37, 50, 52, 60, 62, 67, 71, 73, 74, 79] (3,676 participants, summary OR 4.27, 95% CI 1.83, 9.98; I^2 87.3%; predictive interval 0.27, 68.07) (Figure S4.1); LBW in two studies[12, 65] (506 participants, OR 2.24, 95% CI 1.16, 4.33; I^2 0.0%) (Figure S4.2); SA in four studies[6, 7, 9, 40] (2,140 women, summary OR 1.74, 95% CI 1.02, 2.95; I^2 0.0%; predictive interval 0.54, 5.58) (Figure S4.3); and PND in two studies[40, 60] (1,043 participants, summary OR 9.50, 95% CI 2.99, 30.13; I^2 0.0%) (Figure S4.4).

Associations between *U. parvum* and adverse pregnancy outcomes

Twelve studies included data about associations between *U. parvum* and 17 outcomes. Eleven studies reported PTB.[1, 10, 12, 15, 38, 40, 46, 47, 56, 64, 78] In meta-analysis of unadjusted ORs, *U. parvum* was associated with PTB (8,002 women, summary OR 1.59, 95% CI 1.12, 2.72; I^2 57.4%; predictive interval 0.60, 4.26) (Supplementary Figure S5.1). In one study,[47] a multivariable analysis found a stronger association with PTB when both *U. parvum* and BV were present (aOR 2.6, 95% CI 1.7, 4.0) than when *U. parvum* was present without BV (aOR 1.6, 95% CI 1.2, 2.1), when compared with women with neither infection. In one, no numerical results were reported.[1]

[Figure 3]

For all other outcomes, data were only available for meta-analysis of unadjusted ORs. *U. parvum* was associated with PROM in two studies[10, 40] (946 participants, OR 3.19, 95%

For peer review only

CI 1.25, 8.15; I^2 0.0%) (Figure S5.2) and with SA in two studies[7, 40] (986 participant, summary OR 1.65, 95% CI 0.67, 4.05; I^2 0.0%) (Figure S5.3). One study reported on LBW (22 participants, 1 event, OR 0.56, 95% CI 0.01, 12.75)[12] and one on PND (872 women, 1 event, OR 2.79).[40]

DISCUSSION

Principal findings

This systematic review and meta-analysis included 57 studies about associations between *M. hominis, U. urealyticum* and *U. parvum* and five adverse pregnancy outcomes. Only 6/57 studies reported any multivariable analysis. In 51 studies, meta-analyses of unadjusted ORs found that *M. hominis* was associated with an increase in PTB, PROM, LBW, and PND, *U. urealyticum* with an increase in PTB, PROM, SA, and PND, and *U. parvum* with an increase in PTB and PROM. In three studies from which data about both genital mycoplasmas and BV could be extracted; *M. hominis* and *U. parvum* were less strongly associated with PTB in the absence of BV than in the presence of BV and no association with *U. urealyticum* was found in the presence or absence of BV.

Strengths and weaknesses of the study

The strengths of this systematic review and meta-analysis are first, that we followed a published protocol[22] with predefined outcomes and statistical analysis plan. Study selection, data extraction and risk of bias assessment were undertaken independently by two reviewers, to reduce subjectivity. Second, we examined evidence for heterogeneity visually and statistically, and calculated prediction intervals that show the variability in estimates from different studies.[31] Third, we triangulated findings across study designs;[23, 26] despite the different potential sources of bias, the summary estimates were compatible and we

For peer review only

judged it reasonable to combine effect estimates.[30] There were also limitations in the design of the review. Despite a predefined search strategy, with broad search terms, we might have missed relevant studies, particularly by restriction to languages not spoken fluently by the authors. There were too few studies to conduct all the planned sensitivity analyses by organism, but we described all studies that allowed stratification by BV status.

Comparison with existing literature and interpretation

We found a systematic review about genital mycoplasmas that included studies published in English or Chinese up to March 2020.[80] The focus of the review was on infertility, however, and limited search terms for studies about adverse pregnancy outcomes identified only 11 of the 57 studies that we included, making comparison difficult.

The findings from this systematic review cannot be interpreted as showing causal associations between colonisation with *M. hominis*, *U. urealyticum*, or *U. parvum* in pregnancy and some adverse pregnancy outcomes. Whilst meta-analysis of unadjusted associations increases precision, the confounder adjusted estimates could not be summarised. Most studies in this systematic review did not control for confounding by either sociodemographic characteristics, or co-infection with another organism or BV. Specific investigation of the role of co-infection with BV,[4, 5] could only be studied in a small number of studies. Rittenschober-Böhm *et al.*, studied more than 4000 women in Germany.[47] They found univariable associations between both *U. parvum* (OR 1.7, 95% CI 1.3, 2.2) and *U. urealyticum* (1.4, 95% CI 0.9, 2.3) and spontaneous PTB. A strength of their study is the multivariable analysis, controlling for age, smoking, history of PTB and other infections. For *U. parvum,* the association with PTB was stronger when both BV and *U. parvum* were present than for *U. parvum* alone. The authors did not analyse the association with *U. urealyticum* further. Hillier *et al.*, investigated the association between *M. hominis*

For peer review only

and PTB of LBW infants in more than 10,000 women in the USA.[33] The association was stronger in the presence (1.58, 95% CI 0.94, 2.77) than absence (1.18, 95% CI 0.91, 1.52) of BV, but confidence intervals for both estimates include the null value. Hillier *et al*. also reported a stronger association with PTB when *M. hominis* was present with Bacteroides and BV (OR 2.1, 95% CI 1.5, 3.0). The authors did not, however, control for any other confounding factors.

Several of the limitations that we found in our review apply to systematic reviews of observational studies in general. Most included studies did not set out to study our review question and have small sample sizes. We extracted most data about genital mycoplasmas, our exposures of interest, from tables of covariates. Differences in the performance characteristics of diagnostic methods might have resulted in misclassification of infection status. Bacteriological culture has been considered the gold standard for the identification of genital mycoplasmas, but problems can arise from their fastidious growth requirements and a lack of reliable media. Commercialised kits for both culture and NAAT diagnosis are less laborious and have greater sensitivity and specificity compared with earlier in-house approaches.[81, 82] Sample integrity is also important and greatly influenced by sample collection methods (e.g. type of swab, transport medium), transportation (e.g. cold chain maintenance) and storage (e.g. duration and temperature at which kept in long-term storage). It was not possible to account for differences in anatomical sampling site that may have affected detection in individual studies, e.g. *M. hominis* is more commonly isolated in the lower genital tract whilst *Ureaplasma* spp. colonise the upper genital tract.[83] Other limitations include misclassification, for example, gestational age was assessed by obstetric ultrasound in only one third of studies and inconsistency in the timing during pregnancy of sampling for genital mycoplasmas.

For peer review only

The specificity of associations between different genital mycoplasmas and adverse pregnancy, and their mechanisms of action, remain unclear. Several studies included in this review postulate that subclinical ascending *Ureaplasma* spp. to the choriodecidual space is followed by placental transfer into the amniotic cavity,[7, 76, 78, 84, 85] which then leads to PROM, SA, and PND in women with high bacterial load in the upper genital tract.[85, 86] The presence of genital mycoplasmas in the placental membranes and amniotic fluid might have a direct effect, but they also increase levels of a variety of cytokines and other inflammatory mediators, which might be the key drivers of adverse pregnancy outcomes.[32, 37, 52, 64, 67, 85, 87] Gene sequencing methods show the complexity and diversity of the vaginal microbiota during pregnancy [15, 16, 88] and genital mycoplasmas are often among the most plentiful of the many bacterial species identified. In our review, one study using 16s rRNA sequencing found a group of bacteria, including *U. parvum*, that was associated with PTB,[15] but another smaller study did not.[56] Analysis of associations between microbial communities and PTB was beyond the scope of our systematic review. A better understanding of antimicrobial susceptibility is also needed. Genital mycoplasmas lack a rigid cell wall, which allows them to evade some antibiotics. Beta-lactam antibiotics and vancomycin are considered ineffective but macrolides, fluoroquinolones and tetracyclines are often effective.[89] In pregnant women, only macrolides should be used[90] but high rates of antibiotic resistance are reported in many settings,[4, 91, 92] and in the absence of definitive evidence of the benefits of treatment, cannot currently be recommended.

Implications

The findings of this systematic review show key areas for future research. First, there is a need for epidemiological studies that are designed specifically to investigate the pathogenicity of vaginal and cervical organisms alone and in the context of the vaginal microbiome. A holistic approach that includes gene sequencing and other molecular and

For peer review only

culture methods to detect other endogenous and sexually transmitted organisms is required,[14-16] taking into account the need for consistent strategies for specimen collection both in terms of the trimester(s) and the timing and types of specimens collected. These studies should also define potential causal pathways and address confounding from factors such as maternal age, smoking, obstetric history, co-infections and comorbidities. Second, there is a critical need to conduct research in low- and middle-income settings where the prevalence of sexually transmitted infections, BV and genital mycoplasma are high, and the burden of adverse pregnancy outcomes greatest. If consistent and reproducible associations are found in observational studies, potential interventions need to be evaluated. Randomised controlled trials of screening and treatment for a range of vaginal and endocervical infections in pregnancy are underway.[93, 94] If these interventions prevent adverse pregnancy outcomes, further research will still be needed to understand the contributions of specific organisms or combinations thereof. Multiplex assays will facilitate these research studies but should not be used in routine clinical practice because of the risks of overdiagnosis and overtreatment.[18, 19]

Conclusions

In this systematic review and meta-analysis, we found that genital mycoplasmas are associated with several different adverse pregnancy outcomes in univariable analysis only. The currently available literature does not allow conclusions about the role of mycoplasmas in adverse pregnancy and birth outcomes, alone or with co-existing bacterial vaginosis. Future studies that consider genital mycoplasmas in the context of the vaginal microbiome are needed.

For peer review only

Authors' roles

DEG, NL, AV, LV conceived the idea for the review and DEG, JK, NL, AV, LV, HW wrote the protocol. MJ and LV did the searches, screened, and selected studies and extracted data. DEG, NL, ES resolved disagreements. NL and HW did statistical analyses. MJ wrote the first draft of the manuscript. MJ, NL did review and editing. All authors commented on revisions of the manuscript and accept responsibility for its content.

Funding

NL receives funding from the Swiss National Science Foundation, project numbers 197831, 160909; LV is supported by an Australian National Health & Medical Research Council (NHMRC) Early Career Fellowship Grant (2018-2021); MJ is a PhD research student is supported through the Women And Newborns Trial of Antenatal Interventions and Management (WANTAIM) trial (ISRCTN No: ISRCTN37134032), funded by DFID/MRC/Wellcome Trust Joint Global Health Trials, Australian NHMRC Grant and Swiss National Science Foundation. DEG received salary support from r4d programme (Swiss Programme for Research on Global Issues for Development), grant number IZ07Z0-160909. AV receives salary support from the Australian NHMRC, through a Career Development Fellowship.

For peer review only

Ethics

This study does not involve huma participants or animal subjects. All data used are only from published data. Patients or the public were not involved in the design, or conduct, or reporting, or dissemination plans of our research.

Data availability

No additional or unpublished data available.

Conflict of interests

NL is on the advisory board of Sefunda AG, a start-up company that develops point-of-care tests for sexually transmitted infections.

For peer review only

REFERENCES

1. Agger WA, Siddiqui D, Lovrich SD, et al. Epidemiologic factors and urogenital infections associated with preterm birth in a midwestern U.S. population. *Obstet Gynecol* 2014;124(5):969-77. doi: https://dx.doi.org/10.1097/AOG.0000000000000470

2. Plummer EL, Vodstrcil LA, Bodiyabadu K, et al. Are Mycoplasma hominis, Ureaplasma urealyticum and Ureaplasma parvum Associated With Specific Genital Symptoms and Clinical Signs in Nonpregnant Women? *Clin Infect Dis* 2021

3. Horner P, Donders G, Cusini M, et al. Should we be testing for urogenital Mycoplasma hominis, Ureaplasma parvum and Ureaplasma urealyticum in men and women?–a position statement from the European STI Guidelines Editorial Board. *J Eur Acad Dermatol Venereol* 2018;32(11):1845-51.

4. Capoccia R, Greub G, Baud D. Ureaplasma urealyticum, Mycoplasma hominis and adverse pregnancy outcomes. *Curr Opin Infect Dis* 2013;26(3):231-40. doi: 10.1097/QCO.0b013e328360db58

5. Taylor-Robinson D, Lamont R. Mycoplasmas in pregnancy. *BJOG: Int J Obstet Gy* 2011;118(2):164-74.

6. Lee MY, Kim MH, Lee WI, et al. Prevalence and Antibiotic Susceptibility of Mycoplasma hominis and Ureaplasma urealyticum in Pregnant Women. *Yonsei Med J* 2016;57(5):1271-5. doi: https://dx.doi.org/10.3349/ymj.2016.57.5.1271

7. Oliveira C, Oliveira M, Oliveira H, et al. Association of spontaneous abortion and Ureaplasma parvum detected in placental tissue. *Epidemiol Infect* 2020;148:e126. doi: 10.1017/S0950268820001302 [published Online First: 2020/07/07]

8. Luton D, Ville Y, Luton-Sigy A, et al. Prevalence and influence of Mycoplasma hominis and Ureaplasma urealyticum in 218 African pregnant women and their infants. *Eur J Obstet Gynecol Reprod Biol* 1994;56(2):95-101.

9. Ahmadi A, Khodabandehloo M, Ramazanzadeh R, et al. Association between Ureaplasma urealyticum endocervical infection and spontaneous abortion in in Sanandaj, Iran. *Iran J Microbiol* 2014;6(6):392-97.

For peer review only

10. Jones HE, Harris KA, Azizia M, et al. Differing prevalence and diversity of bacterial species in fetal membranes from very preterm and term labor. *PLoS One* 2009;4 (12) (no pagination)(e8205) doi: http://dx.doi.org/10.1371/journal.pone.0008205

11. Farhadifar F, Khodabandehloo M, Ramazanzadeh R, et al. Survey on association between Mycoplasma hominis endocervical infection and spontaneous abortion using Polymerase Chain Reaction. *Int J Reprod Biomed* 2016;14(3):181-86.

12. Peretz A, Tameri O, Azrad M, et al. Mycoplasma and Ureaplasma carriage in pregnant women: the prevalence of transmission from mother to newborn. *BMC Pregnancy Childbirth* 2020;20(1):456. doi: 10.1186/s12884-020-03147-9

13. Donders GG, Ruban K, Bellen G, et al. Mycoplasma/Ureaplasma infection in pregnancy: to screen or not to screen. *J Perinat Med* 2017;45(5):505-15. doi: 10.1515/jpm-2016-0111

14. van de Wijgert JH. The vaginal microbiome and sexually transmitted infections are interlinked: consequences for treatment and prevention. *PLoS Med* 2017;14(12):e1002478.

15. Payne MS, Newnham JP, Doherty DA, et al. A Specific Bacterial DNA Signature in the Vagina of Australian Women in Mid-Pregnancy Predicts High Risk of Spontaneous Preterm Birth (The Predict1000 Study). *Am J Obstet Gynecol* 2021 doi: 10.1016/j.ajog.2020.08.034 [published Online First: 2020/08/31]

16. Pace RM, Chu DM, Prince AL, et al. Complex species and strain ecology of the vaginal microbiome from pregnancy to postpartum and association with preterm birth. *Med (N Y)* 2021;2(9):1027-49. doi: 10.1016/j.medj.2021.06.001 [published Online First: 2021/10/08]

17. Vouga M, Greub G, Prod'hom G, et al. Treatment of genital mycoplasma in colonized pregnant women in late pregnancy is associated with a lower rate of premature labour and neonatal complications. *Clin Microbiol Infect* 2014;20(10):1074-79. doi: http://dx.doi.org/10.1111/1469-0691.12686

18. Jensen JS. To Test or Not to Test for Mycoplasma hominis and Ureaplasmas: That's (Not) the Question. *Clin Infect Dis* 2021;73(4):669-71. doi: 10.1093/cid/ciab065 [published Online First: 2021/01/26]

For peer review only

19. Taylor-Robinson D, Horner P, Pallecaros A. Diagnosis of some genital-tract infections: part 2. Molecular tests and the new challenges. *Int J STD AIDS* 2020;31(3):198-207. doi: 10.1177/0956462419890526 [published Online First: 2020/02/06]

20. Kong F, Ma Z, James G, et al. Species identification and subtyping of Ureaplasma parvum and Ureaplasma urealyticum using PCR-based assays. *J Clin Microbiol* 2000;38(3):1175-9.

21. Robertson JA, Stemke GW, Davis Jr JW, et al. Proposal of Ureaplasma parvum sp. nov. and emended description of Ureaplasma urealyticum (Shepard et al. 1974) Robertson et al. 2001. *Int J Syst Evol Microbiol* 2002;52(2):587-97.

22. Vallely LM, Egli-Gany D, Pomat W, et al. Adverse pregnancy and neonatal outcomes associated with Neisseria gonorrhoeae, Mycoplasma genitalium, M. hominis, Ureaplasma urealyticum and U. parvum: a systematic review and meta-analysis protocol. *BMJ Open* 2018;8(11)

23. Vallely LM, Egli-Gany D, Wand H, et al. Adverse pregnancy and neonatal outcomes associated with Neisseria gonorrhoeae: systematic review and meta-analysis. *Sex Transm Infect* 2021;97(2):104-11.

24. Frenzer C, Egli-Gany D, Vallely LM, et al. Adverse pregnancy and neonatal outcomes associated with Mycoplasma genitalium: systematic review and meta-analysis [In Press]. *Sex Transm Infect* 2022;Epub ahead of print: doi: doi:10.1136/sextrans-2021-055352

25. Page MJ, McKenzie JE, Bossuyt PM, et al. The PRISMA 2020 statement: an updated guideline for reporting systematic reviews. *BMJ* 2021;372:n71. doi: 10.1136/bmj.n71 [published Online First: 2021/03/31]

26. Dekkers OM, Vandenbroucke JP, Cevallos M, et al. COSMOS-E: guidance on conducting systematic reviews and meta-analyses of observational studies of etiology. *PLoS Med* 2019;16(2):e1002742.

27. National Institute for Health Care Excellence. The social care guidance manual. Great Britain: National Institute for Health and Care Excellence, 2016.

For peer review only

28. National Institute for Health Care Excellence. Methods for the development of NICE public health guidance. Great Britain: National Institute for Health and Care Excellence 2012.

29. Egger M, Davey SG, Schneider M, et al. Bias in meta-analysis detected by a simple, graphical test. *BMJ* 1997;315(7109):629-34.

30. Low N. Chlamydia trachomatis and reproductive health: what can we learn from systematic reviews of observational studies? *Sex Transm Infect* 2020 doi: 10.1136/sextrans-2019-054279 [published Online First: 2020/01/29]

31. Riley RD, Higgins JP, Deeks JJ. Interpretation of random effects meta-analyses. *BMJ* 2011;342:d549.

32. Daskalakis G, Thomakos N, Papapanagiotou A, et al. Amniotic fluid interleukin-18 at mid-trimester genetic amniocentesis: Relationship to intraamniotic microbial invasion and preterm delivery. *BJOG: Int J Obstet Gy* 2009;116(13):1743-48. doi: http://dx.doi.org/10.1111/j.1471-0528.2009.02364.x

33. Hillier SL, Nugent RP, Eschenbach DA, et al. Association between bacterial vaginosis and preterm delivery of a low-birth-weight infant. *N Engl J Med* 1995;333(26):1737-42.

34. Berman SM, Harrison HR, Boyce WT, et al. Low birth weight, prematurity, and postpartum endometritis. Association with prenatal cervical Mycoplasma hominis and Chlamydia trachomatis infections. *JAMA* 1987;257(9):1189-94. [published Online First: 1987/03/06]

35. Braun P, Lee YH, Klein JO, et al. Birth Weight and Genital Mycoplasmas in Pregnancy. *N Engl J Med* 1971;284(4):167-71. doi: 10.1056/NEJM197101282840401

36. Donders GG, Van Calsteren K, Bellen G, et al. Predictive value for preterm birth of abnormal vaginal flora, bacterial vaginosis and aerobic vaginitis during the first trimester of pregnancy. *BJOG: Int J Obstet Gy* 2009;116(10):1315-24. doi: https://dx.doi.org/10.1111/j.1471-0528.2009.02237.x

37. Gerber S, Vial Y, Hohlfeld P, et al. Detection of Ureaplasma urealyticum in second-trimester amniotic fluid by polymerase chain reaction correlates with subsequent preterm labor and delivery. *J Infect Dis* 2003;187(3):518-21. doi: https://dx.doi.org/10.1086/368205

For peer review only

38. Govender S, Theron GB, Odendaal HJ, et al. Prevalence of genital mycoplasmas, ureaplasmas and chlamydia in pregnancy. *J Obstet Gynaecol* 2009;29(8):698-701. doi: 10.3109/01443610903184033 [published Online First: 2009/10/14]

39. Harrison HR, Alexander ER, Weinstein L, et al. Cervical Chlamydia trachomatis and mycoplasmal infections in pregnancy. Epidemiology and outcomes. *JAMA* 1983;250(13):1721-7. [published Online First: 1983/10/07]

40. Kataoka S, Yamada T, Chou K, et al. Association between preterm birth and vaginal colonization by mycoplasmas in early pregnancy. *J Clin Microbiol* 2006;44(1):51-5. doi: https://dx.doi.org/10.1128/JCM.44.1.51-55.2006

41. Koucky M, Malickova K, Cindrova-Davies T, et al. Prolonged progesterone administration is associated with less frequent cervicovaginal colonization by Ureaplasma urealyticum during pregnancy - Results of a pilot study. *J Reprod Immunol* 2016;116:35-41. doi: http://dx.doi.org/10.1016/j.jri.2016.04.285

42. McDonald HM, O'Loughlin JA, Jolley PT, et al. Changes in vaginal flora during pregnancy and association with preterm birth. *J Infect Dis* 1994;170(3):724-8.

43. Menard JP, Mazouni C, Salem-Cherif I, et al. High vaginal concentrations of atopobium vaginae and gardnerella vaginalis in women undergoing preterm labor. *Obstet Gynecol* 2010;115(1):134-40. doi: http://dx.doi.org/10.1097/AOG.0b013e3181c391d7

44. Minkoff H, Grunebaum AN, Schwarz RH. Risk factors for prematurity and premature rupture of membranes: A prospective study of the vaginal flora in pregnancy. *Am J Obstet Gynecol* 1984;150(8):965-72.

45. Nguyen DP, Gerber S, Hohlfeld P, et al. Mycoplasma hominis in mid-trimester amniotic fluid: relation to pregnancy outcome. *J Perinat Med* 2004;32(4):323-6. doi: 10.1515/JPM.2004.060 [published Online First: 2004/09/07]

46. Payne MS, Ireland DJ, Watts R, et al. Ureaplasma parvum genotype, combined vaginal colonisation with Candida albicans, and spontaneous preterm birth in an Australian cohort of pregnant women. *BMC Pregnancy Childbirth* 2016;16:312-12.

For peer review only

47. Rittenschober-Böhm J, Waldhoer T, Schulz SM, et al. First Trimester Vaginal Ureaplasma Biovar Colonization and Preterm Birth: Results of a Prospective Multicenter Study. *Neonatology* 2018;113(1):1-6. doi: http://dx.doi.org/10.1159/000480065 [published Online First: 2017/09/22]

48. Usui R, Ohkuchi A, Matsubara S, et al. Vaginal lactobacilli and preterm birth. *J Perinat Med* 2002;30(6):458-66. doi: 10.1515/JPM.2002.072 [published Online First: 2003/01/18]

49. Sperling RS, Newton E, Gibbs RS. Intraamniotic Infection in Low-Birth-Weight Infants. *J Infect Dis* 1988;157(1):113-17.

50. Kwak DW, Hwang HS, Kwon JY, et al. Co-infection with vaginal Ureaplasma urealyticum and Mycoplasma hominis increases adverse pregnancy outcomes in patients with preterm labor or preterm premature rupture of membranes. *J Matern -Fetal Neonatal Med* 2014;27(4):333-7. doi: https://dx.doi.org/10.3109/14767058.2013.818124

51. Odendaal HJ, Popov I, Schoeman J, et al. Preterm labour--is Mycoplasma hominis involved? *S Afr Med J* 2002;92(3):235-7. [published Online First: 2002/06/04]

52. Perni SC, Vardhana S, Korneeva I, et al. Mycoplasma hominis and Ureaplasma urealyticum in midtrimester amniotic fluid: association with amniotic fluid cytokine levels and pregnancy outcome. *Am J Obstet Gynecol* 2004;191(4):1382-6. doi: 10.1016/j.ajog.2004.05.070 [published Online First: 2004/10/28]

53. Schwab FD, Zettler EK, Moh A, et al. Predictive factors for preterm delivery under rural conditions in post-tsunami Banda Aceh. *J Perinat Med* 2015;44(5):511-5. doi: 10.1515/jpm-2015-0004 [published Online First: 2015/05/20]

54. Cassell GH, Davis RO, Waites KB, et al. Isolation of Mycoplasma hominis and Ureaplasma urealyticum from amniotic fluid at 16-20 weeks of gestation: Potential effect on outcome of pregnancy. *Sex Transm Dis* 1983;10(4 SUPPL.):294-302.

55. Embree JE, Krause VW, Embil JA, et al. Placental infection with Mycoplasma homonis and Ureaplasma urealyticum: clinical correlation. *Obstet Gynecol* 1980;56(4):475-81.

For peer review only

56. Freitas AC, Bocking A, Hill JE, et al. Increased richness and diversity of the vaginal microbiota and spontaneous preterm birth. *Microbiome* 2018;6(1):117. doi: 10.1186/s40168-018-0502-8 [published Online First: 2018/06/30]

57. Harada K, Tanaka H, Komori S, et al. Vaginal infection with Ureaplasma urealyticum accounts for preterm delivery via induction of inflammatory responses. *Microbiol Immunol* 2008;52(6):297-304. doi: https://dx.doi.org/10.1111/j.1348-0421.2008.00039.x

58. Hillier SL, Martius J, Krohn M, et al. A case-control study of chorioamnionic infection and histologic chorioamnionitis in prematurity. *N Engl J Med* 1988;319(15):972-78.

59. Holst E, Goffeng AR, Andersch B. Bacterial vaginosis and vaginal microorganisms in idiopathic premature labor and association with pregnancy outcome. *J Clin Microbiol* 1994;32(1):176-86.

60. Kafetzis DA, Skevaki CL, Skouteri V, et al. Maternal genital colonization with Ureaplasma urealyticum promotes preterm delivery: association of the respiratory colonization of premature infants with chronic lung disease and increased mortality. *Clin Infect Dis* 2004;39(8):1113-22. doi: https://dx.doi.org/10.1086/424505

61. McDonald HM, O'Loughlin JA, Jolley P, et al. Prenatal microbiological risk factors associated with preterm birth. *Br J Obstet Gynaecol* 1992;99(3):190-6. [published Online First: 1992/03/01]

62. Mitsunari M, Yoshida S, Deura I, et al. Cervical Ureaplasma urealyticum colonization might be associated with increased incidence of preterm delivery in pregnant women without prophlogistic microorganisms on routine examination. *J Obstet Gynaecol Res* 2005;31(1):16-21. doi: https://dx.doi.org/10.1111/j.1447-0756.2005.00246.x

63. Munday PE, Porter R, Falder PF. Spontaneous abortion, an infectious aetiology? *Br J Obstet Gynaecol* 1984;91(12):1177-80.

64. Payne MS, Feng Z, Li S, et al. Second trimester amniotic fluid cytokine concentrations, Ureaplasma sp. colonisation status and sexual activity as predictors of preterm birth in Chinese and Australian women. *BMC Pregnancy Childbirth* 2014;14:340. doi: https://dx.doi.org/10.1186/1471-2393-14-340

For peer review only

65. Povlsen K, Thorsen P, Lind I. Relationship of Ureaplasma urealyticum biovars to the presence or absence of bacterial vaginosis in pregnant women and to the time of delivery. *Eur J Clin Microbiol Infect Dis* 2001;20(1):65-7. [published Online First: 2001/03/14]

66. Toth KS, Letchworth AT, Noble AD, et al. The significance of infection in the aetiology of preterm labour. A prospective controlled study. *J Obstet Gynaecol* 1992;12(2):94-99.

67. Yoon BH, Oh SY, Romero R, et al. An elevated amniotic fluid matrix metalloproteinase-8 level at the time of mid-trimester genetic amniocentesis is a risk factor for spontaneous preterm delivery. *Am J Obstet Gynecol* 2001;185(5):1162-7. doi: https://dx.doi.org/10.1067/mob.2001.117678

68. Gonzàlez Bosquet E, Gene A, Ferrer I, et al. Value of endocervical Ureaplasma species colonization as a marker of preterm delivery. *Gynecol Obstet Invest* 2006;61(3):119-23. doi: http://dx.doi.org/10.1159/000089457

69. Chua KB, Ngeow YF, Lim CT, et al. Colonization and transmission of Ureaplasma urealyticum and Mycoplasma hominis from mothers to full and preterm babies by normal vaginal delivery. *Med J Malaysia* 1999;54(2):242-6. [published Online First: 2000/09/06]

70. Jalava J, Laurikainen E, Karkkainen U, et al. Cervical ureaplasma urealyticum colonization: comparison of PCR and culture for its detection and association with preterm birth. *Scand J Infect Dis* 2002;34(1):35-40.

71. Kacerovsky M, Pavlovsky M, Tosner J. Preterm premature rupture of the membranes and genital mycoplasmas. *Acta Medica (Hradec Kralove)* 2009;52(3):117-20.

72. Kumar SS, V.; Sharma, M. Bacterial vaginosis in preterm labor. *Int J Gynaecol Obstet* 2006;95(1):40-41. doi: http://dx.doi.org/10.1016/j.ijgo.2006.05.022

73. Montenegro DA, Borda LF, Neuta Y, et al. Oral and uro-vaginal intra-amniotic infection in women with preterm delivery: A case-control study. *J Investig Clin Dent* 2019;10(2):e12396. doi: http://dx.doi.org/10.1111/jicd.12396

74. Abele-Horn M, Scholz M, Wolff C, et al. High-density vaginal Ureaplasma urealyticum colonization as a risk factor for chorioamnionitis and preterm delivery. *Acta Obstet Gynecol Scand* 2000;79(11):973-8.

75. Grattard F, Soleihac B, De Barbeyrac B, et al. Epidemiologic and molecular investigations of genital mycoplasmas from women and neonates at delivery. *Pediatr Infect Dis J* 1995;14(10):853-8.

76. Kundsin RB, Driscoll SG, Monson RR, et al. Association of Ureaplasma urealyticum in the placenta with perinatal morbidity and mortality. *N Engl J Med* 1984;310(15):941-45.

77. McCormack WM, Rosner B, Lee YH, et al. Isolation of genital mycoplasmas from blood obtained shortly after vaginal delivery. *Lancet* 1975;1(7907):596-9.

78. Sweeney EL, Kallapur SG, Gisslen T, et al. Placental Infection With Ureaplasma species Is Associated With Histologic Chorioamnionitis and Adverse Outcomes in Moderately Preterm and Late-Preterm Infants. *J Infect Dis* 2016;213(8):1340-47. doi: 10.1093/infdis/jiv587

79. Nasution TA, Cheong SF, Lim CT, et al. Multiplex PCR for the detection of urogenital pathogens in mothers and newborns. *Malays J Pathol* 2007;29(1):19-24.

80. Ma C, Du J, Dou Y, et al. The Associations of Genital Mycoplasmas with Female Infertility and Adverse Pregnancy Outcomes: a Systematic Review and Meta-analysis. *Reprod Sci* 2021:1-19.

81. D'Inzeo T, De Angelis G, Fiori B, et al. Comparison of Mycoplasma IES, Mycofast Revolution and Mycoplasma IST2 to detect genital mycoplasmas in clinical samples. *J Infect Dev Ctries* 2017;11(01):98-101.

82. Kusanovic JP, Vargas P, Ferrer F, et al. Comparison of two identification and susceptibility test kits for Ureaplasma spp and Mycoplasma hominis in amniotic fluid of patients at high risk for intra-amniotic infection. *J Matern -Fetal Neonatal Med* 2020;33(20):3409-17. doi: 10.1080/14767058.2019.1572742

83. Taylor-Robinson D. Infections Due to Species of Mycoplasma and Ureaplasma: An Update. *Clin Infect Dis* 1996;23(4):671-82. doi: 10.1093/clinids/23.4.671

84. Pavlidis I, Spiller OB, Demarco GS, et al. Cervical epithelial damage promotes Ureaplasma parvum ascending infection, intrauterine inflammation and preterm birth induction in mice. *Nat Commun* 2020;11(1):1-12.

For peer review only

85. Kasper DC, Mechtler TP, Reischer GH, et al. The bacterial load of Ureaplasma parvum in amniotic fluid is correlated with an increased intrauterine inflammatory response. *Diagn Microbiol Infect Dis* 2010;67(2):117-21. doi: https://dx.doi.org/10.1016/j.diagmicrobio.2009.12.023

86. Witt A, Berger A, Gruber CJ, et al. Increased intrauterine frequency of Ureaplasma urealyticum in women with preterm labor and preterm premature rupture of the membranes and subsequent cesarean delivery. *Am J Obstet Gynecol* 2005;193(5):1663-9. doi: 10.1016/j.ajog.2005.03.067 [published Online First: 2005/11/02]

87. Li Y-H, Brauner A, Jonsson B, et al. Ureaplasma urealyticum-induced production of proinflammatory cytokines by macrophages. *Pediatr Res* 2000;48(1):114-19.

88. Doyle RM, Alber DG, Jones HE, et al. Term and preterm labour are associated with distinct microbial community structures in placental membranes which are independent of mode of delivery. *Placenta* 2014;35(12):1099-101. doi: https://dx.doi.org/10.1016/j.placenta.2014.10.007

89. Combaz-Söhnchen N, Kuhn A. A systematic review of Mycoplasma and Ureaplasma in urogynaecology. *Geburtshilfe Frauenheilkd* 2017;77(12):1299.

90. Redelinghuys MJ, Ehlers MM, Dreyer AW, et al. Antimicrobial susceptibility patterns of Ureaplasma species and Mycoplasma hominis in pregnant women. *BMC Infect Dis* 2014;14:171. doi: https://dx.doi.org/10.1186/1471-2334-14-171

91. Bae I, Koh E, Kim S, et al. Prevalence rate and antimicrobial susceptibilities of Ureaplasma urealyticum and Mycoplasma hominis in pregnant women residing in Jinju, Korea. *Clin Microbiol Infect* 2010;2):S485. doi: http://dx.doi.org/10.1111/j.1469-0691.2010.03239.x

92. Bayraktar MR, Ozerol IH, Gucluer N, et al. Prevalence and antibiotic susceptibility of Mycoplasma hominis and Ureaplasma urealyticum in pregnant women. *Int J Infect Dis* 2010;14(2):e90-5. doi: https://dx.doi.org/10.1016/j.ijid.2009.03.020

93. Vallely AJ, Pomat WS, Homer C, et al. Point-of-care testing and treatment of sexually transmitted infections to improve birth outcomes in high-burden, low-income settings: Study

For peer review only

protocol for a cluster randomized crossover trial (the WANTAIM Trial, Papua New Guinea). *Wellcome Open Res* 2019;4

94. Grant JS, Chico RM, Lee AC, et al. Sexually transmitted infections in pregnancy: a narrative review of the global research gaps, challenges, and opportunities. *Sex Transm Dis* 2020;47(12):779.

For peer review only

Figure legends

Figure 1. Forest plot of univariable association between *M. hominis* and preterm birth, from random effects meta-analysis. Studies are in order of precision. Solid diamonds and lines either side are point estimates and 95% confidence intervals for individual studies (arrows show where lower or upper confidence limits extend beyond the x-axis limits). Open diamond shows the point estimate and 95% confidence interval for the summary odds ratio and lines either side of the diamond show the predictive interval.

Figure 2. Forest plot of univariable association between *U. urealyticum* and preterm birth, from random effects meta-analysis. Studies are in order of sample size. Solid diamonds and lines either side are point estimates and 95% confidence intervals for individual studies (arrows show where lower or upper confidence limits extend beyond the x-axis limits). Open diamond shows the point estimate and 95% confidence interval for the summary odds ratio and lines either side of the diamond show the predictive interval.

Figure 3. Forest plot of univariable association between *U. parvum* and preterm birth, from random effects meta-analysis. Studies are in order of precision. Solid diamonds and lines either side are point estimates and 95% confidence intervals for individual studies (arrows show where lower or upper confidence limits extend beyond the x-axis limits). Open diamond shows the point estimate and 95% confidence interval for the summary odds ratio and lines either side of the diamond show the predictive interval.

For peer review only

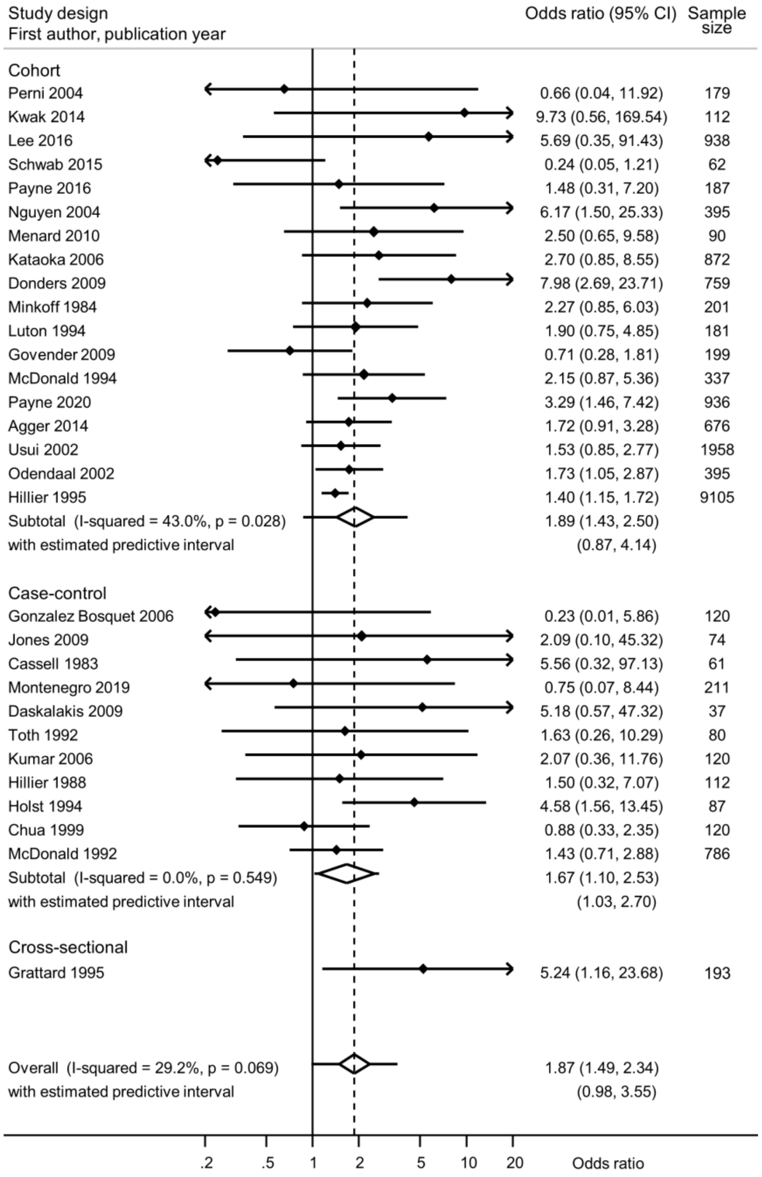

Figure 1. Forest plot of univariable association between M. hominis and preterm birth, from random effects meta-analysis. Studies are in order of precision. Solid diamonds and lines either side are point estimates and 95% confidence intervals for individual studies (arrows show where lower or upper confidence limits extend beyond the x-axis limits). Open diamond shows the point estimate and 95% confidence interval for the summary odds ratio and lines either side of the diamond show the predictive interval.

170x270mm (300 x 300 DPI)

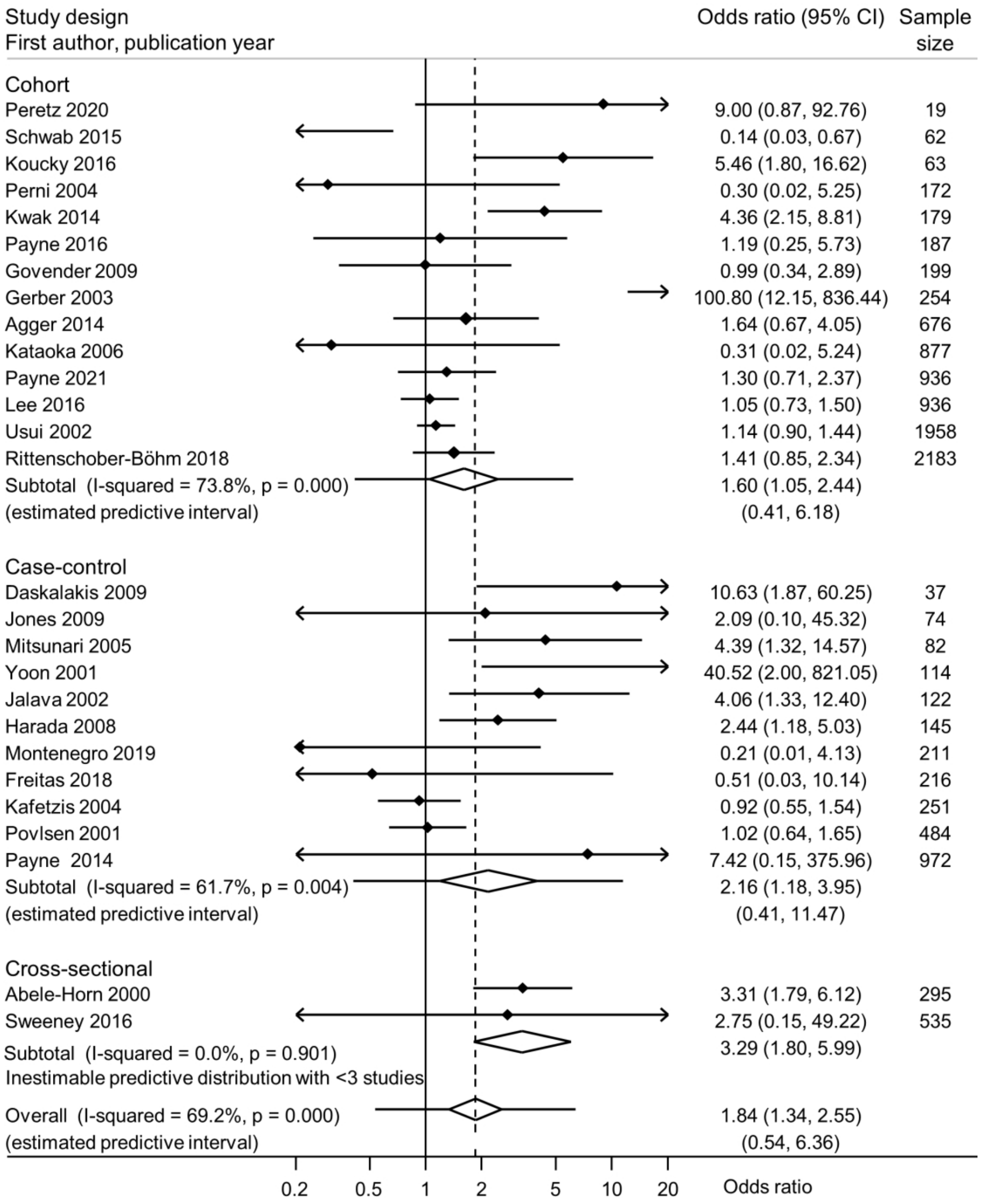

Figure 2. Forest plot of univariable association between U. urealyticum and preterm birth, from random effects meta-analysis. Studies are in order of sample size. Solid diamonds and lines either side are point estimates and 95% confidence intervals for individual studies (arrows show where lower or upper confidence limits extend beyond the x-axis limits). Open diamond shows the point estimate and 95% confidence interval for the summary odds ratio and lines either side of the diamond show the predictive interval.

178x218mm (300 x 300 DPI)

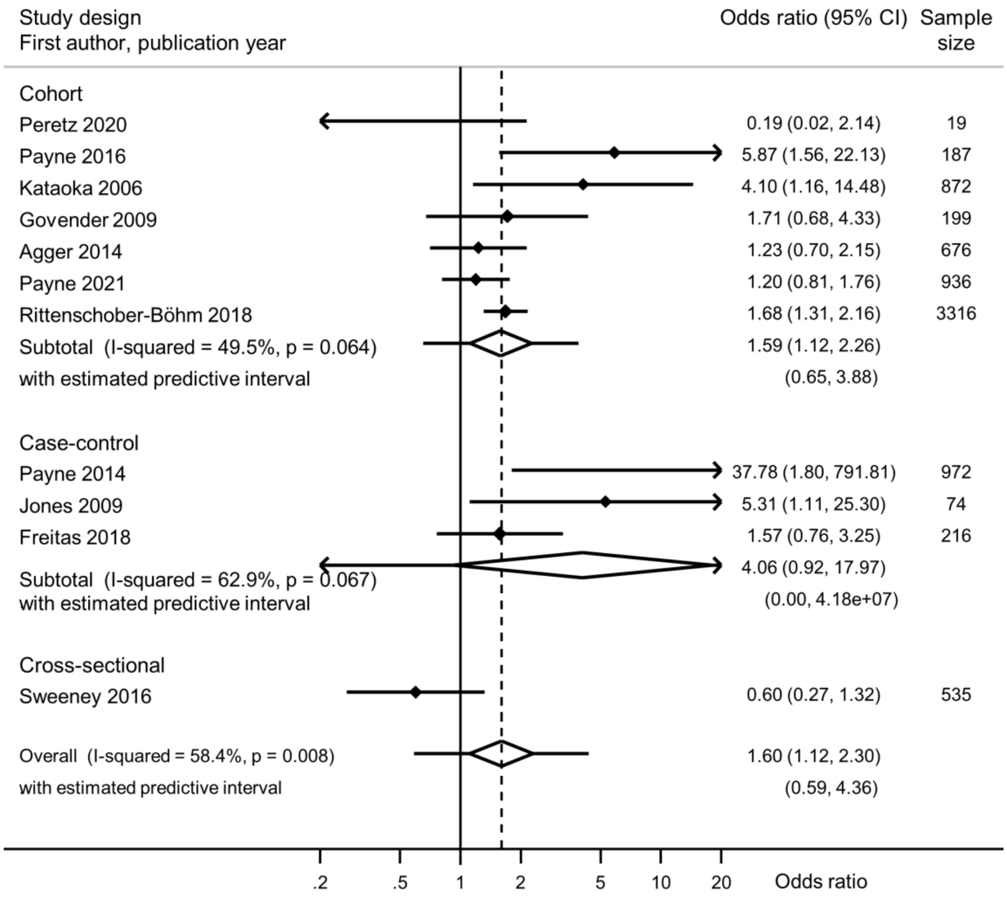

Figure 3. Forest plot of univariable association between U. parvum and preterm birth, from random effects meta-analysis. Studies are in order of precision. Solid diamonds and lines either side are point estimates and 95% confidence intervals for individual studies (arrows show where lower or upper confidence limits extend beyond the x-axis limits). Open diamond shows the point estimate and 95% confidence interval for the summary odds ratio and lines either side of the diamond show the predictive interval.

175x156mm (300 x 300 DPI)

Research checklists

A. 1 Preferred reporting items for systematic review and meta-analysis (PRISMA)

Section and Topic	Item #	Checklist item	Location where item is reported
TITLE			
Title	1	Identify the report as a systematic review.	1
ABSTRACT			
Abstract	2	See the PRISMA 2020 for Abstracts checklist.	4-5
INTRODUCTION			
Rationale	3	Describe the rationale for the review in the context of existing knowledge.	6
Objectives	4	Provide an explicit statement of the objective(s) or question(s) the review addresses.	7
METHODS			
Eligibility criteria	5	Specify the inclusion and exclusion criteria for the review and how studies were grouped for the syntheses.	7
Information sources	6	Specify all databases, registers, websites, organisations, reference lists and other sources searched or consulted to identify studies. Specify the date when each source was last searched or consulted.	8
Search strategy	7	Present the full search strategies for all databases, registers and websites, including any filters and limits used.	Appendix S1
Selection process	8	Specify the methods used to decide whether a study met the inclusion criteria of the review, including how many reviewers screened each record and each report	8

For peer review only - http://bmjopen.bmj.com/site/about/guidelines.xhtml

Research checklists

Section and Topic	Item #	Checklist item	Location where item is reported
		retrieved, whether they worked independently, and if applicable, details of automation tools used in the process.	
Data collection process	9	Specify the methods used to collect data from reports, including how many reviewers collected data from each report, whether they worked independently, any processes for obtaining or confirming data from study investigators, and if applicable, details of automation tools used in the process.	8-9
Data items	10a	List and define all outcomes for which data were sought. Specify whether all results that were compatible with each outcome domain in each study were sought (e.g. for all measures, time points, analyses), and if not, the methods used to decide which results to collect.	6
	10b	List and define all other variables for which data were sought (e.g. participant and intervention characteristics, funding sources). Describe any assumptions made about any missing or unclear information.	8-9
Study risk of bias assessment	11	Specify the methods used to assess risk of bias in the included studies, including details of the tool(s) used, how many reviewers assessed each study and whether they worked independently, and if applicable, details of automation tools used in the process.	9
Effect measures	12	Specify for each outcome the effect measure(s) (e.g. risk ratio, mean difference) used in the synthesis or presentation of results.	9
Synthesis methods	13a	Describe the processes used to decide which studies were eligible for each synthesis (e.g. tabulating the study intervention characteristics and comparing against the planned groups for each synthesis (item #5)).	9

For peer review only

Research checklists

Section and Topic	Item #	Checklist item	Location where item is reported
	13b	Describe any methods required to prepare the data for presentation or synthesis, such as handling of missing summary statistics, or data conversions.	9
	13c	Describe any methods used to tabulate or visually display results of individual studies and syntheses.	9
	13d	Describe any methods used to synthesize results and provide a rationale for the choice(s). If meta-analysis was performed, describe the model(s), method(s) to identify the presence and extent of statistical heterogeneity, and software package(s) used.	9
	13e	Describe any methods used to explore possible causes of heterogeneity among study results (e.g. subgroup analysis, meta-regression).	9
	13f	Describe any sensitivity analyses conducted to assess robustness of the synthesized results.	None
Reporting bias assessment	14	Describe any methods used to assess risk of bias due to missing results in a synthesis (arising from reporting biases).	9
Certainty assessment	15	Describe any methods used to assess certainty (or confidence) in the body of evidence for an outcome.	9
RESULTS			
Study selection	16a	Describe the results of the search and selection process, from the number of records identified in the search to the number of studies included in the review, ideally using a flow diagram.	Figure S1

For peer review only

Research checklists

Section and Topic	Item #	Checklist item	Location where item is reported
	16b	Cite studies that might appear to meet the inclusion criteria, but which were excluded, and explain why they were excluded.	Figure S1
Study characteristics	17	Cite each included study and present its characteristics.	Table S1, reference list
Risk of bias in studies	18	Present assessments of risk of bias for each included study.	11, supporting information
Results of individual studies	19	For all outcomes, present, for each study: (a) summary statistics for each group (where appropriate) and (b) an effect estimate and its precision (e.g. confidence/credible interval), ideally using structured tables or plots.	Table 1, Fig 1, Fig 2, Fig 3, supporting information
Results of syntheses	20a	For each synthesis, briefly summarise the characteristics and risk of bias among contributing studies.	11, supporting information
	20b	Present results of all statistical syntheses conducted. If meta-analysis was done, present for each the summary estimate and its precision (e.g. confidence/credible interval) and measures of statistical heterogeneity. If comparing groups, describe the direction of the effect.	11-14
	20c	Present results of all investigations of possible causes of heterogeneity among study results.	Supporting information
	20d	Present results of all sensitivity analyses conducted to assess the robustness of the synthesized results.	None
Reporting biases	21	Present assessments of risk of bias due to missing results (arising from reporting biases) for each synthesis assessed.	None

For peer review only

Research checklists

Section and Topic	Item #	Checklist item	Location where item is reported
Certainty of evidence	22	Present assessments of certainty (or confidence) in the body of evidence for each outcome assessed.	Fig 1, Fig 2, Fig 3, supporting information
DISCUSSION			
Discussion	23a	Provide a general interpretation of the results in the context of other evidence.	15
	23b	Discuss any limitations of the evidence included in the review.	16
	23c	Discuss any limitations of the review processes used.	15
	23d	Discuss implications of the results for practice, policy, and future research.	17-18
OTHER INFORMATION			
Registration and protocol	24a	Provide registration information for the review, including register name and registration number, or state that the review was not registered.	1
	24b	Indicate where the review protocol can be accessed, or state that a protocol was not prepared.	1
	24c	Describe and explain any amendments to information provided at registration or in the protocol.	None
Support	25	Describe sources of financial or non-financial support for the review, and the role of the funders or sponsors in the review.	18
Competing interests	26	Declare any competing interests of review authors.	19

For peer review only

Research checklists

Section and Topic	Item #	Checklist item	Location where item is reported
Availability of data, code and other materials	27	Report which of the following are publicly available and where they can be found: template data collection forms; data extracted from included studies; data used for all analyses; analytic code; any other materials used in the review.	Supporting information

For peer review only

For peer review only - http://bmjopen.bmj.com/site/about/guidelines.xhtml

A.2 Preferred reporting checklist of Meta-analysis (MOOSE)[1]

**Checklist item**	**Page #**
**Reporting of background should include**	
Problem definition	6
Hypothesis statement	No
Description of study outcome(s)	6
Type of exposure or intervention used	6-7
Type of study designs used	7
Study population	7
**Reporting of search strategy should include**	
Qualifications of searchers (eg. Librarians and investigators)	1
Search strategy, including time period included in the synthesis and keywords	7-8, Appendix S1
Effort to include all available studies, including contact with authors	No
Databases and registries searched	8
Search software used, name and version, including special features used (eg, explosion)	No
Use of hand searching (eg, reference lists of obtained articles	8
List of citations located and those excluded, including justification	Fig 1,<br>Table S1, excluded studies not listed

For peer review only

For peer review only - http://bmjopen.bmj.com/site/about/guidelines.xhtml

Research checklists

Method of addressing articles published in languages other than English	8
Method of handling abstracts and unpublished studies	Excluded
Description of any contact with authors	We did not contact authors
**Reporting of methods should include**	
Description of relevance or appropriateness of studies assembled for assessing the hypothesis to be tested	7-8
Rationale for the selection and coding of data (eg, multiple raters, blinding and interrater reliability)	7-8
Assessment of confounding (eg, comparability of cases and controls in studies where appropriate)	7
Assessment of study quality, including blinding of quality assessors; stratification or regression on possible predictors of study results	9
Assessment of heterogeneity	9
Description of statistical methods (eg, complete description of fixed or random effects models, justification of whether the chosen models account for predictors of study results, dose-response models, cumulative meta-analysis) in sufficient detail to be replicated	9
Provision of appropriate tables and graphs	Main text and supporting information
**Reporting of results should include**	
Graphic summarizing individual study estimates and overall estimate	Main text and forest plots in supporting information

For peer review only - http://bmjopen.bmj.com/site/about/guidelines.xhtml

Research checklists

Table giving descriptive information for each study included	Table S1
Results of sensitivity testing (eg, subgroup analysis)	None
Indication of statistical uncertainty of findings	Main text and forest plots in supporting information
**Reporting of discussion should include**	
Quantitative assessment of bias (eg, publication bias)	15-16
Justification of exclusion (eg. Exclusion of non-English-language citations)	No (provided pg 8 on methods)
Assessment of Quality of included studies	15-17
**Reporting of conclusions should include**	
Consideration of alternative explanations of observations	17-18
Generalization of conclusions (ie, appropriate for the data presented and within the domain of the literature review)	17-18
Guidelines for future research	17-18
Disclosure of funding source	18

[1] Stroup DF, Berlin JA, Morton SC, Olkin I, Williamson GD, Rennie D, et al. Meta-analysis of observational studies in epidemiology: a proposal for reporting. **Jama**. 2000 Apr 19;283(15):2008-12.

For peer review only - http://bmjopen.bmj.com/site/about/guidelines.xhtml

Supplementary Material

Adverse birth outcomes associated with *Mycoplasma hominis*, *Ureaplasma urealyticum* and *Ureaplasma parvum*: A systematic review and meta-analysis.

Marinjho Emely JONDUO (MSc),[1] Lisa Michelle VALLELY (PhD),[1,2] Handan WAND (PhD),[3] Emma Louise SWEENEY (PhD),[4] Dianne EGLI-GANY (PhD),[5] John Martin KALDOR (PhD),[1] Andrew John VALLELY (PhD),[1,2] Nicola LOW (PhD).[5]

[1] Public Health Interventions Research Program, The Kirby Institute, University of New South Wales, Sydney, NSW 2052, Australia.

[2] Sexual and Reproductive Health Unit, Papua New Guinea Institute of Medical Research, Goroka, Eastern Highlands Province 441, Papua New Guinea.

[3] Biostatistics and Databases program, The Kirby Institute, University of New South Wales, Sydney, New South Wales, Australia.

[4] The University of Queensland Center for Clinical Research, Brisbane, Queensland 4029, Australia.

[5] Institute of Social and Preventive Medicine, University of Bern, CH-3012 Bern, Switzerland.

For peer review only

Table of Contents

Table of Contents 2

Text S1 Search strategy 3

Figure S1 Flow chart of identified and selected studies for inclusion 4

Table S1 Summary of characteristics of included studies 5

Figure S3.1 Forest plot of association between *M. hominis* and premature rupture of membrane, random effects model. 23

Figure S3.2 Forest plot of association between *M. hominis* and low birthweight, random effects model. 24

Figure S3.3 Forest plot of association between *M. hominis* and perinatal death random effects model. 25

Figure S3.4 Forest plot of association between *M. hominis* and spontaneous abortion random effects model. 26

Figure S4.1 Forest plot of association between *U. urealyticum* and premature rupture of membrane, random effects model. 27

Figure S4.2 Forest plot of association between *U. urealyticum* and low birth weight, random effects model. 28

Figure S4.3 Forest plot of association between *U. urealyticum* and spontaneous abortion, random effects model 29

Figure S5.1 Forest plot of association between *U. parvum* and preterm birth, random effects model. 31

Figure S5.2 Forest plot of association between *U. parvum* and premature rupture of membrane, random effects model. 32

Table S3.1 Descriptive tables: Cohort studies (n=26) 34

Table S3.2 Descriptive tables: Case control studies (n=25) 40

Table S4.1 Summary description of studies reporting *M. hominis* (n=42), by income status 46

Table S4.2 Summary description of studies reporting *U. urealyticum* (n=31), by income status 55

Table S4.3 Summary description of studies reporting *U. parvum* (n=12), by income status 62

Table S5.1 Study setting and socio-demographics, cohort studies (n=26) 65

Table S5.3 Study setting and socio-demographics, cross-sectional studies (n=6) 71

Table S6 Studies that reported on bacterial vaginosis or sexually transmitted infections and reported associations with adverse birth outcomes 72

Table S7.1 Risk of bias assessment, cohort studies (n=26) 78

Table S7.2 Risk of bias assessment, case-control studies (n=25) 93

Table S7.3 Risk of bias assessment, cross-sectional studies (n=6) 101

For peer review only - http://bmjopen.bmj.com/site/about/guidelines.xhtml

Text S1 Search strategy

1. Terms for population	"pregnancy" or "prenatal" or "antenatal"
2. Terms for exposure	"Mycoplasma hominis" or "M. hominis"; "Ureaplasma urealyticum" or "U. urealyticum"; "Ureaplasma parvum" or "U. parvum"
3. Terms for outcomes	"birth outcome" or "adverse birth outcome" or "adverse pregnancy outcome" or "perinatal morbidity" or "perinatal mortality" or "perinatal outcome" or "premature birth" or "premature delivery" or "very preterm birth" or "preterm birth" or "preterm delivery" or "premature labour" or "preterm labour" or "premature labor" or "preterm labor" or "premature rupture of membranes" or "preterm rupture of membranes" or "preterm premature rupture of membranes" or "low birth weight" or "intrauterine growth retardation" or "intrauterine growth restriction" or "small for gestational age" or "gestational age" or "stillbirth" or "perinatal mortality" or "perinatal morbidity" or "perinatal death" or "neonatal mortality" or "neonatal morbidity" or "neonatal death" or "fetal death" or "miscarriage" or "spontaneous abortion" or "chorioamnionitis"
4. Search = #1 + # 2 + # 3	

Free text terms in the search strategy will use truncated and wildcard forms e.g., pregn*

Explode function used for MeSH headings

Reference lists of retrieved articles searched

For peer review only

Figure S1 Flow chart of identified and selected studies for inclusion

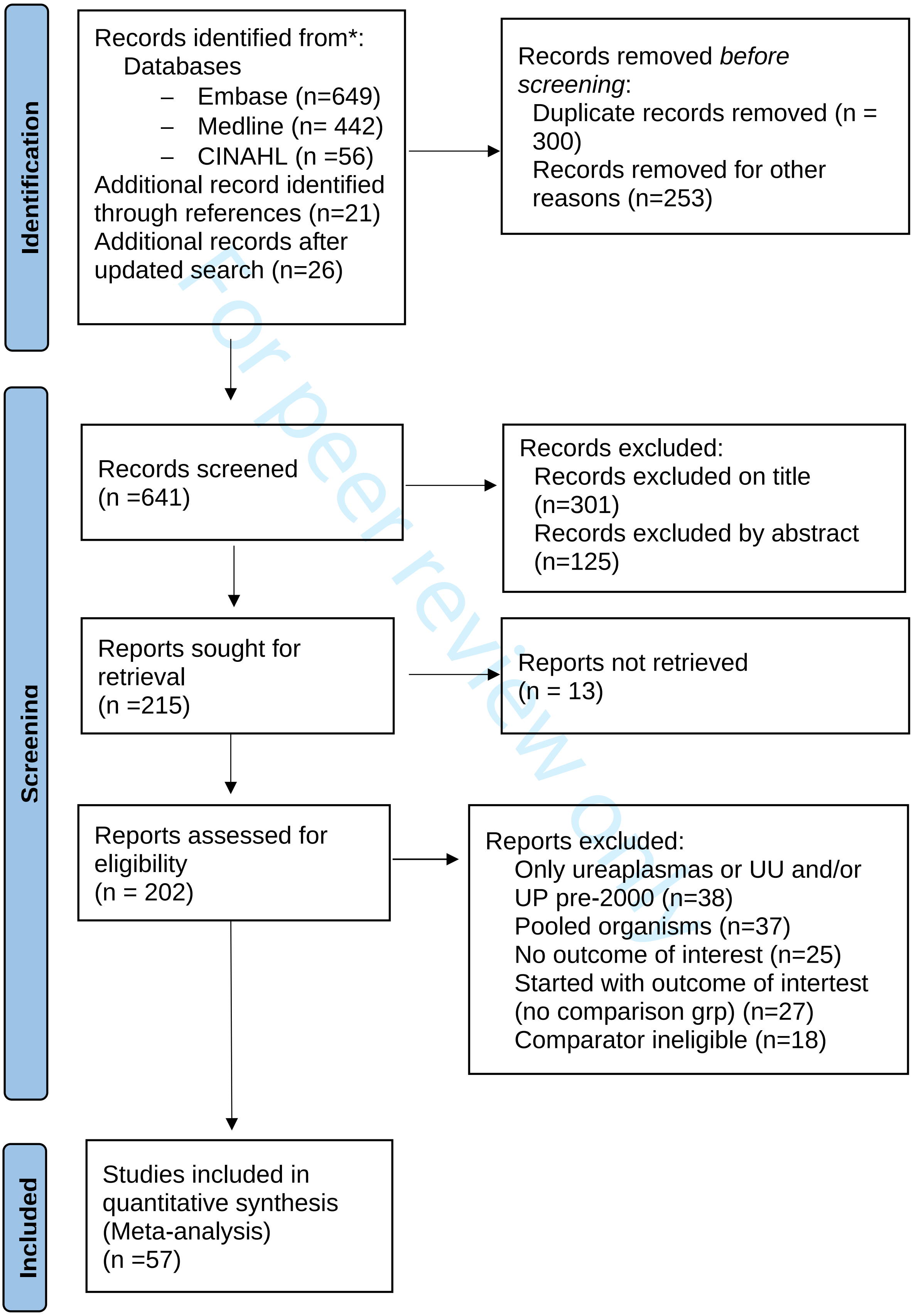

For peer review only

Supporting information

Table S1 Summary of characteristics of included studies

First author, publication year, study reference*†	Country	Study design	Study population	Total no. of women	Outcomes measured	Specimen type; collection time	Diagnostic method	BV assessed
**Abele-Horn, 2000**[73]	Germany	Cross-sectional	Admitted for delivery, Jan - Dec 1996	295	PTB, PROM	Endocervical swab; 1st & 2nd trimester	Culture	Excluded
**Agger, 2014**[1]	USA	Cohort	10 to 14 weeks gestation, initial prenatal visit; currently uncomplicated pregnancy	783	PTB	Endocervical swab; 1st, 2nd trimester	NAAT	NR
**Ahmadi, 2014**[9]	Iran	Case-control	10-20 weeks (cases); normal pregnancy 20-30 weeks (control)	218	SA	Endocervical swab; 1st, 2nd, 3rd trimester	NAAT	NR
**Berman, 1987**[32]	Mexico	Cohort	Women at their prenatal care visit, single centre; Oct 1980 - Oct 1983	1204	LBW	Endocervical swab; 1st, 2nd, 3rd trimester	Culture	NR

For peer review only - http://bmjopen.bmj.com/site/about/guidelines.xhtml

Supporting information

First author, publication year, study reference*†	Country	Study design	Study population	Total no. of women	Outcomes measured	Specimen type; collection time	Diagnostic method	BV assessed
**Braun, 1971[33]**	USA	Cohort	Entering antenatal clinic, single centre; Feb-Jul 1969	688	LBW	Endocervical swab, urine; 1st & 3rd trimester	Culture	NR
**Cassell, 1983[53]**	USA	Case-control	Attending the amniocentesis for prenatal diagnosis, single centre	61	PTB, PND	Amniotic fluid; 2nd trimester	Culture	NR
**Chua, 1999[68]**	Malaysia	Case-control	60 sequential mother who delivered and premature babies, single centre, Jan 1996- June 1997	120	PTB	Endocervical swab; 2nd, 3rd trimester	Culture	NR
**Daskalakis, 2009[30]**	NR	Case-control	Singleton, normal pregnancy, >18 years old, mid-trimester	613	PTB	Amniotic fluid; 2nd trimester	Culture	NR

For peer review only - http://bmjopen.bmj.com/site/about/guidelines.xhtml

Supporting information

First author, publication year, study reference*†	Country	Study design	Study population	Total no. of women	Outcomes measured	Specimen type; collection time	Diagnostic method	BV assessed
			amniocentesis, Feb 2006 - Sept 2007					
**Donders, 2009[34]**	Belgium	Cohort	Singleton, first antenatal visit between 9 -16 weeks with complete data available on *M. hominis* cultures; June 2000 – Dec 2001	759	PTB, SA	Vaginal swab; 1st & 2nd trimester	Culture	Yes
**Embree, 1980[54]**	Canada	Case-control	Single centre, deliveries between May 1977 and Jan 1978	554	SA, PND	Placenta; Post-partum	Culture	NR
**Farhadifar, 2016[11]**	Iran	Case-control	Admitted in obstetrics and gynaecology wards; no	218	SA	Endocervical swab; 1st, 2nd, 3rd trimester	NAAT	NR

For peer review only - http://bmjopen.bmj.com/site/about/guidelines.xhtml

Supporting information

First author, publication year, study reference*†	Country	Study design	Study population	Total no. of women	Outcomes measured	Specimen type; collection time	Diagnostic method	BV assessed
			antibiotics two weeks before sampling, no chronic disease, vaginal infection; Aug 2012 – Jan 2013					
**Freitas, 2018[55]**	Canada	Case-control	Spontaneous preterm birth and term deliveries, multicentre	216	PTB	Vaginal swab; 2nd trimester	NAAT	NR
**Gerber, 2003[80]**	NR	Cohort	Transabdominal amniocentesis at 15-17 weeks GA; singleton without complicated pregnancy	254	PTB, PROM	Amniotic fluid; 2nd trimester	NAAT	NR
**Gonzàlez Bosquet, 2006[67]**	NR	Case-control	Case: 24-34 weeks PTL, intact membranes; control: no history	250	PTB	Endocervical swab; NR	Culture	Yes

For peer review only - http://bmjopen.bmj.com/site/about/guidelines.xhtml

Supporting information

First author, publication year, study reference*†	Country	Study design	Study population	Total no. of women	Outcomes measured	Specimen type; collection time	Diagnostic method	BV assessed
			of preterm birth at same stage of delivery					
**Govender, 2009[79]**	South Africa	Cohort	Low risk obstetric patients at first prenatal visit (16-23 weeks gestation)	199	PTB	Endocervical swab; 2nd trimester	NAAT	NR
**Grattard, 1995[74]**	France	Cross-sectional	Women who delivered between Feb - May 1993 in obstetrical ward and their neonates, single centre	208	PTB, LBW PROM,	Endocervical swab; post-partum	Culture	NR
**Harada, 2008[56]**	Japan	Case-control	Premature and term deliveries, Jan 2006 - July 2007	145	PTB	Endocervical swab; 2nd, 3rd trimester	NAAT, Culture	NR

For peer review only - http://bmjopen.bmj.com/site/about/guidelines.xhtml

Supporting information

First author, publication year, study reference*†	Country	Study design	Study population	Total no. of women	Outcomes measured	Specimen type; collection time	Diagnostic method	BV assessed
**Harrison, 1983**[37]	USA	Cohort	Enrolled at their first prenatal visit, single centre	860	SA	Endocervical swab; 1st, 2nd, 3rd trimester	Culture, ELISA	NR
**Hillier, 1988**[57]	USA	Case-control	Age >16 years; no antibiotics in previous 2 weeks; no known fetal anomaly; June 1984- June 1985	112	PTB	Placenta; Post-partum	Culture	Yes
**Hillier, 1995**[31]	USA	Cohort	> 16 years, singleton pregnancies at routine prenatal visits (23 to 26 weeks), between 1984-1989,	10,397	PTB	Endocervical swab; 2nd trimester	Culture	Yes

For peer review only - http://bmjopen.bmj.com/site/about/guidelines.xhtml

Supporting information

First author, publication year, study reference*†	Country	Study design	Study population	Total no. of women	Outcomes measured	Specimen type; collection time	Diagnostic method	BV assessed
**Holst, 1994[58]**	Sweden	Case-control	Women presenting in PTL; controls were women with no pregnancy history	87	PTB	Endocervical swab; 3rd trimester	Culture	yes
**Jalava, 2002[69]**	NR	Case-control	Control: 3rd trimester, no signs labour. Cases: contractions as sign of premature labour 22-35/40	122	PTB	Endocervical swab; 2nd, 3rd trimester	NAAT	NR
**Jones, 2009[10]**	United Kingdom	Case-control	Single centre, cases: <32 weeks gestation; Control >37 weeks; single centre	74	PTB, PROM	Placenta; Post-partum	NAAT	NR
**Kacerovsky, 2009[70]**	NR	Case-control	Pregnancy with PPROM, single centre, Jan 2004 - Feb 2007.	450	PROM	Endocervical swab; 2nd, 3rd trimester	Culture	NR

For peer review only - http://bmjopen.bmj.com/site/about/guidelines.xhtml

Supporting information

First author, publication year, study reference*†	Country	Study design	Study population	Total no. of women	Outcomes measured	Specimen type; collection time	Diagnostic method	BV assessed
**Kafetzis, 2004[59]**	Greece	Case-control	Case: premature delivery; control: term delivery from June 2000 to Dec 2001	251	PTB, PROM, PND	Vaginal swab; 3rd trimester	Culture	NR
**Kataoka, 2006[38]**	Japan	Cohort	Singleton pregnancies at <11 weeks of gestation, single centre, Jan – Dec 2002	1040	PTB, PROM, SA, PND	Vaginal swab;1st trimester	NAAT	NR
**Koucky, 2016[39]**	Czech Republic	Cohort	Threatened premature deliveries, between Aug 2012 - Feb 2013	63	PTB	Vaginal swab; 2nd, 3rd trimester	NAAT	NR
**Kumar, 2006[71]**	India	Case-control	Women in spontaneous premature/term labour with or	120	PTB	Vaginal swab; 3rd trimester	Culture	Yes

For peer review only - http://bmjopen.bmj.com/site/about/guidelines.xhtml

Supporting information

First author, publication year, study reference*†	Country	Study design	Study population	Total no. of women	Outcomes measured	Specimen type; collection time	Diagnostic method	BV assessed
			without rupture of membrane, single centre					
**Kundsin, 1984[75]**	USA	Cross-sectional	Deliveries at single centre, between Nov 1978 - Jun 1981	801	PND	Placenta; Post-partum	Culture	NR
**Kwak, 2014[49]**	South Korea	Cohort	Women with spontaneous premature labour or preterm PROM, Dec 2005 – Apr 2007, single centre	179	PTB	Vaginal swab; 3rd trimester	Culture	NR
**Lee, 2016[6]**	South Korea	Cohort	Aged 15-47, delivered babies at single centre between Jun 2009 - May 2014	1,035	PTB, PROM, SA	Vaginal swab; NR	Culture	NR

For peer review only

Supporting information

First author, publication year, study reference*†	Country	Study design	Study population	Total no. of women	Outcomes measured	Specimen type; collection time	Diagnostic method	BV assessed
**Luton, 1994**[8]	Gabon	Cohort	Singleton pregnancy at <20 weeks gestation, Sept 1990 to Nov 1991	218	PTB, LBW, PND	Endocervical swab; 1st, 2nd trimester	Culture	NR
**McCormack, 1975**[76]	USA	Cross-sectional	Vaginal deliveries, single centre,	327	LBW, PND	Blood; post-partum	Culture	NR
**McDonald, 1992**[60]	Australia	Case-control	Women who booked at one of 4 study centres, Oct 1986 – Dec 1988	2190	PTB, PROM	Endocervical swab; 2nd trimester	Culture	NR
**McDonald, 1994**[40]	Australia	Cohort	Patients attending the antenatal clinic, Oct 1986 - May 1990	560	PTB	Endocervical swab; 2nd, 3rd trimester	Culture	NR

For peer review only - http://bmjopen.bmj.com/site/about/guidelines.xhtml

Supporting information

First author, publication year, study reference*†	Country	Study design	Study population	Total no. of women	Outcomes measured	Specimen type; collection time	Diagnostic method	BV assessed
**Menard, 2010[41]**	France	Cohort	Admitted preterm labour with no pregnancy related complications from July 2007 - July 2008	90	PTB	Vaginal swab; 2nd, 3rd trimester	NAAT	Yes
**Minkoff, 1984[42]**	USA	Cohort	Attending single centre, for delivery between Mar - Sept 1982	250	PTB, PROM	Vaginal swab; 1st, 2nd trimester	Culture	NR
**Mitsunari, 2005[61]**	Japan	Case-control	Singleton pregnancy delivery, between Jan 2002 - Sept 2003	82	PTB, PROM	Endocervical swab; 2nd, 3rd trimester	NAAT	NR
**Montenegro, 2019[72]**	Colombia	Case-control	Pregnant women >18 years, no pregnancy related problems,	211	PTB, PROM	Placenta; Post-partum	NAAT	NR

For peer review only - http://bmjopen.bmj.com/site/about/guidelines.xhtml

Supporting information

First author, publication year, study reference*†	Country	Study design	Study population	Total no. of women	Outcomes measured	Specimen type; collection time	Diagnostic method	BV assessed
			non-smokers, no alcohol, no antibiotic					
**Munday, 1984[62]**	United Kingdom	Case-control	Women admitted with vaginal bleeding before 28 weeks gestation and women attending one antenatal clinic at same hospital	241	SA	Endocervical swab; 2nd, 3rd trimester	Culture	NR
**Nasution, 2007[78]**	NR	Cross-sectional	Women admitted with preterm PROM (<37weeks), normal vaginal deliveries at term, and women with post-partum fever	120	PROM	Placenta; Post-partum	NAAT	NR

For peer review only - http://bmjopen.bmj.com/site/about/guidelines.xhtml

Supporting information

First author, publication year, study reference*†	Country	Study design	Study population	Total no. of women	Outcomes measured	Specimen type; collection time	Diagnostic method	BV assessed
**Nguyen, 2004[43]**	Switzerland	Cohort	Women with transabdominal amniocentesis at 15-17 weeks gestation, single centre	456	PTB, PROM, PND	Amniotic fluid; 2nd trimester	NAAT	NR
**Odendaal, 2002[50]**	South Africa	Cohort	Primigravid, first visit, 16-26 weeks with previous preterm labour or miscarriage, May-Dec 1996	395	PTB, SA, PND	Endocervical swab; 2nd trimester	Culture	Yes
**Oliveira, 2020[7]**	Brazil	Case-control	> 18 years old, cases: 8 -20 weeks gestation; Control vaginal delivery at 38-40 weeks, Jul 2017 – Aug 2018,	109	SA	Endocervical swab; 1st, 2nd, 3rd trimester	NAAT	NR

For peer review only

Supporting information

First author, publication year, study reference*†	Country	Study design	Study population	Total no. of women	Outcomes measured	Specimen type; collection time	Diagnostic method	BV assessed
**Payne, 2014**[63]	China and Australia	Case-control	Singleton pregnancy referred for genetic amniocentesis	972	PTB	Amniotic fluid; 2nd trimester	NAAT	NR
**Payne, 2016**[44]	Australia	Cohort	Low risk singleton pregnancy, 18-40 years old, at 1st or 2nd trimester when enrolled	191	PTB	Vaginal swab; 1st, 2nd, 3rd trimester	NAAT, Culture	NR
**Payne, 2021**[45]	Australia	Cohort	Nulliparous and multiparous, singleton pregnancy, ≥16 years between 12 - 23 weeks gestation	1000	PTB	Vaginal swab; 1st, 2nd trimester	NAAT	NR
**Peretz, 2020**[12]	Israel	Cohort	Women, 18-45 years, at any stage of labor and any mode of delivery, between Jun 2014 and Jan 2016.	214	PTB, LBW	Vaginal swab; post-partum	NAAT	NR

For peer review only - http://bmjopen.bmj.com/site/about/guidelines.xhtml

Supporting information

First author, publication year, study reference*†	Country	Study design	Study population	Total no. of women	Outcomes measured	Specimen type; collection time	Diagnostic method	BV assessed
**Perni, 2004[51]**	Unknown	Cohort	Singleton pregnancy: underwent transabdominal amniocentesis at 15-19 weeks with clear amniotic fluid	193	PTB, PROM	Amniotic fluid; 2nd trimester	NAAT	NR
**Povlsen, 2001[64]**	Denmark	Case-control	Singleton, single centre; attending first antenatal visit between Nov 1992 - Feb 1994	484	PTB, LBW	Endocervical swab; 1st, 2nd trimester	NAAT	Yes
**Rittenschober-Böhm, 2018[46]**	Austria	Cohort	Attending routine nuchal translucency screening between 12-14 weeks gestation, multicentre study	4330	PTB	Endocervical swab; 1st, 2nd trimester	NAAT	Yes

For peer review only - http://bmjopen.bmj.com/site/about/guidelines.xhtml

Supporting information

First author, publication year, study reference*†	Country	Study design	Study population	Total no. of women	Outcomes measured	Specimen type; collection time	Diagnostic method	BV assessed
**Schwab, 2015[52]**	Indonesia	Cohort	2nd trimester, four centres, from Feb -Jun 2005	159	PTB	Vaginal swab; 2nd trimester	NAAT	Yes
**Sperling, 1988[48]**	USA	Cohort	Clinical diagnosis of intraamniotic infection, July 1979 – Dec 1986	409	LBW	Amniotic fluid; NR	Culture	NR
**Sweeney, 2016[77]**	USA	Cross-sectional	Term deliveries, no HIV infection, congenital infection, or fetal malformation, Jul 2010–Apr 2013	535	PTB	Placenta; Post-partum	NAAT, Culture	NR
**Toth, 1992[65]**	UK	Case-control	Admitted for delivery between Jan 1985 - Dec 1986	100	PTB	Endocervical swab; NR	Culture	NR

For peer review only

Supporting information

First author, publication year, study reference*†	Country	Study design	Study population	Total no. of women	Outcomes measured	Specimen type; collection time	Diagnostic method	BV assessed
**Usui, 2002**[47]	Japan	Cohort	Singleton pregnancy attending first antenatal visit, Jan 1995 – Mar 1998	1958	PTB	Endocervical swab; 1st, 3rd trimester	Culture	NR
**Yoon, 2001**[66]	South Korea	Case-control	Women who underwent mid-trimester amniocentesis	114	PTB, PROM	Amniotic fluid; 2nd trimester	NAAT	NR

Abbreviations: LBW, infant low birth weight, defined as birth weight <2.5kg; NAAT, nucleic acid amplification tests; NR, not reported; PTB, preterm birth - defined as birth before 37 weeks gestation; PROM: premature rupture of membrane- defined as clinically confirmed rupture of membrane before 37weeks of gestation; PND, perinatal death- defined as stillbirth (death after 20/40 gestation) or neonatal death (death <28 day since birth), unless otherwise defined by the study authors; SA: spontaneous abortion - defined as pregnancy loss at <20 weeks gestation or as defined by author

Diagnostic method: a) Mycofast All-in test; b) A7/ A8 culture media; c) Mycoplasma IST-2 kit

USA, United States of America; UK, United Kingdom.

* Study reference is the reference number cited in the main manuscript

† Additional summary information about the characteristics of included studies in Tables S3.1-S3.3, S4.1-S4.3, S5.1-S5.3

For peer review only - http://bmjopen.bmj.com/site/about/guidelines.xhtml

Supporting information

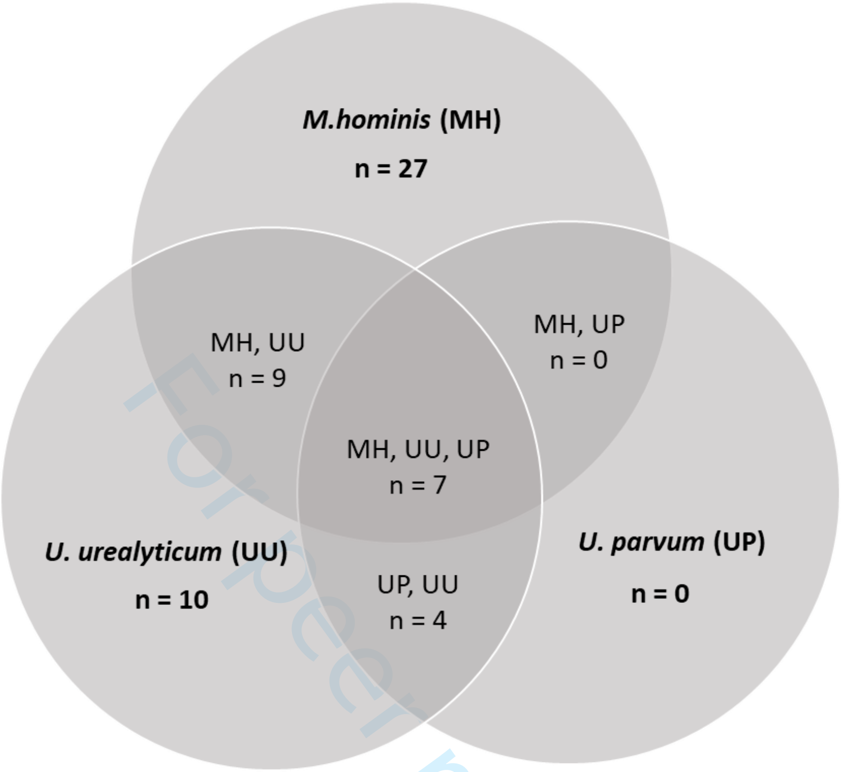

Figure S2 Venn diagram showing organisms reported in in the 57 articles included in the systematic review.

For peer review only - http://bmjopen.bmj.com/site/about/guidelines.xhtml

Supporting information

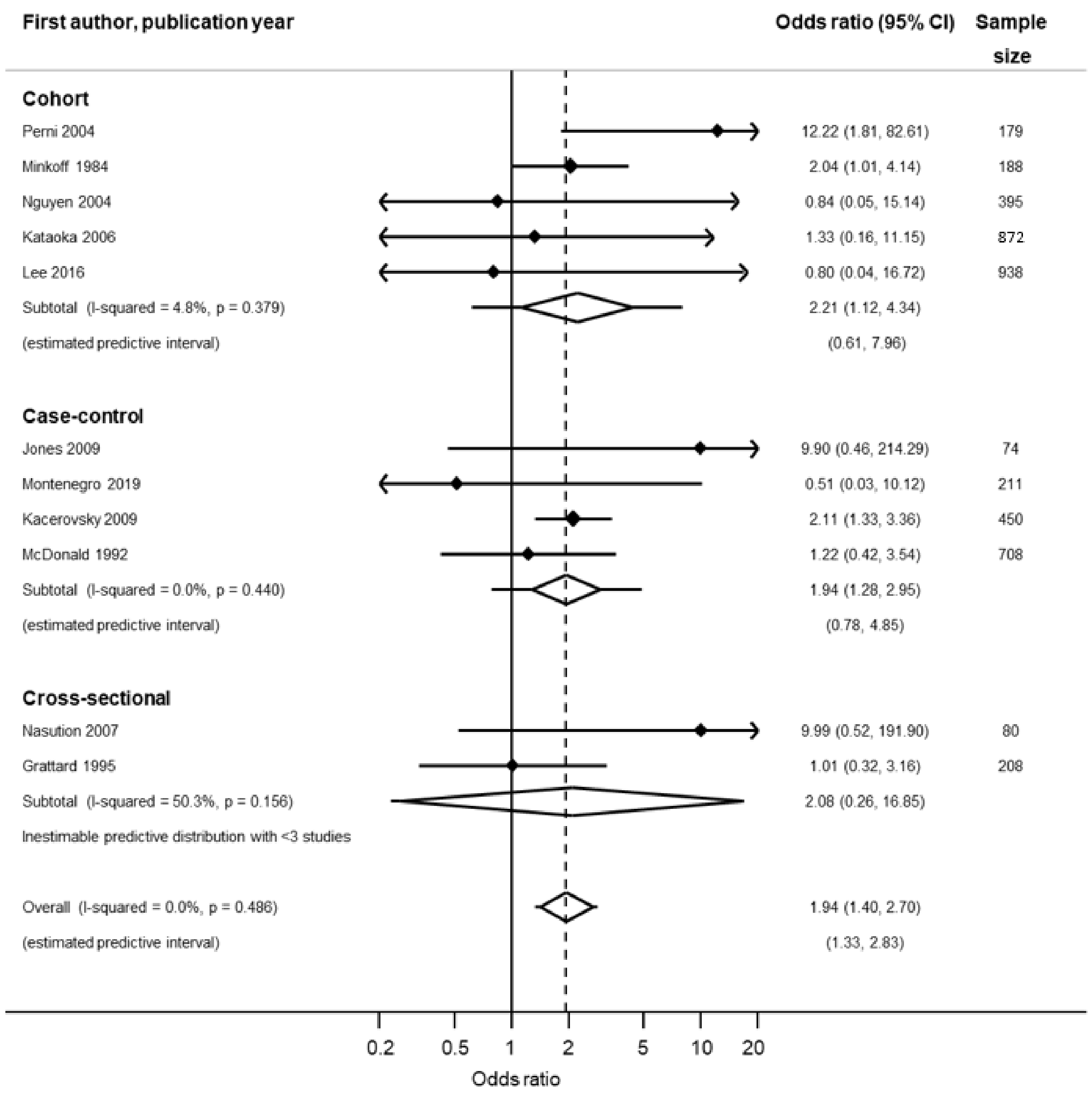

Figure S3.1 Forest plot of association between *M. hominis* and premature rupture of membrane, random effects model.

Solid diamond shows the point estimate and study weight. Solid line shows 95% confidence intervals (CI) for each study. The open diamond shows the point estimate and 95% CI of the summary estimate. Solid lines extending from the diamond show the prediction interval.

For peer review only - http://bmjopen.bmj.com/site/about/guidelines.xhtml

Supporting information

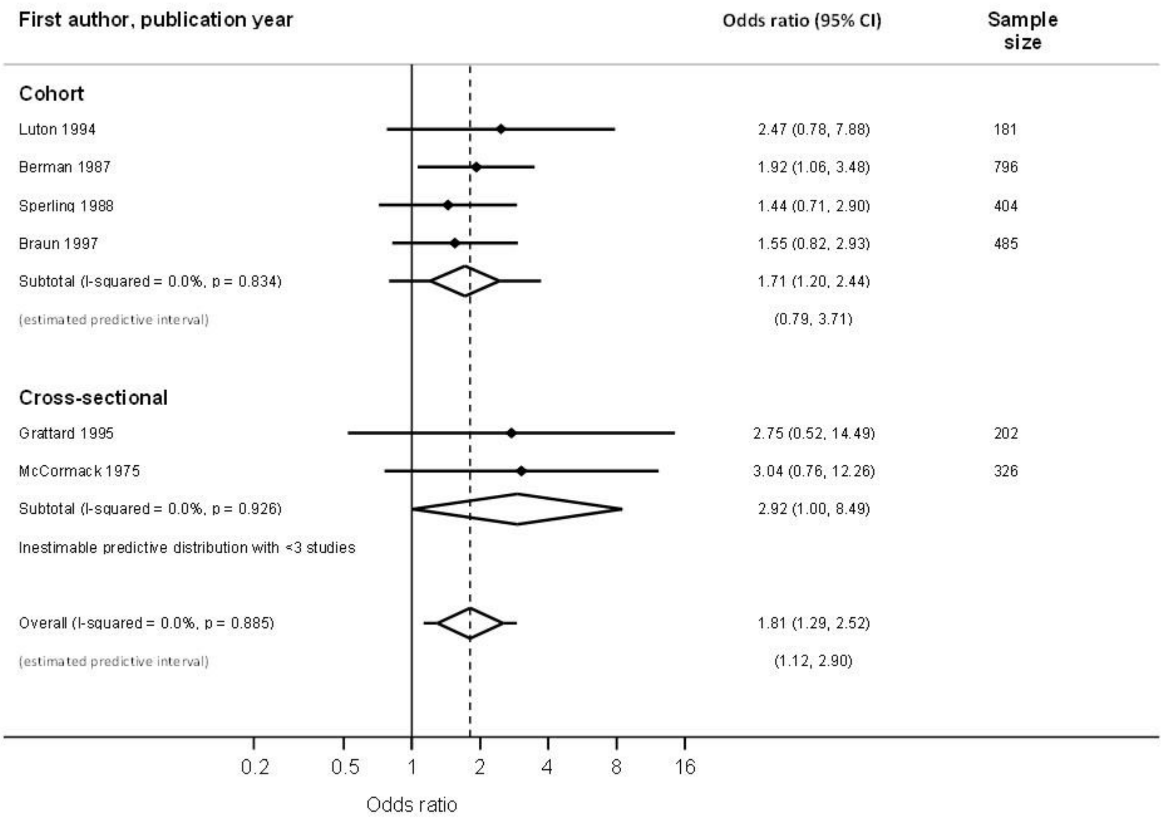

Figure S3.2 Forest plot of association between *M. hominis* and low birthweight, random effects model.

Solid diamond shows the point estimate and study weight. Solid line shows 95% confidence intervals (CI) for each study. The open diamond shows the point estimate and 95% CI of the summary estimate. Solid lines extending from the diamond show the prediction interval.

For peer review only - http://bmjopen.bmj.com/site/about/guidelines.xhtml

Supporting information

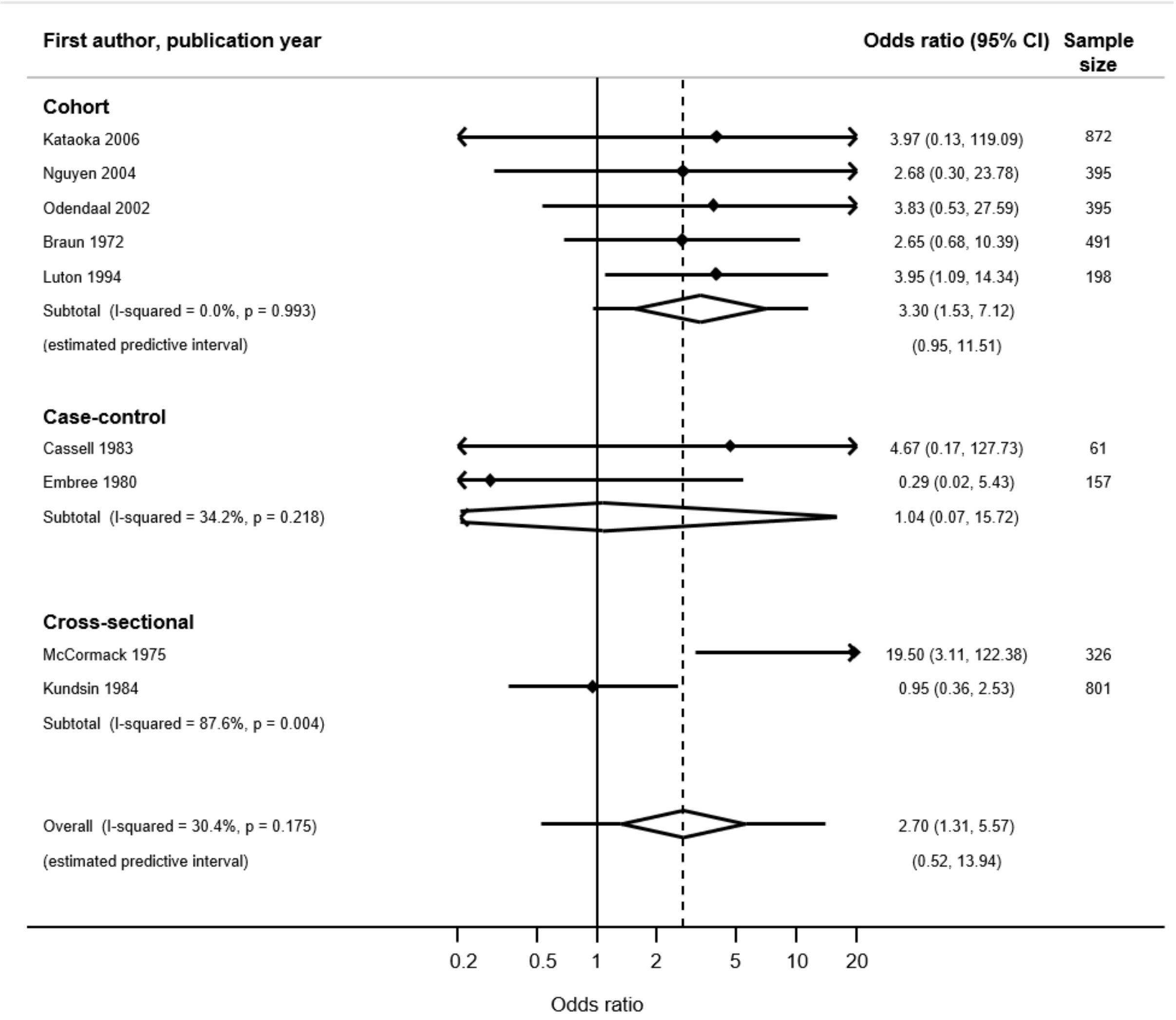

Figure S3.3 Forest plot of association between *M. hominis* and perinatal death random effects model.

Solid diamond shows the point estimate and study weight. Solid line shows 95% confidence intervals (CI) for each study. The open diamond shows the point estimate and 95% CI of the summary estimate. Solid lines extending from the diamond show the prediction interval.

For peer review only - http://bmjopen.bmj.com/site/about/guidelines.xhtml

Supporting information

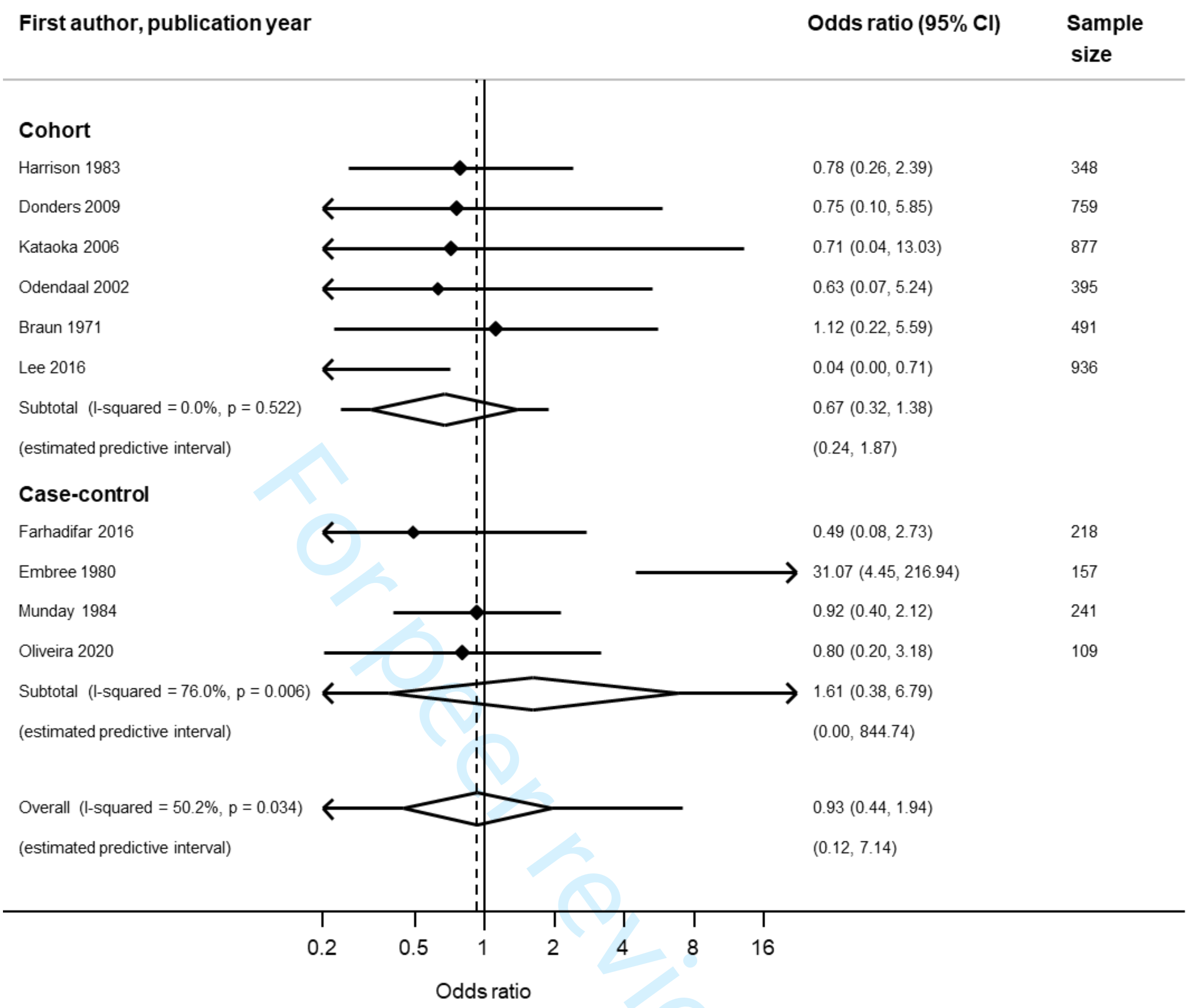

Figure S3.4 Forest plot of association between *M. hominis* and spontaneous abortion random effects model.

Solid diamond shows the point estimate and study weight. Solid line shows 95% confidence intervals (CI) for each study. The open diamond shows the point estimate and 95% CI of the summary estimate. Solid lines extending from the diamond show the prediction interval.

For peer review only

Supporting information

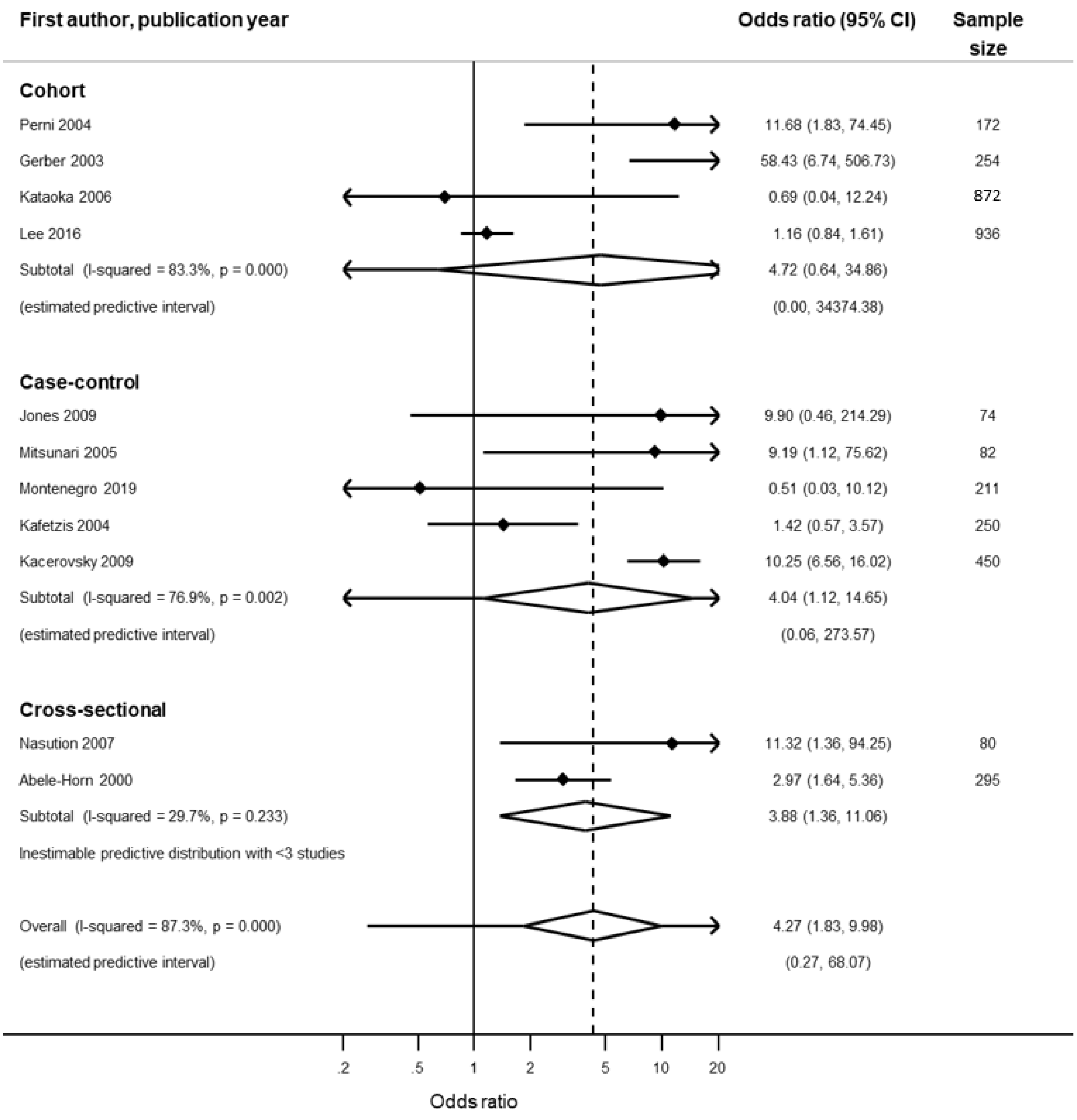

Figure S4.1 Forest plot of association between *U. urealyticum* and premature rupture of membrane, random effects model.

Solid diamond shows the point estimate and study weight. Solid line shows 95% confidence intervals (CI) for each study. The open diamond shows the point estimate and 95% CI of the summary estimate. Solid lines extending from the diamond show the prediction interval.

For peer review only - http://bmjopen.bmj.com/site/about/guidelines.xhtml

Supporting information

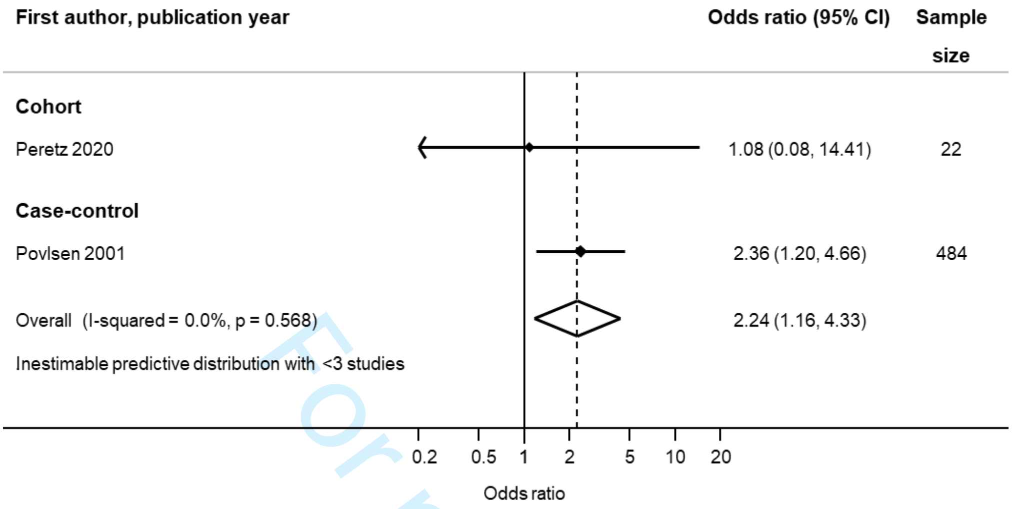

Figure S4.2 Forest plot of association between *U. urealyticum* and low birth weight, random effects model.

Solid diamond shows the point estimate and study weight. Solid line shows 95% confidence intervals (CI) for each study. The open diamond shows the point estimate and 95% CI of the summary estimate. Solid lines extending from the diamond show the prediction interval.

For peer review only

Supporting information

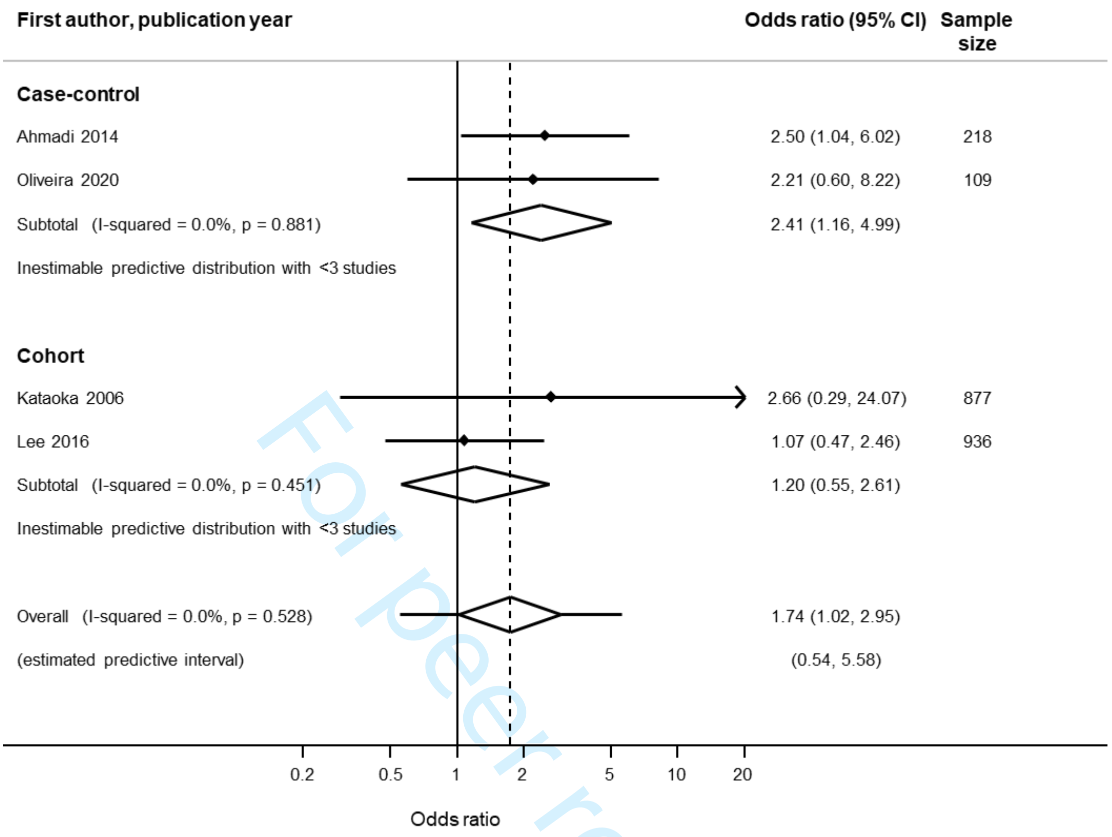

Figure S4.3 Forest plot of association between *U. urealyticum* and spontaneous abortion, random effects model

Solid diamond shows the point estimate and study weight. Solid line shows 95% confidence intervals (CI) for each study. The open diamond shows the point estimate and 95% CI of the summary estimate. Solid lines extending from the diamond show the prediction interval.

For peer review only

Supporting information

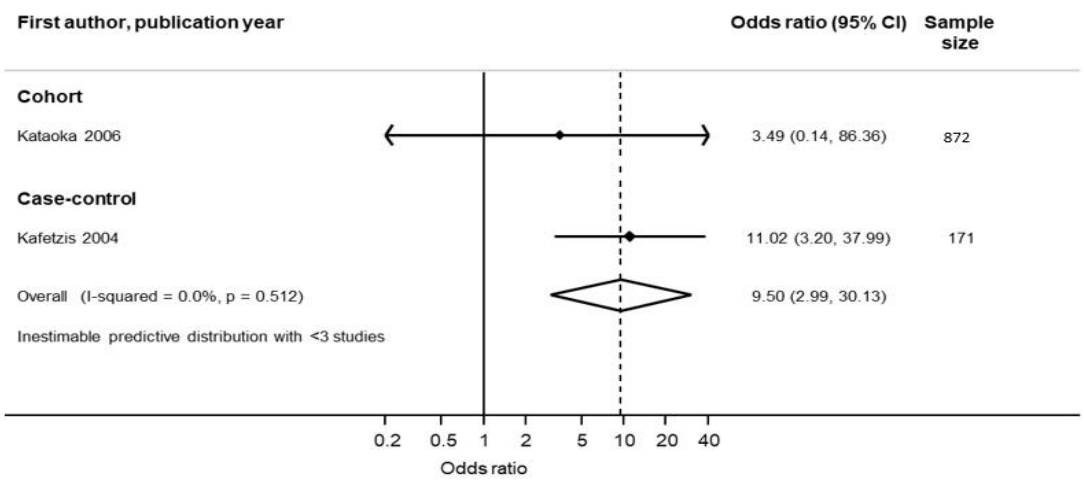

Figure S4.4 Forest plot of association between *U. urealyticum* and perinatal death, random effects model**.** Solid diamond shows the point estimate and study weight. Solid line shows 95% confidence intervals (CI) for each study. The open diamond shows the point estimate and 95% CI of the summary estimate. Solid lines extending from the diamond show the prediction interval.

For peer review only

Supporting information

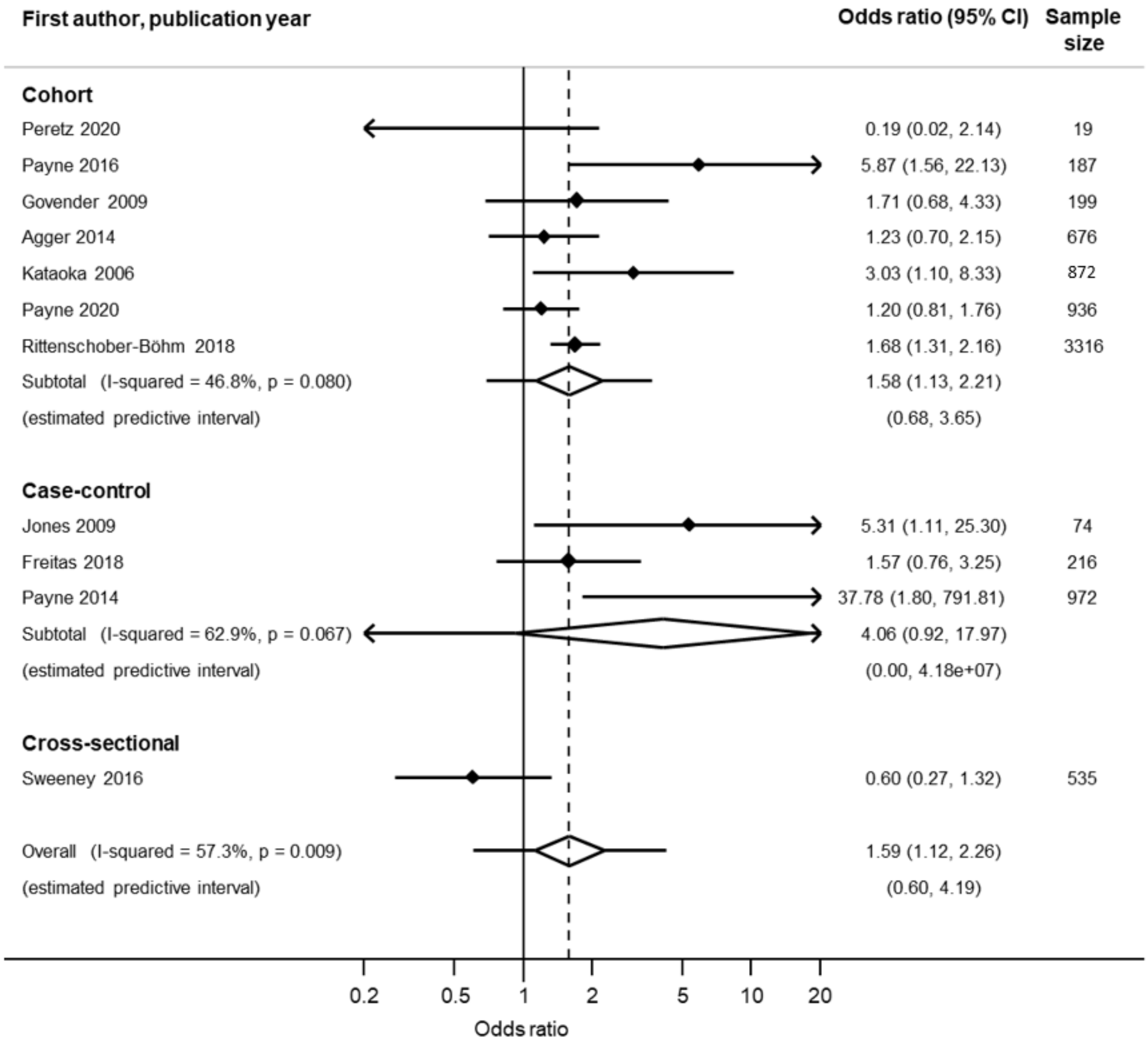

Figure S5.1 Forest plot of association between *U. parvum* and preterm birth, random effects model.

Solid diamond shows the point estimate and study weight. Solid line shows 95% confidence intervals (CI) for each study. The open diamond shows the point estimate and 95% CI of the summary estimate. Solid lines extending from the diamond show the prediction interval.

For peer review only - http://bmjopen.bmj.com/site/about/guidelines.xhtml

Supporting information

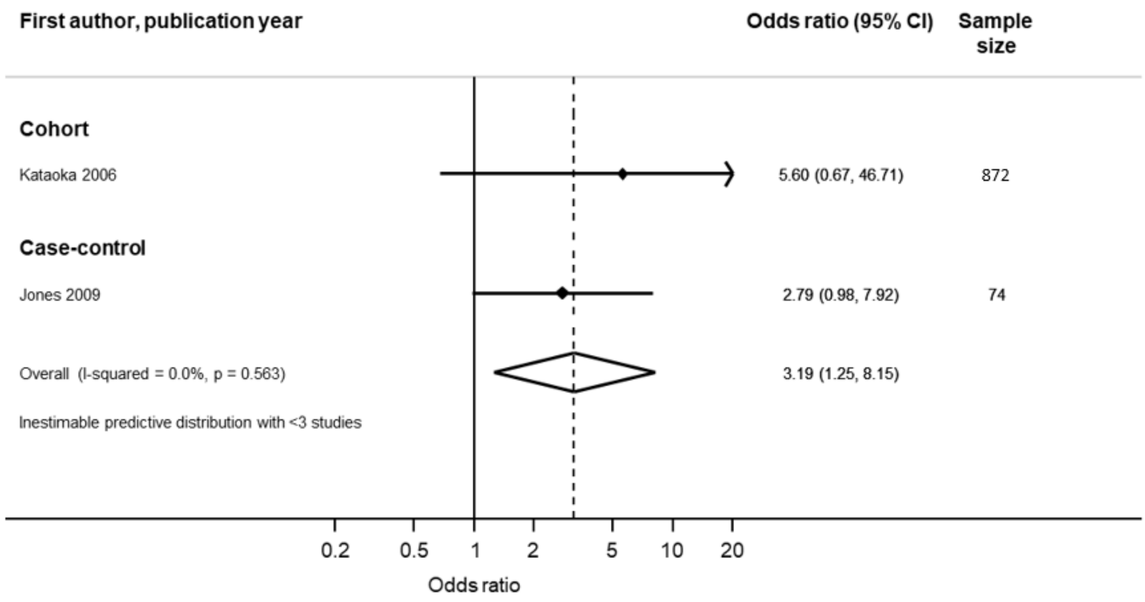

Figure S5.2 Forest plot of association between *U. parvum* and premature rupture of membrane, random effects model.

Solid diamond shows the point estimate and study weight. Solid line shows 95% confidence intervals (CI) for each study. The open diamond shows the point estimate and 95% CI of the summary estimate. Solid lines extending from the diamond show the prediction interval.

For peer review only - http://bmjopen.bmj.com/site/about/guidelines.xhtml

Supporting information

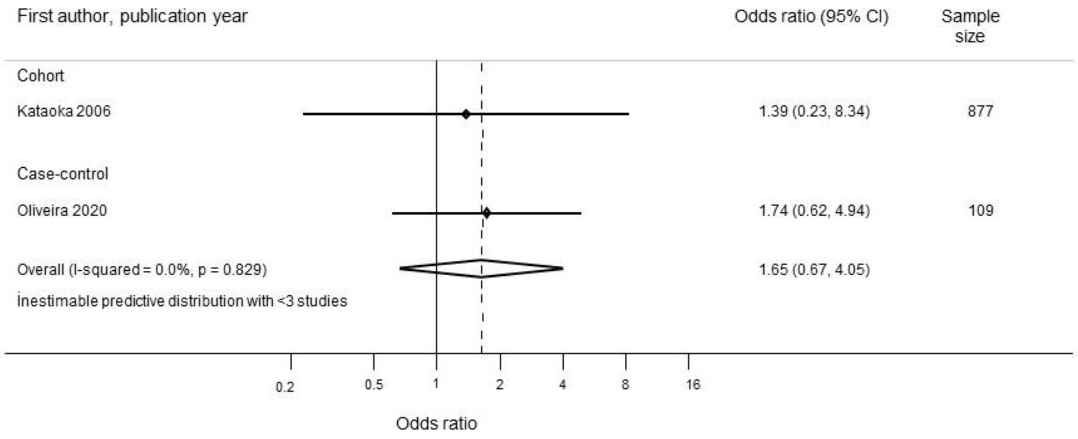

Figure S5.3 Forest plot of association between *U. parvum* and spontaneous abortion random effects model

Solid diamond shows the point estimate and study weight. Solid line shows 95% confidence intervals (CI) for each study. The open diamond shows the point estimate and 95% CI of the summary estimate. Solid lines extending from the diamond show the prediction interval.

Table S2 Summary of assessment of funnel plot asymmetry, for outcomes reported in 10 or more studies

Organism	Outcome	Egger test (95% CI)*	P value
M. hominis	PTB	0.56 (-0.08, 1.2)	0.09
	PROM	0.05 (-1.07, 1.17)	0.92
	SA	-0.28 (-3.20, 2.64)	0.83
U. urealyticum	PTB	0.89 (-0.15, 1.93)	0.09
	PROM	1.2 (-1.7, 4.09)	0.37
U. parvum	PTB	0.53 (-1.27, 2.34)	0.52

Abbreviations: CI, confidence interval; PND: perinatal death; PTB: preterm birth; PROM: premature rupture of membrane; S: spontaneous abortion

* Egger test for small-study effects

For peer review only - http://bmjopen.bmj.com/site/about/guidelines.xhtml

Supporting information

Table S3.1 Descriptive tables: Cohort studies (n=26)

First author, publication year	Organism reported	Outcomes	Definition Provided	OR/ RR (95% CI) reported in by study authors		
				Organism_ outcome	Unadjusted, OR	Adjusted, aOR
Agger, 2014	MH, UU, UP	PTB	Born < 37 weeks	MH_PTB	OR 1.72 (0.91, 3.28)	'Final model... factors… from preliminary models with p>0.15.' No organism in final multivariable model for PTB <37 weeks. MH in final model PTB< 35 weeks, aOR 3.6 (1.4-9.7)
				UU_PTB	OR 1.64 (0.67, 4.05)	
				UP_PTB	OR 1.23 (0.7, 2.15)	
Berman, 1987	MH	LBW	<2.5kg	MH_LBW	RR 1.8 (1.0-3.1)	Birth weight as continuous variable, p=0.01, adjusted for parity, maternal height, weight, marital status, age, enrolment, gestation, *C. trachomatis*
Braun, 1971	MH	LBW	<2.5kg			No multivariable analysis
		SA	Not defined	NR		
		PND	Not defined			
Donders, 2009	MH	PTB	Born < 37 weeks	MH_PTB	OR 8.5 (2.8, 25.5)	No multivariable analysis
		SA	**			

For peer review only - http://bmjopen.bmj.com/site/about/guidelines.xhtml

Supporting information

First author, publication year	Organism reported	Outcomes	Definition Provided	OR/ RR (95% CI) reported in by study authors		
				Organism_ outcome	Unadjusted, OR	Adjusted, aOR
Gerber, 2003	UU	PTB<br>PROM	Born < 37 weeks<br>†	NR		No multivariable analysis
Govender, 2009	MH, UU, UP	PTB	Born < 37 weeks	NR		No multivariable analysis
Harrison, 1983	MH	SA	**	NR		No multivariable analysis
Hillier, 1995	MH	PTB	Born < 37 weeks	MH_PTB		No multivariable analysis
Kataoka, 2006	MH, UU, UP	PTB,<br>PROM,<br>SA,<br>PND	Born < 37 weeks<br>Not defined<br>**<br>∞	NR		No multivariable analysis
Koucky, 2016	UU	PTB	Born < 37 weeks	NR		aOR 3.4 (1.3, 5.5)<br>Adjusted for progesterone treatment, other factors not reported 5.46 (1.80, 16.62)

For peer review only - http://bmjopen.bmj.com/site/about/guidelines.xhtml

Supporting information

First author, publication year	Organism reported	Outcomes	Definition Provided	OR/ RR (95% CI) reported in by study authors		
				Organism_ outcome	Unadjusted, OR	Adjusted, aOR
Kwak, 2014	MH, UU	PTB	Born < 37 weeks	NR		No multivariable analysis
Lee, 2016	MH, UU	PTB	Born < 37 weeks	NR		No multivariable analysis
		PROM	Not defined			
		SA	**			
Luton, 1994	MH	PTB	Born < 37 weeks	NR		No multivariable analysis
		LBW	<2.5kg			
		PND	∞			
McDonald, 1994	MH	PTB	Born < 37 weeks	NR		No multivariable analysis
Menard, 2010	MH	PTB	Born < 37 weeks	NR		
Minkoff, 1984	MH	PTB	Born < 37 weeks	NR		Stepwise multiple logistic regression. Results for MH not reported for either outcome
		PROM	†			

For peer review only

Supporting information

First author, publication year	Organism reported	Outcomes	Definition Provided	OR/ RR (95% CI) reported in by study authors		
				Organism_ outcome	Unadjusted, OR	Adjusted, aOR
Nguyen, 2004	MH	PTB	Born < 37 weeks	MH_PTB	RR 4.6 (1.7, 12.8)	No multivariable analysis
		PROM	†			
		PND	Not defined			
Odendaal, 2002	MH	PTB	Born < 37 weeks	NR		No multivariable analysis
		SA,	**			
		PND	Not defined			
Payne, 2016	MH, UU, UP	PTB	Born < 37 weeks	NR		No multivariable analysis
Payne, 2020	MH, UU, UP	PTB	Born < 37 weeks	NR		No multivariable analysis
Peretz, 2020	UU, UP	PTB,	Born < 37 weeks	NR		No multivariable analysis
		LBW	<2.5kg			
Perni, 2004	MH, UU	PTB,	Born < 37 weeks	NR		
		PROM	†			

For peer review only - http://bmjopen.bmj.com/site/about/guidelines.xhtml

Supporting information

First author, publication year	Organism reported	Outcomes	Definition Provided	OR/ RR (95% CI) reported in by study authors		
				Organism_ outcome	Unadjusted, OR	Adjusted, aOR
Rittenschober-Bohm, 2018	UU, UP	PTB	Born < 37 weeks	UU_PTB<br>UP_PTB	OR 1.4 (0.9, 2.3)<br>OR 1.7 (1.3, 2.2)	aOR 1.4 (0.8, 2.2)<br>aOR 1.6 (1.2, 2.1)<br>Adjusted for age, smoking, history of PTB, BV, smoking UU or UP
Schwab, 2015	MH, UU	PTB	Born < 37 weeks	MH_PTB<br>UU_PTB	OR 0.26 (0.03, 1.13)<br>OR 0.52 (0.15, 1.57)	No multivariable analysis
Sperling, 1988	MH,	LBW	<2.5kg	NR		No multivariable analysis
Usui, 2002	MH, UU	PTB	Born < 37 weeks	NR	OR not reported by study authors	aOR 1.5 (0.8, 2.7)<br>Adjusted for *Lactobacillus* spp., *E. coli*, glucose non-fermenting Gram negative rods

Abbreviations LBW: low birth weight, defined as birth weight <2.5kg; NAAT, nucleic acid amplification tests; NR, not reported; MH, *M. hominis*; PND, perinatal mortality; PROM, premature rupture of membrane; PTB, preterm birth; SA: spontaneous abortion; UP, *U. parvum*; UU, *U. urealyticum*;

For peer review only - http://bmjopen.bmj.com/site/about/guidelines.xhtml

Supporting information

**SA defined as pregnancy loss at <20 weeks gestation or as defined by author; †PROM defined as rupture of membrane before 37 completed weeks or as defined by author; ∞ Perinatal death, defined as birth after 20 weeks gestation (stillbirth) or death within 28 days after birth (neonatal death) unless otherwise defined by the study authors.

For peer review only

Supporting information

Table S3.2 Descriptive tables: Case control studies (n=25)

First author, publication year	Organism reported	Outcome	Definition Provided	OR/ RR (95% CI) reported by study authors		
				Organism outcome	Unadjusted, OR	Adjusted, aOR
Ahmadi, 2014	UU	SA	**	NR		No multivariable analysis
Bosquet, 2006	MH	PTB	Born < 37 weeks	NR		No multivariable analysis
Cassell, 1983	MH	PTB<br>PND	Born < 37 weeks<br>∞	NR		No multivariable analysis
Chua, 1999	MH	PTB	Born < 37 weeks	NR		No multivariable analysis
Daskalakis, 2009,	MH, UU	PTB	Born < 37 weeks	NR		No multivariable analysis
Embree,1980	MH	SA<br>PND	Not defined<br>Partially defined			No multivariable analysis
arhadifar, 2017	MH	SA	**	MH_SA	OR 0.49 (0.08, 2.73)	No multivariable analysis
Freitas, 2018	UU, UP	PTB	Born < 37 weeks			No multivariable analysis

For peer review only - http://bmjopen.bmj.com/site/about/guidelines.xhtml

Supporting information

First author, publication year	Organism reported	Outcome	Definition Provided	OR/ RR (95% CI) reported by study authors		
				Organism outcome	Unadjusted, OR	Adjusted, aOR
						No multivariable analysis
Harada, 2008	UU	PTB	Not defined	NR		No multivariable analysis
Hillier, 1988	MH	PTB	Born < 37 weeks	NR		No multivariable analysis
Holst, 1994	MH	PTB	Born < 37 weeks	NR		No multivariable analysis
Jalava, 2002	UU	PTB	Born < 37 weeks	UU_PTB	RR 3.34 (1.27, 8.8)	No multivariable analysis
Jones, 2009	MH, UU, UP	PTB<br>PROM	Born < 37 weeks<br>†	NR		No multivariable analysis
Kacerovsky, 2009	MH, UU	PROM	†	NR		No multivariable analysis
Kafetzis, 2004	UU	PTB<br>PROM<br>PND	Born < 37 weeks<br>†<br>Not defined	NR		No multivariable analysis

For peer review only - http://bmjopen.bmj.com/site/about/guidelines.xhtml

Supporting information

First author, publication year	Organism reported	Outcome	Definition Provided	OR/ RR (95% CI) reported by study authors		
				Organism outcome	Unadjusted, OR	Adjusted, aOR
Kumar, 2006	MH	PTB	Born < 37 weeks			No multivariable analysis
McDonald, 1992	MH	PTB	Born < 37 weeks	MH_PTB	OR 1.7 (0.9, 3.5)	aOR 1.1 (0.5, 2.5)
		PROM	Not defined	MH_PROM	OR 1.5 (0.5, 4.3)	aOR 1.1 (0.3, 3.7)<br>Adjusted for ‘confounding demographic and obstetric variables’
Mitsunari, 2005	UU	PTB	Not defined	NR		No multivariable analysis
		PROM	Not defined			
Montenegro, 2019	MH, UU	PTB	Born < 37 weeks	NR		No multivariable analysis
		PROM	Not defined			
Munday, 1984	MH	SA	Not defined	NR		No multivariable analysis

For peer review only - http://bmjopen.bmj.com/site/about/guidelines.xhtml

Supporting information

First author, publication year	Organism reported	Outcome	Definition Provided	OR/ RR (95% CI) reported by study authors		
				**Organism outcome**	**Unadjusted, OR**	**Adjusted, aOR**
Oliveira, 2020	MHUU, UP	SA	**	MH_SA<br>UU_SA<br>UP_SA	OR 0.08 (0.2, 3.17)<br>OR 2.21 (0.6, 8.22)<br>OR 1.74 (0.61, 4.93)	No multivariable analysis
Payne, 2014	UU, UP	PTB	Born < 37 weeks			NR
Povlsen, 2001	UU	PTB<br>LBW	Born < 37 weeks<br><2.5kg	UU_PTB	OR 1.0 (0.6, 1.7)	aOR 0.7 (0.4, 1.2)<br>Adjusted for LBW
Toth, 1992	MH	PTB	Born < 37 weeks	NR		No multivariable analysis
Yoon, 2001	UU	PTB<br>PROM	Born < 37 weeks<br>Not defined	NR		No multivariable analysis

Abbreviations LBW: low birth weight defined as birth weight <2.5kg; NAAT, nucleic acid amplification tests; NR, not reported; MH, *M. hominis*; PND, perinatal mortality; PROM, premature rupture of membrane; PTB, preterm birth; SA: spontaneous abortion; UP, *U. parvum*; UU, *U. urealyticum*

For peer review only - http://bmjopen.bmj.com/site/about/guidelines.xhtml

Supporting information

**SA defined as pregnancy loss at <20 weeks gestation or as defined by author; †PROM defined as rupture of membrane before 37 completed weeks or as defined by author.

Table S3.3 Descriptive tables: Cross sectional studies (n=6)

First author, publication year	Organism reported	Total enrolled	Outcome	Definition Provided	OR/ RR (95% CI) reported by study authors		
					Organism outcome	Unadjusted, OR	Adjusted, aOR
**Abele-Horn, 2000**	UU	295	PTB<br>PROM	Born < 37 weeks<br>†	NR	Multivariable analysis reported in text: UU $>10^5$ cfu/ml associated with PTB, adjusted for PROM, prolonged rupture of membranes, chorioamnionitis, obstetric risk factors	
**Grattard, 1995**	MH	208	PTB,<br>PROM<br>LBW	Born < 37 weeks<br>†<br><2.5kg	NR	No multivariable analysis	
**Kundsin, 1984**	MH	801	PND	∞	NR	No multivariable analysis	

For peer review only - http://bmjopen.bmj.com/site/about/guidelines.xhtml

Supporting information

First author, publication year	Organism reported	Total enrolled	Outcome	Definition Provided	OR/ RR (95% CI) reported by study authors		
					Organism outcome	Unadjusted, OR	Adjusted, aOR
**McCormack, 1975**	MH	327	LBW<br>PND	<2.5kg<br>Not defined	NR	No multivariable analysis	
**Nasution, 2007**	MH, UU	120	PROM	Not defined	NR	No multivariable analysis	
**Sweeney, 2016**	UU, UP	535	PTB	Born < 37 weeks	NR	No multivariable analysis	

Abbreviations LBW: low birth weight defined as birth weight <2.5kg; NAAT, nucleic acid amplification tests; NR, not reported; MH, *M. hominis*; PND, perinatal mortality; PROM, premature rupture of membrane; PTB, preterm birth; SA: spontaneous abortion; UP, *U. parvum*; UU, *U. urealyticum*

†PROM defined as rupture of membrane before 37 completed weeks or as defined by author; ∞ Perinatal death, defined as birth after 20 weeks gestation (stillbirth) or death within 28 days after birth (neonatal death) unless otherwise defined by the study authors.

For peer review only - http://bmjopen.bmj.com/site/about/guidelines.xhtml

Supporting information

Table S4.1 Summary description of studies reporting *M. hominis* (n=42), by income status

First author, Pub year, country	Gestational age assessment	Sample size for outcome of interest; Number of adverse outcomes in women with *M. hominis*/ total number of women with adverse outcome (%)					NICE checklist criteria fulfilled*
		PTB	LBW	PROM	SA	PND	
**High-income country‡**							
Agger, 2014, USA	NR	676<br>14/54 (26)					+/+
Braun, 1971, USA	LMP		485<br>24/42 (57)		491<br>3/6 (50)	491<br>7/10 (70)	+/-
Cassell, 1983, USA	US	61<br>1/10 (10)				61<br>0/3 (0)	+/-
Donders, 2009, Belgium	US	744<br>5/50 (10)			759<br>1/15 (7)		+/+
Embree, 1980, Canada	LMP, NN assessment				157<br>3/10 (30)	157<br>0/39 (0)	-/-

For peer review only - http://bmjopen.bmj.com/site/about/guidelines.xhtml

Supporting information

First author, Pub year, country	Gestational age assessment	Sample size for outcome of interest; Number of adverse outcomes in women with *M. hominis*/ total number of women with adverse outcome (%)					NICE checklist criteria fulfilled*
		PTB	LBW	PROM	SA	PND	
Grattard, 1995, France	NR	193<br>3/8 (38)	202<br>2/8 (25)	208<br>4/36 (11)			-/+
Harrison, 1983, USA	NR				348<br>4/22 (18)		-/-
Hillier, 1988, USA	US, FH, LMP	112<br>3/38 (8)					+/+
Hillier, 1995, USA	LMP	9105<br>161/423 (38)					
Holst, 1994, Sweden	US, LMP	87<br>10/22 (45)					++/+
Jones, 2009, United Kingdom	NR	74<br>2/53 (4)		74<br>2/26 (8)			-/-

For peer review only - http://bmjopen.bmj.com/site/about/guidelines.xhtml

Supporting information

First author, Pub year, country	Gestational age assessment	Sample size for outcome of interest; Number of adverse outcomes in women with *M. hominis*/ total number of women with adverse outcome (%)					NICE checklist criteria fulfilled*
		PTB	LBW	PROM	SA	PND	
Kataoka, 2006, Japan	US, LMP	872<br>4/16 (25)		872<br>1/7 (14)	877<br>0/5 (0)	872<br>0/1 (0)	+/+
Kundsin, 1984, USA	NR					801<br>5/29 (17)	-/+
Kwak, 2014, South Korea	NR	112<br>13/86 (15)					+/+
Lee, 2016, South Korea	NR	466<br>1/141 (<1)		466<br>0/187 (0)	466<br>0/11 (0)		-/-
McCormack,1975, USA	NR		326<br>3/42 (7)			326<br>2/6 (33)	+/-
McDonald, 1992, Australia	LMP, US	786<br>11/135 (8)		708<br>4/57 (8)			-/-

For peer review only - http://bmjopen.bmj.com/site/about/guidelines.xhtml

Supporting information

First author, Pub year, country	Gestational age assessment	Sample size for outcome of interest; Number of adverse outcomes in women with *M. hominis*/ total number of women with adverse outcome (%)					NICE checklist criteria fulfilled*
		PTB	LBW	PROM	SA	PND	
McDonald, 1994, Australia	US, LMP	337<br>7/45 (16)					-/-
Menard,2010, France	US, LMP	90<br>6/36 (17)					-/-
Minkoff, 1984, USA	NR	201<br>10/18 (56)		188<br>21/40 (53)			-/-
Munday, 1984, United Kingdom	NR				241<br>9/76 (12)		+/-
Nguyen,2004, Switzerland	NR	395<br>3/10 (30)		365<br>0/7 (0%)		395<br>1/6 (17)	+/+
Payne, 2016, Australia	NR	187<br>2/13 (15)					+/+

For peer review only - http://bmjopen.bmj.com/site/about/guidelines.xhtml

Supporting information

First author, Pub year, country	Gestational age assessment	Sample size for outcome of interest; Number of adverse outcomes in women with *M. hominis*/ total number of women with adverse outcome (%)					NICE checklist criteria fulfilled*
		PTB	LBW	PROM	SA	PND	
Payne, 2020, Australia	NR	1000<br>9/118 (8)					+/+
Sperling, 1988, USA	NR		404<br>14/37 (38)				-/-
Toth, 1992, United Kingdom	NR	80<br>3/39 (8)					-/-
Usui, 2002, Japan	LMP	1958<br>15/342 (4)					+/-
**Upper-middle income‡**							
Berman, 1987, Mexico	NR		796<br>28/48 (58)				-/+

For peer review only - http://bmjopen.bmj.com/site/about/guidelines.xhtml

Supporting information

First author, Pub year, country	Gestational age assessment	Sample size for outcome of interest; Number of adverse outcomes in women with *M. hominis*/ total number of women with adverse outcome (%)					NICE checklist criteria fulfilled*
		PTB	LBW	PROM	SA	PND	
Chua, 1999, Malaysia	LMP, NN assessment	120<br>9/60 (15)					+/+
Farhadifar, 2016, Iran	US/LMP				218<br>2/109 (2)		+/+
Govender, 2009, South Africa	NR	199<br>11/20 (55)					-/-
Luton, 1994, Gabon	US, LMP	181<br>11/20 (55)	181<br>8/13 (62)			198<br>5/10 (50)	-/-
Montenegro, 2019, Colombia	NR	211<br>1/84 (1)		211<br>0/3 (0)			+/+
Odendaal, 2002, South Africa	US	395<br>33/119 (28)			395<br>1/7 (14)	395<br>2/4 (50)	+/-

For peer review only - http://bmjopen.bmj.com/site/about/guidelines.xhtml

Supporting information

First author, Pub year, country	Gestational age assessment	Sample size for outcome of interest; Number of adverse outcomes in women with *M. hominis*/ total number of women with adverse outcome (%)					NICE checklist criteria fulfilled*
		PTB	LBW	PROM	SA	PND	
Oliveria, 2020, Brazil	NR				109<br>11/89 (12)		+/+
**Lower-middle/low income‡**							
Schwab,2015, Indonesia	LMP	62<br>2/23 (9)					-/-
Kumar, 2006, India	NR	120<br>4/60 (7)					+/+
**Country not reported**							
Gonzàlez Bosquet, 2006	US	120<br>0/70 (0)					+/+
Daskalakis, 2009	US, LMP	37<br>8/25 (32)					+/+

Supporting information

First author, Pub year, country	Gestational age assessment	Sample size for outcome of interest; Number of adverse outcomes in women with *M. hominis*/ total number of women with adverse outcome (%)					NICE checklist criteria fulfilled*
		PTB	LBW	PROM	SA	PND	
Kacerovsky, 2009	NR			450<br>63/225 (28)			-/-
Nasution, 2007	NR			80<br>4/40 (10)			-/-
Perni, 2004	NR	179<br>0/10 (0)		179<br>2/5 (40)			+/+

Abbreviations: MH, *Mycoplasma hominis*; UU, *Ureaplasma urealyticum*; UP, *Ureaplasma parvum*; NR, not reported.

LBW: low birth weight, defined as birth weight <2.5kg ; PTB: preterm birth, defined as delivery before 37weeks gestation; PROM: premature rupture of membranes, - defined as clinically confirmed rupture of membrane before 37weeks gestation; PND, perinatal mortality, defined as stillbirth (death after 20/40 gestation) or neonatal death (death <28 day since birth), unless otherwise defined by the study authors; SA: spontaneous abortion- defined as pregnancy loss at <20/40 or as defined by author.

For peer review only - http://bmjopen.bmj.com/site/about/guidelines.xhtml

Supporting information

* UK National Institute of Health and Care Excellence (NICE) checklist, summary of criteria for internal/external validity: +/+ Some checklist criteria fulfilled; -/-Few or no checklist criteria fulfilled

‡ high-income ($12,376 or more); upper-middle income ($3,996 to $1,2375); lower-middle-income ($1,025 to $3,995); low-income ($1,025 or less) [Source: World Bank, Gross national income per capita, 2019-2020 https://datatopics.worldbank.org/world-development-indicators/the-world-by-income-and-region.html]

For peer review only

Supporting information

Table S4.2 Summary description of studies reporting *U. urealyticum* (n=31), by income status

First author, pub. year, country	Gestational age assessment	Sample size for outcome of interest; Number of adverse outcomes in women with *U. urealyticum*/ total number of women with adverse outcome (%)					NICE checklist criteria fulfilled
		**PTB**	**LBW**	**PROM**	**SA**	**PND**	
**High-income country**[‡]							
Abele-Horn, 2000, Germany	US, LMP	295<br>57/ 73 (78)		295<br>58/76 (76)			-/++
Agger, 2014, USA	NR	676<br>4/54 (11)					+/+
Freitas, 2018, Canada	NR	216<br>0/46 (0)					+/+
Harada, 2008, Japan	NR	145<br>23/45 (51)					+/+

For peer review only - http://bmjopen.bmj.com/site/about/guidelines.xhtml

Supporting information

First author, pub. year, country	Gestational age assessment	Sample size for outcome of interest; Number of adverse outcomes in women with *U. urealyticum*/ total number of women with adverse outcome (%)					NICE checklist criteria fulfilled
		PTB	LBW	PROM	SA	PND	
Jones, 2009, UK	NR	74<br>2/53 (4)		74<br>2/26 (8)			-/-
Kafetzis, 2004, Greece	NR	251<br>46/126 (37)		250<br>9/20 (45)		171<br>6/16 (38)	+/+
Kataoka, 2006, Japan	US, LMP	872<br>0/16 (0)		8772<br>0/7 (0)	877<br>1/5 (20)	872<br>0/1 (0)	+/+
Koucky, 2016, Czech Republic	US, LMP	63<br>17/29 (59)					+/+
Kwak, 2014, South Korea	NR	179<br>84/129 (65)					+/+
Lee, 2016, South Korea	NR	936<br>72/141 (51)		936<br>100/187 (53)	936<br>12/23 (52)		-/-

For peer review only - http://bmjopen.bmj.com/site/about/guidelines.xhtml

Supporting information

First author, pub. year, country	Gestational age assessment	Sample size for outcome of interest; Number of adverse outcomes in women with *U. urealyticum*/ total number of women with adverse outcome (%)					NICE checklist criteria fulfilled
		**PTB**	**LBW**	**PROM**	**SA**	**PND**	
Mitsunari, 2005, Japan	NR	82<br>17/21 (81)		82<br>10/11 (91)			+/+
Payne, 2014, China & Australia	US	972<br>2/13 (15)					+/+
Payne, 2016, Australia	NR	187<br>0/115 (0)					+/+
Payne, 2020, Australia	NR	1000<br>14/118 (12)					+/+
Povlsen, 2001, Denmark	NR	484<br>49/84 (58)	484<br>36/48 (75)				+/+
Peretz, 2020, Israel	NR	214<br>3/5 (60)	214<br>1/3 (33)				-/-

For peer review only - http://bmjopen.bmj.com/site/about/guidelines.xhtml

Supporting information

First author, pub. year, country	Gestational age assessment	Sample size for outcome of interest; Number of adverse outcomes in women with *U. urealyticum*/ total number of women with adverse outcome (%)					NICE checklist criteria fulfilled
		PTB	LBW	PROM	SA	PND	
Rittenschober-Böhm, 2018, Austria	US	2183<br>19/146 (13)					+/+
Sweeney, 2016, USA	NR	535<br>6/443 (1)					+/-
Usui, 2002, Japan	NR	1958<br>189/342 (55)					+/-
Yoon, 2001, South Korea	NR	114<br>3/19 (16)		Missing data<br>2/9 (22)**			+/+
**Upper-middle income**‡							
Ahmadi, 2014, Iran	US, LMP				218<br>18/109 (17)		+/+

For peer review only - http://bmjopen.bmj.com/site/about/guidelines.xhtml

Supporting information

First author, pub. year, country	Gestational age assessment	Sample size for outcome of interest; Number of adverse outcomes in women with *U. urealyticum*/ total number of women with adverse outcome (%)					NICE checklist criteria fulfilled
		**PTB**	**LBW**	**PROM**	**SA**	**PND**	
Govender,2009, South Africa	NR	199<br>5/20 (25)					-/-
Oliveiria, 2020, Brazil	NR				109<br>25/89 (28)		+/+
Montenegro, 2019, Colombia	NR	211<br>0/84 (0)		211<br>0/3 (0)			+/+
**Lower-middle income**[‡]							
Schwab, 2015, Indonesia	LMP	62<br>2/23 (9)					-/-
**Country not reported**							
Daskalakis, 2009	US, LMP	37<br>17/25 (68)					+/+

For peer review only - http://bmjopen.bmj.com/site/about/guidelines.xhtml

Supporting information

First author, pub. year, country	Gestational age assessment	Sample size for outcome of interest; Number of adverse outcomes in women with *U. urealyticum*/ total number of women with adverse outcome (%)					NICE checklist criteria fulfilled
		**PTB**	**LBW**	**PROM**	**SA**	**PND**	
Gerber, 2003,	NR	254<br>9/10 (90)		254<br>6/7 (86)			+/-
Jalava, 2002	NR	122<br>12/17 (71)					+/+
Kacerovsky, 2009	NR			450<br>152/225 (68)			-/-
Nasution, 2007	NR			80<br>9/40 (23)			-/-
Perni, 2004	Cohort	NR	172<br>0/10 (0)		172<br>3/5 (60)		+/+

Abbreviations: MH, *Mycoplasma hominis*; UU, *Ureaplasma urealyticum*; UP, *Ureaplasma parvum*; NR, not reported.

For peer review only - http://bmjopen.bmj.com/site/about/guidelines.xhtml

Supporting information

LBW: low birth weight, defined as birth weight <2.5kg ; PTB: preterm birth, defined as delivery before 37weeks gestation; PROM: premature rupture of membranes, - defined as clinically confirmed rupture of membrane before 37weeks gestation; PND, perinatal mortality, defined as stillbirth (death after 20/40 gestation) or neonatal death (death <28 day since birth), unless otherwise defined by the study authors; SA: spontaneous abortion- defined as pregnancy loss at <20/40 or as defined by author.

* UK National Institute of Health and Care Excellence (NICE) checklist, summary of criteria for internal/external validity: +/+ Some checklist criteria fulfilled; -/-Few or no checklist criteria fulfilled

‡high-income ($12,376 or more); upper-middle income ($3,996 to $1,2375); lower-middle-income ($1,025 to $3,995); low-income ($1,025 or less) [Source: World Bank, Gross national income per capita, 2019-2020 https://datatopics.worldbank.org/world-development-indicators/the-world-by-income-and-region.html]

For peer review only

Supporting information

Table S4.3 Summary description of studies reporting *U. parvum* (n=12), by income status

**First author, Pub. year, country**	**Study design**	**Gestational age assessment**	**Sample size for outcome of interest. Number of adverse outcomes in women with *U. parvum*/ total number of women with adverse outcome (%)**					**NICE checklist criteria fulfilled**
			**PTB**	**LBW**	**PROM**	**SA**	**PND**	
**Upper-middle and high-income country‡**								
**Agger, 2014, USA**	Cohort	NR	676<br>29/54 (54)					+/+
**Freitas, 2018, Canada**	Case-control	NR	216<br>14/46 (30)					+/+
**Govender, 2009, South Africa**	Cohort	NR	199<br>10/20 (50)					-/-
**Jones, 2009,** United Kingdom	Case-control	NR	74<br>19/53 (36)		74<br>11/26 (42)			-/-
**Kataoka, 2006, Japan**	Cohort	US, LMP	872<br>4/16 (25)		872<br>6/7 (86)	877<br>3/5 (60)	872<br>1/1 (100)	+/+

For peer review only - http://bmjopen.bmj.com/site/about/guidelines.xhtml

Supporting information

**Oliveiia, 2020, Brazil**	Case-control	NR			109<br>68/89 (76)	+/+
**Payne, 2014, China & Australia**	Case-control	NR	972<br>2/115 (2)			+/+
**Payne, 2016, Australia**	Cohort	NR	187<br>10/13 (77)			+/+
**Payne, 2020, Australia**	Cohort	NR	1000<br>56/118 (48)			+/+
**Peretz, 2020, Israel**	Cohort	NR	214<br>1/5 (20)	214<br>1/3 (33)		-/-
**Rittenschober-Böhm, 2018, Austria**	Cohort	US	3316<br>140/267 (52)			+/+
**Sweeney, 2016, USA**	Cross-sectional	NR	535<br>27/443 (4)			+/-

Abbreviations: MH, *Mycoplasma hominis*; UU, *Ureaplasma urealyticum*; UP, *Ureaplasma parvum*; NR, not reported.

For peer review only - http://bmjopen.bmj.com/site/about/guidelines.xhtml

Supporting information

LBW: low birth weight, defined as birth weight <2.5kg ; PTB: preterm birth, defined as delivery before 37weeks gestation; PROM: premature rupture of membranes, - defined as clinically confirmed rupture of membrane before 37weeks gestation; PND, perinatal mortality, defined as stillbirth (death after 20/40 gestation) or neonatal death (death <28 day since birth), unless otherwise defined by the study authors; SA: spontaneous abortion- defined as pregnancy loss at <20/40 or as defined by author.

* UK National Institute of Health and Care Excellence (NICE) checklist, summary of criteria for internal/external validity: +/+ Some checklist criteria fulfilled; -/-Few or no checklist criteria fulfilled

‡ high-income ($12,376 or more); upper-middle income ($3,996 to $1,2375) [Source: World Bank, Gross national income per capita, 2019-2020 https://datatopics.worldbank.org/world-development-indicators/the-world-by-income-and-region.html]

For peer review only

For peer review only - http://bmjopen.bmj.com/site/about/guidelines.xhtml

Supporting information

Table S5.1 Study setting and socio-demographics, cohort studies (n=26)

First author, year of publication	Location of study	Study setting	Urban/ rural location	Mean†/ median age years (range)	Ethnicity	Other infections included/ (excluded)	Smokers included (%)	Multiple pregnancies
**Agger, 2014**	USA	NR/unclear;	Mixed	NR	Mixed	CT, NG, HPV, herpes, syphilis‡	NR	Yes
**Berman, 1987**	Mexico	Health facility	NR/unclear	NR	NR	CT	NR	No
**Braun, 1971**	USA	Health facility	Urban	NR	Mixed	NR	NR	NR
**Donders, 2009**	Belgium	Health facility	Urban	29†	Mixed	BV; (CT, TV, NG, syphilis)	120/761 (15.8%)	No
**Gerber, 2003**	NR	Health facility	NR/unclear	19-42	White	NR	NR	Yes
**Govender, 2009**	South Africa	Health facility	Urban	NA	NR	HIV, CT‡	NR	NR
**Harrison, 1983**	USA	Health facility	Urban	NR	Mixed	CT	NR	Yes
**Hillier, 1995**	USA	Health facility	Urban	NR	Mixed	BV, NG, CT, TV	Yes but #/%NR	No
**Kataoka, 2006**	Japan	Health facility	Urban	28.9†	NR	CT, NG‡	NR	No

For peer review only - http://bmjopen.bmj.com/site/about/guidelines.xhtml

Supporting information

First author, year of publication	Location of study	Study setting	Urban/ rural location	Mean†/ median age years (range)	Ethnicity	Other infections included/ (excluded)	Smokers included (%)	Multiple pregnancies
**Koucky, 2016**	Czech Republic	Health facility	Urban	31	NR	NR	NR	No
**Kwak, 2014**	South Korea	Health facility	Urban	30.7	NR	NR	NR	No
**Lee, 2016**	South Korea	Health facility	Urban	31 (15-47)	NR	NR	NR	NR
**Luton, 1994**	Gabon	Health facility	NR/unclear	NR	NR	HIV, CT, TV, NG, Syphilis	NR	No
**Minkoff, 1984**	USA	Health facility	NR	27† (17-39)	Mixed	CT, TV	NR	Yes
**McDonald, 1994**	Australia	Health facility	NR	NR	NR	NR	NR	NR
**Menard, 2010**	France	Health facility	Urban	NR	NR	BV	NR	No
**Nguyen, 2004**	Switzerland	Health facility	Urban	19-42	NR	NR	NR	No
**Odendaal, 2002**	South Africa	Health facility	Urban	NR	NR	CT, BV, NG	161/395 (40.8%)	No

For peer review only - http://bmjopen.bmj.com/site/about/guidelines.xhtml

Supporting information

First author, year of publication	Location of study	Study setting	Urban/ rural location	Mean†/ median age years (range)	Ethnicity	Other infections included/ (excluded)	Smokers included (%)	Multiple pregnancies
**Payne, 2016**	Australia	Health facility	Urban	30 (18-43)	Mixed	NR	21/191 (11%)	No
**Payne, 2020**	Australia	Health facility	Urban	NR	Mixed	(HIV) ‡	135/ 1000 (13.5%)	No
**Peretz, 2020**	Israel	Health facility	Urban	29.8†	Mixed	NR ‡	NR	Yes
**Perni, 2004**	NR	Health facility	NR/unclear	18-44	Mixed	NR	NR	No
**Rittenschober-Böhm, 2018**	Austria	Health facility	Urban	30.3†	NR	BV	670/3643 (18.4%)	No
**Schwab, 2015**	Indonesia	Health facility	Urban	26.6† (17-42)	NR	CT, BV, NG	NR	NR
**Sperling, 1988**	USA	Health facility	Urban	NR	Mixed	NR	NR	NR
**Usui, 2002**	Japan	Health facility	Urban	NR	Asian	CT	NR	No

Abbreviations: BV, bacterial vaginosis; CT, *Chlamydia trachomatis*; HIV, human immunodeficiency virus; NG, *Neisseria gonorrhoeae*; NR, not reported; infections; TV, *Trichomonas vaginalis*; USA, United States of America;

†reported mean age; ‡Detected *Mycoplasma genitalium*

For peer review only - http://bmjopen.bmj.com/site/about/guidelines.xhtml

Supporting information

Table S5.2 Study setting and socio-demographics, case-control studies (n=25)

First author, year of publication	Location of study	Study setting	Urban /rural location	Mean†/ median age years (range)	Ethnicity	Other infections included (or excluded)	Smokers included/ (%)	Multiple pregnancies
**Ahmadi, 2014**	Iran	Health facility	Urban	19-43	NR	NR	3/218 (1.4)	NR
**Gonzàlez Bosquet, 2006**	NR	Health facility	NR/ unclear	NR	NR	CA, BV-associated bacteria, *E. coli*, GBS, TV,	NR	No
**Cassell, 1983**	USA	Health facility	Urban	NR	White, Black	NR	NR	NR
**Chua, 1999**	Malaysia	Health facility	Urban	NR	NR	NR	NR	No
**Daskalakis, 2009**	NR	Health facility	Urban	NR	NR	NR	36/144 (25)	No
**Embree,**	Canada	Health facility	Urban	14-45	NR	NR	NR	Yes
**Farhadifar, 2016**	Iran	Health facility	Urban	25 (19-43)	NR	NR	NR	NR
**Freitas, 2018**	Canada	Health facility	Urban	33† (21-45)	Mixed	NR	4/216 (2.3%)	NR
**Harada, 2008**	Japan	Health facility	Urban	NR	NR	NR	NR	No

For peer review only - http://bmjopen.bmj.com/site/about/guidelines.xhtml

Supporting information

**Hillier, 1988**	USA	Health facility	Urban	NR	NR	CT, TV, BV	NR	No
**Holst, 1994**	Sweden	Health facility	Urban	NR	NR	CT, BV, NG	20/49 (40.8)	No
**Jalava, 2002**	NR	Health facility	NR/ unclear	NR	NR	(CT)	NR	NR
**Jones, 2009**	United Kingdom	Health facility	Urban	NR	NR	NR	NR	No
**Kacerovsky, 2009**	NR	Health facility	NR/ unclear	26 (19-38)	NR	NR	NR	No
**Kafetzis, 2004**	Greece	Health facility	Urban	NR	NR	NR	NR	NR
**Kumar, 2006**	India	Health facility	Urban	NR	NR	BV	NR	NR
**McDonald, 1992**	Australia	Health facility	Urban	NR	NR	NR	839/ 2190 (39.8%)	NR
**Mitsunari, 2005**	Japan	Health facility	Urban	NR	Asian	(CT)	NR	No
**Montenegro, 2019**	Colombia	Health facility	Urban	NR	NR	NR	NR	NR
**Munday, 1984**	United Kingdom	Health facility	Urban	NR	Mixed	CT	NR	NR

Supporting information

**Oliveira, 2020**	Brazil	Health facility	Urban	27.3	Mixed	NG ‡	5/109 (4.6)	NR
**Payne, 2014**	China, Australia	Health facility	NR/unclear	17-49	Mixed	NR	69/972 (7.1%)	No
**Povlsen, 2001**	Denmark	Health facility	Urban	NR	NR	BV	NR	No
**Toth, 1992**	United Kingdom	Health facility	Urban	NR	NR	CT, TV	NR	NR
**Yoon, 2001***	South Korea	Health facility	Urban	NR	NR	NR	NR	No

Abbreviations: BV, bacterial vaginosis; CA, *Candida albicans*; CT, *Chlamydia trachomatis*; HIV, human immunodeficiency virus; NG, *Neisseria gonorrhoeae*; NR, not reported; TV, *Trichomonas vaginalis*; USA, United States of America

† Reported mean age; ‡detected *Mycoplasma genitalium*

For peer review only - http://bmjopen.bmj.com/site/about/guidelines.xhtml

Supporting information

Table S5.3 Study setting and socio-demographics, cross-sectional studies (n=6)

First author, year of publication	Location of study	Study setting	Urban /rural location	Mean†/ median age years (range)	Ethnicity	Other infections included (or excluded)	Smokers included/ (%)	Multiple pregnancies
**Abele-Horn, 2000**	Germany	Health facility	Urban	NR	Mixed	(BV, CT, NG, TV, yeast)	NR	NR
**Grattard, 1995**	France	Health facility	Urban	NR	NR	NR	NR	NR
**Kundsin, 1984**	USA	Health facility	Urban	NR	Mixed	NR	105/801 (31.4%)	Yes
**McCormack, 1975**	USA	Health facility	Urban	23.6†	Mixed	NR	NR	Yes
**Nasution, 2007**	NR	Health facility	NR/ unclear	24-38	Asian	CT, NG	NR	NR
**Sweeney, 2016**	USA	Health facility	Urban	NR	Mixed	NR	NR	Yes

Abbreviations: BV, bacterial vaginosis; CT, *Chlamydia trachomatis*; HIV, human immunodeficiency virus; NG, *Neisseria gonorrhoeae*; NR, not reported; TV, *Trichomonas vaginalis*; UK, United Kingdom; USA, United States of America

† Reported mean age

For peer review only - http://bmjopen.bmj.com/site/about/guidelines.xhtml

Supporting information

Table S6 Studies that reported on bacterial vaginosis or sexually transmitted infections and reported associations with adverse birth outcomes

First author, publication year, reference number*	Study population	Organism/ outcome	OR (95% CI)	Reported associations with genital mycoplasmas
**Donders, 2009**[34]	Cohort study: 759 women; 55 PTB; 64 BV; 14 *M. hominis*	BV/PTB	2.43 (1.1, 4.7)	Association between lactobacilli and PTB, and between BV and PTB reported as primary analysis. Proportion of women with *M. hominis* but no BV reported (0.5% of 759), but association between *M. hominis* and PTB in absence of BV could not be calculated from data presented. Discussion includes, "In the literature, the presence of *M. hominis* has generally been related to an increased risk of miscarriage, and premature delivery if found in combination with bacterial vaginosis."
**Hillier, 1988**[57]	Case-control study: 94 women; 38 PTB; 28	BV/PTB	3.31 (1.20, 9.24)	Association between organisms in chorioamnion and PTB reported as primary analysis. BV measured in vaginal

For peer review only - http://bmjopen.bmj.com/site/about/guidelines.xhtml

Supporting information

First author, publication year, reference number*	Study population	Organism/ outcome	OR (95% CI)	Reported associations with genital mycoplasmas
	BV; 29 *U. urealyticum*; 5 *M. hominis*			smears. Association between genital mycoplasmas and PTB in absence of BV could not be calculated from data presented.
**Hillier, 1995**[31]	Cohort study: 9105 women; 423 PTB; 1392 BV; 2805 *M. hominis*	BV/ PTB<br>MH+, BV+/ PTB<br>MH+, BV-/PTB	1.60 (1.25, 2.03)<br>1.58 (0.94, 2.77)<br>1.18 (0.91, 1.52)	Association between BV and PTB of low birthweight infants reported as primary analysis. Raw data not available for association between *M. hominis* and PTB, but reported in text and can be extracted from bar chart of ORs for PTB, stratified by *M. hominis*, bacteroides and BV. OR for BV and BV with *M. hominis* similar, and stronger than association for *M. hominis* alone.

For peer review only - http://bmjopen.bmj.com/site/about/guidelines.xhtml

Supporting information

First author, publication year, reference number*	Study population	Organism/ outcome	OR (95% CI)	Reported associations with genital mycoplasmas
**Kumar, 2006**[71]	Case-control study: 120 women; 60 PTB; 31 BV; 6 *M. hominis*	BV/PTB	5.05 (1.97, 12.95)	Association between BV and PTB reported as primary analysis. Association between *M. hominis* and PTB in absence of BV could not be calculated from data presented. Discussion does not mention *M. hominis.*
**Menard, 2010**[41]	Cohort study: 90 women; 36 PTB; 2 BV; 10 *M. hominis*			Association between quantities of BV-associated bacteria and PTB reported as primary analysis. Association between *M. hominis* and PTB in absence of BV could not be calculated from data presented.
**Odendaal, 2002**[50]	Cohort study as sub-study of a randomised controlled trial: 395	MH/BV	10.21 (5.63, 18.65)	Association between *M. hominis* and PTB reported as primary analysis. Association between *M. hominis* and BV reported, but not association between BV and PTB. Discussion includes, "It is also possible that the BV is not

For peer review only - http://bmjopen.bmj.com/site/about/guidelines.xhtml

Supporting information

First author, publication year, reference number*	Study population	Organism/ outcome	OR (95% CI)	Reported associations with genital mycoplasmas
	women; 119 PTB; 132 BV; 83 *M. hominis*			directly involved in the causation of premature labour but that it is only a marker of a more important underlying condition such as *M. hominis* infection,"
**Povlsen, 2001**[64]	Nested case-control study: 484 women; 84 PTB; 70 BV; 280 *U. urealyticum*	BV/PTB<br>UU+, BV+/PTB<br>UU+/PV-/PTB	0.77 (0.33, 1.6)<br>0.47 (0.09, 3.31)<br>1.15 (0.67, 1.98)	Associations between *U. urealyticum* biovars and PTB reported as primary analysis. Numbers, stratified by BV status and low birth weight reported. Discussion mentions that *U. urealyticum* and BV were associated with each other overall, but that this association was only seen in women who delivered at term and was not associated with PTB.
**Rittenschober-Bohm, 2018**[46]	Cohort study: 3,643 women; 292 PTB; 279	BV/PTB<br><br>UP-,BV-/PTB	Crude 1.7 (1.3, 2.2)<br>Adjusted 1.6 (1.1, 2.4)	Associations between *Ureaplasma* spp. and PTB reported as primary analysis. Associations with *U. parvum*, stratified by BV status and adjusted for maternal age, diagnosis of vaginal

For peer review only - http://bmjopen.bmj.com/site/about/guidelines.xhtml

Supporting information

First author, publication year, reference number*	Study population	Organism/ outcome	OR (95% CI)	Reported associations with genital mycoplasmas
	BV; 1,347 *U. parvum*; 214 *U. urealyticum*	UP+,BV-/PTB<br>UP-,BV+/PTB<br>UP+,BV+/PTB	Adjusted 1.6 (1.2, 2.1)<br>Adjusted 1.6 (1.1, 2.3)<br>Adjusted 2.6 (1.7, 4.0)	candida, smoking and history of previous PTB. Stratified associations with *U. urealyticum* not reported on basis of univariable analysis (OR 1.4, 95% CI 0.8, 2.2). Discussion does not mention potential associations between both BV and *Ureaplasma* spp.
**Schwab, 2015**[52]	Cohort study: 62 women; 23 PTB; 13 BV; 13 *M. hominis*; 22 *U. urealyticum*	None reported		Descriptive study of infections in pregnancy. Association between *M. hominis*, *U. urealyticum* and PTB reported, but not association between BV and PTB.

Abbreviations: BV, bacterial vaginosis; CI, confidence interval; *M. hominis*, *Mycoplasma hominis*; PTB, premature birth; *U. parvum*, *Ureaplasma parvum*; *U. urealyticum*, *Ureaplasma urealyticum.*

For peer review only - http://bmjopen.bmj.com/site/about/guidelines.xhtml

Supporting information

* Study reference is the reference number cited in the main manuscript

For peer review only

For peer review only - http://bmjopen.bmj.com/site/about/guidelines.xhtml

Supporting information

Table S7.1 Risk of bias assessment, cohort studies (n=26)

Questions	Agger, 2014	Berman, 1987	Braun, 1971	Donders, 2009	Gerber, 2003
1) The method of allocation to exposure groups was unrelated to potential confounding factors	NA	NA	NA	NA	NA
2) Attempts made within design or analysis to balance both groups for potential confounders.	Yes	Unclear	Unclear	Unclear	Unclear
3) The groups were comparable at baseline, including all major confounding factors.	No	Yes	Unclear	Yes	Unclear
4) Based on above answers, was selection bias present?	No	Low	Unclear	Unclear	Unclear
5) If so, what is the likely direction of its effect?	Unclear	Unclear	Unclear	Unclear	Unclear
6) The comparison groups received the same care and support apart from the exposure(s) studied.	Yes	Unclear	Unclear	Yes	Unclear
7) Participants receiving care and support were kept 'blind' to intervention allocation.	NA	NA	NA	NA	NA

For peer review only - http://bmjopen.bmj.com/site/about/guidelines.xhtml

Supporting information

Questions	Agger, 2014	Berman, 1987	Braun, 1971	Donders, 2009	Gerber, 2003
8) Individuals administering care, support were kept 'blind' to intervention allocation.	NA	NA	NA	NA	NA
9) Based on above answers, was performance bias present?	Unclear	Unclear	Unclear	Unclear	Unclear
10) If so, what is the likely direction of its effect?	Unclear	Unclear	Unclear	Unclear	Unclear
11) All groups followed up for an equal length of time?	Low	Low	Low	Low	Low
12) Number of participants did not complete the intervention in each group?	NA	NA	NA	NA	NA
13) The groups were comparable for intervention completion.	NA	NA	Unclear	NA	NA
14) For how many participants were no outcome data available?[‡]	107/783, (13.7%)	104/1204 (8.6%)	203/688 (30%)	42/801 (5.2%)	63/317 (19.9%)
15) Were groups comparable for outcome data?	Unclear	Unclear	Unclear	Yes	Unclear
16) Based on above answers, was attrition bias present?	Unclear	Unclear	Unclear	Unclear	Unclear
17) If so, what is the likely direction of its effect?	Unclear	Unclear	Unclear	Unclear	Unclear
18) The study had an appropriate length of follow-up.	Yes	Yes	Yes	Yes	Yes
19) The study used a precise definition of outcome.	Yes	Unclear	No	Yes	Yes

For peer review only - http://bmjopen.bmj.com/site/about/guidelines.xhtml

Supporting information

Questions	Agger, 2014	Berman, 1987	Braun, 1971	Donders, 2009	Gerber, 2003
20) A valid, reliable method used to determine the outcome?	Unclear	Unclear	No	Yes	Yes
21) Investigators were kept 'blind' to participants' exposure to the intervention.	NA	NA	NA	NA	NA
22) Investigators were kept 'blind' to other important confounding factors.	Unclear	Unclear	Unclear	Unclear	Unclear
23) Based on above answers, was detection bias present?	Unclear	Unclear	Unclear	No	Unclear
24) If so, what is the likely direction of its effect?	Unclear	Unclear	Unclear	NA	Unclear
**25) Overall assessment of internal validity[a]**	+	-	+	+	+
**26) Overall assessment of external validity[a]**	+	+	-	+	-

Abbreviations: High, high risk of bias; Low, low risk of bias; NA, not applicable; Unclear, unclear of risk of bias;

‡, both groups combined unless stated; .[a]++, all or most of checklist criteria fulfilled; + some of checklist criteria fulfilled; - few or no checklist criteria fulfilled.

For peer review only - http://bmjopen.bmj.com/site/about/guidelines.xhtml

Supporting information

Table S7.1 Risk of bias assessment, cohort studies (n=26) (continued)

Questions	Govender, 2009	Harrison, 1983	Hillier, 1995	Kataoka, 2006	Koucky, 2016
1) The method of allocation to exposure groups was unrelated to potential confounding factors	NA	NA	NA	NA	NA
2) Attempts made within design or analysis to balance both groups for potential confounders.	Unclear	Unclear	Unclear	Yes	Unclear
3) The groups were comparable at baseline, including all major confounding factors.	Unclear	Unclear	Unclear	No	Unclear
4) Based on above answers, was selection bias present?	Unclear	Unclear	Unclear	Unclear	Unclear
5) If so, what is the likely direction of its effect?	Unclear	Unclear		Unclear	Unclear
6) The comparison groups received the same care and support apart from the exposure(s) studied.	Unclear	Yes	Yes	Yes	Yes
7) Participants receiving care and support were kept 'blind' to intervention allocation.	NA	NA	NA	NA	NA

For peer review only - http://bmjopen.bmj.com/site/about/guidelines.xhtml

Supporting information

Questions	Govender, 2009	Harrison, 1983	Hillier, 1995	Kataoka, 2006	Koucky, 2016
8) Individuals administering care and support were kept 'blind' to intervention allocation.	NA	NA	NA	NA	NA
9) Based on above answers, was performance bias present?	Unclear	Unclear	Unclear	Unclear	Unclear
10) If so, what is the likely direction of its effect?	Unclear	Unclear	Unclear	Unclear	Unclear
11) All groups followed up for an equal length of time?	Low	Low	Low	Low	Low
12) Number of participants did not complete the intervention in each group?	NA	NA	Na	NA	NA
13) The groups were comparable for intervention completion.	NA	NA	NA	NA	NA
14) For how many participants were no outcome data available?‡	0/199 (0%)	SA (13/361), 3.6%; PND (0/467, 0%)	1292/10397 (12.4%)	163/1040 (15.7%)	0/36 (0%)
15) Were groups comparable for outcome data?	Unclear	Unclear	No	Unclear	Yes
16) Based on above answers, was attrition bias present?	Unclear	Unclear	Unclear	Unclear	Unclear
17) If so, what is the likely direction of its effect?	Unclear	Unclear	Unclear	Unclear	Unclear
18) The study had an appropriate length of follow-up.	Yes	Yes	Yes	Yes	Yes

For peer review only - http://bmjopen.bmj.com/site/about/guidelines.xhtml

Supporting information

Questions	Govender, 2009	Harrison, 1983	Hillier, 1995	Kataoka, 2006	Koucky, 2016
19) The study used a precise definition of outcome.	Yes	No	Yes	Yes	Yes
20) A valid, reliable method used to determine the outcome?	Unclear	Unclear	No	Yes	Yes
21) Investigators were kept 'blind' to participants' exposure to the intervention.	NA	NA	NA	NA	NA
22) Investigators were kept 'blind' to other important confounding factors.	Unclear	Unclear	Unclear	Unclear	Unclear
23) Based on above answers, was detection bias present?	Unclear	Unclear	Unclear	No	Unclear
24) If so, what is the likely direction of its effect?	Unclear	Unclear	Unclear	NA	Unclear
**25) Overall assessment of internal validity[a]**	-	-	-	+	+
**26) Overall assessment of external validity[a]**	-	-	-	+	+

Abbreviations: High, high risk of bias; Low, low risk of bias; NA, not applicable; Unclear, unclear of risk of bias;

‡, both groups combined unless stated; [a]++, all or most of checklist criteria fulfilled; + some of checklist criteria fulfilled; - few or no checklist criteria fulfilled.

For peer review only - http://bmjopen.bmj.com/site/about/guidelines.xhtml

Supporting information

Table S7.1 Risk of bias assessment, cohort studies (n=26) (continued)

Questions	Kwak, 2014	Lee, 2016	Luton, 1994	McDonald, 1994	Menard, 2010
1) The method of allocation to exposure groups was unrelated to potential confounding factors	NA	NA	NA	NA	NA
2) Attempts made within design or analysis to balance both groups for potential confounders.	Unclear	Unclear	Yes	Yes	Unclear
3) The groups were comparable at baseline, including all major confounding factors.	Unclear	Unclear	Yes	Unclear	Unclear
4) Based on above answers, was selection bias present?	Unclear	Unclear	Low	Unclear	Unclear
5) If so, what is the likely direction of its effect?	Unclear	Unclear	Unclear	Unclear	Unclear
6) The comparison groups received the same care and support apart from the exposure(s) studied.	Unclear	Unclear	Yes	Unclear	Yes
7) Participants receiving care and support were kept 'blind' to intervention allocation.	NA	NA	NA	NA	NA
8) Individuals administering care and support were kept 'blind' to intervention allocation.	NA	NA	NA	NA	NA
9) Based on above answers, was performance bias present?	Unclear	Unclear	Unclear	Unclear	Unclear

For peer review only - http://bmjopen.bmj.com/site/about/guidelines.xhtml

Supporting information

Questions	Kwak, 2014	Lee, 2016	Luton, 1994	McDonald, 1994	Menard, 2010
10) If so, what is the likely direction of its effect?	Unclear	Unclear	Unclear	Unclear	Unclear
11) All groups followed up for an equal length of time?	Low	Low	Low	Low	Low
12) Number of participants did not complete the intervention in each group?	NA	NA	NA	NA	NA
13) The groups were comparable for intervention completion.	NA	NA	NA	NA	NA
14) For how many participants were no outcome data available?‡	0 (0%)	0/1035 (0%)	37/218 (17%)	Control 182/649, (28%); Cases 42/135 (31%)	0 (0%)
15) Were groups comparable for outcome data?	Yes	Yes	No	Unclear	Yes
16) Based on above answers, was attrition bias present?	Low	Low	Unclear	Unclear	Unclear
17) If so, what is the likely direction of its effect?	Unclear	Unclear	Unclear	Unclear	Unclear
18) The study had an appropriate length of follow-up.	Yes	Yes	Yes	Yes	Yes
19) The study used a precise definition of outcome.	Yes	No	Yes	Yes	Yes
20) A valid, reliable method used to determine the outcome?	Yes	No	Yes	No	Yes

For peer review only - http://bmjopen.bmj.com/site/about/guidelines.xhtml

Supporting information

Questions	Kwak, 2014	Lee, 2016	Luton, 1994	McDonald, 1994	Menard, 2010
21) Investigators were kept ‘blind’ to participants’ exposure to the intervention.	NA	NA	NA	Unclear	NA
22) Investigators were kept ‘blind’ to other important confounding factors.	Unclear	Unclear	Unclear	Unclear	NA
23) Based on above answers, was detection bias present?	No	Yes	Unclear	Unclear	No
24) If so, what is the likely direction of its effect?	NA	Unclear	Unclear	Unclear	NA
**25) Overall assessment of internal validity[a]**	+	-	-	-	-
**26) Overall assessment of external validity[a]**	+	-	-	-	-

Abbreviations: High, high risk of bias; Low, low risk of bias; NA, not applicable;Unclear, unclear of risk of bias.

‡, both groups combined unless stated; [a]++, all or most of checklist criteria fulfilled; + some of checklist criteria fulfilled; - few or no checklist criteria fulfilled.

For peer review only - http://bmjopen.bmj.com/site/about/guidelines.xhtml

Supporting information

Table S7.1 Risk of bias assessment, cohort studies (n=26) (continued)

Questions	Minkoff, 1984	Nguyen, 2004	Odendaal, 2002	Payne, 2016	Payne, 2020
1) The method of allocation to exposure groups was unrelated to potential confounding factors	NA	NA	NA	NA	NA
2) Attempts made within design or analysis to balance both groups for potential confounders.	Unclear	Unclear	Unclear	Unclear	No
3) The groups were comparable at baseline, including all major confounding factors.	Unclear	Unclear	Unclear	Unclear	Unclear
4) Based on above answers, was selection bias present?	Unclear	Unclear	Unclear	Unclear	Low
5) If so, what is the likely direction of its effect?	Unclear	Unclear	Unclear	Unclear	Unclear
6) The comparison groups received the same care and support apart from the exposure(s) studied.	Unclear	Unclear	Yes	Unclear	Yes
7) Participants receiving care and support were kept 'blind' to intervention allocation.	NA	NA	NA	NA	NA

For peer review only - http://bmjopen.bmj.com/site/about/guidelines.xhtml

Supporting information

Questions	Minkoff, 1984	Nguyen, 2004	Odendaal, 2002	Payne, 2016	Payne, 2020
8) Individuals administering care and support were kept 'blind' to intervention allocation.	NA	NA	NA	NA	NA
9) Based on above answers, was performance bias present?	Unclear	Unclear	Unclear	Unclear	Unclear
10) If so, what is the likely direction of its effect?	Unclear	Unclear	Unclear	Unclear	Unclear
11) All groups followed up for an equal length of time?	Low	Low	Low	Low	Low
12) Number of participants did not complete the intervention in each group?	NA	NA	NA	NA	NA
13) The groups were comparable for intervention completion.	NA	NA	NA	NA	NA
14) For how many participants were no outcome data available?‡	PROM 45/233 (19.3%); PTB 15/233 (6.4%)	61/456 (13.4%)	31/426 (7.3%)	15/206 (7.3%)	6.4% (64/1000)
15) Were groups comparable for outcome data?	Unclear	Yes	Yes	Yes	Unclear
16) Based on above answers, was attrition bias present?	Unclear	Low	Unclear	Unclear	Low
17) If so, what is the likely direction of its effect?	Unclear	Unclear	Unclear	Unclear	Unclear

For peer review only - http://bmjopen.bmj.com/site/about/guidelines.xhtml

Supporting information

Questions	Minkoff, 1984	Nguyen, 2004	Odendaal, 2002	Payne, 2016	Payne, 2020
18) The study had an appropriate length of follow-up.	Yes	Yes	Yes	Yes	Yes
19) The study used a precise definition of outcome.	Yes	Yes	Yes	Yes	Yes
20) A valid, reliable method used to determine the outcome?	Yes	Unclear	Yes	Unclear	Unclear
21) Investigators were kept 'blind' to participants' exposure to the intervention.	NA	NA	NA	NA	NA
22) Investigators were kept 'blind' to other important confounding factors.	Unclear	Unclear	Unclear	Unclear	Unclear
23) Based on above answers, was detection bias present?	No	Unclear	Unclear	Unclear	No
24) If so, what is the likely direction of its effect?	NA	Unclear	Unclear	Unclear	NA
**25) Overall assessment of internal validity[a]**	-	+	+	+	+
**26) Overall assessment of external validity[a]**	-	+	-	+	+

Abbreviations: AO, adverse outcomes; High, high risk of bias; Low, low risk of bias; NA, not applicable; NK, not known; STI, sexually transmitted infections; Unclear, unclear of risk of bias;

‡, both groups combined unless stated; [a]++, all or most of checklist criteria fulfilled; + some of checklist criteria fulfilled; - few or no checklist criteria fulfilled.

For peer review only - http://bmjopen.bmj.com/site/about/guidelines.xhtml

Supporting information

Table S7.1 Risk of bias assessment, cohort studies (n=26) (continued)

Questions	Peretz, 2020	Perni, 2004	Rittenschober, 2018	Schwab, 2015	Sperling, 1988	Usui, 2002
1) The method of allocation to exposure groups was unrelated to potential confounding factors	NA	NA	NA	NA	NA	NA
2) Attempts made within design or analysis to balance both groups for potential confounders.	Yes	Unclear	Yes	No	Unclear	Unclear
3) The groups were comparable at baseline, including all major confounding factors.	Unclear	Unclear	Unclear	Unclear	Unclear	Unclear
4) Based on above answers, was selection bias present?	Unclear	Unclear	Low	High	Unclear	Unclear
5) If so, what is the likely direction of its effect?	Unclear	Unclear	Unclear	Unclear	Unclear	Unclear
6) The comparison groups received the same care and support apart from the exposure(s) studied.	Yes	Unclear	Unclear	Unclear	Yes	Yes
7) Participants receiving care and support were kept 'blind' to intervention allocation.	NA	NA	NA	NA	NA	NA

For peer review only - http://bmjopen.bmj.com/site/about/guidelines.xhtml

Supporting information

Questions	Peretz, 2020	Perni, 2004	Rittenschober, 2018	Schwab, 2015	Sperling, 1988	Usui, 2002
8) Individuals administering care and support were kept 'blind' to intervention allocation.	NA	NA	NA	NA	NA	NA
9) Based on above answers, was performance bias present?	Unclear	Unclear	Unclear	Unclear	Unclear	Low
10) If so, what is the likely direction of its effect?	Unclear	Unclear	Unclear	Unclear	Unclear	NA
11) All groups followed up for an equal length of time?	Low	Low	Low	Low	Low	Low
12) Number of participants did not complete the intervention in each group?	NA	NA	NA	NA	NA	NA
13) The groups were comparable for intervention completion.	NA	NA	NA	NA	NA	NA
14) For how many participants were no outcome data available?‡	PTB 91% (195/214); LBW 90% (192/214)	14/193 (7.3%)	687/4330; (15.9%)	97/159 (61.0%)	1.2% (5/409)	0,0%
15) Were groups comparable for outcome data?	No	Unclear	Unclear	Unclear	Yes	Yes
16) Based on above answers, was attrition bias present?	High	Unclear	Unclear	High	Low	Low
17) If so, what is the likely direction of its effect?	Unclear	Unclear	Unclear	Unclear	NA	NA
18) The study had an appropriate length of follow-up.	Yes	Yes	Yes	Yes	Yes	Yes

For peer review only - http://bmjopen.bmj.com/site/about/guidelines.xhtml

Supporting information

Questions	Peretz, 2020	Perni, 2004	Rittenschober, 2018	Schwab, 2015	Sperling, 1988	Usui, 2002
19) The study used a precise definition of outcome.	Yes	Yes	Yes	Yes	Yes	Yes
20) A valid, reliable method used to determine the outcome?	Yes	Unclear	Yes	No	No	Yes
21) Investigators were kept 'blind' to participants' exposure to the intervention.	NA	NA	NA	NA	NA	NA
22) Investigators were kept 'blind' to other important confounding factors.	NA	Unclear	Unclear	Unclear	Unclear	Unclear
23) Based on above answers, was detection bias present?	No	Unclear	No	Yes	Unclear	Unclear
24) If so, what is the likely direction of its effect?	NA	Unclear	NA	Unclear	Unclear	Unclear
**25) Overall assessment of internal validity**[a]	-	+	+	-	-	+
**26) Overall assessment of external validity**[a]	-	+	+	-	-	-

Abbreviations: AO, adverse outcomes; High, high risk of bias; Low, low risk of bias; NA, not applicable; NK, not known; STI, sexually transmitted infections; Unclear, unclear of risk of bias.

‡, both groups combined unless stated; [a]++, all or most of checklist criteria fulfilled; + some of checklist criteria fulfilled; - few or no checklist criteria fulfilled.

For peer review only - http://bmjopen.bmj.com/site/about/guidelines.xhtml

Supporting information

Table S7.2 Risk of bias assessment, case-control studies (n=25)

Questions	Ahmadi, 2014	Cassell, 1993	Chua, 1999	Daskalakis, 2009	Embree, 1980	Farhadifar, 2016	Freitas, 2018
1) Appropriate and clearly focused question.	WC	WC	WC	WC	AA	WC	AA
2) The cases and controls are taken from comparable populations.	AA	WC	WC	AA	PA	AA	PA
3) The same exclusion criteria are used for both cases and controls.	WC	NA	PA	WC	NAd	AA	NAd
4) What was the participation rate (%) for each group (cases)?	Unclear	87.9 (29/33)	Unclear	Unclear	100% (n=446)	Unclear	100% (n=46)
5) What was the participation rate (%) for each group (controls)?	Unclear	100 (28/28)	Unclear	Unclear	100% (n=108)	Unclear	100% (n=170)
6) Both groups compared to establish their similarities or differences.	NAd	NAd	NAd	NAd	NAd	NAd	AA
7) Cases are clearly defined and differentiated from controls.	WC	AA	AA	WC	AA	WC	AA

For peer review only - http://bmjopen.bmj.com/site/about/guidelines.xhtml

Supporting information

Questions	Ahmadi, 2014	Cassell, 1993	Chua, 1999	Daskalakis, 2009	Embree, 1980	Farhadifar, 2016	Freitas, 2018
8) It is clearly established that controls are not cases.	WC	AA	AA	WC	AA	WC	AA
9) Measures taken to prevent knowledge of primary exposure from influencing case ascertainment.	NA	NA	NA	NA	NA	NA	NA
10) Exposure status is measured in a standard, valid and reliable way.	WC	AA	AA	WC	AA	AA	WC
11) Main potential confounders are accounted for in design/analysis	AA	PA	NR	AA	PA	AA	PA
12) Confidence intervals provided?	No	No	No	No	No	Yes	No
**13) Study results internally valid**[a]	+	+	+	+	-	+	+
**14) Study results externally valid**[a]	+	-	+	+	-	+	-

Abbreviations: AA, adequately addressed; NAd, not addressed; NA, not applicable; PA, poorly addressed; WC, well covered; Unclear, does not have data on how many were excluded or declined to participate but only present numbers

[a]++, all or most of checklist criteria fulfilled; + some of checklist criteria fulfilled; - few or no checklist criteria fulfilled.

For peer review only - http://bmjopen.bmj.com/site/about/guidelines.xhtml

Supporting information

Table S7.2 Risk of bias assessment, case-control studies (n=25) (continued)

Questions	Gonzàlez Bosquet, 2006	Harada, 2008	Hillier, 1988	Holst, 1994	Jalava, 2002	Jones, 2009
1) Appropriate and clearly focused question.	WC	WC	WC	WC	NR	WC
2) The cases and controls are taken from comparable populations.	AA	AA	AA	PA	AA	PA
3) The same exclusion criteria are used for both cases and controls.	AA	AA	PA	AA	PA	NAd
4) What was the participation rate for each group (cases)? %	Unclear	Unclear	99/107 (92.5%)	40.8 (49/120)	100 (n=50)	Unclear
5) What was the participation rate for each group (controls)? %	Unclear	Unclear	68/140 (48.6%)	100 (38/38)	72 (72/100)	Unclear
6) Both groups compared to establish their similarities or differences.	NAd	NAd	NAd	NAd	NAd	NAd
7) Cases are clearly defined and differentiated from controls.	WC	AA	WC	WC	WC	AA
8) It is clearly established that controls are not cases.	WC	AA	WC	WC	AA	AA
9) Measures taken to prevent knowledge of primary exposure from influencing case ascertainment.	NA	NA	NA	NA	NA	NA

For peer review only - http://bmjopen.bmj.com/site/about/guidelines.xhtml

Supporting information

Questions	Gonzàlez Bosquet, 2006	Harada, 2008	Hillier, 1988	Holst, 1994	Jalava, 2002	Jones, 2009
10) Exposure status is measured in a standard, valid, and reliable way.	AA	WC	AA	AA	WC	WC
11) Main potential confounders are accounted for in design/analysis	PA	PA	WC	AA	NAd	NAd
12) Confidence intervals provided?	No	Yes	Yes	No	No	No
**13) Study results internally valid[a]**	+	+	+	++	+	-
**14) Study results externally valid[a]**	+	+	+	+	+	-

Abbreviations: AA, adequately addressed; NAd, not addressed; NA, not applicable; PA, poorly addressed; WC, well covered; Unclear, does not have data on how many were excluded or declined to participate but only present numbers.

[a] ++, all or most of checklist criteria fulfilled; + some of checklist criteria fulfilled; - few or no checklist criteria fulfilled.

For peer review only - http://bmjopen.bmj.com/site/about/guidelines.xhtml

Supporting information

Table S7.2 Risk of bias assessment, case-control studies (n=25) (continued)

Questions	Kacerovsky, 2009	Kafetzis, 2004	Kumar, 2006	McDonald, 1992	Matsunari, 2005	Montenegro, 2019
1) Appropriate and clearly focused question.	WC	WC	AA	AA	WC	WC
2) The cases and controls are taken from comparable populations.	PA	WC	PA	AA	WC	AA
3) The same exclusion criteria are used for both cases and controls.	PA	NR	NAd	PA	WC	AA
4) What was the participation rate for each group (cases)?	Unclear	Unclear	100% (n=60)	Unclear	57.5 (23/40)	84 (100%)
5) What was the participation rate for each group (controls)?	Unclear	Unclear	100% (n=60)	Unclear	60.8 (59/97)	127 (1000%)
6) Both groups compared to establish their similarities or differences.	NAd	NAd	NAd	AA	NA	NA
7) Cases are clearly defined and differentiated from controls.	WC	WC	NAd	AA	AA	AA
8) It is clearly established that controls are not cases.	PA	WC	AA	AA	AA	AA
9) Measures taken to prevent knowledge of primary exposure from influencing case ascertainment.	NA	NA	NA	NA	NA	NA

For peer review only - http://bmjopen.bmj.com/site/about/guidelines.xhtml

Supporting information

Questions	Kacerovsky, 2009	Kafetzis, 2004	Kumar, 2006	McDonald, 1992	Matsunari, 2005	Montenegro, 2019
10) Exposure status is measured in a standard, valid, and reliable way.	AA	WC	PA	PA	WC	WC
11) Main potential confounders are accounted for in design/analysis	PA	NAd	NAd	AA	PA	PA
12) Confidence intervals provided?	No	No	No	Yes	No	No
**13) Study results internally valid[a]**	-	+	-	+	+	+
**14) Study results externally valid[a]**	-	+	-	+	+	+

Abbreviations: AA, adequately addressed; NAd, not addressed; NA, not applicable; PA, poorly addressed; WC, well covered; Unclear, does not have data on how many were excluded or declined to participate but only present numbers

[a]++, all or most of checklist criteria fulfilled; + some of checklist criteria fulfilled; - few or no checklist criteria fulfilled.

For peer review only - http://bmjopen.bmj.com/site/about/guidelines.xhtml

Supporting information

Table S7.2 Risk of bias assessment, case-control studies (n=25) (continued)

Questions	Munday, 1984	Oliveira, 2020	Payne, 2014	Povlsen, 2001	Toth, 1992	Yoon, 2001
1) Appropriate and clearly focused question.	WC	AA	WC	WC	WC	WC
2) The cases and controls are taken from comparable populations.	AA	PA	WC	WC	AA	AA
3) The same exclusion criteria are used for both cases and controls.	NAd	PA	AA	NAd	PA	PA
4) What was the participation rate for each group (cases)?	Unclear	100%	100%	Unclear	Unclear	Unclear
5) What was the participation rate for each group (controls)?	Unclear	100%	100%	Unclear	Unclear	Unclear
6) Both groups compared to establish their similarities or differences.	NA	AA	NAd	NA	NA	NA
7) Cases are clearly defined and differentiated from controls.	PA	WC	AA	AA	PA	WC
8) It is clearly established that controls are not cases.	PA	WC	PA	PA	PA	WC
9) Measures taken to prevent knowledge of primary exposure from influencing case ascertainment.	NA	NA	NAd	NA	NA	NA
10) Exposure status is measured in a standard, valid, and reliable way.	AA	AA	AA	AA	PA	WC
11) Main potential confounders are accounted for in design/analysis	PA	AA	NAd	NAd	PA	AA
12) Confidence intervals provided?	No	Yes	Yes	Yes	No	No

For peer review only - http://bmjopen.bmj.com/site/about/guidelines.xhtml

Supporting information

Questions	Munday, 1984	Oliveira, 2020	Payne, 2014	Povlsen, 2001	Toth, 1992	Yoon, 2001
**13) Study results internally valid**[a]	+	+	+	+	-	+
**14) Study results externally valid**[a]	-	+	+	+	-	+

Abbreviations: AA, adequately addressed; NAd, not addressed; NA, not applicable; PA, poorly addressed; Unclear, does not have data on how many were excluded or declined to participate but only present numbers; WC, well covered.

[a]++, all or most of checklist criteria fulfilled; + some of checklist criteria fulfilled; - few or no checklist criteria fulfilled.

For peer review only - http://bmjopen.bmj.com/site/about/guidelines.xhtml

Supporting information

Table S7.3 Risk of bias assessment, cross-sectional studies (n=6)

Questions	Abele-Horn, 2000	Grattard, 1995	Kundsin, 1984	McCormack, 1975	Nasution, 2007	Sweeney, 2016
1) Is the source population, source area well described?	+	+	NR	-	NR	+
2) Is the eligible population or area representative of the source population?	-	+	NR	-	NR	-
3) Do the selected participants or areas represent the eligible population or area?	-	NR	-	-	NR	-
4) Selection of exposure (and comparison) group. How was selection bias minimised?	NR	NR	NR	NR	NR	NR
5) Was the selection of explanatory variables based on a sound theoretical basis?	+	-	+	-	+	+
6) Was the contamination acceptably low?	NA	NA	NA	NA	NR	NA
7) How well were likely confounding factors identified and controlled?	+	NR	NR	NR	NR	-
8) Outcome measures and procedures reliable?	+	-	+	-	+	-
9) Were the outcome measurements complete?	+	+	+	+	++	+

For peer review only - http://bmjopen.bmj.com/site/about/guidelines.xhtml

Supporting information

10) Were all the important outcomes assessed?	-	+	+	+	+	++
11) Was there a similar follow-up time in exposure and comparison groups?	+	-	++	+	++	+
12) Was follow-up time meaningful?	+	+	++	+	++	+
13) Was the study sufficiently powered to detect an exposure effect (if one exists)	NA	NA	NA	NA	NR	NA
14) Were multiple explanatory variables considered in analyses?	NR	NR	NR	NR	NR	NR
15) Were the analytical methods appropriate?	+	-	-	+	-	+
16) Was the precision of association given or calculable?	+	+	+	+	+	+
**17) Overall assessment of internal validity[a]**	**-**	**-**	**-**	**+**	**-**	**+**
**18) Overall assessment of external validity[a]**	**-**	**+**	**+**	**-**	**-**	**-**

Abbreviations: ++, yes; +, mostly; -, no; NR, not reported; NA, not applicable.

[a] ++, all or most of checklist criteria fulfilled; + some of checklist criteria fulfilled; - few or no checklist criteria fulfilled.

For peer review only - http://bmjopen.bmj.com/site/about/guidelines.xhtml

BMJ Open

Adverse pregnancy and birth outcomes associated with Mycoplasma hominis, Ureaplasma urealyticum and Ureaplasma parvum: A systematic review and meta-analysis

Journal:	*BMJ Open*
Manuscript ID	bmjopen-2022-062990.R1
Article Type:	Original research
Date Submitted by the Author:	25-May-2022
Complete List of Authors:	Jonduo, Marinjho; University of New South Wales Faculty of Medicine, Kirby Institute<br>Vallely, Lisa; University of New South Wales Faculty of Medicine, Kirby Institute<br>Wand, Handan; University of New South Wales Faculty of Medicine, Kirby Institute<br>Sweeney, Emma; The University of Queensland Centre for Clinical Research<br>Egli-Gany, Dianne; Universitat Bern Institut fur Sozial- und Praventivmedizin,<br>Kaldor, John; University of New South Wales Faculty of Medicine, Kirby Institute<br>Vallely, Andrew; University of New South Wales Faculty of Medicine, Kirby Institute,; Papua New Guinea Institute of Medical Research, Sexual and Reproductive Health Unit<br>Low, Nicola; University of Bern, Institute of Social and Preventive Medicine
**Primary Subject Heading**:	Obstetrics and gynaecology
Secondary Subject Heading:	Sexual health, Public health, Infectious diseases, Epidemiology
Keywords:	GYNAECOLOGY, MICROBIOLOGY, OBSTETRICS, EPIDEMIOLOGY, Epidemiology < INFECTIOUS DISEASES

SCHOLARONE™
Manuscripts

I, the Submitting Author has the right to grant and does grant on behalf of all authors of the Work (as defined in the below author licence), an exclusive licence and/or a non-exclusive licence for contributions from authors who are: i) UK Crown employees; ii) where BMJ has agreed a CC-BY licence shall apply, and/or iii) in accordance with the terms applicable for US Federal Government officers or employees acting as part of their official duties; on a worldwide, perpetual, irrevocable, royalty-free basis to BMJ Publishing Group Ltd ("BMJ") its licensees and where the relevant Journal is co-owned by BMJ to the co-owners of the Journal, to publish the Work in this journal and any other BMJ products and to exploit all rights, as set out in our licence.

The Submitting Author accepts and understands that any supply made under these terms is made by BMJ to the Submitting Author unless you are acting as an employee on behalf of your employer or a postgraduate student of an affiliated institution which is paying any applicable article publishing charge ("APC") for Open Access articles. Where the Submitting Author wishes to make the Work available on an Open Access basis (and intends to pay the relevant APC), the terms of reuse of such Open Access shall be governed by a Creative Commons licence – details of these licences and which Creative Commons licence will apply to this Work are set out in our licence referred to above.

Other than as permitted in any relevant BMJ Author's Self Archiving Policies, I confirm this Work has not been accepted for publication elsewhere, is not being considered for publication elsewhere and does not duplicate material already published. I confirm all authors consent to publication of this Work and authorise the granting of this licence.

For peer review only

Adverse pregnancy and birth outcomes associated with *Mycoplasma hominis, Ureaplasma urealyticum* and *Ureaplasma parvum*: A systematic review and meta-analysis

Corresponding author: Prof. N Low, MD MSc FFPH, Institute of Social and Preventive Medicine, Mittelstrasse 43, University of Bern, CH-3012 Bern, Switzerland. Tel: +41 30 684 30 92; Email: nicola.low@ispm.unibe.ch. ORCiD 0000-0003-4817-8986

Marinjho Emely Jonduo,[1] Lisa Michelle Vallely,[1,2] Handan Wand,[3] Emma Louise Sweeney,[4] Dianne Egli-Gany,[5] John Martin Kaldor,[1] Andrew John Vallely[1,2] and Nicola Low,[5]

[1] Public Health Interventions Research Program, The Kirby Institute, University of New South Wales, Sydney, NSW 2052, Australia.

[2] Sexual and Reproductive Health Unit, Papua New Guinea Institute of Medical Research, Goroka, Eastern Highlands Province 441, Papua New Guinea.

[3] Biostatistics and Databases program, The Kirby Institute, University of New South Wales, Sydney, New South Wales, Australia.

[4] The University of Queensland Center for Clinical Research, Brisbane, Queensland 4029, Australia.

[5] Institute of Social and Preventive Medicine, University of Bern, CH-3012 Bern, Switzerland.

Conflict of interest: The authors report no conflict of interest.

Study funding: Australian National Health & Medical Research Council (NHMRC); DFID/MRC/Wellcome Trust Joint Global Health Trials; Swiss National Science Foundation.

For peer review only

ABSTRACT

Objectives

Mycoplasma hominis, Ureaplasma urealyticum and *Ureaplasma parvum* (genital mycoplasmas) commonly colonise the urogenital tract in pregnant women. This systematic review aims to investigate their role in adverse pregnancy and birth outcomes, alone or in combination with bacterial vaginosis (BV).

Methods: We searched Embase, Medline and CINAHL databases from January 1971 to February 2021. Eligible studies tested for any of the three genital mycoplasmas during pregnancy and reported on the primary outcome, preterm birth (PTB) and/or secondary outcomes low birth weight (LBW), premature rupture of membranes (PROM), spontaneous abortion (SA) and/or perinatal death (PND).

Two reviewers independently screened titles and abstracts, read potentially eligible full texts and extracted data. Two reviewers independently assessed risks of bias using published checklists. Random effects meta-analysis was used to estimate summary odds ratios (OR, with 95% confidence intervals, and prediction intervals). Multivariable and stratified analyses were synthesised descriptively.

Results

Of 57/1194 included studies, 39 were from high-income countries. In meta-analysis of unadjusted ORs, *M. hominis* was associated with PTB (OR 1.87, 95% CI 1.49, 2.34), PROM, LBW and PND but not SA. *U. urealyticum* was associated with PTB (OR 1.84, 95% CI 1.34, 2.55), PROM, LBW, SA and PND. *U. parvum* was associated with PTB (1.60, 95% CI 1.12, 2.30), PROM and SA. Nine of 57 studies reported any multivariable analysis. In two studies, analyses stratified by BV status showed that *M. hominis* and *U. parvum* were more strongly associated with PTB in the presence than in the absence of BV. The most frequent source of bias was a failure to control for confounding.

For peer review only

Conclusions

The currently available literature does not allow conclusions about the role of mycoplasmas in adverse pregnancy and birth outcomes, alone or with co-existing BV. Future studies that consider genital mycoplasmas in the context of the vaginal microbiome are needed.

PROSPERO published date: 01 Nov 2018; **registration number:** CRD42016050962

Strengths and limitations

- We followed a published protocol with predefined outcomes and statistical analysis plan
- Two reviewers independently selected the studies, extracted data and performed risk of bias assessment
- Evidence for heterogeneity was examined and described both visually and statistically
- We triangulated findings across study designs
- Restriction to studies in English and German might have missed eligible articles.

For peer review only

INTRODUCTION

Mycoplasma hominis, Ureaplasma parvum and *Ureaplasma urealyticum*, referred to together as genital mycoplasmas, commonly colonise the urogenital tract in women, and are often found together.[1, 2] These species do not appear to cause symptoms or harmful effects in nonpregnant women.[2, 3] Plummer et al. found that *M. hominis* was associated with abnormal vaginal discharge only in nonpregnant women who also had bacterial vaginosis (BV).[2] Colonisation with a genital mycoplasma has, however, been reported in many studies to be associated with several adverse pregnancy outcomes[4, 5] including preterm birth (PTB); low birth weight (LBW); premature rupture of membranes (PROM) and preterm premature rupture of the membranes (PPROM), spontaneous abortion (SA), and perinatal death (PND).[1, 6-12] Several research groups have suggested that *M. hominis*, whilst considered a part of the normal vaginal microbiota, might only be pathogenic in the presence of BV as part of a disturbed vaginal microbiota.[4, 5, 13] There are, however, inconsistencies across studies, uncertainty about the interplay between specific organisms and the vaginal microbiota in general,[14-16] and differences in recommendations for testing and treatment.[13, 17]

Technological advances in the molecular detection of multiple vaginal and endocervical organisms in the same assay[18, 19] should make it easier to study the role of genital mycoplasmas in adverse pregnancy outcomes. Methods to distinguish between *U. urealyticum* and *U. parvum* were not widely available before 2000,[20, 21] and unspeciated *Ureaplasma* spp. detected by culture were reported together as *U. urealyticum*.[18] Narrative reviews have not fully elucidated whether the apparent pathogenicity of genital mycoplasmas in pregnancy is associated with a particular organism, concurrent infection with multiple genital mycoplasmas and other lower genital tract organisms, or confounding by other demographic, clinical and behavioural factors.[4, 5, 13] A systematic and quantitative assessment of these questions is therefore timely.

For peer review only

OBJECTIVES

The primary objective of this study was to investigate the associations between *M. hominis*, *U. urealyticum* and/or *U. parvum* and the risk of PTB, alone and in combination with BV. Secondary objectives were to investigate associations between each genital mycoplasma and LBW, PROM, SA and PND.

METHODS

This systematic review followed a registered protocol (PROSPERO CRD42016050962),[22] which covers multiple organisms, for which findings are reported elsewhere, including *Neisseria gonorrhoeae*[23] and *M. genitalium.*[24] We report our findings using the Preferred Reporting Items for Systematic Reviews and Meta-Analyses (PRISMA 2020) (A.1)[25] and methodological guidance about systematic reviews of observational studies (MOOSE) (A.2).[26] Patients or the public were not involved in the design, or conduct, or reporting, or dissemination plans of our research.

Eligibility criteria, information sources and search strategy

Studies were eligible if they reported on pregnant women with and without *M. hominis*, *U. urealyticum* and/or *U. parvum* and included one or more of the outcomes: PTB, LBW, PROM (preterm or term), SA and PND. Standard definitions were used for all outcomes (PTB, delivery at <37 weeks gestation; LBW, birthweight <2.5kg; PROM, rupture of membranes prior to onset of labour; PPROM, premature rupture at <37 weeks gestation; SA, delivery at <20 weeks gestation; stillbirth (death after >20 weeks in utero); perinatal or neonatal death (PND, stillbirths and death <28 days after birth), but we used author's definitions if necessary.[22] We excluded articles published before 2000 if they reported unspeciated *U. urealyticum* alone. If they reported on *M. hominis* and *U. urealyticum* we included the study but did not extract results about

For peer review only

U. urealyticum. We included cohort, cross-sectional and case-control studies, and randomised controlled trials.

A member of the team (MJ) searched Medline, Embase, Cumulative Index to Nursing and Allied Health Literature (CINAHL) for literature published from January 1971 to February 2021. We searched reference lists of included studies for additional potentially eligible studies but did not search grey literature sources. The searches did not include language restrictions, but we only read the full-text of articles in English and German (languages spoken by the review team). The full search strategy is in the online supporting information (A.3). We used Endnote (V7, Thomson Reuters) to import, de-duplicate and manage retrieved records.

Study selection and data extraction

Two reviewers (MJ, LV) independently screened titles and abstracts, and read the full text of potentially eligible papers. Disparities were resolved by discussion or by a third reviewer (DEG). Where multiple reports presented data from the same study population, we identified a primary record with the most detailed information but included data from other publications. Two reviewers (MJ, LV) extracted data independently into an online database (Research Electronic Data Capture, REDCap, Vanderbilt University, Tennessee). Disparities were resolved by discussion or by a third reviewer (DEG, NL or ES).

Data extraction

Each reviewer extracted data about the study design, study setting and sociodemographic characteristics, specimen type and timing, laboratory tests, organisms tested for, outcomes reported, raw numbers of participants with and without each outcome and organism, where available, or author reported effect size and 95% confidence intervals (CI). They extracted the adjusted odds ratio (aOR, 95% CI) and recorded variables included in multivariable models, where possible. If results were described for more than one anatomical site, we used the

For peer review only

For peer review only - http://bmjopen.bmj.com/site/about/guidelines.xhtml

following order of preference: vaginal or cervical swabs, amniotic fluid, placenta, urine, blood. Where more than one diagnostic method, we used data from nucleic acid amplification test (NAAT), then bacterial culture, followed by ELISA. The data underlying this article are available in the article and in its online supplementary material.

Risk of bias assessments

Two reviewers (MJ, LV) appraised each article independently, using checklists published by the UK National Institute for Health and Care Excellence (NICE).[27, 28] A qualitative judgement about internal and external validity was summarised as: all or most checklist criteria fulfilled (++), some criteria fulfilled (+), or few or no criteria fulfilled (-). We used funnel plots and the Egger test[29] to investigate evidence for publication or small study biases across studies for outcomes reported by more than nine studies.

Data synthesis

We used Stata 14.0 (StataCorp, College Station, TX) for all analyses. We used the OR, with 95% CI as the measure of association for all study designs, since the OR and risk ratio are similar for rare outcomes, as is the case for most of the outcomes of interest. This allowed us to analyse findings from different study designs together, where appropriate.[30] We constructed 2x2 tables to calculate of the OR or used the authors' calculation when raw data were unavailable. We added 0.5 to each cell in the table if there were zero observations in any cell. For each exposure-outcome pair, we examined forest plots of univariable associations visually, displaying the OR (with 95% CI) and the I^2 statistic, to examine between study heterogeneity. We used a random effects model to estimate a summary OR (95% CI), which is the average effect across all included studies.[31] We stratified studies by study design in forest plots and, where the stratified estimates were compatible, we estimated the overall estimated OR with its 95% CI and a prediction interval, where there were three or more studies. The prediction interval takes into account all sources of between study variability to estimate a range of values- for the OR in a new

For peer review only

study that is similar to the types of study included in the meta-analysis.[31] We then examined evidence for from studies that also reported on BV. We described findings from analyses that were stratified by BV status, or in studies with a multivariable analysis, we reported the aOR, controlling for BV and other measured confounding variables.[26]

RESULTS

Study selection

Our searches identified 1194 records and we screened 641, after exclusion of duplicates (Figure S1). Of 215 full-text articles, we included 57 studies. Articles excluded based on title and abstract mostly concerned neonatal respiratory outcomes, chorioamnionitis and infertility. Exclusion of full-text articles had various reasons (Figure S1).

Study characteristics

Of the 57 studies, we identified 42 reporting on *M. hominis* (proportion detected <1-70%), 31 reporting on *U. urealyticum* (proportion detected 0-91%) and 12 reporting on *U. parvum* (2–100%) and median sample size 250, interquartile range, IQR 145-613, range 37 [32] to 9105 [33] (Table 1, Supporting information Table S1). There were 26 cohort studies (Table S2.1),[1, 6, 8, 12, 15, 33-53] 25 case-control studies (Table S2.2)[7, 9-11, 32, 54-73] and six cross-sectional studies (Table S2.3).[74-79] Most studies were from high-income settings (39/57) (Table S3.1, S3.2, S3.3); ethnicity was reported in 24 studies, and maternal smoking in 12 (Table S4.1, S4.2, S4.3). Most studies (54/57) stated the timing of specimen collection, and all described the laboratory tests used (Table S1): 29/57 bacterial culture only; 24/57 NAAT only (Table 1, Table S1). Three studies reported on antimicrobial susceptibilities[8, 50] with *M. hominis* resistant to erythromycin, clarithromycin, tetracycline and *U. urealyticum* resistant to ciprofloxacin, tetracycline and erythromycin.[6, 50]

For peer review only

Table 1. Summary of characteristics of studies included in the systematic review

Characteristic	Total	*M. hominis*	*U. urealyticum*	*U. parvum*
Number of studies, n[*]	57	42	31	12
Study design, n				
Cohort	26	23	16	9
Case-control	25	13	12	1
Cross-sectional	6	6	3	2
Number of women, total	36,992	28,697	16,609	9,663
(median; IQR)	(250; 145-613)	(250; 159-759)	(216; 145-613)	(376; 195-986)
Study setting, income category, n				
High income	38	27	20	10
Upper-middle income	9	8	4	2
Lower middle-income or low	3	2	1	0
Not reported	11	5	6	0
Outcomes reported, n				
Preterm birth	43	29	27	11
Low birth weight	8	6	2	1
Premature ruptures of membrane	15	11	11	2
Spontaneous abortion	11	10	4	2
Perinatal death	11	10	2	1
Specimen type, n[†]				
Endocervical swab	24	18	12	4

For peer review only

Vaginal swab	15	10	11	5
Urine	1	1	0	0
Amnotic fluid	9	6	5	2
Placental membrane	8	7	3	2
Diagnostic method*				
NAAT	24	13	20	10
Culture	29	27	7	0
Culture and NAAT	3	1	3	2
Other**	1	1	0	0
Bacterial vaginosis assessed, n	10	8	3	1
Reported presence of STI, n	20	14	8	3
Reported on smoking status, n	13	7	6	4
Reported on Multiple pregnancy, n*‡				
Excluded	26	18	15	6
Included	8	5	4	3

184 **Abbreviations**: IQR, interquartile range; STI, sexually transmitted infection

185 * The total number of studies included is 57. The totals for each organism sum to more than 57 because one study might have reported on more than
186 one organism;

187 † One study used both urine and endocervical swab;

188 **ELISA (with NAAT/ Culture)

189 ‡ 22 studies included women with multiple pregnancy

190

For peer review only

Of the 57 studies, 37 reported on a single microorganism (*M. hominis*, n=27; *U. urealyticum*, n=10); 13 included two genital mycoplasmas (*M. hominis* and *U. urealyticum*, n=9; ureaplasmas, n=4) and seven reported on all three organisms (Figure S2). Only two studies presented findings for combinations of more than one genital mycoplasma;[6, 47] the rest presented data separately, even if they had tested for more than one organism. Ten studies reported on the presence of BV;[33, 36, 43, 47, 51, 53, 58, 59, 65, 72] we report the findings of these studies in the relevant section of the results for each genital mycoplasma. Twenty-three studies reported on other sexually transmitted infections (Table S4.1, S4.3, S4.3), including 2/23 reporting on syphilis, 5/23 gonorrhoea, 14/23 chlamydia, 5/23 *M. genitalium*, 5/23 trichomonas, and 2/23 HIV.

Table 2 summarises the meta-analyses of each exposure-outcome pair and information about genital mycoplasmas in the presence or absence of BV (Table S5). In most meta-analyses, heterogeneity was low or moderate. Summary findings from different study designs were compatible, so we present summary measures across all study designs (Figures 1, 2, and S2.1-S2.3).

For peer review only

Table 2 Summary estimates, by outcome and organism, from random effects meta-analysis of unadjusted odds ratios, for associations between genital mycoplasmas and adverse birth outcomes, and summary of multivariable and analyses that stratify the main association by BV status

**Adverse outcome** ***Organism***	**No. of studies**	**Summary estimate*** **OR (95% CI)**	**I^2, %**	**Prediction interval**	**Any multivariable analysis†**	**Analyses of genital mycoplasmas and adverse birth outcomes in presence and absence of BV‡**
**Preterm birth**						
M. hominis	30	1.87 (1.49, 2.34)	29.2	0.98, 3.55	5 studies[1, 44, 45, 48, 61]	MH+,BV+/PTB OR 1.58 (95% CI 0.94, 2.77); MH+,BV-/PTB 1.18 (0.91, 1.52)[33]
U. urealyticum	27	1.84 (1.34, 2.55)	69.2	0.54, 6.36	5 studies[1, 41, 47, 65, 74]	UU+,BV+/PTB 0.47 (0.09, 3.31); UU+,BV-/PTB 1.15 (0.67, 1.98)[65]
U. parvum	11	1.60 (1.12, 2.30)	58.4	0.59, 4.36	2 studies[1, 47]	UP-,BV-/PTB; UP+,BV-/PTB Adjusted 1.6 (1.2, 2.1); UP-,BV+/PTB aOR 1.6 (1.1, 2.3); UP+,BV+/PTB aOR 2.6 (1.7, 4.0)[47]
**Premature rupture of membrane**						
M. hominis	11	1.94 (1.43, 2.70)	0.0	1.33, 2.83	1 study[61]	None reported
U. urealyticum	11	4.27 (1.83, 9.98)	87.3	0.27, 68.07	0 studies	
U. parvum	2	3.19 (1.25, 8.15)	0.0	NC	0 studies	
**Low birth weight**						None reported
M. hominis	6	1.81 (1.29, 2.52)	0.0	1.12, 2.90	1 study[34]	
U. urealyticum	2	2.24 (1.16, 4.33)	0.0	NC	0 studies	
U. parvum	0	NA	NA	NA	0 studies	
**Spontaneous abortion**						None reported
M. hominis	10	0.93 (0.44, 1.94)	50.2	0.12, 7.14	0 studies	

For peer review only

U. urealyticum	4	1.74 (1.02, 2.95)	0.0	0.54, 5.58	0 studies	
U. parvum	2	1.65 (0.67, 4.05)	0.0	NC	0 studies	
**Perinatal death**						None reported
M. hominis	9	2.70 (1.31, 5.57)	30.4	0.52, 13.94	0 studies	
U. urealyticum	2	9.50 (2.99, 30.13)	0.0	NC	0 studies	
U. parvum	1	NA			0 studies	

Abbreviations: aOR, adjusted odds ratio; BV, bacterial vaginosis; CI, confidence interval; I^2, heterogeneity; MH, *Mycoplasma hominis*; NA, not applicable; NC, could not be calculated; OR, odds ratio; UP, *Ureaplasma parvum*; UU, *Ureaplasma urealyticum*

* Meta-analysis of unadjusted ORs, using random effects model

† Details for individual studies reported in Tables S4.1-4.3

‡ Further details of analyses based on exclusion of other infections, stratification, or multivariable analyses in Table S5

For peer review only

Risk of bias within and across studies

Based on the NICE checklists,[27, 28] none of the 57 studies met all or most (++/++) checklist criteria for internal and external validity, 29 studies met some (+/+)[7, 9, 11, 15, 32, 33, 36, 40, 41, 45-47, 50, 52, 56-58, 60, 62, 64, 65, 67-70, 72-74] and 17 met few or no checklist criteria (-/-)[6, 8, 10, 12, 38, 39, 42-44, 49, 53, 55, 61, 66, 71, 79] (Table S6.1, 6.2, 6.3). Poor reporting of study methods meant that many items could not be assessed. In all study designs, control of confounding in most studies was poorly addressed or not addressed. Funnel plots for *M. hominis* (PTB, PROM, SA and PND), *U. urealyticum* (PTB, PROM) and *U. parvum* (PTB) did not show evidence of asymmetry (Table S7).

Associations between *M. hominis* and adverse pregnancy outcomes

There were 42 studies with data about *M. hominis*, reporting on 66 outcomes (Tables S2.1 - S2.3, S3.1). Of these, 30 included data about PTB.[1, 6, 8, 10, 15, 32, 33, 36, 38, 40, 42-46, 48, 50-54, 58, 59, 61, 66, 68, 69, 72, 73, 75] *M. hominis* was associated with PTB in meta-analysis of unadjusted ORs (19,576 women, summary OR 1.87, 95% CI 1.49, 2.34; I^2 29.2%; prediction interval 0.98, 3.55) (Figure 1). Five studies reporting a univariable association between *M. hominis* and PTB conducted multivariable analyses (Table 2, Table S5).[1, 44, 45, 48, 61] The association was attenuated in one (aOR 1.1, 95% CI 0.5, 2.5), after controlling for obstetric factors (previous PTB, miscarriage, multiple pregnancy and cervical incompetence).[61] In two others, authors reported no association with PTB <37 weeks, but subgroup analyses showed associations with PTB <35 [1] or <33[48] weeks. In two studies, no numerical results were reported (Table S2.1). In seven studies, authors also reported on BV.[33, 36, 43, 51, 58, 59, 72] In one study, the associations between *M. hominis*, BV and PTB could be examined in detail.[33] *M. hominis*, in the absence of BV, was less strongly

For peer review only

associated with PTB (OR 1.18, 95% CI 0.91, 1.52) than in the presence of BV (OR 1.58, 95% CI 0.94, 2.77).

[Figure 1]

Eleven studies included data about PROM.[6, 10, 40, 44, 45, 52, 61, 71, 73, 75, 79] *M. hominis* was associated with PROM in meta-analysis of unadjusted ORs (4,303 women, summary OR 1.94, 95% CI 1.40, 2.70; I^2 0.0 %; prediction interval 1.33, 2.83) (Figure S3.1). In one study with a multivariable analysis, the association was attenuated (aOR 1.1, 95% CI 0.3, 3.7)[61]. Six studies included data about LBW.[8, 34, 35, 49, 75, 77] *M. hominis* was associated with LBW in meta-analysis of unadjusted ORs (2,394 new-borns, summary OR 1.81, 95% CI 1.29, 2.52; I^2 0.0 %; prediction interval 1.12, 2.90) (Figure S3.2). In one study, *M hominis* was associated with LBW in multivariable analysis, when considered as a continuous variable (reported p=0.01).[34] In 10 studies with data about PND,[8, 35, 40, 45, 51, 54, 55, 76, 77] meta-analysis of unadjusted ORs found an association with *M. hominis* (3,696 women, summary OR 2.70, 95% CI 1.31, 4.54; I^2 30.4%; prediction interval 0.52, 13.94) (Figure S3.3). In 10 studies with data about SA,[6, 7, 11, 35, 36, 39, 40, 51, 55, 63] there was no association with *M. hominis* in meta-analysis of unadjusted ORs (4,531 women, summary OR 0.93, 95% CI 0.44, 1.49 ; I^2 50.2%; prediction interval 0.12, 7.14) (Figure S3.4). No results of multivariable analyses were reported for PND or SA.

Associations between *U. urealyticum* and adverse pregnancy outcomes

Thirty-one studies included data about *U. urealyticum* and 46 outcomes (Tables S2.1 -S2.3, S3.2). There were 27 studies with data about PTB.[1, 6, 10, 12, 15, 32, 38, 40, 41, 46-48, 50, 52, 53, 56, 57, 60, 62, 64, 65, 67, 70, 73-75, 78] In meta-analysis of unadjusted ORs, *U. urealyticum* was associated with PTB (12,234 women, summary OR 1.84, 95% CI 1.34, 2.55; I^2 69.2%; prediction interval 0.54, 6.36) (Figure 2). Five studies reported multivariable

For peer review only

analyses (Table 2, Table S5).[1, 41, 47, 65, 74] In one, multivariable and univariable associations were similar (aOR 1.4, 95% CI 0.8, 2.2).[47] In four, no numerical results were reported.[1] In one study with information about BV, there was no strong evidence of an association between *U. urealyticum* and PTB in the presence (OR 0.47, 95% CI 0.09, 3.31) or absence of BV (OR 1.15, 95% CI 0.67, 1.98).[65]

[Figure 2]

For all other outcomes, data were only available for meta-analysis of unadjusted ORs. *U. urealyticum* was associated with: PROM in 12 studies[6, 10, 37, 50, 52, 60, 62, 67, 71, 73, 74, 79] (3,676 participants, summary OR 4.27, 95% CI 1.83, 9.98; I^2 87.3%; prediction interval 0.27, 68.07) (Figure S4.1); LBW in two studies[12, 65] (506 participants, OR 2.24, 95% CI 1.16, 4.33; I^2 0.0%) (Figure S4.2); SA in four studies[6, 7, 9, 40] (2,140 women, summary OR 1.74, 95% CI 1.02, 2.95; I^2 0.0%; prediction interval 0.54, 5.58) (Figure S4.3); and PND in two studies[40, 60] (1,043 participants, summary OR 9.50, 95% CI 2.99, 30.13; I^2 0.0%) (Figure S4.4).

Associations between *U. parvum* and adverse pregnancy outcomes

Twelve studies included data about associations between *U. parvum* and 17 outcomes (Tables S2.1 -S2.3, S3.1). Eleven studies reported PTB.[1, 10, 12, 15, 38, 40, 46, 47, 56, 64, 78] In meta-analysis of unadjusted ORs, *U. parvum* was associated with PTB (8,002 women, summary OR 1.60, 95% CI 1.12, 2.30; I^2 58.4%; prediction interval 0.59, 4.36) (Figure 3). In one study,[47] a multivariable analysis found a stronger association with PTB when both *U. parvum* and BV were present (aOR 2.6, 95% CI 1.7, 4.0) than when *U. parvum* was present without BV (aOR 1.6, 95% CI 1.2, 2.1), when compared with women with neither infection (Table 2, Table S5). In one, no numerical results were reported.[1]

For peer review only

[Figure 3]

For all other outcomes, data were only available for meta-analysis of unadjusted ORs. *U. parvum* was associated with PROM in two studies[10, 40] (946 participants, OR 3.19, 95% CI 1.25, 8.15; I^2 0.0%) (Figure S5.1) and with SA in two studies[7, 40] (986 participant, summary OR 1.65, 95% CI 0.67, 4.05; I^2 0.0%) (Figure S5.2). One study reported on LBW (22 participants, 1 event, OR 0.56, 95% CI 0.01, 12.75)[12] and one on PND (872 women, 1 event, OR 2.79).[40]

DISCUSSION

Principal findings

This systematic review and meta-analysis included 57 studies about associations between *M. hominis, U. urealyticum* and *U. parvum* and five adverse pregnancy outcomes. Only 6/57 studies reported any multivariable analysis. In 51 studies, meta-analyses of unadjusted ORs found that *M. hominis* was associated with an increase in PTB, PROM, LBW, and PND, *U. urealyticum* with an increase in PTB, PROM, LBW, SA, and PND, and *U. parvum* with an increase in PTB and PROM. In three studies from which data about both genital mycoplasmas and BV could be extracted; *M. hominis* and *U. parvum* were less strongly associated with PTB in the absence of BV than in the presence of BV and no association with *U. urealyticum* was found in the presence or absence of BV.

Strengths and weaknesses of the study

The strengths of this systematic review and meta-analysis are first, that we followed a published protocol[22] with predefined outcomes and statistical analysis plan. Study selection, data extraction and risk of bias assessment were undertaken independently by two reviewers, to reduce subjectivity. Second, we examined evidence for heterogeneity visually

For peer review only

and statistically, and calculated prediction intervals that take into account the variability in estimates from different studies and predict a range of values that could be expected in a new study.[31] In several of the random effects models, the I^2 value was zero, suggesting that the variability between the estimates is due to chance. This is consistent with meta-analyses in which the sampling error is high and confidence intervals for estimates in individual studies all overlap (e.g., Figure S3.1 and S3.2). Third, we triangulated findings across study designs;[23, 26] despite the different potential sources of bias, the summary estimates were compatible and we judged it reasonable to combine effect estimates.[30] There were also limitations in the design of the review. Despite a predefined search strategy, with broad search terms, we might have missed relevant studies, particularly by restriction to languages not spoken fluently by the authors. There were too few studies to conduct all the planned sensitivity analyses by organism, but we described all studies that allowed stratification by BV status.

Comparison with existing literature and interpretation

We found a systematic review about genital mycoplasmas that included studies published in English or Chinese up to March 2020.[80] The focus of the review was on infertility, however, and limited search terms for studies about adverse pregnancy outcomes identified only 11 of the 57 studies that we included, making comparison difficult.

The findings from this systematic review cannot be interpreted as showing causal associations between colonisation with *M. hominis*, *U. urealyticum*, or *U. parvum* in pregnancy and some adverse pregnancy outcomes. We found associations in meta-analysis of unadjusted associations, but the confounder adjusted estimates could not be summarised. Most studies in this systematic review did not control for confounding by either sociodemographic characteristics, or co-infection with another organism or BV. We could not

For peer review only

elucidate the role of co-infection with BV,[4, 5] because there were only two relevant studies, with imprecise estimates. Rittenschober-Böhm *et al*., studied more than 4000 women in Germany.[47] They found univariable associations between both *U. parvum* (OR 1.7, 95% CI 1.3, 2.2) and *U. urealyticum* (1.4, 95% CI 0.9, 2.3) and spontaneous PTB. A strength of their study is the multivariable analysis, controlling for age, smoking, history of PTB and other infections. For *U. parvum,* the association with PTB was stronger when both BV and *U. parvum* were present than for *U. parvum* alone. The authors did not analyse the association with *U. urealyticum* further. Hillier *et al*., investigated the association between *M. hominis* and PTB of LBW infants in more than 10,000 women in the USA.[33] The association was stronger in the presence (1.58, 95% CI 0.94, 2.77) than absence (1.18, 95% CI 0.91, 1.52) of BV, but confidence intervals for both estimates include the null value. Hillier *et al*. also reported a stronger association with PTB when *M. hominis* was present with Bacteroides and BV (OR 2.1, 95% CI 1.5, 3.0). The authors did not, however, control for any other confounding factors.

Several of the limitations that we found in our review apply to systematic reviews of observational studies in general. Most included studies did not set out to study our review question and have small sample sizes. We extracted most data about genital mycoplasmas, our exposures of interest, from tables of covariates. Differences in the performance characteristics of diagnostic methods might have resulted in misclassification of infection status. Bacteriological culture has been considered the gold standard for the identification of genital mycoplasmas, but problems can arise from their fastidious growth requirements and a lack of reliable media. Commercialised kits for both culture and NAAT diagnosis are less laborious and have greater sensitivity and specificity compared with earlier in-house approaches.[81, 82] Sample integrity is also important and greatly influenced by sample collection methods (e.g. type of swab, transport medium), transportation (e.g. cold chain

For peer review only

maintenance) and storage (e.g. duration and temperature at which kept in long-term storage). It was not possible to account for differences in anatomical sampling site that may have affected detection in individual studies, e.g. *M. hominis* is more commonly isolated in the lower genital tract whilst *Ureaplasma* spp. colonise the upper genital tract.[83] Other limitations include misclassification, for example, gestational age was assessed by obstetric ultrasound in only one third of studies and inconsistency in the timing during pregnancy of sampling for genital mycoplasmas.

The specificity of associations between different genital mycoplasmas and adverse pregnancy, and their mechanisms of action, remain unclear. Several studies included in this review postulate that subclinical ascending *Ureaplasma* spp. to the choriodecidual space is followed by placental transfer into the amniotic cavity,[7, 76, 78, 84, 85] which then leads to PROM, SA, and PND in women with high bacterial load in the upper genital tract.[85, 86] The presence of genital mycoplasmas in the placental membranes and amniotic fluid might have a direct effect, but they also increase levels of a variety of cytokines and other inflammatory mediators, which might be the key drivers of adverse pregnancy outcomes.[32, 37, 52, 64, 67, 85, 87] Gene sequencing methods show the complexity and diversity of the vaginal microbiota during pregnancy [15, 16, 88] and genital mycoplasmas are often among the most plentiful of the many bacterial species identified. In our review, one study using 16s rRNA sequencing found a group of bacteria, including *U. parvum*, that was associated with PTB,[15] but another smaller study did not.[56] Analysis of associations between microbial communities and PTB was beyond the scope of our systematic review. A better understanding of antimicrobial susceptibility is also needed. Genital mycoplasmas lack a rigid cell wall, which allows them to evade some antibiotics. Beta-lactam antibiotics and vancomycin are considered ineffective but macrolides, fluoroquinolones and tetracyclines are often effective.[89] In pregnant women, only macrolides should be used[90] but high rates of

For peer review only

380 antibiotic resistance are reported in many settings,[4, 91, 92] and in the absence of definitive
381 evidence of the benefits of treatment, cannot currently be recommended.

382 **Implications**

383 The findings of this systematic review show key areas for future research. First, there is a
384 need for epidemiological studies that are designed specifically to investigate the
385 pathogenicity of vaginal and cervical organisms alone and in the context of the vaginal
386 microbiome. A holistic approach that includes gene sequencing and other molecular and
387 culture methods to detect other endogenous and sexually transmitted organisms is
388 required,[14-16] taking into account the need for consistent strategies for specimen collection
389 both in terms of the trimester(s) and the timing and types of specimens collected. These
390 studies should also define potential causal pathways and address confounding from factors
391 such as maternal age, smoking, obstetric history, co-infections and comorbidities. Second,
392 there is a critical need to conduct research in low- and middle-income settings where the
393 prevalence of sexually transmitted infections, BV and genital mycoplasma are high, and the
394 burden of adverse pregnancy outcomes greatest. If consistent and reproducible associations
395 are found in observational studies, potential interventions need to be evaluated. Randomised
396 controlled trials of screening and treatment for a range of vaginal and endocervical infections
397 in pregnancy are underway.[93, 94] If these interventions prevent adverse pregnancy
398 outcomes, further research will still be needed to understand the contributions of specific
399 organisms or combinations thereof. Multiplex assays will facilitate these research studies but
400 should not be used in routine clinical practice because of the risks of overdiagnosis and
401 overtreatment.[18, 19]

For peer review only

Conclusions

In this systematic review and meta-analysis, we found that genital mycoplasmas are associated with several different adverse pregnancy outcomes in univariable analysis only. The currently available literature does not allow conclusions about the role of mycoplasmas in adverse pregnancy and birth outcomes, alone or with co-existing bacterial vaginosis. Future studies that consider genital mycoplasmas in the context of the vaginal microbiome are needed.

Authors' roles

DEG, NL, AV, LV conceived the idea for the review and DEG, JK, NL, AV, LV, HW wrote the protocol. MJ and LV did the searches, screened, and selected studies and extracted data. DEG, NL, ES resolved disagreements. NL and HW did statistical analyses. MJ wrote the first draft of the manuscript. MJ, NL did review and editing. All authors commented on revisions of the manuscript and accept responsibility for its content.

Funding

NL receives funding from the Swiss National Science Foundation, project numbers 197831, 160909; LV is supported by an Australian National Health & Medical Research Council (NHMRC) Early Career Fellowship Grant (2018-2021); MJ is a PhD research student is supported through the Women And Newborns Trial of Antenatal Interventions and Management (WANTAIM) trial (ISRCTN No: ISRCTN37134032), funded by DFID/MRC/Wellcome Trust Joint Global Health Trials, Australian NHMRC Grant and Swiss National Science Foundation. DEG received salary support from r4d programme (Swiss Programme for Research on Global Issues for Development), grant number IZ07Z0-

For peer review only

160909. AV receives salary support from the Australian NHMRC, through a Career Development Fellowship.

Ethics, Patient and Public Involvement

This study does not involve huma participants or animal subjects. All data used are only from published data. Patients or the public were not involved in the design, or conduct, or reporting, or dissemination plans of our research.

Data availability

No additional or unpublished data available.

Conflict of interests

NL is on the advisory board of Sefunda AG, a start-up company that develops point-of-care tests for sexually transmitted infections.

For peer review only

REFERENCES

1. Agger WA, Siddiqui D, Lovrich SD, et al. Epidemiologic factors and urogenital infections associated with preterm birth in a midwestern U.S. population. *Obstet Gynecol* 2014;124(5):969-77. doi: https://dx.doi.org/10.1097/AOG.0000000000000470

2. Plummer EL, Vodstrcil LA, Bodiyabadu K, et al. Are Mycoplasma hominis, Ureaplasma urealyticum and Ureaplasma parvum Associated With Specific Genital Symptoms and Clinical Signs in Nonpregnant Women? *Clin Infect Dis* 2021

3. Horner P, Donders G, Cusini M, et al. Should we be testing for urogenital Mycoplasma hominis, Ureaplasma parvum and Ureaplasma urealyticum in men and women?–a position statement from the European STI Guidelines Editorial Board. *J Eur Acad Dermatol Venereol* 2018;32(11):1845-51.

4. Capoccia R, Greub G, Baud D. Ureaplasma urealyticum, Mycoplasma hominis and adverse pregnancy outcomes. *Curr Opin Infect Dis* 2013;26(3):231-40. doi: 10.1097/QCO.0b013e328360db58

5. Taylor-Robinson D, Lamont R. Mycoplasmas in pregnancy. *BJOG: Int J Obstet Gy* 2011;118(2):164-74.

6. Lee MY, Kim MH, Lee WI, et al. Prevalence and Antibiotic Susceptibility of Mycoplasma hominis and Ureaplasma urealyticum in Pregnant Women. *Yonsei Med J* 2016;57(5):1271-5. doi: https://dx.doi.org/10.3349/ymj.2016.57.5.1271

7. Oliveira C, Oliveira M, Oliveira H, et al. Association of spontaneous abortion and Ureaplasma parvum detected in placental tissue. *Epidemiol Infect* 2020;148:e126. doi: 10.1017/S0950268820001302 [published Online First: 2020/07/07]

8. Luton D, Ville Y, Luton-Sigy A, et al. Prevalence and influence of Mycoplasma hominis and Ureaplasma urealyticum in 218 African pregnant women and their infants. *Eur J Obstet Gynecol Reprod Biol* 1994;56(2):95-101.

9. Ahmadi A, Khodabandehloo M, Ramazanzadeh R, et al. Association between Ureaplasma urealyticum endocervical infection and spontaneous abortion in in Sanandaj, Iran. *Iran J Microbiol* 2014;6(6):392-97.

10. Jones HE, Harris KA, Azizia M, et al. Differing prevalence and diversity of bacterial species in fetal membranes from very preterm and term labor. *PLoS One* 2009;4 (12) (no pagination)(e8205) doi: http://dx.doi.org/10.1371/journal.pone.0008205

11. Farhadifar F, Khodabandehloo M, Ramazanzadeh R, et al. Survey on association between Mycoplasma hominis endocervical infection and spontaneous abortion using Polymerase Chain Reaction. *Int J Reprod Biomed* 2016;14(3):181-86.

12. Peretz A, Tameri O, Azrad M, et al. Mycoplasma and Ureaplasma carriage in pregnant women: the prevalence of transmission from mother to newborn. *BMC Pregnancy Childbirth* 2020;20(1):456. doi: 10.1186/s12884-020-03147-9

13. Donders GG, Ruban K, Bellen G, et al. Mycoplasma/Ureaplasma infection in pregnancy: to screen or not to screen. *J Perinat Med* 2017;45(5):505-15. doi: 10.1515/jpm-2016-0111

14. van de Wijgert JH. The vaginal microbiome and sexually transmitted infections are interlinked: consequences for treatment and prevention. *PLoS Med* 2017;14(12):e1002478.

15. Payne MS, Newnham JP, Doherty DA, et al. A Specific Bacterial DNA Signature in the Vagina of Australian Women in Mid-Pregnancy Predicts High Risk of Spontaneous Preterm Birth (The Predict1000 Study). *Am J Obstet Gynecol* 2021 doi: 10.1016/j.ajog.2020.08.034 [published Online First: 2020/08/31]

16. Pace RM, Chu DM, Prince AL, et al. Complex species and strain ecology of the vaginal microbiome from pregnancy to postpartum and association with preterm birth. *Med (N Y)* 2021;2(9):1027-49. doi: 10.1016/j.medj.2021.06.001 [published Online First: 2021/10/08]

17. Vouga M, Greub G, Prod'hom G, et al. Treatment of genital mycoplasma in colonized pregnant women in late pregnancy is associated with a lower rate of premature labour and neonatal complications. *Clin Microbiol Infect* 2014;20(10):1074-79. doi: http://dx.doi.org/10.1111/1469-0691.12686

18. Jensen JS. To Test or Not to Test for Mycoplasma hominis and Ureaplasmas: That's (Not) the Question. *Clin Infect Dis* 2021;73(4):669-71. doi: 10.1093/cid/ciab065 [published Online First: 2021/01/26]

For peer review only

19. Taylor-Robinson D, Horner P, Pallecaros A. Diagnosis of some genital-tract infections: part 2. Molecular tests and the new challenges. *Int J STD AIDS* 2020;31(3):198-207. doi: 10.1177/0956462419890526 [published Online First: 2020/02/06]

20. Kong F, Ma Z, James G, et al. Species identification and subtyping of Ureaplasma parvum and Ureaplasma urealyticum using PCR-based assays. *J Clin Microbiol* 2000;38(3):1175-9.

21. Robertson JA, Stemke GW, Davis Jr JW, et al. Proposal of Ureaplasma parvum sp. nov. and emended description of Ureaplasma urealyticum (Shepard et al. 1974) Robertson et al. 2001. *Int J Syst Evol Microbiol* 2002;52(2):587-97.

22. Vallely LM, Egli-Gany D, Pomat W, et al. Adverse pregnancy and neonatal outcomes associated with Neisseria gonorrhoeae, Mycoplasma genitalium, M. hominis, Ureaplasma urealyticum and U. parvum: a systematic review and meta-analysis protocol. *BMJ Open* 2018;8(11)

23. Vallely LM, Egli-Gany D, Wand H, et al. Adverse pregnancy and neonatal outcomes associated with Neisseria gonorrhoeae: systematic review and meta-analysis. *Sex Transm Infect* 2021;97(2):104-11.

24. Frenzer C, Egli-Gany D, Vallely LM, et al. Adverse pregnancy and neonatal outcomes associated with Mycoplasma genitalium: systematic review and meta-analysis [In Press]. *Sex Transm Infect* 2022;Epub ahead of print: doi: doi:10.1136/sextrans-2021-055352

25. Page MJ, McKenzie JE, Bossuyt PM, et al. The PRISMA 2020 statement: an updated guideline for reporting systematic reviews. *BMJ* 2021;372:n71. doi: 10.1136/bmj.n71 [published Online First: 2021/03/31]

26. Dekkers OM, Vandenbroucke JP, Cevallos M, et al. COSMOS-E: guidance on conducting systematic reviews and meta-analyses of observational studies of etiology. *PLoS Med* 2019;16(2):e1002742.

27. National Institute for Health Care Excellence. The social care guidance manual. Great Britain: National Institute for Health and Care Excellence, 2016.

For peer review only

28. National Institute for Health Care Excellence. Methods for the development of NICE public health guidance. Great Britain: National Institute for Health and Care Excellence 2012.

29. Egger M, Davey SG, Schneider M, et al. Bias in meta-analysis detected by a simple, graphical test. *BMJ* 1997;315(7109):629-34.

30. Low N. Chlamydia trachomatis and reproductive health: what can we learn from systematic reviews of observational studies? *Sex Transm Infect* 2020 doi: 10.1136/sextrans-2019-054279 [published Online First: 2020/01/29]

31. Riley RD, Higgins JP, Deeks JJ. Interpretation of random effects meta-analyses. *BMJ* 2011;342:d549.

32. Daskalakis G, Thomakos N, Papapanagiotou A, et al. Amniotic fluid interleukin-18 at mid-trimester genetic amniocentesis: Relationship to intraamniotic microbial invasion and preterm delivery. *BJOG: Int J Obstet Gy* 2009;116(13):1743-48. doi: http://dx.doi.org/10.1111/j.1471-0528.2009.02364.x

33. Hillier SL, Nugent RP, Eschenbach DA, et al. Association between bacterial vaginosis and preterm delivery of a low-birth-weight infant. *N Engl J Med* 1995;333(26):1737-42.

34. Berman SM, Harrison HR, Boyce WT, et al. Low birth weight, prematurity, and postpartum endometritis. Association with prenatal cervical Mycoplasma hominis and Chlamydia trachomatis infections. *JAMA* 1987;257(9):1189-94. [published Online First: 1987/03/06]

35. Braun P, Lee YH, Klein JO, et al. Birth Weight and Genital Mycoplasmas in Pregnancy. *N Engl J Med* 1971;284(4):167-71. doi: 10.1056/NEJM197101282840401

36. Donders GG, Van Calsteren K, Bellen G, et al. Predictive value for preterm birth of abnormal vaginal flora, bacterial vaginosis and aerobic vaginitis during the first trimester of pregnancy. *BJOG: Int J Obstet Gy* 2009;116(10):1315-24. doi: https://dx.doi.org/10.1111/j.1471-0528.2009.02237.x

37. Gerber S, Vial Y, Hohlfeld P, et al. Detection of Ureaplasma urealyticum in second-trimester amniotic fluid by polymerase chain reaction correlates with subsequent preterm labor and delivery. *J Infect Dis* 2003;187(3):518-21. doi: https://dx.doi.org/10.1086/368205

For peer review only

38. Govender S, Theron GB, Odendaal HJ, et al. Prevalence of genital mycoplasmas, ureaplasmas and chlamydia in pregnancy. *J Obstet Gynaecol* 2009;29(8):698-701. doi: 10.3109/01443610903184033 [published Online First: 2009/10/14]

39. Harrison HR, Alexander ER, Weinstein L, et al. Cervical Chlamydia trachomatis and mycoplasmal infections in pregnancy. Epidemiology and outcomes. *JAMA* 1983;250(13):1721-7. [published Online First: 1983/10/07]

40. Kataoka S, Yamada T, Chou K, et al. Association between preterm birth and vaginal colonization by mycoplasmas in early pregnancy. *J Clin Microbiol* 2006;44(1):51-5. doi: https://dx.doi.org/10.1128/JCM.44.1.51-55.2006

41. Koucky M, Malickova K, Cindrova-Davies T, et al. Prolonged progesterone administration is associated with less frequent cervicovaginal colonization by Ureaplasma urealyticum during pregnancy - Results of a pilot study. *J Reprod Immunol* 2016;116:35-41. doi: http://dx.doi.org/10.1016/j.jri.2016.04.285

42. McDonald HM, O'Loughlin JA, Jolley PT, et al. Changes in vaginal flora during pregnancy and association with preterm birth. *J Infect Dis* 1994;170(3):724-8.

43. Menard JP, Mazouni C, Salem-Cherif I, et al. High vaginal concentrations of atopobium vaginae and gardnerella vaginalis in women undergoing preterm labor. *Obstet Gynecol* 2010;115(1):134-40. doi: http://dx.doi.org/10.1097/AOG.0b013e3181c391d7

44. Minkoff H, Grunebaum AN, Schwarz RH. Risk factors for prematurity and premature rupture of membranes: A prospective study of the vaginal flora in pregnancy. *Am J Obstet Gynecol* 1984;150(8):965-72.

45. Nguyen DP, Gerber S, Hohlfeld P, et al. Mycoplasma hominis in mid-trimester amniotic fluid: relation to pregnancy outcome. *J Perinat Med* 2004;32(4):323-6. doi: 10.1515/JPM.2004.060 [published Online First: 2004/09/07]

46. Payne MS, Ireland DJ, Watts R, et al. Ureaplasma parvum genotype, combined vaginal colonisation with Candida albicans, and spontaneous preterm birth in an Australian cohort of pregnant women. *BMC Pregnancy Childbirth* 2016;16:312-12.

For peer review only

47. Rittenschober-Böhm J, Waldhoer T, Schulz SM, et al. First Trimester Vaginal Ureaplasma Biovar Colonization and Preterm Birth: Results of a Prospective Multicenter Study. *Neonatology* 2018;113(1):1-6. doi: http://dx.doi.org/10.1159/000480065 [published Online First: 2017/09/22]

48. Usui R, Ohkuchi A, Matsubara S, et al. Vaginal lactobacilli and preterm birth. *J Perinat Med* 2002;30(6):458-66. doi: 10.1515/JPM.2002.072 [published Online First: 2003/01/18]

49. Sperling RS, Newton E, Gibbs RS. Intraamniotic Infection in Low-Birth-Weight Infants. *J Infect Dis* 1988;157(1):113-17.

50. Kwak DW, Hwang HS, Kwon JY, et al. Co-infection with vaginal Ureaplasma urealyticum and Mycoplasma hominis increases adverse pregnancy outcomes in patients with preterm labor or preterm premature rupture of membranes. *J Matern -Fetal Neonatal Med* 2014;27(4):333-7. doi: https://dx.doi.org/10.3109/14767058.2013.818124

51. Odendaal HJ, Popov I, Schoeman J, et al. Preterm labour--is Mycoplasma hominis involved? *S Afr Med J* 2002;92(3):235-7. [published Online First: 2002/06/04]

52. Perni SC, Vardhana S, Korneeva I, et al. Mycoplasma hominis and Ureaplasma urealyticum in midtrimester amniotic fluid: association with amniotic fluid cytokine levels and pregnancy outcome. *Am J Obstet Gynecol* 2004;191(4):1382-6. doi: 10.1016/j.ajog.2004.05.070 [published Online First: 2004/10/28]

53. Schwab FD, Zettler EK, Moh A, et al. Predictive factors for preterm delivery under rural conditions in post-tsunami Banda Aceh. *J Perinat Med* 2015;44(5):511-5. doi: 10.1515/jpm-2015-0004 [published Online First: 2015/05/20]

54. Cassell GH, Davis RO, Waites KB, et al. Isolation of Mycoplasma hominis and Ureaplasma urealyticum from amniotic fluid at 16-20 weeks of gestation: Potential effect on outcome of pregnancy. *Sex Transm Dis* 1983;10(4 SUPPL.):294-302.

55. Embree JE, Krause VW, Embil JA, et al. Placental infection with Mycoplasma homonis and Ureaplasma urealyticum: clinical correlation. *Obstet Gynecol* 1980;56(4):475-81.

For peer review only

56. Freitas AC, Bocking A, Hill JE, et al. Increased richness and diversity of the vaginal microbiota and spontaneous preterm birth. *Microbiome* 2018;6(1):117. doi: 10.1186/s40168-018-0502-8 [published Online First: 2018/06/30]

57. Harada K, Tanaka H, Komori S, et al. Vaginal infection with Ureaplasma urealyticum accounts for preterm delivery via induction of inflammatory responses. *Microbiol Immunol* 2008;52(6):297-304. doi: https://dx.doi.org/10.1111/j.1348-0421.2008.00039.x

58. Hillier SL, Martius J, Krohn M, et al. A case-control study of chorioamnionic infection and histologic chorioamnionitis in prematurity. *N Engl J Med* 1988;319(15):972-78.

59. Holst E, Goffeng AR, Andersch B. Bacterial vaginosis and vaginal microorganisms in idiopathic premature labor and association with pregnancy outcome. *J Clin Microbiol* 1994;32(1):176-86.

60. Kafetzis DA, Skevaki CL, Skouteri V, et al. Maternal genital colonization with Ureaplasma urealyticum promotes preterm delivery: association of the respiratory colonization of premature infants with chronic lung disease and increased mortality. *Clin Infect Dis* 2004;39(8):1113-22. doi: https://dx.doi.org/10.1086/424505

61. McDonald HM, O'Loughlin JA, Jolley P, et al. Prenatal microbiological risk factors associated with preterm birth. *Br J Obstet Gynaecol* 1992;99(3):190-6. [published Online First: 1992/03/01]

62. Mitsunari M, Yoshida S, Deura I, et al. Cervical Ureaplasma urealyticum colonization might be associated with increased incidence of preterm delivery in pregnant women without prophlogistic microorganisms on routine examination. *J Obstet Gynaecol Res* 2005;31(1):16-21. doi: https://dx.doi.org/10.1111/j.1447-0756.2005.00246.x

63. Munday PE, Porter R, Falder PF. Spontaneous abortion, an infectious aetiology? *Br J Obstet Gynaecol* 1984;91(12):1177-80.

64. Payne MS, Feng Z, Li S, et al. Second trimester amniotic fluid cytokine concentrations, Ureaplasma sp. colonisation status and sexual activity as predictors of preterm birth in Chinese and Australian women. *BMC Pregnancy Childbirth* 2014;14:340. doi: https://dx.doi.org/10.1186/1471-2393-14-340

For peer review only

65. Povlsen K, Thorsen P, Lind I. Relationship of Ureaplasma urealyticum biovars to the presence or absence of bacterial vaginosis in pregnant women and to the time of delivery. *Eur J Clin Microbiol Infect Dis* 2001;20(1):65-7. [published Online First: 2001/03/14]

66. Toth KS, Letchworth AT, Noble AD, et al. The significance of infection in the aetiology of preterm labour. A prospective controlled study. *J Obstet Gynaecol* 1992;12(2):94-99.

67. Yoon BH, Oh SY, Romero R, et al. An elevated amniotic fluid matrix metalloproteinase-8 level at the time of mid-trimester genetic amniocentesis is a risk factor for spontaneous preterm delivery. *Am J Obstet Gynecol* 2001;185(5):1162-7. doi: https://dx.doi.org/10.1067/mob.2001.117678

68. Gonzàlez Bosquet E, Gene A, Ferrer I, et al. Value of endocervical Ureaplasma species colonization as a marker of preterm delivery. *Gynecol Obstet Invest* 2006;61(3):119-23. doi: http://dx.doi.org/10.1159/000089457

69. Chua KB, Ngeow YF, Lim CT, et al. Colonization and transmission of Ureaplasma urealyticum and Mycoplasma hominis from mothers to full and preterm babies by normal vaginal delivery. *Med J Malaysia* 1999;54(2):242-6. [published Online First: 2000/09/06]

70. Jalava J, Laurikainen E, Karkkainen U, et al. Cervical ureaplasma urealyticum colonization: comparison of PCR and culture for its detection and association with preterm birth. *Scand J Infect Dis* 2002;34(1):35-40.

71. Kacerovsky M, Pavlovsky M, Tosner J. Preterm premature rupture of the membranes and genital mycoplasmas. *Acta Medica (Hradec Kralove)* 2009;52(3):117-20.

72. Kumar SS, V.; Sharma, M. Bacterial vaginosis in preterm labor. *Int J Gynaecol Obstet* 2006;95(1):40-41. doi: http://dx.doi.org/10.1016/j.ijgo.2006.05.022

73. Montenegro DA, Borda LF, Neuta Y, et al. Oral and uro-vaginal intra-amniotic infection in women with preterm delivery: A case-control study. *J Investig Clin Dent* 2019;10(2):e12396. doi: http://dx.doi.org/10.1111/jicd.12396

74. Abele-Horn M, Scholz M, Wolff C, et al. High-density vaginal Ureaplasma urealyticum colonization as a risk factor for chorioamnionitis and preterm delivery. *Acta Obstet Gynecol Scand* 2000;79(11):973-8.

For peer review only

75. Grattard F, Soleihac B, De Barbeyrac B, et al. Epidemiologic and molecular investigations of genital mycoplasmas from women and neonates at delivery. *Pediatr Infect Dis J* 1995;14(10):853-8.

76. Kundsin RB, Driscoll SG, Monson RR, et al. Association of Ureaplasma urealyticum in the placenta with perinatal morbidity and mortality. *N Engl J Med* 1984;310(15):941-45.

77. McCormack WM, Rosner B, Lee YH, et al. Isolation of genital mycoplasmas from blood obtained shortly after vaginal delivery. *Lancet* 1975;1(7907):596-9.

78. Sweeney EL, Kallapur SG, Gisslen T, et al. Placental Infection With Ureaplasma species Is Associated With Histologic Chorioamnionitis and Adverse Outcomes in Moderately Preterm and Late-Preterm Infants. *J Infect Dis* 2016;213(8):1340-47. doi: 10.1093/infdis/jiv587

79. Nasution TA, Cheong SF, Lim CT, et al. Multiplex PCR for the detection of urogenital pathogens in mothers and newborns. *Malays J Pathol* 2007;29(1):19-24.

80. Ma C, Du J, Dou Y, et al. The Associations of Genital Mycoplasmas with Female Infertility and Adverse Pregnancy Outcomes: a Systematic Review and Meta-analysis. *Reprod Sci* 2021:1-19.

81. D'Inzeo T, De Angelis G, Fiori B, et al. Comparison of Mycoplasma IES, Mycofast Revolution and Mycoplasma IST2 to detect genital mycoplasmas in clinical samples. *J Infect Dev Ctries* 2017;11(01):98-101.

82. Kusanovic JP, Vargas P, Ferrer F, et al. Comparison of two identification and susceptibility test kits for Ureaplasma spp and Mycoplasma hominis in amniotic fluid of patients at high risk for intra-amniotic infection. *J Matern -Fetal Neonatal Med* 2020;33(20):3409-17. doi: 10.1080/14767058.2019.1572742

83. Taylor-Robinson D. Infections Due to Species of Mycoplasma and Ureaplasma: An Update. *Clin Infect Dis* 1996;23(4):671-82. doi: 10.1093/clinids/23.4.671

84. Pavlidis I, Spiller OB, Demarco GS, et al. Cervical epithelial damage promotes Ureaplasma parvum ascending infection, intrauterine inflammation and preterm birth induction in mice. *Nat Commun* 2020;11(1):1-12.

For peer review only

85. Kasper DC, Mechtler TP, Reischer GH, et al. The bacterial load of Ureaplasma parvum in amniotic fluid is correlated with an increased intrauterine inflammatory response. *Diagn Microbiol Infect Dis* 2010;67(2):117-21. doi: https://dx.doi.org/10.1016/j.diagmicrobio.2009.12.023

86. Witt A, Berger A, Gruber CJ, et al. Increased intrauterine frequency of Ureaplasma urealyticum in women with preterm labor and preterm premature rupture of the membranes and subsequent cesarean delivery. *Am J Obstet Gynecol* 2005;193(5):1663-9. doi: 10.1016/j.ajog.2005.03.067 [published Online First: 2005/11/02]

87. Li Y-H, Brauner A, Jonsson B, et al. Ureaplasma urealyticum-induced production of proinflammatory cytokines by macrophages. *Pediatr Res* 2000;48(1):114-19.

88. Doyle RM, Alber DG, Jones HE, et al. Term and preterm labour are associated with distinct microbial community structures in placental membranes which are independent of mode of delivery. *Placenta* 2014;35(12):1099-101. doi: https://dx.doi.org/10.1016/j.placenta.2014.10.007

89. Combaz-Söhnchen N, Kuhn A. A systematic review of Mycoplasma and Ureaplasma in urogynaecology. *Geburtshilfe Frauenheilkd* 2017;77(12):1299.

90. Redelinghuys MJ, Ehlers MM, Dreyer AW, et al. Antimicrobial susceptibility patterns of Ureaplasma species and Mycoplasma hominis in pregnant women. *BMC Infect Dis* 2014;14:171. doi: https://dx.doi.org/10.1186/1471-2334-14-171

91. Bae I, Koh E, Kim S, et al. Prevalence rate and antimicrobial susceptibilities of Ureaplasma urealyticum and Mycoplasma hominis in pregnant women residing in Jinju, Korea. *Clin Microbiol Infect* 2010;2):S485. doi: http://dx.doi.org/10.1111/j.1469-0691.2010.03239.x

92. Bayraktar MR, Ozerol IH, Gucluer N, et al. Prevalence and antibiotic susceptibility of Mycoplasma hominis and Ureaplasma urealyticum in pregnant women. *Int J Infect Dis* 2010;14(2):e90-5. doi: https://dx.doi.org/10.1016/j.ijid.2009.03.020

93. Vallely AJ, Pomat WS, Homer C, et al. Point-of-care testing and treatment of sexually transmitted infections to improve birth outcomes in high-burden, low-income settings: Study

For peer review only

protocol for a cluster randomized crossover trial (the WANTAIM Trial, Papua New Guinea). *Wellcome Open Res* 2019;4

94. Grant JS, Chico RM, Lee AC, et al. Sexually transmitted infections in pregnancy: a narrative review of the global research gaps, challenges, and opportunities. *Sex Transm Dis* 2020;47(12):779.

For peer review only

Figure legends

Figure 1. Forest plot of univariable association between *M. hominis* and preterm birth, from random effects meta-analysis. Studies are in order of precision. Solid diamonds and lines either side are point estimates and 95% confidence intervals for individual studies (arrows show where lower or upper confidence limits extend beyond the x-axis limits). Open diamond shows the point estimate and 95% confidence interval for the summary odds ratio and lines either side of the diamond show the prediction interval.

Figure 2. Forest plot of univariable association between *U. urealyticum* and preterm birth, from random effects meta-analysis. Studies are in order of sample size. Solid diamonds and lines either side are point estimates and 95% confidence intervals for individual studies (arrows show where lower or upper confidence limits extend beyond the x-axis limits). Open diamond shows the point estimate and 95% confidence interval for the summary odds ratio and lines either side of the diamond show the prediction interval.

Figure 3. Forest plot of univariable association between *U. parvum* and preterm birth, from random effects meta-analysis. Studies are in order of precision. Solid diamonds and lines either side are point estimates and 95% confidence intervals for individual studies (arrows show where lower or upper confidence limits extend beyond the x-axis limits). Open diamond shows the point estimate and 95% confidence interval for the summary odds ratio and lines either side of the diamond show the prediction interval.

For peer review only

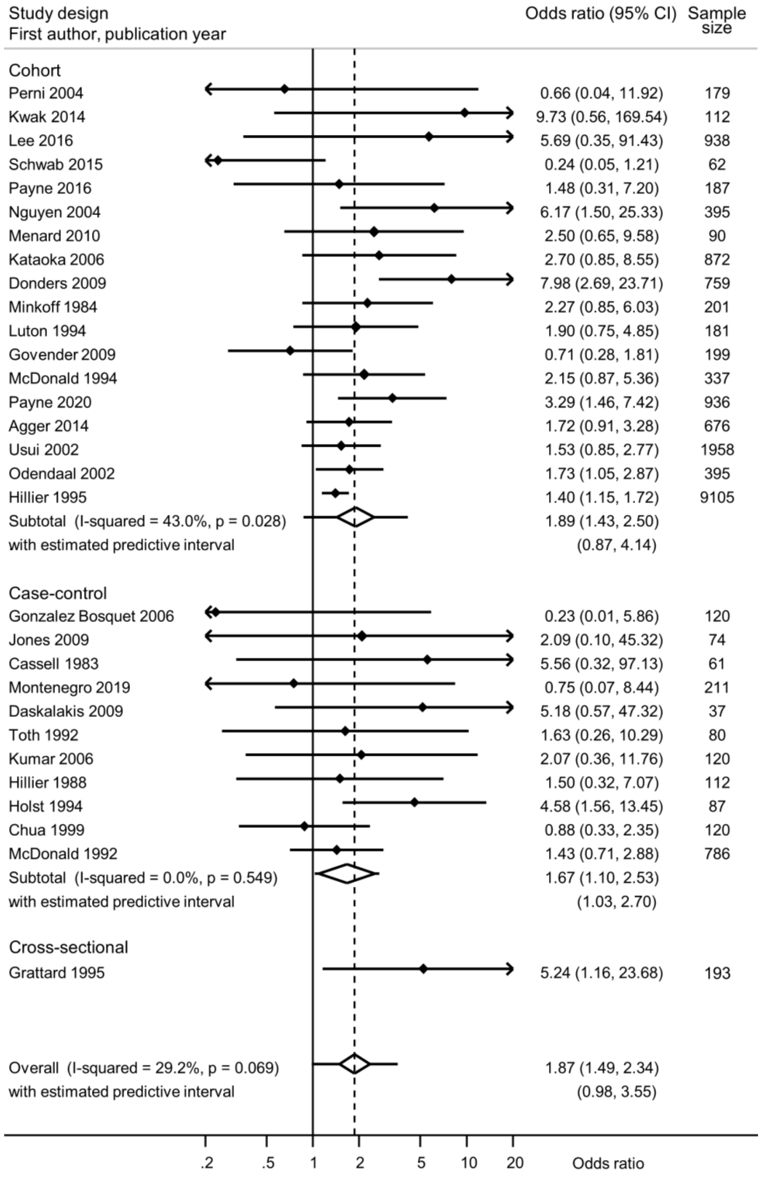

Figure 1. Forest plot of univariable association between M. hominis and preterm birth, from random effects meta-analysis. Studies are in order of precision. Solid diamonds and lines either side are point estimates and 95% confidence intervals for individual studies (arrows show where lower or upper confidence limits extend beyond the x-axis limits). Open diamond shows the point estimate and 95% confidence interval for the summary odds ratio and lines either side of the diamond show the predictive interval.

170x270mm (300 x 300 DPI)

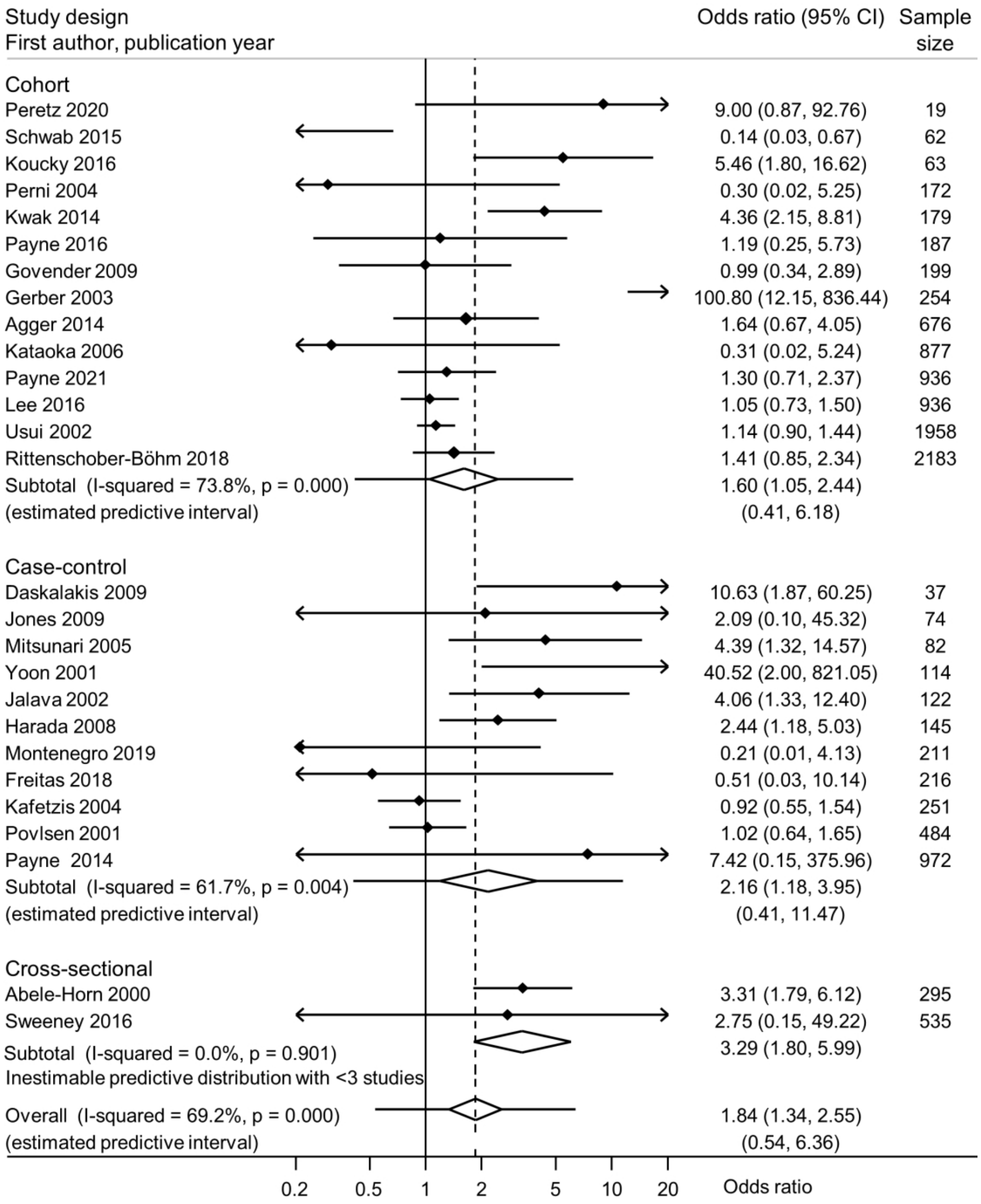

Figure 2. Forest plot of univariable association between U. urealyticum and preterm birth, from random effects meta-analysis. Studies are in order of sample size. Solid diamonds and lines either side are point estimates and 95% confidence intervals for individual studies (arrows show where lower or upper confidence limits extend beyond the x-axis limits). Open diamond shows the point estimate and 95% confidence interval for the summary odds ratio and lines either side of the diamond show the predictive interval.

178x218mm (300 x 300 DPI)

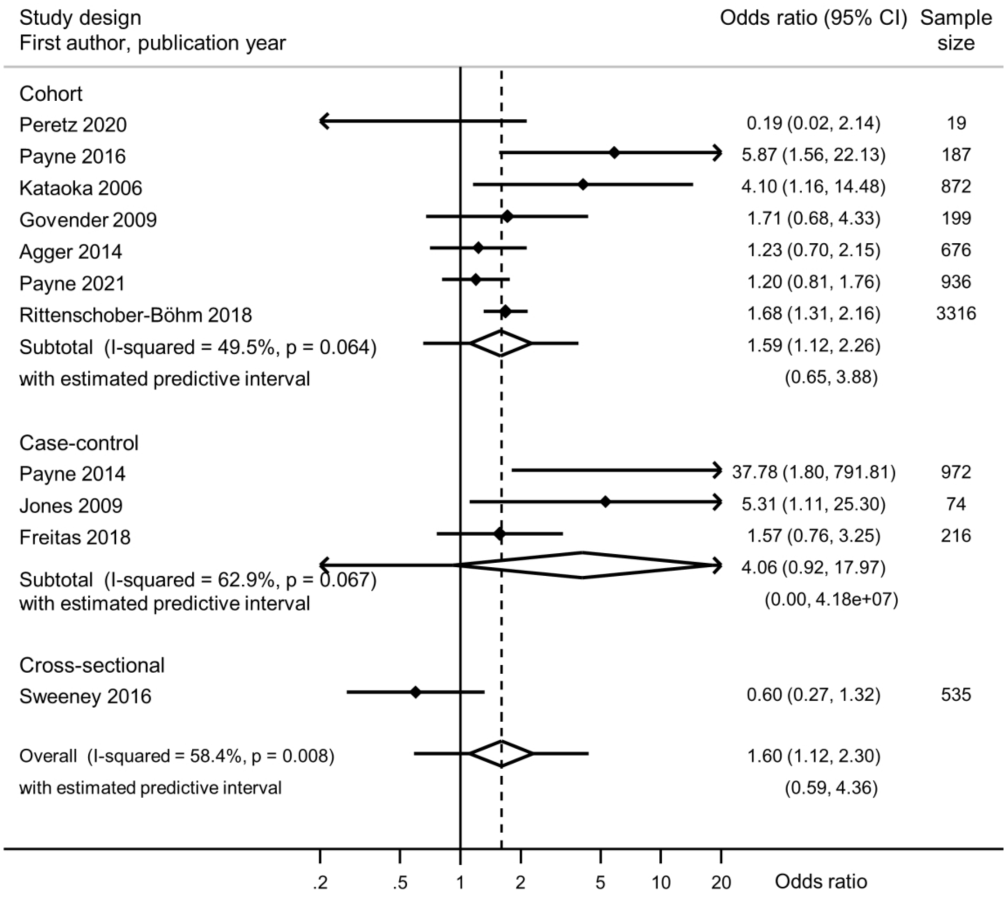

Figure 3. Forest plot of univariable association between U. parvum and preterm birth, from random effects meta-analysis. Studies are in order of precision. Solid diamonds and lines either side are point estimates and 95% confidence intervals for individual studies (arrows show where lower or upper confidence limits extend beyond the x-axis limits). Open diamond shows the point estimate and 95% confidence interval for the summary odds ratio and lines either side of the diamond show the predictive interval.

175x156mm (300 x 300 DPI)

Supplementary Material

Adverse birth outcomes associated with *Mycoplasma hominis*, *Ureaplasma urealyticum* and *Ureaplasma parvum*: A systematic review and meta-analysis.

Marinjho Emely JONDUO (MSc),[1] Lisa Michelle VALLELY (PhD),[1,2] Handan WAND (PhD),[3] Emma Louise SWEENEY (PhD),[4] Dianne EGLI-GANY (PhD),[5] John Martin KALDOR (PhD),[1] Andrew John VALLELY (PhD),[1,2] Nicola LOW (PhD).[5]

[1] Public Health Interventions Research Program, The Kirby Institute, University of New South Wales, Sydney, NSW 2052, Australia.

[2] Sexual and Reproductive Health Unit, Papua New Guinea Institute of Medical Research, Goroka, Eastern Highlands Province 441, Papua New Guinea.

[3] Biostatistics and Databases program, The Kirby Institute, University of New South Wales, Sydney, New South Wales, Australia.

[4] The University of Queensland Center for Clinical Research, Brisbane, Queensland 4029, Australia.

[5] Institute of Social and Preventive Medicine, University of Bern, CH-3012 Bern, Switzerland.

For peer review only

Table of Contents

Table of Contents ... 2

A. 1 Preferred reporting items for systematic review and meta-analysis (PRISMA) ... 4

A.2 Preferred reporting checklist of Meta-analysis (MOOSE)[1] ... 10

A.3 Search strategy ... 13

Figure S1 Flow chart of identified and selected studies for inclusion ... 14

Table S1 Summary of characteristics of included studies ... 15

Figure S3.1 Forest plot of association between *M. hominis* and premature rupture of membrane, random effects model. ... 33

Figure S3.2 Forest plot of association between *M. hominis* and low birthweight, random effects model. ... 34

Figure S3.3 Forest plot of association between *M. hominis* and perinatal death random effects model. ... 35

Figure S3.4 Forest plot of association between *M. hominis* and spontaneous abortion random effects model. ... 36

Figure S4.1 Forest plot of association between *U. urealyticum* and premature rupture of membrane, random effects model. ... 37

Figure S4.2 Forest plot of association between *U. urealyticum* and low birth weight, random effects model. ... 38

Figure S4.3 Forest plot of association between *U. urealyticum* and spontaneous abortion, random effects model ... 39

Figure S5.1 Forest plot of association between *U. parvum* and premature rupture of membrane, random effects model. ... 41

Figure S5.2 Forest plot of association between *U. parvum* and spontaneous abortion random effects model ... 42

Table S2.1 Descriptive tables: Cohort studies (n=26) ... 43

Table S2.2 Descriptive tables: Case control studies (n=25) ... 49

Table S2.3 Descriptive tables: Cross sectional studies (n=6) ... 53

Table S3.1 Summary description of studies reporting *M. hominis* (n=42), by income status ... 55

Table S3.2 Summary description of studies reporting *U. urealyticum* (n=31), by income status ... 64

Table S3.3 Summary description of studies reporting *U. parvum* (n=12), by income status ... 71

Table S4.1 Study setting and socio-demographics, cohort studies (n=26) ... 74

Table S4.2 Study setting and socio-demographics, case-control studies (n=25) ... 77

Table S4.3 Study setting and socio-demographics, cross-sectional studies (n=6) ... 80

Table S5 Studies that reported on bacterial vaginosis or sexually transmitted infections and reported associations with adverse birth outcomes ... 81

Table S6.1 Risk of bias assessment, cohort studies (n=26) ... 86

For peer review only

Table S6.2 Risk of bias assessment, case-control studies (n=25) 101

Table S6.3 Risk of bias assessment, cross-sectional studies (n=6) 109

Table S7 Summary of assessment of funnel plot asymmetry, for outcomes reported in 10 or more studies 111

For peer review only

For peer review only - http://bmjopen.bmj.com/site/about/guidelines.xhtml

A. 1 Preferred reporting items for systematic review and meta-analysis (PRISMA)

Section and Topic	Item #	Checklist item	Location where item is reported
TITLE			
Title	1	Identify the report as a systematic review.	1
ABSTRACT			
Abstract	2	See the PRISMA 2020 for Abstracts checklist.	4-5
INTRODUCTION			
Rationale	3	Describe the rationale for the review in the context of existing knowledge.	6
Objectives	4	Provide an explicit statement of the objective(s) or question(s) the review addresses.	7
METHODS			
Eligibility criteria	5	Specify the inclusion and exclusion criteria for the review and how studies were grouped for the syntheses.	7
Information sources	6	Specify all databases, registers, websites, organisations, reference lists and other sources searched or consulted to identify studies. Specify the date when each source was last searched or consulted.	8
Search strategy	7	Present the full search strategies for all databases, registers and websites, including any filters and limits used.	Appendix S1
Selection process	8	Specify the methods used to decide whether a study met the inclusion criteria of the review, including how many reviewers screened each record and each report	8

For peer review only

Section and Topic	Item #	Checklist item	Location where item is reported
		retrieved, whether they worked independently, and if applicable, details of automation tools used in the process.	
Data collection process	9	Specify the methods used to collect data from reports, including how many reviewers collected data from each report, whether they worked independently, any processes for obtaining or confirming data from study investigators, and if applicable, details of automation tools used in the process.	8-9
Data items	10a	List and define all outcomes for which data were sought. Specify whether all results that were compatible with each outcome domain in each study were sought (e.g. for all measures, time points, analyses), and if not, the methods used to decide which results to collect.	6
	10b	List and define all other variables for which data were sought (e.g. participant and intervention characteristics, funding sources). Describe any assumptions made about any missing or unclear information.	8-9
Study risk of bias assessment	11	Specify the methods used to assess risk of bias in the included studies, including details of the tool(s) used, how many reviewers assessed each study and whether they worked independently, and if applicable, details of automation tools used in the process.	9
Effect measures	12	Specify for each outcome the effect measure(s) (e.g. risk ratio, mean difference) used in the synthesis or presentation of results.	9
Synthesis methods	13a	Describe the processes used to decide which studies were eligible for each synthesis (e.g. tabulating the study intervention characteristics and comparing against the planned groups for each synthesis (item #5)).	9

For peer review only - http://bmjopen.bmj.com/site/about/guidelines.xhtml

Section and Topic	Item #	Checklist item	Location where item is reported
	13b	Describe any methods required to prepare the data for presentation or synthesis, such as handling of missing summary statistics, or data conversions.	9
	13c	Describe any methods used to tabulate or visually display results of individual studies and syntheses.	9
	13d	Describe any methods used to synthesize results and provide a rationale for the choice(s). If meta-analysis was performed, describe the model(s), method(s) to identify the presence and extent of statistical heterogeneity, and software package(s) used.	9
	13e	Describe any methods used to explore possible causes of heterogeneity among study results (e.g. subgroup analysis, meta-regression).	9
	13f	Describe any sensitivity analyses conducted to assess robustness of the synthesized results.	None
Reporting bias assessment	14	Describe any methods used to assess risk of bias due to missing results in a synthesis (arising from reporting biases).	9
Certainty assessment	15	Describe any methods used to assess certainty (or confidence) in the body of evidence for an outcome.	9
RESULTS			
Study selection	16a	Describe the results of the search and selection process, from the number of records identified in the search to the number of studies included in the review, ideally using a flow diagram.	Figure S1

For peer review only - http://bmjopen.bmj.com/site/about/guidelines.xhtml

Section and Topic	Item #	Checklist item	Location where item is reported
	16b	Cite studies that might appear to meet the inclusion criteria, but which were excluded, and explain why they were excluded.	Figure S1
Study characteristics	17	Cite each included study and present its characteristics.	Table S1, reference list
Risk of bias in studies	18	Present assessments of risk of bias for each included study.	11, supporting information
Results of individual studies	19	For all outcomes, present, for each study: (a) summary statistics for each group (where appropriate) and (b) an effect estimate and its precision (e.g. confidence/credible interval), ideally using structured tables or plots.	Table 1, Fig 1, Fig 2, Fig 3, supporting information
Results of syntheses	20a	For each synthesis, briefly summarise the characteristics and risk of bias among contributing studies.	11, supporting information
	20b	Present results of all statistical syntheses conducted. If meta-analysis was done, present for each the summary estimate and its precision (e.g. confidence/credible interval) and measures of statistical heterogeneity. If comparing groups, describe the direction of the effect.	11-14
	20c	Present results of all investigations of possible causes of heterogeneity among study results.	Supporting information
	20d	Present results of all sensitivity analyses conducted to assess the robustness of the synthesized results.	None
Reporting biases	21	Present assessments of risk of bias due to missing results (arising from reporting biases) for each synthesis assessed.	None

For peer review only

Section and Topic	Item #	Checklist item	Location where item is reported
Certainty of evidence	22	Present assessments of certainty (or confidence) in the body of evidence for each outcome assessed.	Fig 1, Fig 2, Fig 3, supporting information
DISCUSSION			
Discussion	23a	Provide a general interpretation of the results in the context of other evidence.	15
	23b	Discuss any limitations of the evidence included in the review.	16
	23c	Discuss any limitations of the review processes used.	15
	23d	Discuss implications of the results for practice, policy, and future research.	17-18
OTHER INFORMATION			
Registration and protocol	24a	Provide registration information for the review, including register name and registration number, or state that the review was not registered.	1
	24b	Indicate where the review protocol can be accessed, or state that a protocol was not prepared.	1
	24c	Describe and explain any amendments to information provided at registration or in the protocol.	None
Support	25	Describe sources of financial or non-financial support for the review, and the role of the funders or sponsors in the review.	18
Competing interests	26	Declare any competing interests of review authors.	19

For peer review only

Section and Topic	Item #	Checklist item	Location where item is reported
Availability of data, code and other materials	27	Report which of the following are publicly available and where they can be found: template data collection forms; data extracted from included studies; data used for all analyses; analytic code; any other materials used in the review.	Supporting information

For peer review only

A.2 Preferred reporting checklist of Meta-analysis (MOOSE)[1]

**Checklist item**	**Page #**
**Reporting of background should include**	
Problem definition	6
Hypothesis statement	No
Description of study outcome(s)	6
Type of exposure or intervention used	6-7
Type of study designs used	7
Study population	7
**Reporting of search strategy should include**	
Qualifications of searchers (eg. Librarians and investigators)	1
Search strategy, including time period included in the synthesis and keywords	7-8, Appendix S1
Effort to include all available studies, including contact with authors	No
Databases and registries searched	8
Search software used, name and version, including special features used (eg, explosion)	No
Use of hand searching (eg, reference lists of obtained articles	8
List of citations located and those excluded, including justification	Fig 1,<br>Table S1, excluded studies not listed

For peer review only

Method of addressing articles published in languages other than English	8
Method of handling abstracts and unpublished studies	Excluded
Description of any contact with authors	We did not contact authors
**Reporting of methods should include**	
Description of relevance or appropriateness of studies assembled for assessing the hypothesis to be tested	7-8
Rationale for the selection and coding of data (eg, multiple raters, blinding and interrater reliability)	7-8
Assessment of confounding (eg, comparability of cases and controls in studies where appropriate)	7
Assessment of study quality, including blinding of quality assessors; stratification or regression on possible predictors of study results	9
Assessment of heterogeneity	9
Description of statistical methods (eg, complete description of fixed or random effects models, justification of whether the chosen models account for predictors of study results, dose-response models, cumulative meta-analysis) in sufficient detail to be replicated	9
Provision of appropriate tables and graphs	Main text and supporting information
**Reporting of results should include**	
Graphic summarizing individual study estimates and overall estimate	Main text and forest plots in supporting information

For peer review only

Table giving descriptive information for each study included	Table S1
Results of sensitivity testing (eg, subgroup analysis)	None
Indication of statistical uncertainty of findings	Main text and forest plots in supporting information
**Reporting of discussion should include**	
Quantitative assessment of bias (eg, publication bias)	15-16
Justification of exclusion (eg. Exclusion of non-English-language citations)	No (provided pg 8 on methods)
Assessment of Quality of included studies	15-17
**Reporting of conclusions should include**	
Consideration of alternative explanations of observations	17-18
Generalization of conclusions (ie, appropriate for the data presented and within the domain of the literature review)	17-18
Guidelines for future research	17-18
Disclosure of funding source	18

[1] Stroup DF, Berlin JA, Morton SC, Olkin I, Williamson GD, Rennie D, et al. Meta-analysis of observational studies in epidemiology: a proposal for reporting. **Jama**. 2000 Apr 19;283(15):2008-12.

For peer review only

A.3 Search strategy

1. Terms for population	"pregnancy" or "prenatal" or "antenatal"
2. Terms for exposure	"Mycoplasma hominis" or "M. hominis"; "Ureaplasma urealyticum" or "U. urealyticum"; "Ureaplasma parvum" or "U. parvum"
3. Terms for outcomes	"birth outcome" or "adverse birth outcome" or "adverse pregnancy outcome" or "perinatal morbidity" or "perinatal mortality" or "perinatal outcome" or "premature birth" or "premature delivery" or "very preterm birth" or "preterm birth" or "preterm delivery" or "premature labour" or "preterm labour" or "premature labor" or "preterm labor" or "premature rupture of membranes" or "preterm rupture of membranes" or "preterm premature rupture of membranes" or "low birth weight" or "intrauterine growth retardation" or "intrauterine growth restriction" or "small for gestational age" or "gestational age" or "stillbirth" or "perinatal mortality" or "perinatal morbidity" or "perinatal death" or "neonatal mortality" or "neonatal morbidity" or "neonatal death" or "fetal death" or "miscarriage" or "spontaneous abortion" or "chorioamnionitis"
4. Search = #1 + # 2 + # 3	

Free text terms in the search strategy will use truncated and wildcard forms e.g., pregn*

Explode function used for MeSH headings

Reference lists of retrieved articles searched

For peer review only - http://bmjopen.bmj.com/site/about/guidelines.xhtml

Figure S1 Flow chart of identified and selected studies for inclusion

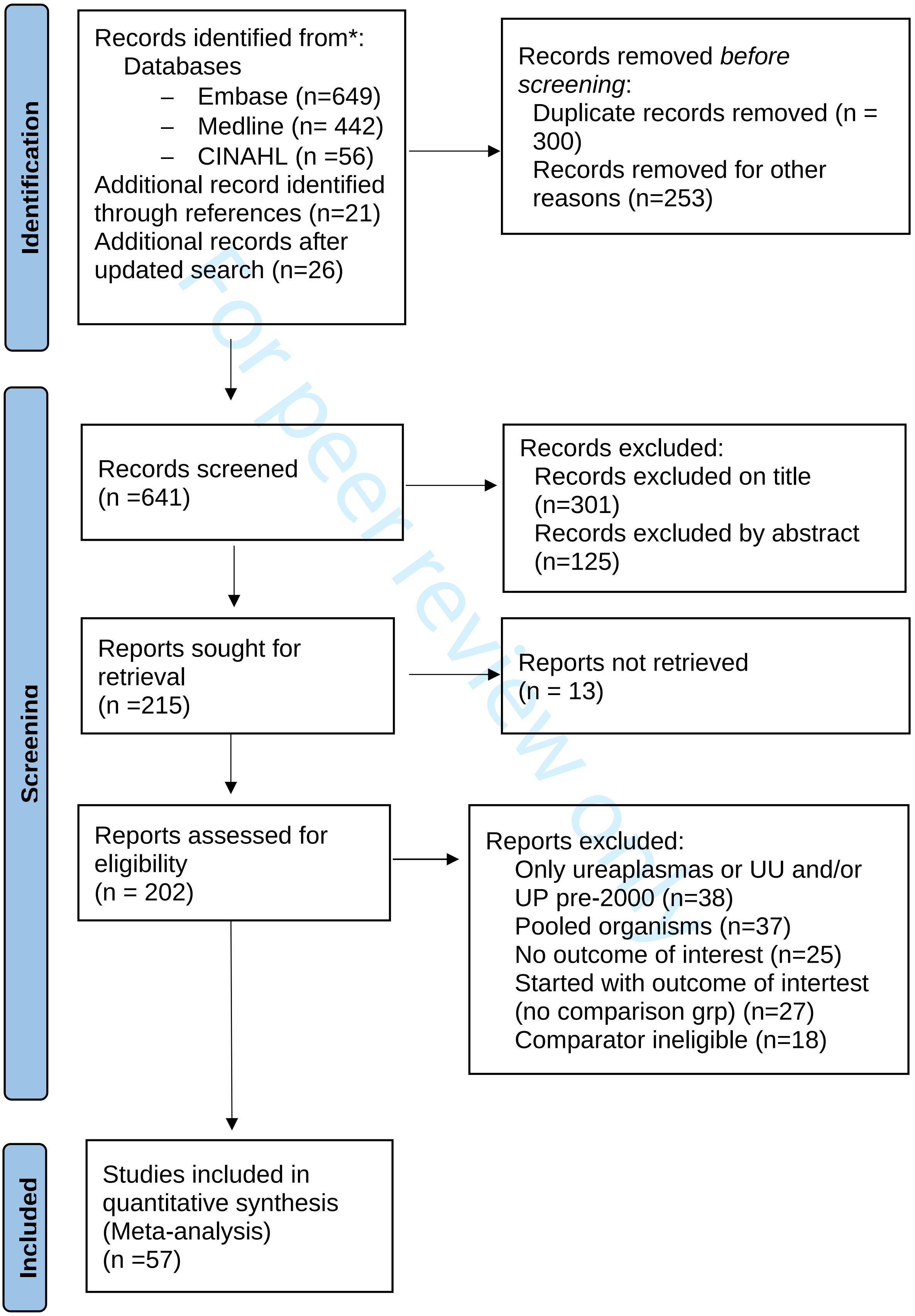

For peer review only - http://bmjopen.bmj.com/site/about/guidelines.xhtml

Table S1 Summary of characteristics of included studies

First author, publication year, study reference*†	Country	Study design	Study population	Total no. of women	Outcomes measured	Specimen type; collection time	Diagnostic method	BV assessed
**Abele-Horn, 2000[73]**	Germany	Cross-sectional	Admitted for delivery, Jan - Dec 1996	295	PTB, PROM	Endocervical swab; 1st & 2nd trimester	Culture	Excluded
**Agger, 2014[1]**	USA	Cohort	10 to 14 weeks gestation, initial prenatal visit; currently uncomplicated pregnancy	783	PTB	Endocervical swab; 1st, 2nd trimester	NAAT	NR
**Ahmadi, 2014[9]**	Iran	Case-control	10-20 weeks (cases); normal pregnancy 20-30 weeks (control)	218	SA	Endocervical swab; 1st, 2nd, 3rd trimester	NAAT	NR
**Berman, 1987[32]**	Mexico	Cohort	Women at their prenatal care visit, single centre; Oct 1980 - Oct 1983	1204	LBW	Endocervical swab; 1st, 2nd, 3rd trimester	Culture	NR

For peer review only - http://bmjopen.bmj.com/site/about/guidelines.xhtml

First author, publication year, study reference*†	Country	Study design	Study population	Total no. of women	Outcomes measured	Specimen type; collection time	Diagnostic method	BV assessed
**Braun, 1971[33]**	USA	Cohort	Entering antenatal clinic, single centre; Feb-Jul 1969	688	LBW	Endocervical swab, urine; 1st & 3rd trimester	Culture	NR
**Cassell, 1983[53]**	USA	Case-control	Attending the amniocentesis for prenatal diagnosis, single centre	61	PTB, PND	Amniotic fluid; 2nd trimester	Culture	NR
**Chua, 1999[68]**	Malaysia	Case-control	60 sequential mother who delivered and premature babies, single centre, Jan 1996- June 1997	120	PTB	Endocervical swab; 2nd, 3rd trimester	Culture	NR
**Daskalakis, 2009[30]**	NR	Case-control	Singleton, normal pregnancy, >18 years old, mid-trimester	613	PTB	Amniotic fluid; 2nd trimester	Culture	NR

Supporting information

First author, publication year, study reference*†	Country	Study design	Study population	Total no. of women	Outcomes measured	Specimen type; collection time	Diagnostic method	BV assessed
			amniocentesis, Feb 2006 - Sept 2007					
**Donders, 2009[34]**	Belgium	Cohort	Singleton, first antenatal visit between 9 -16 weeks with complete data available on *M. hominis* cultures; June 2000 – Dec 2001	759	PTB, SA	Vaginal swab; 1st & 2nd trimester	Culture	Yes
**Embree, 1980[54]**	Canada	Case-control	Single centre, deliveries between May 1977 and Jan 1978	554	SA, PND	Placenta; Post-partum	Culture	NR
**Farhadifar, 2016[11]**	Iran	Case-control	Admitted in obstetrics and gynaecology wards; no	218	SA	Endocervical swab; 1st, 2nd, 3rd trimester	NAAT	NR

Supporting information

First author, publication year, study reference*†	Country	Study design	Study population	Total no. of women	Outcomes measured	Specimen type; collection time	Diagnostic method	BV assessed
			antibiotics two weeks before sampling, no chronic disease, vaginal infection; Aug 2012 – Jan 2013					
**Freitas, 2018[55]**	Canada	Case-control	Spontaneous preterm birth and term deliveries, multicentre	216	PTB	Vaginal swab; 2nd trimester	NAAT	NR
**Gerber, 2003[80]**	NR	Cohort	Transabdominal amniocentesis at 15-17 weeks GA; singleton without complicated pregnancy	254	PTB, PROM	Amniotic fluid; 2nd trimester	NAAT	NR
**Gonzàlez Bosquet, 2006[67]**	NR	Case-control	Case: 24-34 weeks PTL, intact membranes; control: no history	250	PTB	Endocervical swab; NR	Culture	Yes

For peer review only - http://bmjopen.bmj.com/site/about/guidelines.xhtml

First author, publication year, study reference*†	Country	Study design	Study population	Total no. of women	Outcomes measured	Specimen type; collection time	Diagnostic method	BV assessed
			of preterm birth at same stage of delivery					
**Govender, 2009[79]**	South Africa	Cohort	Low risk obstetric patients at first prenatal visit (16-23 weeks gestation)	199	PTB	Endocervical swab; 2nd trimester	NAAT	NR
**Grattard, 1995[74]**	France	Cross-sectional	Women who delivered between Feb - May 1993 in obstetrical ward and their neonates, single centre	208	PTB, LBW PROM,	Endocervical swab; post-partum	Culture	NR
**Harada, 2008[56]**	Japan	Case-control	Premature and term deliveries, Jan 2006 - July 2007	145	PTB	Endocervical swab; 2nd, 3rd trimester	NAAT, Culture	NR

Supporting information

First author, publication year, study reference*†	Country	Study design	Study population	Total no. of women	Outcomes measured	Specimen type; collection time	Diagnostic method	BV assessed
**Harrison, 1983**[37]	USA	Cohort	Enrolled at their first prenatal visit, single centre	860	SA	Endocervical swab; 1st, 2nd, 3rd trimester	Culture, ELISA	NR
**Hillier, 1988**[57]	USA	Case-control	Age >16 years; no antibiotics in previous 2 weeks; no known fetal anomaly; June 1984- June 1985	112	PTB	Placenta; Post-partum	Culture	Yes
**Hillier, 1995**[31]	USA	Cohort	> 16 years, singleton pregnancies at routine prenatal visits (23 to 26 weeks), between 1984-1989,	10,397	PTB	Endocervical swab; 2nd trimester	Culture	Yes

Supporting information

For peer review only - http://bmjopen.bmj.com/site/about/guidelines.xhtml

First author, publication year, study reference*†	Country	Study design	Study population	Total no. of women	Outcomes measured	Specimen type; collection time	Diagnostic method	BV assessed
**Holst, 1994[58]**	Sweden	Case-control	Women presenting in PTL; controls were women with no pregnancy history	87	PTB	Endocervical swab; 3rd trimester	Culture	yes
**Jalava, 2002[69]**	NR	Case-control	Control: 3rd trimester, no signs labour. Cases: contractions as sign of premature labour 22-35/40	122	PTB	Endocervical swab; 2nd, 3rd trimester	NAAT	NR
**Jones, 2009[10]**	United Kingdom	Case-control	Single centre, cases: <32 weeks gestation; Control >37 weeks; single centre	74	PTB, PROM	Placenta; Post-partum	NAAT	NR
**Kacerovsky, 2009[70]**	NR	Case-control	Pregnancy with PPROM, single centre, Jan 2004 - Feb 2007.	450	PROM	Endocervical swab; 2nd, 3rd trimester	Culture	NR

For peer review only - http://bmjopen.bmj.com/site/about/guidelines.xhtml

First author, publication year, study reference*†	Country	Study design	Study population	Total no. of women	Outcomes measured	Specimen type; collection time	Diagnostic method	BV assessed
**Kafetzis, 2004[59]**	Greece	Case-control	Case: premature delivery; control: term delivery from June 2000 to Dec 2001	251	PTB, PROM, PND	Vaginal swab; 3rd trimester	Culture	NR
**Kataoka, 2006[38]**	Japan	Cohort	Singleton pregnancies at <11 weeks of gestation, single centre, Jan – Dec 2002	1040	PTB, PROM, SA, PND	Vaginal swab;1st trimester	NAAT	NR
**Koucky, 2016[39]**	Czech Republic	Cohort	Threatened premature deliveries, between Aug 2012 - Feb 2013	63	PTB	Vaginal swab; 2nd, 3rd trimester	NAAT	NR
**Kumar, 2006[71]**	India	Case-control	Women in spontaneous premature/term labour with or	120	PTB	Vaginal swab; 3rd trimester	Culture	Yes

For peer review only - http://bmjopen.bmj.com/site/about/guidelines.xhtml

First author, publication year, study reference*†	Country	Study design	Study population	Total no. of women	Outcomes measured	Specimen type; collection time	Diagnostic method	BV assessed
			without rupture of membrane, single centre					
**Kundsin, 1984[75]**	USA	Cross-sectional	Deliveries at single centre, between Nov 1978 - Jun 1981	801	PND	Placenta; Post-partum	Culture	NR
**Kwak, 2014[49]**	South Korea	Cohort	Women with spontaneous premature labour or preterm PROM, Dec 2005 – Apr 2007, single centre	179	PTB	Vaginal swab; 3rd trimester	Culture	NR
**Lee, 2016[6]**	South Korea	Cohort	Aged 15-47, delivered babies at single centre between Jun 2009 - May 2014	1,035	PTB, PROM, SA	Vaginal swab; NR	Culture	NR

For peer review only - http://bmjopen.bmj.com/site/about/guidelines.xhtml

First author, publication year, study reference*†	Country	Study design	Study population	Total no. of women	Outcomes measured	Specimen type; collection time	Diagnostic method	BV assessed
**Luton, 1994[8]**	Gabon	Cohort	Singleton pregnancy at <20 weeks gestation, Sept 1990 to Nov 1991	218	PTB, LBW, PND	Endocervical swab; 1st, 2nd trimester	Culture	NR
**McCormack, 1975[76]**	USA	Cross-sectional	Vaginal deliveries, single centre,	327	LBW, PND	Blood; post-partum	Culture	NR
**McDonald, 1992[60]**	Australia	Case-control	Women who booked at one of 4 study centres, Oct 1986 – Dec 1988	2190	PTB, PROM	Endocervical swab; 2nd trimester	Culture	NR
**McDonald, 1994[40]**	Australia	Cohort	Patients attending the antenatal clinic, Oct 1986 - May 1990	560	PTB	Endocervical swab; 2nd, 3rd trimester	Culture	NR

Supporting information

First author, publication year, study reference*†	Country	Study design	Study population	Total no. of women	Outcomes measured	Specimen type; collection time	Diagnostic method	BV assessed
**Menard, 2010[41]**	France	Cohort	Admitted preterm labour with no pregnancy related complications from July 2007 - July 2008	90	PTB	Vaginal swab; 2nd, 3rd trimester	NAAT	Yes
**Minkoff, 1984[42]**	USA	Cohort	Attending single centre, for delivery between Mar - Sept 1982	250	PTB, PROM	Vaginal swab; 1st, 2nd trimester	Culture	NR
**Mitsunari, 2005[61]**	Japan	Case-control	Singleton pregnancy delivery, between Jan 2002 - Sept 2003	82	PTB, PROM	Endocervical swab; 2nd, 3rd trimester	NAAT	NR
**Montenegro, 2019[72]**	Colombia	Case-control	Pregnant women >18 years, no pregnancy related problems,	211	PTB, PROM	Placenta; Post-partum	NAAT	NR

Supporting information

For peer review only - http://bmjopen.bmj.com/site/about/guidelines.xhtml

First author, publication year, study reference*†	Country	Study design	Study population	Total no. of women	Outcomes measured	Specimen type; collection time	Diagnostic method	BV assessed
			non-smokers, no alcohol, no antibiotic					
**Munday, 1984[62]**	United Kingdom	Case-control	Women admitted with vaginal bleeding before 28 weeks gestation and women attending one antenatal clinic at same hospital	241	SA	Endocervical swab; 2nd, 3rd trimester	Culture	NR
**Nasution, 2007[78]**	NR	Cross-sectional	Women admitted with preterm PROM (<37weeks), normal vaginal deliveries at term, and women with post-partum fever	120	PROM	Placenta; Post-partum	NAAT	NR

Supporting information

For peer review only - http://bmjopen.bmj.com/site/about/guidelines.xhtml

First author, publication year, study reference*†	Country	Study design	Study population	Total no. of women	Outcomes measured	Specimen type; collection time	Diagnostic method	BV assessed
**Nguyen, 2004**[43]	Switzerland	Cohort	Women with transabdominal amniocentesis at 15-17 weeks gestation, single centre	456	PTB, PROM, PND	Amniotic fluid; 2nd trimester	NAAT	NR
**Odendaal, 2002**[50]	South Africa	Cohort	Primigravid, first visit, 16-26 weeks with previous preterm labour or miscarriage, May-Dec 1996	395	PTB, SA, PND	Endocervical swab; 2nd trimester	Culture	Yes
**Oliveira, 2020**[7]	Brazil	Case-control	> 18 years old, cases: 8 -20 weeks gestation; Control vaginal delivery at 38-40 weeks, Jul 2017 – Aug 2018,	109	SA	Endocervical swab; 1st, 2nd, 3rd trimester	NAAT	NR

First author, publication year, study reference*†	Country	Study design	Study population	Total no. of women	Outcomes measured	Specimen type; collection time	Diagnostic method	BV assessed
**Payne, 2014[63]**	China and Australia	Case-control	Singleton pregnancy referred for genetic amniocentesis	972	PTB	Amniotic fluid; 2nd trimester	NAAT	NR
**Payne, 2016[44]**	Australia	Cohort	Low risk singleton pregnancy, 18-40 years old, at 1st or 2nd trimester when enrolled	191	PTB	Vaginal swab; 1st, 2nd, 3rd trimester	NAAT, Culture	NR
**Payne, 2021[45]**	Australia	Cohort	Nulliparous and multiparous, singleton pregnancy, ≥16 years between 12 - 23 weeks gestation	1000	PTB	Vaginal swab; 1st, 2nd trimester	NAAT	NR
**Peretz, 2020[12]**	Israel	Cohort	Women, 18-45 years, at any stage of labor and any mode of delivery, between Jun 2014 and Jan 2016.	214	PTB, LBW	Vaginal swab; post-partum	NAAT	NR

Supporting information

First author, publication year, study reference*†	Country	Study design	Study population	Total no. of women	Outcomes measured	Specimen type; collection time	Diagnostic method	BV assessed
**Perni, 2004[51]**	Unknown	Cohort	Singleton pregnancy: underwent transabdominal amniocentesis at 15-19 weeks with clear amniotic fluid	193	PTB, PROM	Amniotic fluid; 2nd trimester	NAAT	NR
**Povlsen, 2001[64]**	Denmark	Case-control	Singleton, single centre; attending first antenatal visit between Nov 1992 - Feb 1994	484	PTB, LBW	Endocervical swab; 1st, 2nd trimester	NAAT	Yes
**Rittenschober-Böhm, 2018[46]**	Austria	Cohort	Attending routine nuchal translucency screening between 12-14 weeks gestation, multicentre study	4330	PTB	Endocervical swab; 1st, 2nd trimester	NAAT	Yes

Supporting information

For peer review only - http://bmjopen.bmj.com/site/about/guidelines.xhtml

**First author, publication year, study reference*†**	**Country**	**Study design**	**Study population**	**Total no. of women**	**Outcomes measured**	**Specimen type; collection time**	**Diagnostic method**	**BV assessed**
**Schwab, 2015[52]**	Indonesia	Cohort	2nd trimester, four centres, from Feb -Jun 2005	159	PTB	Vaginal swab; 2nd trimester	NAAT	Yes
**Sperling, 1988[48]**	USA	Cohort	Clinical diagnosis of intraamniotic infection, July 1979 – Dec 1986	409	LBW	Amniotic fluid; NR	Culture	NR
**Sweeney, 2016[77]**	USA	Cross-sectional	Term deliveries, no HIV infection, congenital infection, or fetal malformation, Jul 2010–Apr 2013	535	PTB	Placenta; Post-partum	NAAT, Culture	NR
**Toth, 1992[65]**	UK	Case-control	Admitted for delivery between Jan 1985 - Dec 1986	100	PTB	Endocervical swab; NR	Culture	NR

Supporting information

First author, publication year, study reference*†	Country	Study design	Study population	Total no. of women	Outcomes measured	Specimen type; collection time	Diagnostic method	BV assessed
**Usui, 2002[47]**	Japan	Cohort	Singleton pregnancy attending first antenatal visit, Jan 1995 – Mar 1998	1958	PTB	Endocervical swab; 1st, 3rd trimester	Culture	NR
**Yoon, 2001[66]**	South Korea	Case-control	Women who underwent mid-trimester amniocentesis	114	PTB, PROM	Amniotic fluid; 2nd trimester	NAAT	NR

Abbreviations: LBW, infant low birth weight, defined as birth weight <2.5kg; NAAT, nucleic acid amplification tests; NR, not reported; PTB, preterm birth - defined as birth before 37 weeks gestation; PROM: premature rupture of membrane- defined as clinically confirmed rupture of membrane before 37weeks of gestation; PND, perinatal death- defined as stillbirth (death after 20/40 gestation) or neonatal death (death <28 day since birth), unless otherwise defined by the study authors; SA: spontaneous abortion - defined as pregnancy loss at <20 weeks gestation or as defined by author

USA, United States of America; UK, United Kingdom.

* Study reference is the reference number cited in the main manuscript

† Additional summary information about the characteristics of included studies in Tables S3.1-S3.3, S4.1-S4.3, S5.1-S5.3

Supporting information

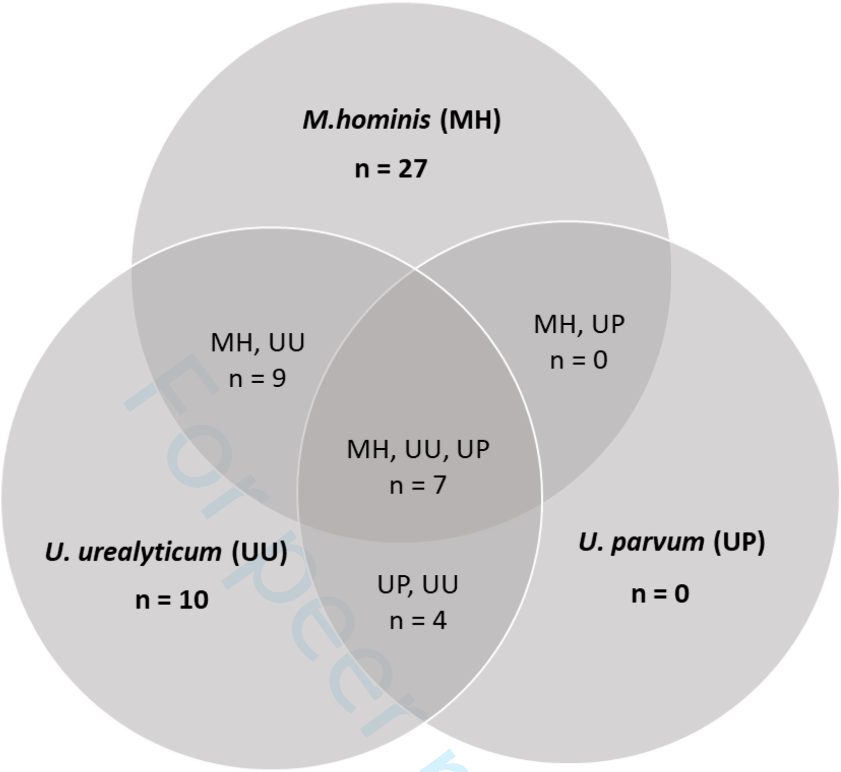

Figure S2 Venn diagram showing organisms reported in in the 57 articles included in the systematic review.

For peer review only - http://bmjopen.bmj.com/site/about/guidelines.xhtml

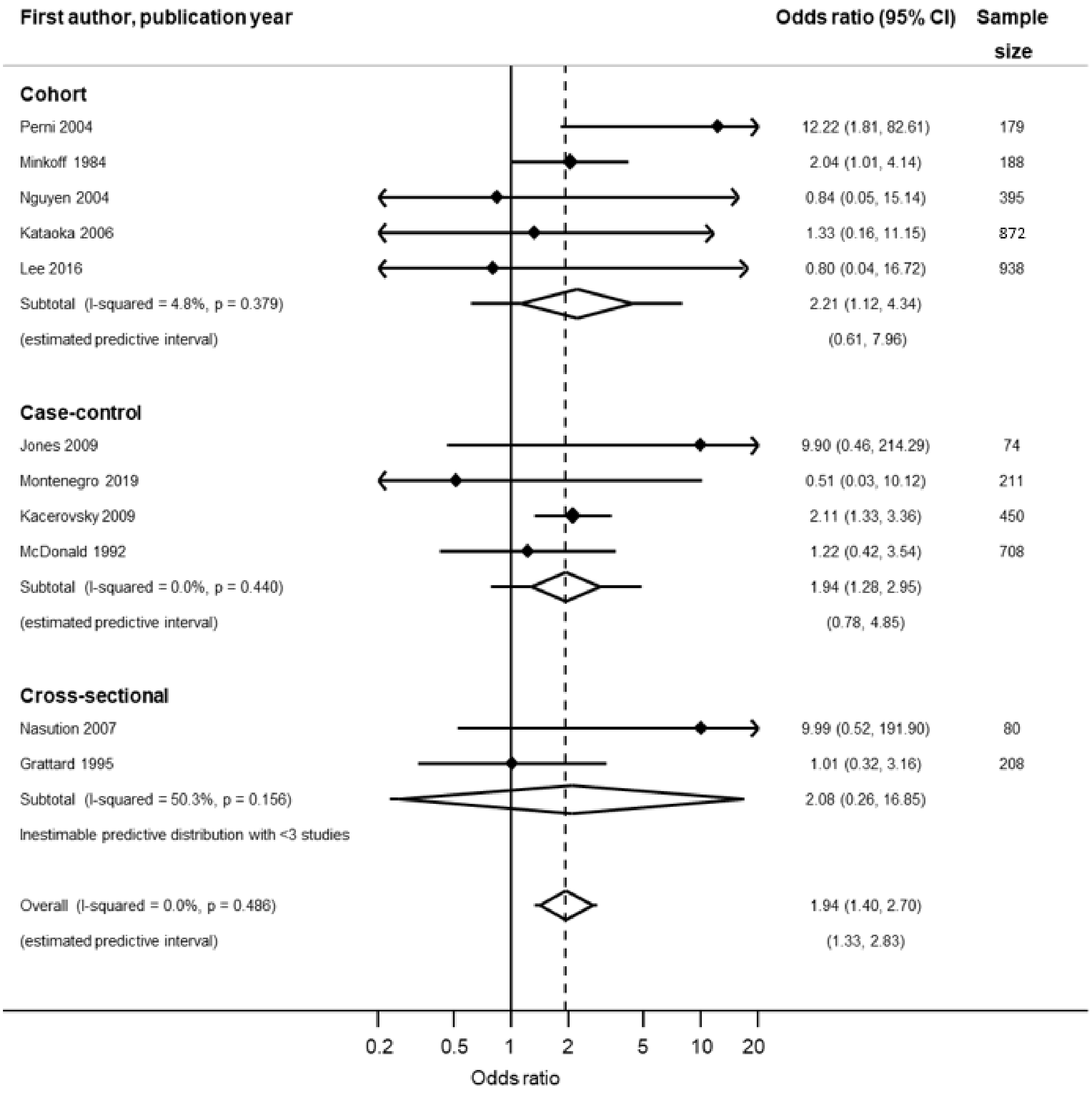

Figure S3.1 Forest plot of association between *M. hominis* and premature rupture of membrane, random effects model.

Solid diamond shows the point estimate and study weight. Solid line shows 95% confidence intervals (CI) for each study. The open diamond shows the point estimate and 95% CI of the summary estimate. Solid lines extending from the diamond show the prediction interval.

For peer review only - http://bmjopen.bmj.com/site/about/guidelines.xhtml

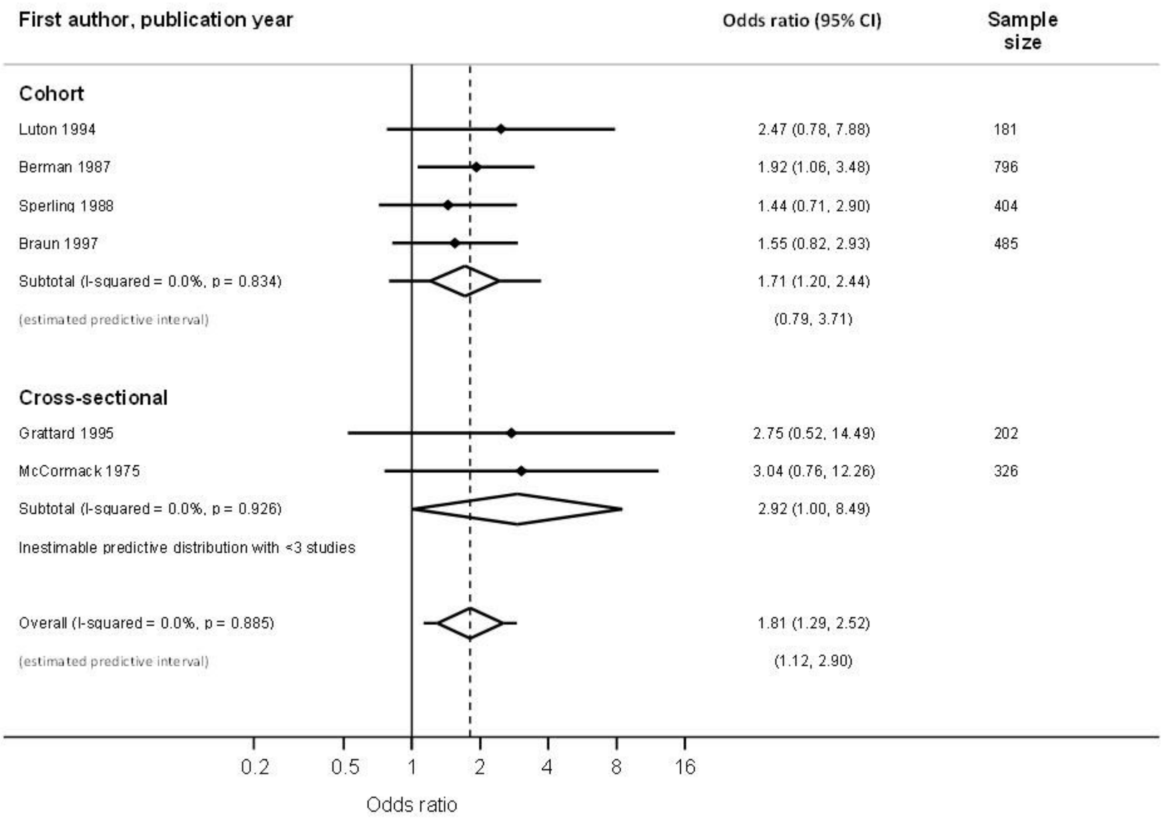

Figure S3.2 Forest plot of association between *M. hominis* and low birthweight, random effects model.

Solid diamond shows the point estimate and study weight. Solid line shows 95% confidence intervals (CI) for each study. The open diamond shows the point estimate and 95% CI of the summary estimate. Solid lines extending from the diamond show the prediction interval.

For peer review only - http://bmjopen.bmj.com/site/about/guidelines.xhtml

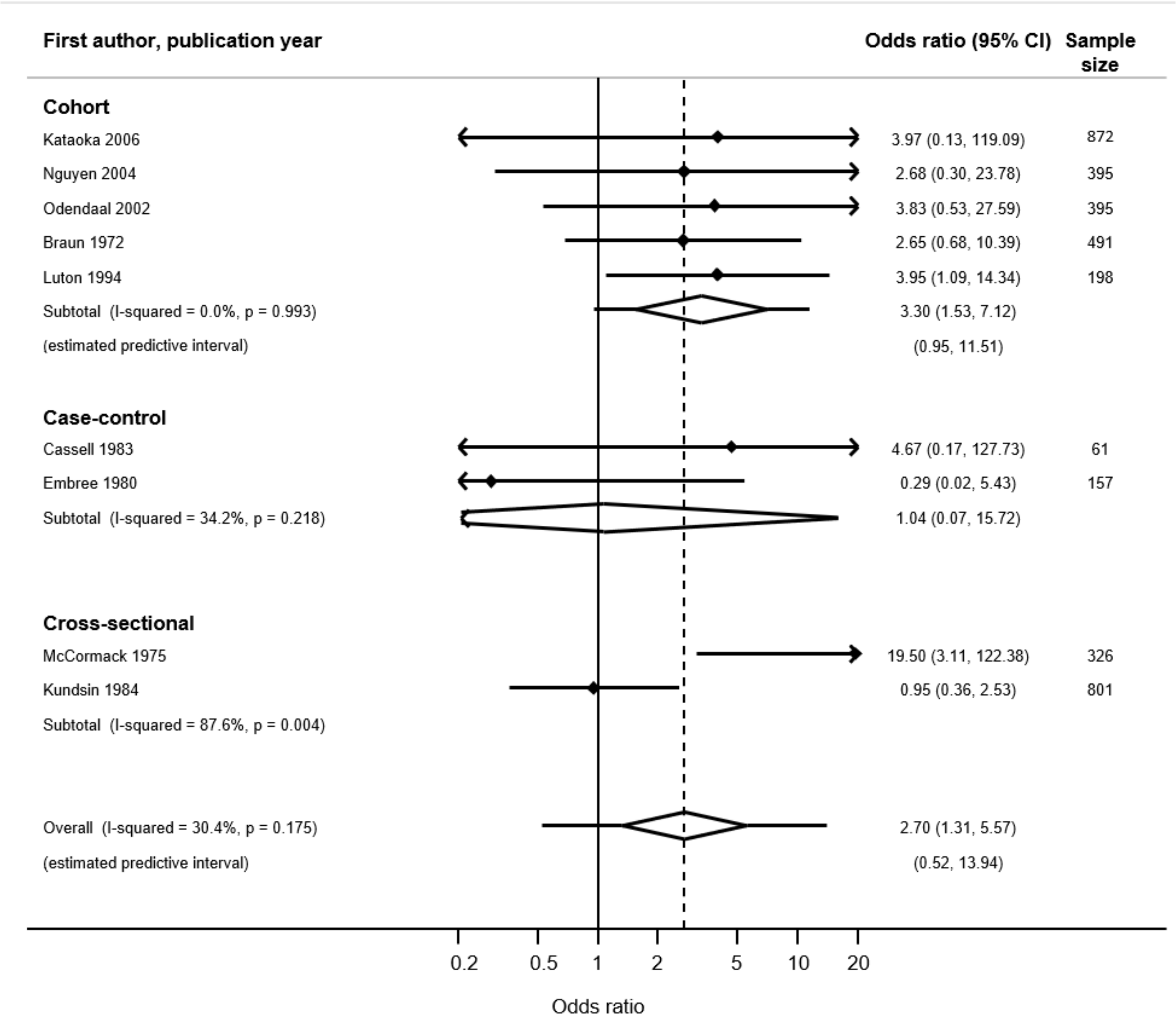

Figure S3.3 Forest plot of association between *M. hominis* and perinatal death random effects model.

Solid diamond shows the point estimate and study weight. Solid line shows 95% confidence intervals (CI) for each study. The open diamond shows the point estimate and 95% CI of the summary estimate. Solid lines extending from the diamond show the prediction interval.

Supporting information

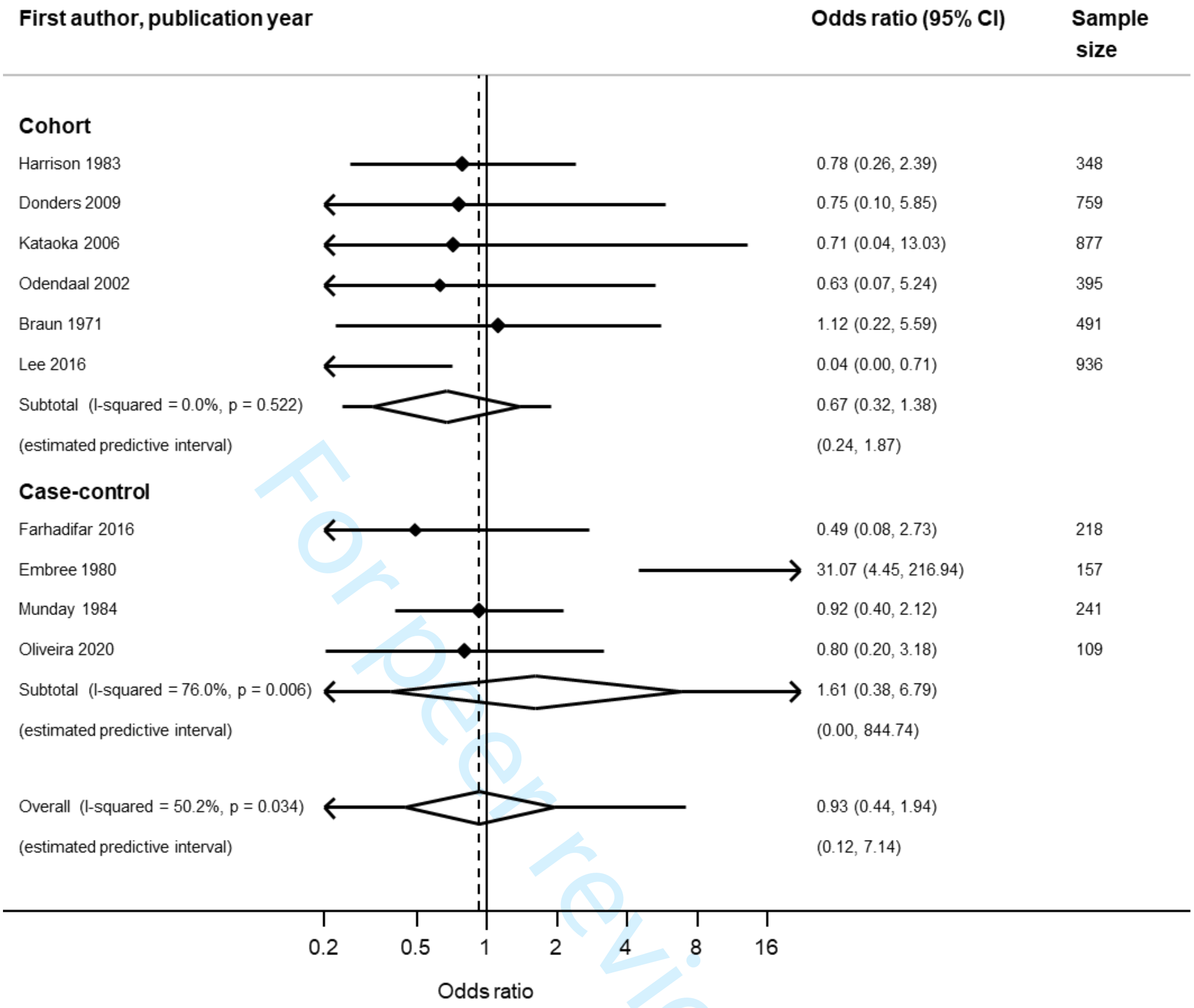

Figure S3.4 Forest plot of association between *M. hominis* and spontaneous abortion random effects model.

Solid diamond shows the point estimate and study weight. Solid line shows 95% confidence intervals (CI) for each study. The open diamond shows the point estimate and 95% CI of the summary estimate. Solid lines extending from the diamond show the prediction interval.

For peer review only - http://bmjopen.bmj.com/site/about/guidelines.xhtml
For peer review only

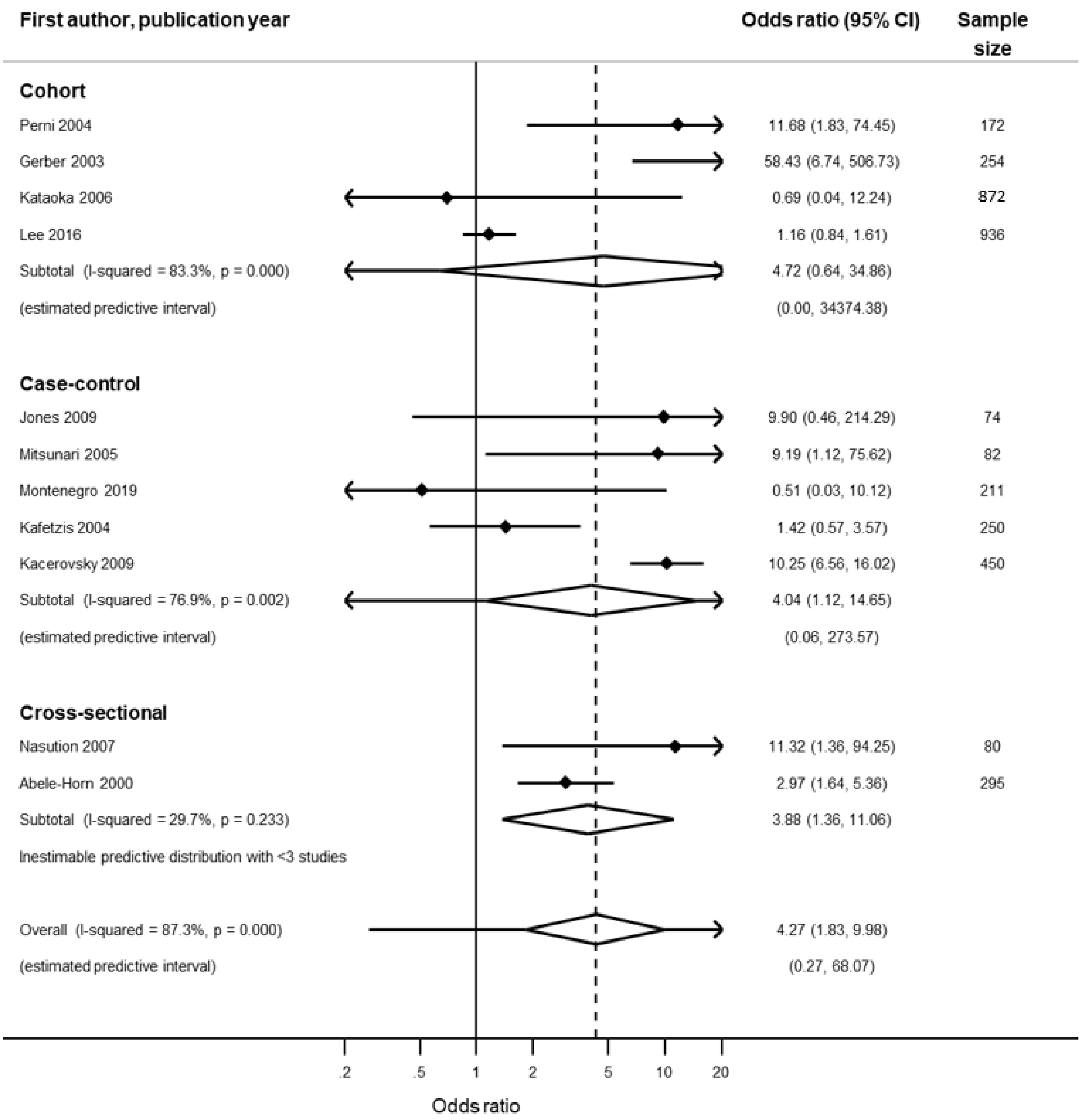

Figure S4.1 Forest plot of association between *U. urealyticum* and premature rupture of membrane, random effects model.

Solid diamond shows the point estimate and study weight. Solid line shows 95% confidence intervals (CI) for each study. The open diamond shows the point estimate and 95% CI of the summary estimate. Solid lines extending from the diamond show the prediction interval.

For peer review only - http://bmjopen.bmj.com/site/about/guidelines.xhtml

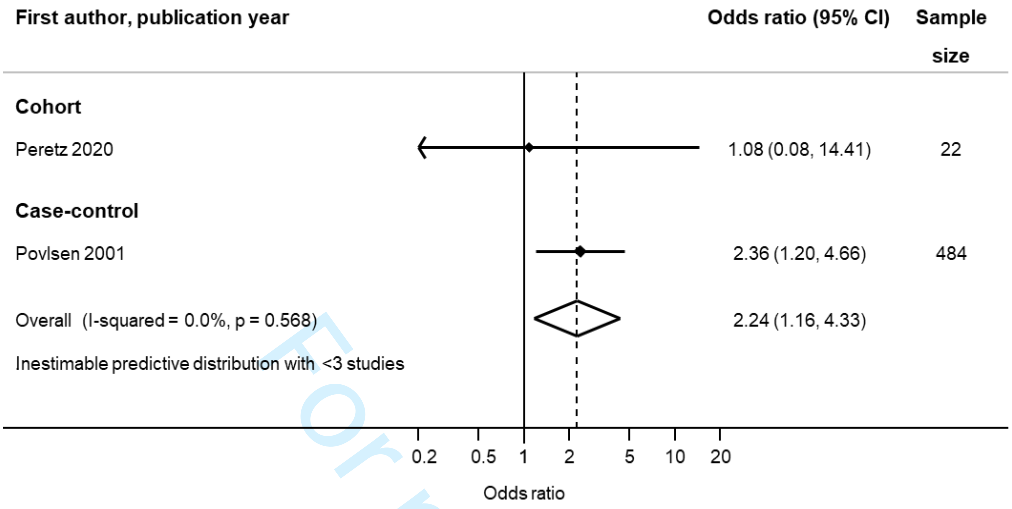

Figure S4.2 Forest plot of association between *U. urealyticum* and low birth weight, random effects model.

Solid diamond shows the point estimate and study weight. Solid line shows 95% confidence intervals (CI) for each study. The open diamond shows the point estimate and 95% CI of the summary estimate. Solid lines extending from the diamond show the prediction interval.

For peer review only

For peer review only - http://bmjopen.bmj.com/site/about/guidelines.xhtml

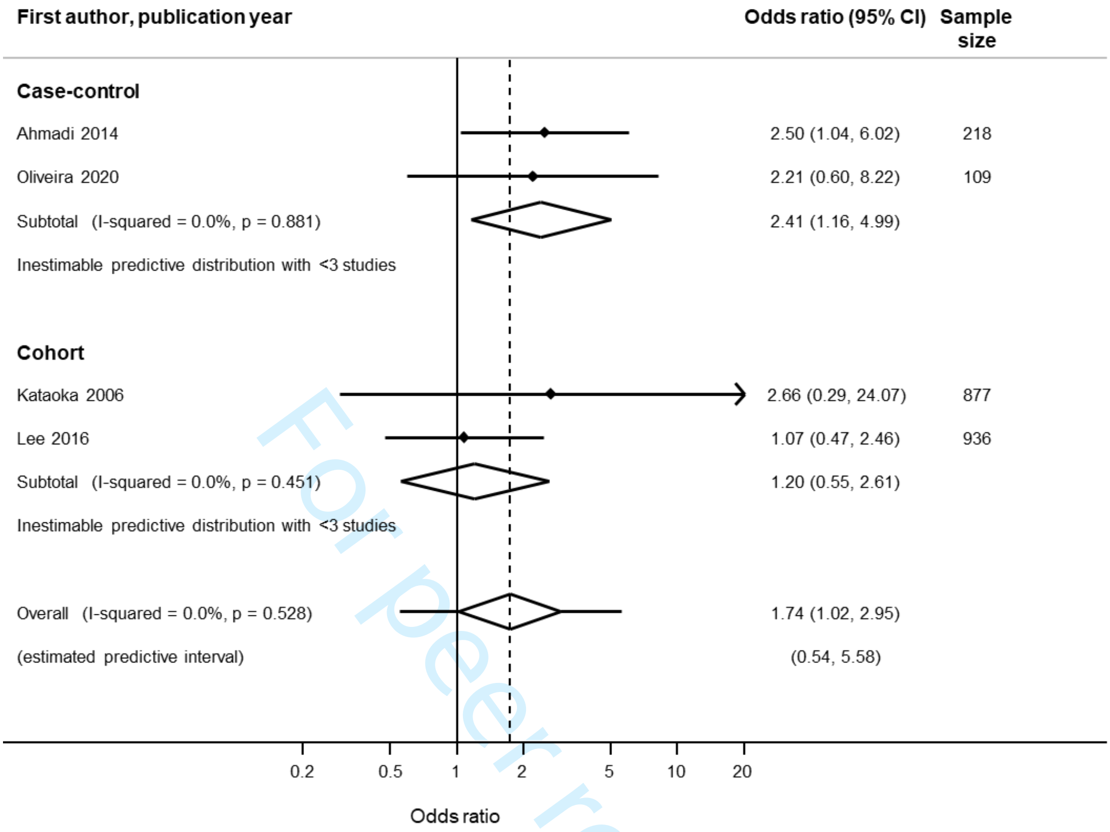

Figure S4.3 Forest plot of association between *U. urealyticum* and spontaneous abortion, random effects model

Solid diamond shows the point estimate and study weight. Solid line shows 95% confidence intervals (CI) for each study. The open diamond shows the point estimate and 95% CI of the summary estimate. Solid lines extending from the diamond show the prediction interval.

Supporting information

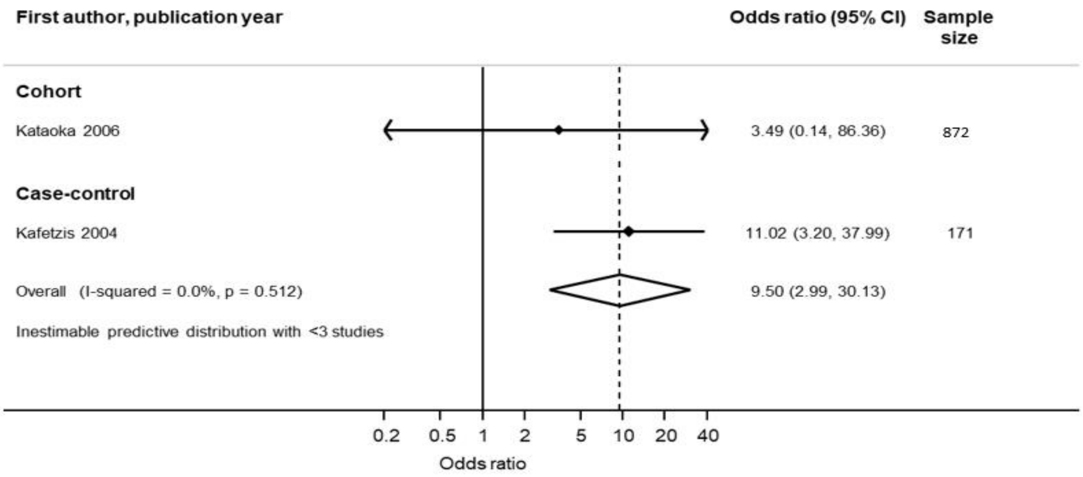

Figure S4.4 Forest plot of association between *U. urealyticum* and perinatal death, random effects model**.** Solid diamond shows the point estimate and study weight. Solid line shows 95% confidence intervals (CI) for each study. The open diamond shows the point estimate and 95% CI of the summary estimate. Solid lines extending from the diamond show the prediction interval.

For peer review only - http://bmjopen.bmj.com/site/about/guidelines.xhtml

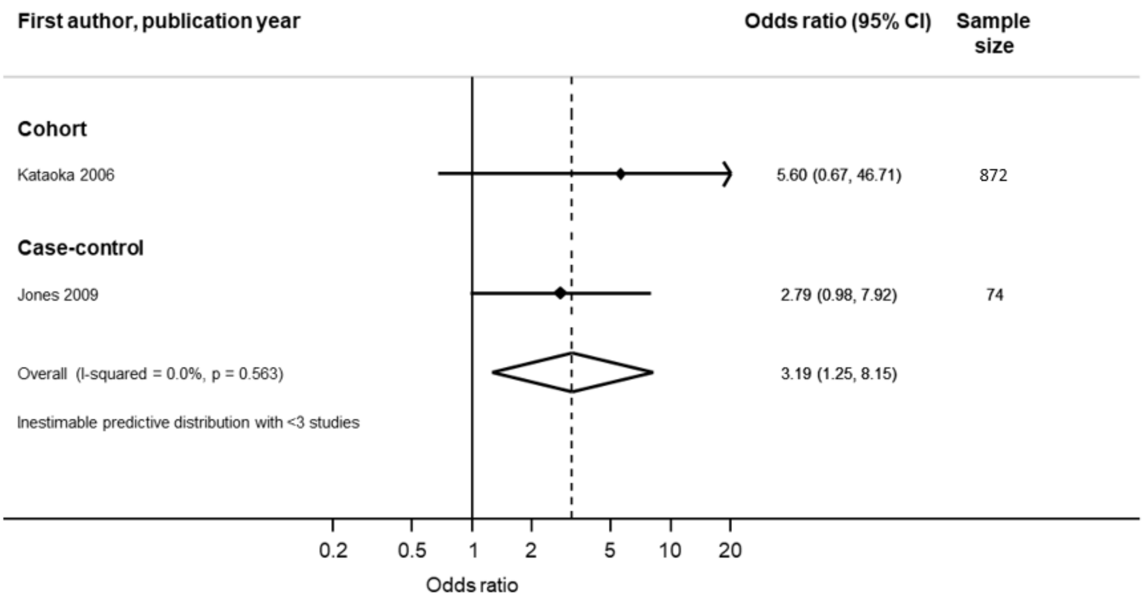

Figure S5.1 Forest plot of association between *U. parvum* and premature rupture of membrane, random effects model.

Solid diamond shows the point estimate and study weight. Solid line shows 95% confidence intervals (CI) for each study. The open diamond shows the point estimate and 95% CI of the summary estimate. Solid lines extending from the diamond show the prediction interval.

For peer review only - http://bmjopen.bmj.com/site/about/guidelines.xhtml

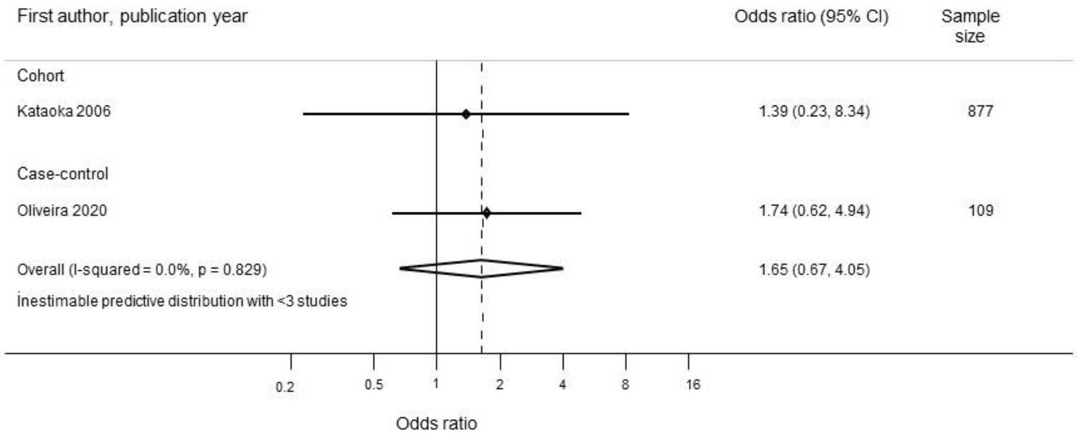

Figure S5.2 Forest plot of association between *U. parvum* and spontaneous abortion random effects model

Solid diamond shows the point estimate and study weight. Solid line shows 95% confidence intervals (CI) for each study. The open diamond shows the point estimate and 95% CI of the summary estimate. Solid lines extending from the diamond show the prediction interval.

For peer review only - http://bmjopen.bmj.com/site/about/guidelines.xhtml

Table S2.1 Descriptive tables: Cohort studies (n=26)

First author, publication year	Organism reported	Outcomes	Definition Provided	OR/ RR (95% CI) reported in by study authors		
				**Organism_ outcome**	**Unadjusted, OR**	**Adjusted, aOR**
Agger, 2014	MH, UU, UP	PTB	Born < 37 weeks	MH_PTB<br>UU_PTB<br>UP_PTB	OR 1.72 (0.91, 3.28)<br>OR 1.64 (0.67, 4.05)<br>OR 1.23 (0.7, 2.15)	'Final model... factors… from preliminary models with p>0.15.' No organism in final multivariable model for PTB <37 weeks. MH in final model PTB< 35 weeks, aOR 3.6 (1.4-9.7)
Berman, 1987	MH	LBW	<2.5kg	MH_LBW	RR 1.8 (1.0-3.1)	Birth weight as continuous variable, p=0.01, adjusted for parity, maternal height, weight, marital status, age, enrolment, gestation, *C. trachomatis*
Braun, 1971	MH	LBW<br>SA<br>PND	<2.5kg<br>Not defined<br>Not defined	NR		No multivariable analysis
Donders, 2009	MH	PTB<br>SA	Born < 37 weeks<br>**	MH_PTB	OR 8.5 (2.8, 25.5)	No multivariable analysis

Supporting information

First author, publication year	Organism reported	Outcomes	Definition Provided	OR/ RR (95% CI) reported in by study authors		
				Organism_ outcome	Unadjusted, OR	Adjusted, aOR
Gerber, 2003	UU	PTB<br>PROM	Born < 37 weeks<br>†	NR		No multivariable analysis
Govender, 2009	MH, UU, UP	PTB	Born < 37 weeks	NR		No multivariable analysis
Harrison, 1983	MH	SA	**	NR		No multivariable analysis
Hillier, 1995	MH	PTB	Born < 37 weeks	MH_PTB		No multivariable analysis
Kataoka, 2006	MH, UU, UP	PTB,<br>PROM,<br>SA,<br>PND	Born < 37 weeks<br>Not defined<br>**<br>∞	NR		No multivariable analysis
Koucky, 2016	UU	PTB	Born < 37 weeks	NR		aOR 3.4 (1.3, 5.5)<br>Adjusted for progesterone treatment, other factors not reported 5.46 (1.80, 16.62)

For peer review only - http://bmjopen.bmj.com/site/about/guidelines.xhtml

First author, publication year	Organism reported	Outcomes	Definition Provided	OR/ RR (95% CI) reported in by study authors		
				Organism_ outcome	Unadjusted, OR	Adjusted, aOR
Kwak, 2014	MH, UU	PTB	Born < 37 weeks	NR		No multivariable analysis
Lee, 2016	MH, UU	PTB	Born < 37 weeks	NR		No multivariable analysis
		PROM	Not defined			
		SA	**			
Luton, 1994	MH	PTB	Born < 37 weeks	NR		No multivariable analysis
		LBW	<2.5kg			
		PND	∞			
McDonald, 1994	MH	PTB	Born < 37 weeks	NR		No multivariable analysis
Menard, 2010	MH	PTB	Born < 37 weeks	NR		No multivariable analysis
Minkoff, 1984	MH	PTB	Born < 37 weeks	NR		Stepwise multiple logistic regression. Results for MH not reported for either outcome
		PROM	†			

Supporting information

First author, publication year	Organism reported	Outcomes	Definition Provided	OR/ RR (95% CI) reported in by study authors		
				Organism_ outcome	Unadjusted, OR	Adjusted, aOR
Nguyen, 2004	MH	PTB	Born < 37 weeks	MH_PTB	RR 4.6 (1.7, 12.8)	No multivariable analysis
		PROM	†			
		PND	Not defined			
Odendaal, 2002	MH	PTB	Born < 37 weeks	NR		No multivariable analysis
		SA,	**			
		PND	Not defined			
Payne, 2016	MH, UU, UP	PTB	Born < 37 weeks	NR		No multivariable analysis
Payne, 2020	MH, UU, UP	PTB	Born < 37 weeks	NR		No multivariable analysis
Peretz, 2020	UU, UP	PTB,	Born < 37 weeks	NR		No multivariable analysis
		LBW	<2.5kg			
Perni, 2004	MH, UU	PTB,	Born < 37 weeks	NR		No multivariable analysis
		PROM	†			

Supporting information

First author, publication year	Organism reported	Outcomes	Definition Provided	OR/ RR (95% CI) reported in by study authors		
				**Organism_ outcome**	**Unadjusted, OR**	**Adjusted, aOR**
Rittenschober-Bohm, 2018	UU, UP	PTB	Born < 37 weeks	UU_PTB<br>UP_PTB	OR 1.4 (0.9, 2.3)<br>OR 1.7 (1.3, 2.2)	aOR 1.4 (0.8, 2.2)<br>aOR 1.6 (1.2, 2.1)<br>Adjusted for age, smoking, history of PTB, BV, smoking UU or UP
Schwab, 2015	MH, UU	PTB	Born < 37 weeks	MH_PTB<br>UU_PTB	OR 0.26 (0.03, 1.13)<br>OR 0.52 (0.15, 1.57)	No multivariable analysis
Sperling, 1988	MH,	LBW	<2.5kg	NR		No multivariable analysis
Usui, 2002	MH, UU	PTB	Born < 37 weeks	NR	OR not reported by study authors	aOR 1.5 (0.8, 2.7)<br>Adjusted for *Lactobacillus* spp., *E. coli*, glucose non-fermenting Gram negative rods

Abbreviations LBW: low birth weight, defined as birth weight <2.5kg; NAAT, nucleic acid amplification tests; NR, not reported; MH, *M. hominis*; PND, perinatal mortality; PROM, premature rupture of membrane; PTB, preterm birth; SA: spontaneous abortion; UP, *U. parvum*; UU, *U. urealyticum*;

Supporting information

**SA defined as pregnancy loss at <20 weeks gestation or as defined by author; †PROM defined as rupture of membrane before 37 completed weeks or as defined by author; ∞ Perinatal death, defined as birth after 20 weeks gestation (stillbirth) or death within 28 days after birth (neonatal death) unless otherwise defined by the study authors.

Supporting information

For peer review only

Table S2.2 Descriptive tables: Case control studies (n=25)

First author, publication year	Organism reported	Outcome	Definition Provided	OR/ RR (95% CI) reported by study authors		
				**Organism outcome**	**Unadjusted, OR**	**Adjusted, aOR**
Ahmadi, 2014	UU	SA	**	NR		No multivariable analysis
Bosquet, 2006	MH	PTB	Born < 37 weeks	NR		No multivariable analysis
Cassell, 1983	MH	PTB<br>PND	Born < 37 weeks<br>∞	NR		No multivariable analysis
Chua, 1999	MH	PTB	Born < 37 weeks	NR		No multivariable analysis
Daskalakis, 2009,	MH, UU	PTB	Born < 37 weeks	NR		No multivariable analysis
Embree,1980	MH	SA<br>PND	Not defined<br>Partially defined	NR		No multivariable analysis
arhadifar, 2017	MH	SA	**	MH_SA	OR 0.49 (0.08, 2.73)	No multivariable analysis
Freitas, 2018	UU, UP	PTB	Born < 37 weeks	NR		No multivariable analysis

Supporting information

First author, publication year	Organism reported	Outcome	Definition Provided	OR/ RR (95% CI) reported by study authors		
				Organism outcome	Unadjusted, OR	Adjusted, aOR
						No multivariable analysis
Harada, 2008	UU	PTB	Not defined	NR		No multivariable analysis
Hillier, 1988	MH	PTB	Born < 37 weeks	NR		No multivariable analysis
Holst, 1994	MH	PTB	Born < 37 weeks	NR		No multivariable analysis
Jalava, 2002	UU	PTB	Born < 37 weeks	UU_PTB	RR 3.34 (1.27, 8.8)	No multivariable analysis
Jones, 2009	MH, UU, UP	PTB<br>PROM	Born < 37 weeks<br>†	NR		No multivariable analysis
Kacerovsky, 2009	MH, UU	PROM	†	NR		No multivariable analysis
Kafetzis, 2004	UU	PTB<br>PROM<br>PND	Born < 37 weeks<br>†<br>Not defined	NR		No multivariable analysis

For peer review only - http://bmjopen.bmj.com/site/about/guidelines.xhtml

First author, publication year	Organism reported	Outcome	Definition Provided	OR/ RR (95% CI) reported by study authors		
				Organism outcome	Unadjusted, OR	Adjusted, aOR
Kumar, 2006	MH	PTB	Born < 37 weeks			No multivariable analysis
McDonald, 1992	MH	PTB	Born < 37 weeks	MH_PTB	OR 1.7 (0.9, 3.5)	aOR 1.1 (0.5, 2.5)
		PROM	Not defined	MH_PROM	OR 1.5 (0.5, 4.3)	aOR 1.1 (0.3, 3.7)<br>Adjusted for ‘confounding demographic and obstetric variables’
Mitsunari, 2005	UU	PTB	Not defined	NR		No multivariable analysis
		PROM	Not defined			
Montenegro, 2019	MH, UU	PTB	Born < 37 weeks	NR		No multivariable analysis
		PROM	Not defined			
Munday, 1984	MH	SA	Not defined	NR		No multivariable analysis

Supporting information

First author, publication year	Organism reported	Outcome	Definition Provided	OR/ RR (95% CI) reported by study authors		
				Organism outcome	Unadjusted, OR	Adjusted, aOR
Oliveira, 2020	MH, UU, UP	SA	**	MH_SA<br>UU_SA<br>UP_SA	OR 0.08 (0.2, 3.17)<br>OR 2.21 (0.6, 8.22)<br>OR 1.74 (0.61, 4.93)	No multivariable analysis
Payne, 2014	UU, UP	PTB	Born < 37 weeks			NR
Povlsen, 2001	UU	PTB<br>LBW	Born < 37 weeks<br><2.5kg	UU_PTB	OR 1.0 (0.6, 1.7)	aOR 0.7 (0.4, 1.2)<br>Adjusted for LBW
Toth, 1992	MH	PTB	Born < 37 weeks	NR		No multivariable analysis
Yoon, 2001	UU	PTB<br>PROM	Born < 37 weeks<br>Not defined	NR		No multivariable analysis

Abbreviations LBW: low birth weight defined as birth weight <2.5kg; NAAT, nucleic acid amplification tests; NR, not reported; MH, *M. hominis*; PND, perinatal mortality; PROM, premature rupture of membrane; PTB, preterm birth; SA: spontaneous abortion; UP, *U. parvum*; UU, *U. urealyticum*

Supporting information

For peer review only - http://bmjopen.bmj.com/site/about/guidelines.xhtml

**SA defined as pregnancy loss at <20 weeks gestation or as defined by author; †PROM defined as rupture of membrane before 37 completed weeks or as defined by author.

Table S2.3 Descriptive tables: Cross sectional studies (n=6)

**First author, publication year**	**Organism reported**	**Total enrolled**	**Outcome**	**Definition Provided**	**OR/ RR (95% CI) reported by study authors**		
					**Organism outcome**	**Unadjusted, OR**	**Adjusted, aOR**
**Abele-Horn, 2000**	UU	295	PTB<br>PROM	Born < 37 weeks<br>†	NR	Multivariable analysis reported in text: UU >10^5 cfu/ml associated with PTB, adjusted for PROM, prolonged rupture of membranes, chorioamnionitis, obstetric risk factors	
**Grattard, 1995**	MH	208	PTB,<br>PROM<br>LBW	Born < 37 weeks<br>†<br><2.5kg	NR	No multivariable analysis	
**Kundsin, 1984**	MH	801	PND	∞	NR	No multivariable analysis	

Supporting information

For peer review only - http://bmjopen.bmj.com/site/about/guidelines.xhtml

First author, publication year	Organism reported	Total enrolled	Outcome	Definition Provided	OR/ RR (95% CI) reported by study authors		
					Organism outcome	Unadjusted, OR	Adjusted, aOR
**McCormack, 1975**	MH	327	LBW<br>PND	<2.5kg<br>Not defined	NR	No multivariable analysis	
**Nasution, 2007**	MH, UU	120	PROM	Not defined	NR	No multivariable analysis	
**Sweeney, 2016**	UU, UP	535	PTB	Born < 37 weeks	NR	No multivariable analysis	

Abbreviations LBW: low birth weight defined as birth weight <2.5kg; NAAT, nucleic acid amplification tests; NR, not reported; MH, *M. hominis*; PND, perinatal mortality; PROM, premature rupture of membrane; PTB, preterm birth; SA: spontaneous abortion; UP, *U. parvum*; UU, *U. urealyticum*

†PROM defined as rupture of membrane before 37 completed weeks or as defined by author; ∞ Perinatal death, defined as birth after 20 weeks gestation (stillbirth) or death within 28 days after birth (neonatal death) unless otherwise defined by the study authors.

Supporting information

For peer review only

Table S3.1 Summary description of studies reporting *M. hominis* (n=42), by income status

First author, Pub year, country	Gestational age assessment	Sample size for outcome of interest; Number of adverse outcomes in women with *M. hominis*/ total number of women with adverse outcome (%)					NICE checklist criteria fulfilled*
		PTB	LBW	PROM	SA	PND	
**High-income country‡**							
Agger, 2014, USA	NR	676<br>14/54 (26)					+/+
Braun, 1971, USA	LMP		485<br>24/42 (57)		491<br>3/6 (50)	491<br>7/10 (70)	+/-
Cassell, 1983, USA	US	61<br>1/10 (10)				61<br>0/3 (0)	+/-
Donders, 2009, Belgium	US	744<br>5/50 (10)			759<br>1/15 (7)		+/+
Embree, 1980, Canada	LMP, NN assessment				157<br>3/10 (30)	157<br>0/39 (0)	-/-

For peer review only - http://bmjopen.bmj.com/site/about/guidelines.xhtml

First author, Pub year, country	Gestational age assessment	Sample size for outcome of interest; Number of adverse outcomes in women with *M. hominis*/ total number of women with adverse outcome (%)					NICE checklist criteria fulfilled*
		**PTB**	**LBW**	**PROM**	**SA**	**PND**	
Grattard, 1995, France	NR	193<br>3/8 (38)	202<br>2/8 (25)	208<br>4/36 (11)			-/+
Harrison, 1983, USA	NR				348<br>4/22 (18)		-/-
Hillier, 1988, USA	US, FH, LMP	112<br>3/38 (8)					+/+
Hillier, 1995, USA	LMP	9105<br>161/423 (38)					
Holst, 1994, Sweden	US, LMP	87<br>10/22 (45)					++/+
Jones, 2009, United Kingdom	NR	74<br>2/53 (4)		74<br>2/26 (8)			-/-

Supporting information

For peer review only

First author, Pub year, country	Gestational age assessment	Sample size for outcome of interest; Number of adverse outcomes in women with *M. hominis*/ total number of women with adverse outcome (%)					NICE checklist criteria fulfilled*
		PTB	LBW	PROM	SA	PND	
Kataoka, 2006, Japan	US, LMP	872<br>4/16 (25)		872<br>1/7 (14)	877<br>0/5 (0)	872<br>0/1 (0)	+/+
Kundsin, 1984, USA	NR					801<br>5/29 (17)	-/+
Kwak, 2014, South Korea	NR	112<br>13/86 (15)					+/+
Lee, 2016, South Korea	NR	466<br>1/141 (<1)		466<br>0/187 (0)	466<br>0/11 (0)		-/-
McCormack,1975, USA	NR		326<br>3/42 (7)			326<br>2/6 (33)	+/-
McDonald, 1992, Australia	LMP, US	786<br>11/135 (8)		708<br>4/57 (8)			-/-

Supporting information

First author, Pub year, country	Gestational age assessment	Sample size for outcome of interest; Number of adverse outcomes in women with *M. hominis*/ total number of women with adverse outcome (%)					NICE checklist criteria fulfilled*
		**PTB**	**LBW**	**PROM**	**SA**	**PND**	
McDonald, 1994, Australia	US, LMP	337<br>7/45 (16)					-/-
Menard,2010, France	US, LMP	90<br>6/36 (17)					-/-
Minkoff, 1984, USA	NR	201<br>10/18 (56)		188<br>21/40 (53)			-/-
Munday, 1984, United Kingdom	NR				241<br>9/76 (12)		+/-
Nguyen,2004, Switzerland	NR	395<br>3/10 (30)		365<br>0/7 (0%)		395<br>1/6 (17)	+/+
Payne, 2016, Australia	NR	187<br>2/13 (15)					+/+

Supporting information

First author, Pub year, country	Gestational age assessment	Sample size for outcome of interest; Number of adverse outcomes in women with *M. hominis*/ total number of women with adverse outcome (%)					NICE checklist criteria fulfilled*
		PTB	LBW	PROM	SA	PND	
Payne, 2020, Australia	NR	1000<br>9/118 (8)					+/+
Sperling, 1988, USA	NR		404<br>14/37 (38)				-/-
Toth, 1992, United Kingdom	NR	80<br>3/39 (8)					-/-
Usui, 2002, Japan	LMP	1958<br>15/342 (4)					+/-
**Upper-middle income**‡							
Berman, 1987, Mexico	NR		796<br>28/48 (58)				-/+

For peer review only - http://bmjopen.bmj.com/site/about/guidelines.xhtml

First author, Pub year, country	Gestational age assessment	Sample size for outcome of interest; Number of adverse outcomes in women with *M. hominis*/ total number of women with adverse outcome (%)					NICE checklist criteria fulfilled*
		PTB	LBW	PROM	SA	PND	
Chua, 1999, Malaysia	LMP, NN assessment	120<br>9/60 (15)					+/+
Farhadifar, 2016, Iran	US/LMP				218<br>2/109 (2)		+/+
Govender, 2009, South Africa	NR	199<br>11/20 (55)					-/-
Luton, 1994, Gabon	US, LMP	181<br>11/20 (55)	181<br>8/13 (62)			198<br>5/10 (50)	-/-
Montenegro, 2019, Colombia	NR	211<br>1/84 (1)		211<br>0/3 (0)			+/+
Odendaal, 2002, South Africa	US	395<br>33/119 (28)			395<br>1/7 (14)	395<br>2/4 (50)	+/-

Supporting information

First author, Pub year, country	Gestational age assessment	Sample size for outcome of interest; Number of adverse outcomes in women with *M. hominis*/ total number of women with adverse outcome (%)					NICE checklist criteria fulfilled*
		PTB	LBW	PROM	SA	PND	
Oliveria, 2020, Brazil	NR				109<br>11/89 (12)		+/+
**Lower-middle/low income‡**							
Schwab,2015, Indonesia	LMP	62<br>2/23 (9)					-/-
Kumar, 2006, India	NR	120<br>4/60 (7)					+/+
**Country not reported**							
Gonzàlez Bosquet, 2006	US	120<br>0/70 (0)					+/+
Daskalakis, 2009	US, LMP	37<br>8/25 (32)					+/+

Supporting information

For peer review only - http://bmjopen.bmj.com/site/about/guidelines.xhtml

First author, Pub year, country	Gestational age assessment	Sample size for outcome of interest; Number of adverse outcomes in women with *M. hominis*/ total number of women with adverse outcome (%)					NICE checklist criteria fulfilled*
		PTB	LBW	PROM	SA	PND	
Kacerovsky, 2009	NR			450<br>63/225 (28)			-/-
Nasution, 2007	NR			80<br>4/40 (10)			-/-
Perni, 2004	NR	179<br>0/10 (0)		179<br>2/5 (40)			+/+

Abbreviations: MH, *Mycoplasma hominis*; UU, *Ureaplasma urealyticum*; UP, *Ureaplasma parvum*; NR, not reported.

LBW: low birth weight, defined as birth weight <2.5kg ; PTB: preterm birth, defined as delivery before 37weeks gestation; PROM: premature rupture of membranes, - defined as clinically confirmed rupture of membrane before 37weeks gestation; PND, perinatal mortality, defined as stillbirth (death after 20/40 gestation) or neonatal death (death <28 day since birth), unless otherwise defined by the study authors; SA: spontaneous abortion- defined as pregnancy loss at <20/40 or as defined by author.

Supporting information

* UK National Institute of Health and Care Excellence (NICE) checklist, summary of criteria for internal/external validity: +/+ Some checklist criteria fulfilled; -/-Few or no checklist criteria fulfilled

‡ high-income ($12,376 or more); upper-middle income ($3,996 to $1,2375); lower-middle-income ($1,025 to $3,995); low-income ($1,025 or less) [Source: World Bank, Gross national income per capita, 2019-2020 https://datatopics.worldbank.org/world-development-indicators/the-world-by-income-and-region.html]

For peer review only

Supporting information

Table S3.2 Summary description of studies reporting *U. urealyticum* (n=31), by income status

First author, pub. year, country	Gestational age assessment	Sample size for outcome of interest; Number of adverse outcomes in women with *U. urealyticum*/ total number of women with adverse outcome (%)					NICE checklist criteria fulfilled
		PTB	LBW	PROM	SA	PND	
**High-income country**‡							
Abele-Horn, 2000, Germany	US, LMP	295<br>57/ 73 (78)		295<br>58/76 (76)			-/++
Agger, 2014, USA	NR	676<br>4/54 (11)					+/+
Freitas, 2018, Canada	NR	216<br>0/46 (0)					+/+
Harada, 2008, Japan	NR	145<br>23/45 (51)					+/+

For peer review only - http://bmjopen.bmj.com/site/about/guidelines.xhtml

First author, pub. year, country	Gestational age assessment	Sample size for outcome of interest;<br>Number of adverse outcomes in women with *U. urealyticum*/ total number of women with adverse outcome (%)					NICE checklist criteria fulfilled
		**PTB**	**LBW**	**PROM**	**SA**	**PND**	
Jones, 2009, UK	NR	74<br>2/53 (4)		74<br>2/26 (8)			-/-
Kafetzis, 2004, Greece	NR	251<br>46/126 (37)		250<br>9/20 (45)		171<br>6/16 (38)	+/+
Kataoka, 2006, Japan	US, LMP	872<br>0/16 (0)		8772<br>0/7 (0)	877<br>1/5 (20)	872<br>0/1 (0)	+/+
Koucky, 2016, Czech Republic	US, LMP	63<br>17/29 (59)					+/+
Kwak, 2014, South Korea	NR	179<br>84/129 (65)					+/+
Lee, 2016, South Korea	NR	936<br>72/141 (51)		936<br>100/187 (53)	936<br>12/23 (52)		-/-

Supporting information

First author, pub. year, country	Gestational age assessment	Sample size for outcome of interest; Number of adverse outcomes in women with *U. urealyticum*/ total number of women with adverse outcome (%)					NICE checklist criteria fulfilled
		PTB	LBW	PROM	SA	PND	
Mitsunari, 2005, Japan	NR	82<br>17/21 (81)		82<br>10/11 (91)			+/+
Payne, 2014, China & Australia	US	972<br>2/13 (15)					+/+
Payne, 2016, Australia	NR	187<br>0/115 (0)					+/+
Payne, 2020, Australia	NR	1000<br>14/118 (12)					+/+
Povlsen, 2001, Denmark	NR	484<br>49/84 (58)	484<br>36/48 (75)				+/+
Peretz, 2020, Israel	NR	214<br>3/5 (60)	214<br>1/3 (33)				-/-

Supporting information

For peer review only - http://bmjopen.bmj.com/site/about/guidelines.xhtml

First author, pub. year, country	Gestational age assessment	Sample size for outcome of interest; Number of adverse outcomes in women with *U. urealyticum*/ total number of women with adverse outcome (%)					NICE checklist criteria fulfilled
		PTB	LBW	PROM	SA	PND	
Rittenschober-Böhm, 2018, Austria	US	2183<br>19/146 (13)					+/+
Sweeney, 2016, USA	NR	535<br>6/443 (1)					+/-
Usui, 2002, Japan	NR	1958<br>189/342 (55)					+/-
Yoon, 2001, South Korea	NR	114<br>3/19 (16)		Missing data<br>2/9 (22)**			+/+
**Upper-middle income**[‡]							
Ahmadi, 2014, Iran	US, LMP				218<br>18/109 (17)		+/+

Supporting information

First author, pub. year, country	Gestational age assessment	Sample size for outcome of interest; Number of adverse outcomes in women with *U. urealyticum*/ total number of women with adverse outcome (%)					NICE checklist criteria fulfilled
		**PTB**	**LBW**	**PROM**	**SA**	**PND**	
Govender,2009, South Africa	NR	199<br>5/20 (25)					-/-
Oliveiria, 2020, Brazil	NR				109<br>25/89 (28)		+/+
Montenegro, 2019, Colombia	NR	211<br>0/84 (0)		211<br>0/3 (0)			+/+
**Lower-middle income**[‡]							
Schwab, 2015, Indonesia	LMP	62<br>2/23 (9)					-/-
**Country not reported**							
Daskalakis, 2009	US, LMP	37<br>17/25 (68)					+/+

Supporting information

First author, pub. year, country	Gestational age assessment	Sample size for outcome of interest; Number of adverse outcomes in women with *U. urealyticum*/ total number of women with adverse outcome (%)					NICE checklist criteria fulfilled
		**PTB**	**LBW**	**PROM**	**SA**	**PND**	
Gerber, 2003,	NR	254<br>9/10 (90)		254<br>6/7 (86)			+/-
Jalava, 2002	NR	122<br>12/17 (71)					+/+
Kacerovsky, 2009	NR			450<br>152/225 (68)			-/-
Nasution, 2007	NR			80<br>9/40 (23)			-/-
Perni, 2004	Cohort	NR	172<br>0/10 (0)		172<br>3/5 (60)		+/+

Abbreviations: MH, *Mycoplasma hominis*; UU, *Ureaplasma urealyticum*; UP, *Ureaplasma parvum*; NR, not reported.

Supporting information

For peer review only - http://bmjopen.bmj.com/site/about/guidelines.xhtml

LBW: low birth weight, defined as birth weight <2.5kg ; PTB: preterm birth, defined as delivery before 37weeks gestation; PROM: premature rupture of membranes, - defined as clinically confirmed rupture of membrane before 37weeks gestation; PND, perinatal mortality, defined as stillbirth (death after 20/40 gestation) or neonatal death (death <28 day since birth), unless otherwise defined by the study authors; SA: spontaneous abortion- defined as pregnancy loss at <20/40 or as defined by author.

* UK National Institute of Health and Care Excellence (NICE) checklist, summary of criteria for internal/external validity: +/+ Some checklist criteria fulfilled; -/-Few or no checklist criteria fulfilled

‡high-income ($12,376 or more); upper-middle income ($3,996 to $1,2375); lower-middle-income ($1,025 to $3,995); low-income ($1,025 or less) [Source: World Bank, Gross national income per capita, 2019-2020 https://datatopics.worldbank.org/world-development-indicators/the-world-by-income-and-region.html]

Supporting information

For peer review only - http://bmjopen.bmj.com/site/about/guidelines.xhtml

Table S3.3 Summary description of studies reporting *U. parvum* (n=12), by income status

First author,<br>Pub. year, country	Study<br>design	Gestational age<br>assessment	Sample size for outcome of interest.<br>Number of adverse outcomes in women with *U. parvum*/ total<br>number of women with adverse outcome (%)<br>PTB	LBW	PROM	SA	PND	NICE checklist<br>criteria fulfilled
**Upper-middle and high-income country**[‡]								
**Agger, 2014, USA**	Cohort	NR	676<br>29/54 (54)					+/+
**Freitas, 2018, Canada**	Case-control	NR	216<br>14/46 (30)					+/+
**Govender, 2009,<br>South Africa**	Cohort	NR	199<br>10/20 (50)					-/-
**Jones, 2009,** United<br>Kingdom	Case-control	NR	74<br>19/53 (36)		74<br>11/26 (42)			-/-
**Kataoka, 2006, Japan**	Cohort	US, LMP	872<br>4/16 (25)		872<br>6/7 (86)	877<br>3/5 (60)	872<br>1/1 (100)	+/+

For peer review only - http://bmjopen.bmj.com/site/about/guidelines.xhtml

**Oliveiia, 2020, Brazil**	Case-control	NR				109<br>68/89 (76)	+/+
**Payne, 2014, China & Australia**	Case-control	NR	972<br>2/115 (2)				+/+
**Payne, 2016, Australia**	Cohort	NR	187<br>10/13 (77)				+/+
**Payne, 2020, Australia**	Cohort	NR	1000<br>56/118 (48)				+/+
**Peretz, 2020, Israel**	Cohort	NR	214<br>1/5 (20)	214<br>1/3 (33)			-/-
**Rittenschober-Böhm, 2018, Austria**	Cohort	US	3316<br>140/267 (52)				+/+
**Sweeney, 2016, USA**	Cross-sectional	NR	535<br>27/443 (4)				+/-

Abbreviations: MH, *Mycoplasma hominis*; UU, *Ureaplasma urealyticum*; UP, *Ureaplasma parvum*; NR, not reported.

Supporting information

LBW: low birth weight, defined as birth weight <2.5kg ; PTB: preterm birth, defined as delivery before 37weeks gestation; PROM: premature rupture of membranes, - defined as clinically confirmed rupture of membrane before 37weeks gestation; PND, perinatal mortality, defined as stillbirth (death after 20/40 gestation) or neonatal death (death <28 day since birth), unless otherwise defined by the study authors; SA: spontaneous abortion- defined as pregnancy loss at <20/40 or as defined by author.

* UK National Institute of Health and Care Excellence (NICE) checklist, summary of criteria for internal/external validity: +/+ Some checklist criteria fulfilled; -/-Few or no checklist criteria fulfilled

‡ high-income ($12,376 or more); upper-middle income ($3,996 to $1,2375) [Source: World Bank, Gross national income per capita, 2019-2020 https://datatopics.worldbank.org/world-development-indicators/the-world-by-income-and-region.html]

Supporting information

For peer review only - http://bmjopen.bmj.com/site/about/guidelines.xhtml

Table S4.1 Study setting and socio-demographics, cohort studies (n=26)

First author, year of publication	Location of study	Study setting	Urban/ rural location	Mean†/ median age years (range)	Ethnicity	Other infections included/ (excluded)	Smokers included (%)	Multiple pregnancies
**Agger, 2014**	USA	NR/unclear;	Mixed	NR	Mixed	CT, NG, HPV, herpes, syphilis‡	NR	Yes
**Berman, 1987**	Mexico	Health facility	NR/unclear	NR	NR	CT	NR	No
**Braun, 1971**	USA	Health facility	Urban	NR	Mixed	NR	NR	NR
**Donders, 2009**	Belgium	Health facility	Urban	29†	Mixed	BV; (CT, TV, NG, syphilis)	120/761 (15.8%)	No
**Gerber, 2003**	NR	Health facility	NR/unclear	19-42	White	NR	NR	Yes
**Govender, 2009**	South Africa	Health facility	Urban	NA	NR	HIV, CT‡	NR	NR
**Harrison, 1983**	USA	Health facility	Urban	NR	Mixed	CT	NR	Yes
**Hillier, 1995**	USA	Health facility	Urban	NR	Mixed	BV, NG, CT, TV	Yes but #/%NR	No
**Kataoka, 2006**	Japan	Health facility	Urban	28.9†	NR	CT, NG‡	NR	No

For peer review only - http://bmjopen.bmj.com/site/about/guidelines.xhtml

First author, year of publication	Location of study	Study setting	Urban/ rural location	Mean†/ median age years (range)	Ethnicity	Other infections included/ (excluded)	Smokers included (%)	Multiple pregnancies
**Koucky, 2016**	Czech Republic	Health facility	Urban	31	NR	NR	NR	No
**Kwak, 2014**	South Korea	Health facility	Urban	30.7	NR	NR	NR	No
**Lee, 2016**	South Korea	Health facility	Urban	31 (15-47)	NR	NR	NR	NR
**Luton, 1994**	Gabon	Health facility	NR/unclear	NR	NR	HIV, CT, TV, NG, Syphilis	NR	No
**Minkoff, 1984**	USA	Health facility	NR	27† (17-39)	Mixed	CT, TV	NR	Yes
**McDonald, 1994**	Australia	Health facility	NR	NR	NR	NR	NR	NR
**Menard, 2010**	France	Health facility	Urban	NR	NR	BV	NR	No
**Nguyen, 2004**	Switzerland	Health facility	Urban	19-42	NR	NR	NR	No
**Odendaal, 2002**	South Africa	Health facility	Urban	NR	NR	CT, BV, NG	161/395 (40.8%)	No

Supporting information

First author, year of publication	Location of study	Study setting	Urban/ rural location	Mean†/ median age years (range)	Ethnicity	Other infections included/ (excluded)	Smokers included (%)	Multiple pregnancies
**Payne, 2016**	Australia	Health facility	Urban	30 (18-43)	Mixed	NR	21/191 (11%)	No
**Payne, 2020**	Australia	Health facility	Urban	NR	Mixed	(HIV) ‡	135/ 1000 (13.5%)	No
**Peretz, 2020**	Israel	Health facility	Urban	29.8†	Mixed	NR ‡	NR	Yes
**Perni, 2004**	NR	Health facility	NR/unclear	18-44	Mixed	NR	NR	No
**Rittenschober-Böhm, 2018**	Austria	Health facility	Urban	30.3†	NR	BV	670/3643 (18.4%)	No
**Schwab, 2015**	Indonesia	Health facility	Urban	26.6† (17-42)	NR	CT, BV, NG	NR	NR
**Sperling, 1988**	USA	Health facility	Urban	NR	Mixed	NR	NR	NR
**Usui, 2002**	Japan	Health facility	Urban	NR	Asian	CT	NR	No

Abbreviations: BV, bacterial vaginosis; CT, *Chlamydia trachomatis*; HIV, human immunodeficiency virus; NG, *Neisseria gonorrhoeae*; NR, not reported; infections; TV, *Trichomonas vaginalis*; USA, United States of America;

†reported mean age; ‡Detected *Mycoplasma genitalium*

Supporting information

Table S4.2 Study setting and socio-demographics, case-control studies (n=25)

First author, year of publication	Location of study	Study setting	Urban /rural location	Mean†/ median age years (range)	Ethnicity	Other infections included (or excluded)	Smokers included/ (%)	Multiple pregnancies
**Ahmadi, 2014**	Iran	Health facility	Urban	19-43	NR	NR	3/218 (1.4)	NR
**Gonzàlez Bosquet, 2006**	NR	Health facility	NR/ unclear	NR	NR	CA, BV-associated bacteria, *E. coli*, GBS, TV,	NR	No
**Cassell, 1983**	USA	Health facility	Urban	NR	White, Black	NR	NR	NR
**Chua, 1999**	Malaysia	Health facility	Urban	NR	NR	NR	NR	No
**Daskalakis, 2009**	NR	Health facility	Urban	NR	NR	NR	36/144 (25)	No
**Embree,**	Canada	Health facility	Urban	14-45	NR	NR	NR	Yes
**Farhadifar, 2016**	Iran	Health facility	Urban	25 (19-43)	NR	NR	NR	NR
**Freitas, 2018**	Canada	Health facility	Urban	33† (21-45)	Mixed	NR	4/216 (2.3%)	NR
**Harada, 2008**	Japan	Health facility	Urban	NR	NR	NR	NR	No

For peer review only - http://bmjopen.bmj.com/site/about/guidelines.xhtml

**Hillier, 1988**	USA	Health facility	Urban	NR	NR	CT, TV, BV	NR	No
**Holst, 1994**	Sweden	Health facility	Urban	NR	NR	CT, BV, NG	20/49 (40.8)	No
**Jalava, 2002**	NR	Health facility	NR/ unclear	NR	NR	(CT)	NR	NR
**Jones, 2009**	United Kingdom	Health facility	Urban	NR	NR	NR	NR	No
**Kacerovsky, 2009**	NR	Health facility	NR/ unclear	26 (19-38)	NR	NR	NR	No
**Kafetzis, 2004**	Greece	Health facility	Urban	NR	NR	NR	NR	NR
**Kumar, 2006**	India	Health facility	Urban	NR	NR	BV	NR	NR
**McDonald, 1992**	Australia	Health facility	Urban	NR	NR	NR	839/ 2190 (39.8%)	NR
**Mitsunari, 2005**	Japan	Health facility	Urban	NR	Asian	(CT)	NR	No
**Montenegro, 2019**	Colombia	Health facility	Urban	NR	NR	NR	NR	NR
**Munday, 1984**	United Kingdom	Health facility	Urban	NR	Mixed	CT	NR	NR

Supporting information

**Oliveira, 2020**	Brazil	Health facility	Urban	27.3	Mixed	NG ‡	5/109 (4.6)	NR
**Payne, 2014**	China, Australia	Health facility	NR/unclear	17-49	Mixed	NR	69/972 (7.1%)	No
**Povlsen, 2001**	Denmark	Health facility	Urban	NR	NR	BV	NR	No
**Toth, 1992**	United Kingdom	Health facility	Urban	NR	NR	CT, TV	NR	NR
**Yoon, 2001***	South Korea	Health facility	Urban	NR	NR	NR	NR	No

Abbreviations: BV, bacterial vaginosis; CA, *Candida albicans*; CT, *Chlamydia trachomatis*; HIV, human immunodeficiency virus; NG, *Neisseria gonorrhoeae*; NR, not reported; TV, *Trichomonas vaginalis*; USA, United States of America

† Reported mean age; ‡detected *Mycoplasma genitalium*

For peer review only

Supporting information

Table S4.3 Study setting and socio-demographics, cross-sectional studies (n=6)

First author, year of publication	Location of study	Study setting	Urban /rural location	Mean†/ median age years (range)	Ethnicity	Other infections included (or excluded)	Smokers included/ (%)	Multiple pregnancies
**Abele-Horn, 2000**	Germany	Health facility	Urban	NR	Mixed	(BV, CT, NG, TV, yeast)	NR	NR
**Grattard, 1995**	France	Health facility	Urban	NR	NR	NR	NR	NR
**Kundsin, 1984**	USA	Health facility	Urban	NR	Mixed	NR	105/801 (31.4%)	Yes
**McCormack, 1975**	USA	Health facility	Urban	23.6†	Mixed	NR	NR	Yes
**Nasution, 2007**	NR	Health facility	NR/ unclear	24-38	Asian	CT, NG	NR	NR
**Sweeney, 2016**	USA	Health facility	Urban	NR	Mixed	NR	NR	Yes

Abbreviations: BV, bacterial vaginosis; CT, *Chlamydia trachomatis*; HIV, human immunodeficiency virus; NG, *Neisseria gonorrhoeae*; NR, not reported; TV, *Trichomonas vaginalis*; UK, United Kingdom; USA, United States of America

† Reported mean age

For peer review only - http://bmjopen.bmj.com/site/about/guidelines.xhtml

Table S5 Studies that reported on bacterial vaginosis or sexually transmitted infections and reported associations with adverse birth outcomes

First author, publication year, reference number*	Study population	Organism/ outcome	OR (95% CI)	Reported associations with genital mycoplasmas
**Donders, 2009**[34]	Cohort study: 759 women; 55 PTB; 64 BV; 14 *M. hominis*	BV/PTB	2.43 (1.1, 4.7)	Association between lactobacilli and PTB, and between BV and PTB reported as primary analysis. Proportion of women with *M. hominis* but no BV reported (0.5% of 759), but association between *M. hominis* and PTB in absence of BV could not be calculated from data presented. Discussion includes, "In the literature, the presence of *M. hominis* has generally been related to an increased risk of miscarriage, and premature delivery if found in combination with bacterial vaginosis."
**Hillier, 1988**[57]	Case-control study: 94 women; 38 PTB; 28	BV/PTB	3.31 (1.20, 9.24)	Association between organisms in chorioamnion and PTB reported as primary analysis. BV measured in vaginal

For peer review only - http://bmjopen.bmj.com/site/about/guidelines.xhtml

First author, publication year, reference number*	Study population	Organism/ outcome	OR (95% CI)	Reported associations with genital mycoplasmas
	BV; 29 *U. urealyticum*; 5 *M. hominis*			smears. Association between genital mycoplasmas and PTB in absence of BV could not be calculated from data presented.
**Hillier, 1995**[31]	Cohort study: 9105 women; 423 PTB; 1392 BV; 2805 *M. hominis*	BV/ PTB<br>MH+, BV+/ PTB<br>MH+, BV-/PTB	1.60 (1.25, 2.03)<br>1.58 (0.94, 2.77)<br>1.18 (0.91, 1.52)	Association between BV and PTB of low birthweight infants reported as primary analysis. Raw data not available for association between *M. hominis* and PTB, but reported in text and can be extracted from bar chart of ORs for PTB, stratified by *M. hominis*, bacteroides and BV. OR for BV and BV with *M. hominis* similar, and stronger than association for *M. hominis* alone.

For peer review only - http://bmjopen.bmj.com/site/about/guidelines.xhtml

First author, publication year, reference number*	Study population	Organism/ outcome	OR (95% CI)	Reported associations with genital mycoplasmas
**Kumar, 2006**[71]	Case-control study: 120 women; 60 PTB; 31 BV; 6 *M. hominis*	BV/PTB	5.05 (1.97, 12.95)	Association between BV and PTB reported as primary analysis. Association between *M. hominis* and PTB in absence of BV could not be calculated from data presented. Discussion does not mention *M. hominis.*
**Menard, 2010**[41]	Cohort study: 90 women; 36 PTB; 2 BV; 10 *M. hominis*			Association between quantities of BV-associated bacteria and PTB reported as primary analysis. Association between *M. hominis* and PTB in absence of BV could not be calculated from data presented.
**Odendaal, 2002**[50]	Cohort study as sub-study of a randomised controlled trial: 395	MH/BV	10.21 (5.63, 18.65)	Association between *M. hominis* and PTB reported as primary analysis. Association between *M. hominis* and BV reported, but not association between BV and PTB. Discussion includes, "It is also possible that the BV is not

For peer review only - http://bmjopen.bmj.com/site/about/guidelines.xhtml

First author, publication year, reference number*	Study population	Organism/ outcome	OR (95% CI)	Reported associations with genital mycoplasmas
	women; 119 PTB; 132 BV; 83 *M. hominis*			directly involved in the causation of premature labour but that it is only a marker of a more important underlying condition such as *M. hominis* infection,"
**Povlsen, 2001**[64]	Nested case-control study: 484 women; 84 PTB; 70 BV; 280 *U. urealyticum*	BV/PTB<br>UU+, BV+/PTB<br>UU+/PV-/PTB	0.77 (0.33, 1.6)<br>0.47 (0.09, 3.31)<br>1.15 (0.67, 1.98)	Associations between *U. urealyticum* biovars and PTB reported as primary analysis. Numbers, stratified by BV status and low birth weight reported. Discussion mentions that *U. urealyticum* and BV were associated with each other overall, but that this association was only seen in women who delivered at term and was not associated with PTB.
**Rittenschober-Bohm, 2018**[46]	Cohort study: 3,643 women; 292 PTB; 279	BV/PTB<br><br>UP-,BV-/PTB	Crude 1.7 (1.3, 2.2)<br>Adjusted 1.6 (1.1, 2.4)	Associations between *Ureaplasma* spp. and PTB reported as primary analysis. Associations with *U. parvum*, stratified by BV status and adjusted for maternal age, diagnosis of vaginal

For peer review only - http://bmjopen.bmj.com/site/about/guidelines.xhtml

First author, publication year, reference number*	Study population	Organism/ outcome	OR (95% CI)	Reported associations with genital mycoplasmas
	BV; 1,347 *U. parvum*; 214 *U. urealyticum*	UP+,BV-/PTB<br>UP-,BV+/PTB<br>UP+,BV+/PTB	Adjusted 1.6 (1.2, 2.1)<br>Adjusted 1.6 (1.1, 2.3)<br>Adjusted 2.6 (1.7, 4.0)	candida, smoking and history of previous PTB. Stratified associations with *U. urealyticum* not reported on basis of univariable analysis (OR 1.4, 95% CI 0.8, 2.2). Discussion does not mention potential associations between both BV and *Ureaplasma* spp.
**Schwab, 2015**[52]	Cohort study: 62 women; 23 PTB; 13 BV; 13 *M. hominis*; 22 *U. urealyticum*	None reported		Descriptive study of infections in pregnancy. Association between *M. hominis*, *U. urealyticum* and PTB reported, but not association between BV and PTB.

Abbreviations: BV, bacterial vaginosis; CI, confidence interval; *M. hominis*, *Mycoplasma hominis*; PTB, premature birth; *U. parvum*, *Ureaplasma parvum*; *U. urealyticum*, *Ureaplasma urealyticum.*

* Study reference is the reference number cited in the main manuscript

Supporting information

For peer review only - http://bmjopen.bmj.com/site/about/guidelines.xhtml

Table S6.1 Risk of bias assessment, cohort studies (n=26)

Questions	Agger, 2014	Berman, 1987	Braun, 1971	Donders, 2009	Gerber, 2003
1) The method of allocation to exposure groups was unrelated to potential confounding factors	NA	NA	NA	NA	NA
2) Attempts made within design or analysis to balance both groups for potential confounders.	Yes	Unclear	Unclear	Unclear	Unclear
3) The groups were comparable at baseline, including all major confounding factors.	No	Yes	Unclear	Yes	Unclear
4) Based on above answers, was selection bias present?	No	Low	Unclear	Unclear	Unclear
5) If so, what is the likely direction of its effect?	Unclear	Unclear	Unclear	Unclear	Unclear
6) The comparison groups received the same care and support apart from the exposure(s) studied.	Yes	Unclear	Unclear	Yes	Unclear
7) Participants receiving care and support were kept 'blind' to intervention allocation.	NA	NA	NA	NA	NA

For peer review only - http://bmjopen.bmj.com/site/about/guidelines.xhtml

Questions	Agger, 2014	Berman, 1987	Braun, 1971	Donders, 2009	Gerber, 2003
8) Individuals administering care, support were kept ‘blind’ to intervention allocation.	NA	NA	NA	NA	NA
9) Based on above answers, was performance bias present?	Unclear	Unclear	Unclear	Unclear	Unclear
10) If so, what is the likely direction of its effect?	Unclear	Unclear	Unclear	Unclear	Unclear
11) All groups followed up for an equal length of time?	Low	Low	Low	Low	Low
12) Number of participants did not complete the intervention in each group?	NA	NA	NA	NA	NA
13) The groups were comparable for intervention completion.	NA	NA	Unclear	NA	NA
14) For how many participants were no outcome data available?‡	107/783, (13.7%)	104/1204 (8.6%)	203/688 (30%)	42/801 (5.2%)	63/317 (19.9%)
15) Were groups comparable for outcome data?	Unclear	Unclear	Unclear	Yes	Unclear
16) Based on above answers, was attrition bias present?	Unclear	Unclear	Unclear	Unclear	Unclear
17) If so, what is the likely direction of its effect?	Unclear	Unclear	Unclear	Unclear	Unclear
18) The study had an appropriate length of follow-up.	Yes	Yes	Yes	Yes	Yes
19) The study used a precise definition of outcome.	Yes	Unclear	No	Yes	Yes

Supporting information

Questions	Agger, 2014	Berman, 1987	Braun, 1971	Donders, 2009	Gerber, 2003
20) A valid, reliable method used to determine the outcome?	Unclear	Unclear	No	Yes	Yes
21) Investigators were kept 'blind' to participants' exposure to the intervention.	NA	NA	NA	NA	NA
22) Investigators were kept 'blind' to other important confounding factors.	Unclear	Unclear	Unclear	Unclear	Unclear
23) Based on above answers, was detection bias present?	Unclear	Unclear	Unclear	No	Unclear
24) If so, what is the likely direction of its effect?	Unclear	Unclear	Unclear	NA	Unclear
**25) Overall assessment of internal validity[a]**	+	-	+	+	+
**26) Overall assessment of external validity[a]**	+	+	-	+	-

Abbreviations: High, high risk of bias; Low, low risk of bias; NA, not applicable; Unclear, unclear of risk of bias;

‡, both groups combined unless stated; .[a]++, all or most of checklist criteria fulfilled; + some of checklist criteria fulfilled; - few or no checklist criteria fulfilled.

For peer review only - http://bmjopen.bmj.com/site/about/guidelines.xhtml

Table S6.1 Risk of bias assessment, cohort studies (n=26) (continued)

Questions	Govender, 2009	Harrison, 1983	Hillier, 1995	Kataoka, 2006	Koucky, 2016
1) The method of allocation to exposure groups was unrelated to potential confounding factors	NA	NA	NA	NA	NA
2) Attempts made within design or analysis to balance both groups for potential confounders.	Unclear	Unclear	Unclear	Yes	Unclear
3) The groups were comparable at baseline, including all major confounding factors.	Unclear	Unclear	Unclear	No	Unclear
4) Based on above answers, was selection bias present?	Unclear	Unclear	Unclear	Unclear	Unclear
5) If so, what is the likely direction of its effect?	Unclear	Unclear		Unclear	Unclear
6) The comparison groups received the same care and support apart from the exposure(s) studied.	Unclear	Yes	Yes	Yes	Yes
7) Participants receiving care and support were kept 'blind' to intervention allocation.	NA	NA	NA	NA	NA

For peer review only - http://bmjopen.bmj.com/site/about/guidelines.xhtml

Questions	Govender, 2009	Harrison, 1983	Hillier, 1995	Kataoka, 2006	Koucky, 2016
8) Individuals administering care and support were kept 'blind' to intervention allocation.	NA	NA	NA	NA	NA
9) Based on above answers, was performance bias present?	Unclear	Unclear	Unclear	Unclear	Unclear
10) If so, what is the likely direction of its effect?	Unclear	Unclear	Unclear	Unclear	Unclear
11) All groups followed up for an equal length of time?	Low	Low	Low	Low	Low
12) Number of participants did not complete the intervention in each group?	NA	NA	Na	NA	NA
13) The groups were comparable for intervention completion.	NA	NA	NA	NA	NA
14) For how many participants were no outcome data available?‡	0/199 (0%)	SA (13/361), 3.6%; PND (0/467, 0%)	1292/10397 (12.4%)	163/1040 (15.7%)	0/36 (0%)
15) Were groups comparable for outcome data?	Unclear	Unclear	No	Unclear	Yes
16) Based on above answers, was attrition bias present?	Unclear	Unclear	Unclear	Unclear	Unclear
17) If so, what is the likely direction of its effect?	Unclear	Unclear	Unclear	Unclear	Unclear
18) The study had an appropriate length of follow-up.	Yes	Yes	Yes	Yes	Yes

Supporting information

Questions	Govender, 2009	Harrison, 1983	Hillier, 1995	Kataoka, 2006	Koucky, 2016
19) The study used a precise definition of outcome.	Yes	No	Yes	Yes	Yes
20) A valid, reliable method used to determine the outcome?	Unclear	Unclear	No	Yes	Yes
21) Investigators were kept 'blind' to participants' exposure to the intervention.	NA	NA	NA	NA	NA
22) Investigators were kept 'blind' to other important confounding factors.	Unclear	Unclear	Unclear	Unclear	Unclear
23) Based on above answers, was detection bias present?	Unclear	Unclear	Unclear	No	Unclear
24) If so, what is the likely direction of its effect?	Unclear	Unclear	Unclear	NA	Unclear
**25) Overall assessment of internal validity[a]**	-	-	-	+	+
**26) Overall assessment of external validity[a]**	-	-	-	+	+

Abbreviations: High, high risk of bias; Low, low risk of bias; NA, not applicable; Unclear, unclear of risk of bias;

‡, both groups combined unless stated; [a]++, all or most of checklist criteria fulfilled; + some of checklist criteria fulfilled; - few or no checklist criteria fulfilled.

Supporting information

Table S6.1 Risk of bias assessment, cohort studies (n=26) (continued)

Questions	Kwak, 2014	Lee, 2016	Luton, 1994	McDonald, 1994	Menard, 2010
1) The method of allocation to exposure groups was unrelated to potential confounding factors	NA	NA	NA	NA	NA
2) Attempts made within design or analysis to balance both groups for potential confounders.	Unclear	Unclear	Yes	Yes	Unclear
3) The groups were comparable at baseline, including all major confounding factors.	Unclear	Unclear	Yes	Unclear	Unclear
4) Based on above answers, was selection bias present?	Unclear	Unclear	Low	Unclear	Unclear
5) If so, what is the likely direction of its effect?	Unclear	Unclear	Unclear	Unclear	Unclear
6) The comparison groups received the same care and support apart from the exposure(s) studied.	Unclear	Unclear	Yes	Unclear	Yes
7) Participants receiving care and support were kept 'blind' to intervention allocation.	NA	NA	NA	NA	NA
8) Individuals administering care and support were kept 'blind' to intervention allocation.	NA	NA	NA	NA	NA
9) Based on above answers, was performance bias present?	Unclear	Unclear	Unclear	Unclear	Unclear

Supporting information

Questions	Kwak, 2014	Lee, 2016	Luton, 1994	McDonald, 1994	Menard, 2010
10) If so, what is the likely direction of its effect?	Unclear	Unclear	Unclear	Unclear	Unclear
11) All groups followed up for an equal length of time?	Low	Low	Low	Low	Low
12) Number of participants did not complete the intervention in each group?	NA	NA	NA	NA	NA
13) The groups were comparable for intervention completion.	NA	NA	NA	NA	NA
14) For how many participants were no outcome data available?‡	0 (0%)	0/1035 (0%)	37/218 (17%)	Control 182/649, (28%); Cases 42/135 (31%)	0 (0%)
15) Were groups comparable for outcome data?	Yes	Yes	No	Unclear	Yes
16) Based on above answers, was attrition bias present?	Low	Low	Unclear	Unclear	Unclear
17) If so, what is the likely direction of its effect?	Unclear	Unclear	Unclear	Unclear	Unclear
18) The study had an appropriate length of follow-up.	Yes	Yes	Yes	Yes	Yes
19) The study used a precise definition of outcome.	Yes	No	Yes	Yes	Yes
20) A valid, reliable method used to determine the outcome?	Yes	No	Yes	No	Yes

For peer review only - http://bmjopen.bmj.com/site/about/guidelines.xhtml

Questions	Kwak, 2014	Lee, 2016	Luton, 1994	McDonald, 1994	Menard, 2010
21) Investigators were kept ‘blind’ to participants’ exposure to the intervention.	NA	NA	NA	Unclear	NA
22) Investigators were kept ‘blind’ to other important confounding factors.	Unclear	Unclear	Unclear	Unclear	NA
23) Based on above answers, was detection bias present?	No	Yes	Unclear	Unclear	No
24) If so, what is the likely direction of its effect?	NA	Unclear	Unclear	Unclear	NA
**25) Overall assessment of internal validity**[a]	+	-	-	-	-
**26) Overall assessment of external validity**[a]	+	-	-	-	-

Abbreviations: High, high risk of bias; Low, low risk of bias; NA, not applicable;Unclear, unclear of risk of bias.

‡, both groups combined unless stated; [a]++, all or most of checklist criteria fulfilled; + some of checklist criteria fulfilled; - few or no checklist criteria fulfilled.

For peer review only - http://bmjopen.bmj.com/site/about/guidelines.xhtml

Table S6.1 Risk of bias assessment, cohort studies (n=26) (continued)

**Questions**	**Minkoff, 1984**	**Nguyen, 2004**	**Odendaal, 2002**	**Payne, 2016**	**Payne, 2020**
1) The method of allocation to exposure groups was unrelated to potential confounding factors	NA	NA	NA	NA	NA
2) Attempts made within design or analysis to balance both groups for potential confounders.	Unclear	Unclear	Unclear	Unclear	No
3) The groups were comparable at baseline, including all major confounding factors.	Unclear	Unclear	Unclear	Unclear	Unclear
4) Based on above answers, was selection bias present?	Unclear	Unclear	Unclear	Unclear	Low
5) If so, what is the likely direction of its effect?	Unclear	Unclear	Unclear	Unclear	Unclear
6) The comparison groups received the same care and support apart from the exposure(s) studied.	Unclear	Unclear	Yes	Unclear	Yes
7) Participants receiving care and support were kept ‘blind’ to intervention allocation.	NA	NA	NA	NA	NA

For peer review only - http://bmjopen.bmj.com/site/about/guidelines.xhtml

Questions	Minkoff, 1984	Nguyen, 2004	Odendaal, 2002	Payne, 2016	Payne, 2020
8) Individuals administering care and support were kept 'blind' to intervention allocation.	NA	NA	NA	NA	NA
9) Based on above answers, was performance bias present?	Unclear	Unclear	Unclear	Unclear	Unclear
10) If so, what is the likely direction of its effect?	Unclear	Unclear	Unclear	Unclear	Unclear
11) All groups followed up for an equal length of time?	Low	Low	Low	Low	Low
12) Number of participants did not complete the intervention in each group?	NA	NA	NA	NA	NA
13) The groups were comparable for intervention completion.	NA	NA	NA	NA	NA
14) For how many participants were no outcome data available?‡	PROM 45/233 (19.3%); PTB 15/233 (6.4%)	61/456 (13.4%)	31/426 (7.3%)	15/206 (7.3%)	6.4% (64/1000)
15) Were groups comparable for outcome data?	Unclear	Yes	Yes	Yes	Unclear
16) Based on above answers, was attrition bias present?	Unclear	Low	Unclear	Unclear	Low
17) If so, what is the likely direction of its effect?	Unclear	Unclear	Unclear	Unclear	Unclear

Supporting information

Questions	Minkoff, 1984	Nguyen, 2004	Odendaal, 2002	Payne, 2016	Payne, 2020
18) The study had an appropriate length of follow-up.	Yes	Yes	Yes	Yes	Yes
19) The study used a precise definition of outcome.	Yes	Yes	Yes	Yes	Yes
20) A valid, reliable method used to determine the outcome?	Yes	Unclear	Yes	Unclear	Unclear
21) Investigators were kept 'blind' to participants' exposure to the intervention.	NA	NA	NA	NA	NA
22) Investigators were kept 'blind' to other important confounding factors.	Unclear	Unclear	Unclear	Unclear	Unclear
23) Based on above answers, was detection bias present?	No	Unclear	Unclear	Unclear	No
24) If so, what is the likely direction of its effect?	NA	Unclear	Unclear	Unclear	NA
**25) Overall assessment of internal validity[a]**	-	+	+	+	+
**26) Overall assessment of external validity[a]**	-	+	-	+	+

Abbreviations: AO, adverse outcomes; High, high risk of bias; Low, low risk of bias; NA, not applicable; NK, not known; STI, sexually transmitted infections; Unclear, unclear of risk of bias;

‡, both groups combined unless stated; [a]++, all or most of checklist criteria fulfilled; + some of checklist criteria fulfilled; - few or no checklist criteria fulfilled.

Supporting information

For peer review only - http://bmjopen.bmj.com/site/about/guidelines.xhtml

Table S6.1 Risk of bias assessment, cohort studies (n=26) (continued)

Questions	Peretz, 2020	Perni, 2004	Rittenschober, 2018	Schwab, 2015	Sperling, 1988	Usui, 2002
1) The method of allocation to exposure groups was unrelated to potential confounding factors	NA	NA	NA	NA	NA	NA
2) Attempts made within design or analysis to balance both groups for potential confounders.	Yes	Unclear	Yes	No	Unclear	Unclear
3) The groups were comparable at baseline, including all major confounding factors.	Unclear	Unclear	Unclear	Unclear	Unclear	Unclear
4) Based on above answers, was selection bias present?	Unclear	Unclear	Low	High	Unclear	Unclear
5) If so, what is the likely direction of its effect?	Unclear	Unclear	Unclear	Unclear	Unclear	Unclear
6) The comparison groups received the same care and support apart from the exposure(s) studied.	Yes	Unclear	Unclear	Unclear	Yes	Yes
7) Participants receiving care and support were kept 'blind' to intervention allocation.	NA	NA	NA	NA	NA	NA

Supporting information

Questions	Peretz, 2020	Perni, 2004	Rittenschober, 2018	Schwab, 2015	Sperling, 1988	Usui, 2002
8) Individuals administering care and support were kept 'blind' to intervention allocation.	NA	NA	NA	NA	NA	NA
9) Based on above answers, was performance bias present?	Unclear	Unclear	Unclear	Unclear	Unclear	Low
10) If so, what is the likely direction of its effect?	Unclear	Unclear	Unclear	Unclear	Unclear	NA
11) All groups followed up for an equal length of time?	Low	Low	Low	Low	Low	Low
12) Number of participants did not complete the intervention in each group?	NA	NA	NA	NA	NA	NA
13) The groups were comparable for intervention completion.	NA	NA	NA	NA	NA	NA
14) For how many participants were no outcome data available?‡	PTB 91% (195/214); LBW 90% (192/214)	14/193 (7.3%)	687/4330; (15.9%)	97/159 (61.0%)	1.2% (5/409)	0,0%
15) Were groups comparable for outcome data?	No	Unclear	Unclear	Unclear	Yes	Yes
16) Based on above answers, was attrition bias present?	High	Unclear	Unclear	High	Low	Low
17) If so, what is the likely direction of its effect?	Unclear	Unclear	Unclear	Unclear	NA	NA
18) The study had an appropriate length of follow-up.	Yes	Yes	Yes	Yes	Yes	Yes

Supporting information

Questions	Peretz, 2020	Perni, 2004	Rittenschober, 2018	Schwab, 2015	Sperling, 1988	Usui, 2002
19) The study used a precise definition of outcome.	Yes	Yes	Yes	Yes	Yes	Yes
20) A valid, reliable method used to determine the outcome?	Yes	Unclear	Yes	No	No	Yes
21) Investigators were kept 'blind' to participants' exposure to the intervention.	NA	NA	NA	NA	NA	NA
22) Investigators were kept 'blind' to other important confounding factors.	NA	Unclear	Unclear	Unclear	Unclear	Unclear
23) Based on above answers, was detection bias present?	No	Unclear	No	Yes	Unclear	Unclear
24) If so, what is the likely direction of its effect?	NA	Unclear	NA	Unclear	Unclear	Unclear
**25) Overall assessment of internal validity[a]**	-	+	+	-	-	+
**26) Overall assessment of external validity[a]**	-	+	+	-	-	-

Abbreviations: AO, adverse outcomes; High, high risk of bias; Low, low risk of bias; NA, not applicable; NK, not known; STI, sexually transmitted infections; Unclear, unclear of risk of bias.

‡, both groups combined unless stated; [a]++, all or most of checklist criteria fulfilled; + some of checklist criteria fulfilled; - few or no checklist criteria fulfilled.

Supporting information

Table S6.2 Risk of bias assessment, case-control studies (n=25)

Questions	Ahmadi, 2014	Cassell, 1993	Chua, 1999	Daskalakis, 2009	Embree, 1980	Farhadifar, 2016	Freitas, 2018
1) Appropriate and clearly focused question.	WC	WC	WC	WC	AA	WC	AA
2) The cases and controls are taken from comparable populations.	AA	WC	WC	AA	PA	AA	PA
3) The same exclusion criteria are used for both cases and controls.	WC	NA	PA	WC	NAd	AA	NAd
4) What was the participation rate (%) for each group (cases)?	Unclear	87.9 (29/33)	Unclear	Unclear	100% (n=446)	Unclear	100% (n=46)
5) What was the participation rate (%) for each group (controls)?	Unclear	100 (28/28)	Unclear	Unclear	100% (n=108)	Unclear	100% (n=170)
6) Both groups compared to establish their similarities or differences.	NAd	NAd	NAd	NAd	NAd	NAd	AA
7) Cases are clearly defined and differentiated from controls.	WC	AA	AA	WC	AA	WC	AA

For peer review only - http://bmjopen.bmj.com/site/about/guidelines.xhtml

Questions	Ahmadi, 2014	Cassell, 1993	Chua, 1999	Daskalakis, 2009	Embree, 1980	Farhadifar, 2016	Freitas, 2018
8) It is clearly established that controls are not cases.	WC	AA	AA	WC	AA	WC	AA
9) Measures taken to prevent knowledge of primary exposure from influencing case ascertainment.	NA	NA	NA	NA	NA	NA	NA
10) Exposure status is measured in a standard, valid and reliable way.	WC	AA	AA	WC	AA	AA	WC
11) Main potential confounders are accounted for in design/analysis	AA	PA	NR	AA	PA	AA	PA
12) Confidence intervals provided?	No	No	No	No	No	Yes	No
**13) Study results internally valid[a]**	+	+	+	+	-	+	+
**14) Study results externally valid[a]**	+	-	+	+	-	+	-

Abbreviations: AA, adequately addressed; NAd, not addressed; NA, not applicable; PA, poorly addressed; WC, well covered; Unclear, does not have data on how many were excluded or declined to participate but only present numbers

[a]++, all or most of checklist criteria fulfilled; + some of checklist criteria fulfilled; - few or no checklist criteria fulfilled.

Supporting information

For peer review only - http://bmjopen.bmj.com/site/about/guidelines.xhtml

Table S6.2 Risk of bias assessment, case-control studies (n=25) (continued)

Questions	Gonzàlez Bosquet, 2006	Harada, 2008	Hillier, 1988	Holst, 1994	Jalava, 2002	Jones, 2009
1) Appropriate and clearly focused question.	WC	WC	WC	WC	NR	WC
2) The cases and controls are taken from comparable populations.	AA	AA	AA	PA	AA	PA
3) The same exclusion criteria are used for both cases and controls.	AA	AA	PA	AA	PA	NAd
4) What was the participation rate for each group (cases)? %	Unclear	Unclear	99/107 (92.5%)	40.8 (49/120)	100 (n=50)	Unclear
5) What was the participation rate for each group (controls)? %	Unclear	Unclear	68/140 (48.6%)	100 (38/38)	72 (72/100)	Unclear
6) Both groups compared to establish their similarities or differences.	NAd	NAd	NAd	NAd	NAd	NAd
7) Cases are clearly defined and differentiated from controls.	WC	AA	WC	WC	WC	AA
8) It is clearly established that controls are not cases.	WC	AA	WC	WC	AA	AA
9) Measures taken to prevent knowledge of primary exposure from influencing case ascertainment.	NA	NA	NA	NA	NA	NA

For peer review only - http://bmjopen.bmj.com/site/about/guidelines.xhtml

**Questions**	**Gonzàlez Bosquet, 2006**	**Harada, 2008**	**Hillier, 1988**	**Holst, 1994**	**Jalava, 2002**	**Jones, 2009**
10) Exposure status is measured in a standard, valid, and reliable way.	AA	WC	AA	AA	WC	WC
11) Main potential confounders are accounted for in design/analysis	PA	PA	WC	AA	NAd	NAd
12) Confidence intervals provided?	No	Yes	Yes	No	No	No
**13) Study results internally valid**[a]	+	+	+	++	+	-
**14) Study results externally valid**[a]	+	+	+	+	+	-

Abbreviations: AA, adequately addressed; NAd, not addressed; NA, not applicable; PA, poorly addressed; WC, well covered; Unclear, does not have data on how many were excluded or declined to participate but only present numbers.

[a] ++, all or most of checklist criteria fulfilled; + some of checklist criteria fulfilled; - few or no checklist criteria fulfilled.

Supporting information

Table S6.2 Risk of bias assessment, case-control studies (n=25) (continued)

Questions	Kacerovsky, 2009	Kafetzis, 2004	Kumar, 2006	McDonald, 1992	Matsunari, 2005	Montenegro, 2019
1) Appropriate and clearly focused question.	WC	WC	AA	AA	WC	WC
2) The cases and controls are taken from comparable populations.	PA	WC	PA	AA	WC	AA
3) The same exclusion criteria are used for both cases and controls.	PA	NR	NAd	PA	WC	AA
4) What was the participation rate for each group (cases)?	Unclear	Unclear	100% (n=60)	Unclear	57.5 (23/40)	84 (100%)
5) What was the participation rate for each group (controls)?	Unclear	Unclear	100% (n=60)	Unclear	60.8 (59/97)	127 (1000%)
6) Both groups compared to establish their similarities or differences.	NAd	NAd	NAd	AA	NA	NA
7) Cases are clearly defined and differentiated from controls.	WC	WC	NAd	AA	AA	AA
8) It is clearly established that controls are not cases.	PA	WC	AA	AA	AA	AA
9) Measures taken to prevent knowledge of primary exposure from influencing case ascertainment.	NA	NA	NA	NA	NA	NA

For peer review only - http://bmjopen.bmj.com/site/about/guidelines.xhtml

Questions	Kacerovsky, 2009	Kafetzis, 2004	Kumar, 2006	McDonald, 1992	Matsunari, 2005	Montenegro, 2019
10) Exposure status is measured in a standard, valid, and reliable way.	AA	WC	PA	PA	WC	WC
11) Main potential confounders are accounted for in design/analysis	PA	NAd	NAd	AA	PA	PA
12) Confidence intervals provided?	No	No	No	Yes	No	No
**13) Study results internally valid[a]**	-	+	-	+	+	+
**14) Study results externally valid[a]**	-	+	-	+	+	+

Abbreviations: AA, adequately addressed; NAd, not addressed; NA, not applicable; PA, poorly addressed; WC, well covered; Unclear, does not have data on how many were excluded or declined to participate but only present numbers

[a]++, all or most of checklist criteria fulfilled; + some of checklist criteria fulfilled; - few or no checklist criteria fulfilled.

Supporting information

Table S6.2 Risk of bias assessment, case-control studies (n=25) (continued)

Questions	Munday, 1984	Oliveira, 2020	Payne, 2014	Povlsen, 2001	Toth, 1992	Yoon, 2001
1) Appropriate and clearly focused question.	WC	AA	WC	WC	WC	WC
2) The cases and controls are taken from comparable populations.	AA	PA	WC	WC	AA	AA
3) The same exclusion criteria are used for both cases and controls.	NAd	PA	AA	NAd	PA	PA
4) What was the participation rate for each group (cases)?	Unclear	100%	100%	Unclear	Unclear	Unclear
5) What was the participation rate for each group (controls)?	Unclear	100%	100%	Unclear	Unclear	Unclear
6) Both groups compared to establish their similarities or differences.	NA	AA	NAd	NA	NA	NA
7) Cases are clearly defined and differentiated from controls.	PA	WC	AA	AA	PA	WC
8) It is clearly established that controls are not cases.	PA	WC	PA	PA	PA	WC
9) Measures taken to prevent knowledge of primary exposure from influencing case ascertainment.	NA	NA	NAd	NA	NA	NA
10) Exposure status is measured in a standard, valid, and reliable way.	AA	AA	AA	AA	PA	WC
11) Main potential confounders are accounted for in design/analysis	PA	AA	NAd	NAd	PA	AA
12) Confidence intervals provided?	No	Yes	Yes	Yes	No	No

For peer review only - http://bmjopen.bmj.com/site/about/guidelines.xhtml

Questions	Munday, 1984	Oliveira, 2020	Payne, 2014	Povlsen, 2001	Toth, 1992	Yoon, 2001
**13) Study results internally valid**[a]	+	+	+	+	-	+
**14) Study results externally valid**[a]	-	+	+	+	-	+

Abbreviations: AA, adequately addressed; NAd, not addressed; NA, not applicable; PA, poorly addressed; Unclear, does not have data on how many were excluded or declined to participate but only present numbers; WC, well covered.

[a]++, all or most of checklist criteria fulfilled; + some of checklist criteria fulfilled; - few or no checklist criteria fulfilled.

Supporting information

For peer review only - http://bmjopen.bmj.com/site/about/guidelines.xhtml

Table S6.3 Risk of bias assessment, cross-sectional studies (n=6)

Questions	Abele-Horn, 2000	Grattard, 1995	Kundsin, 1984	McCormack, 1975	Nasution, 2007	Sweeney, 2016
1) Is the source population, source area well described?	+	+	NR	-	NR	+
2) Is the eligible population or area representative of the source population?	-	+	NR	-	NR	-
3) Do the selected participants or areas represent the eligible population or area?	-	NR	-	-	NR	-
4) Selection of exposure (and comparison) group. How was selection bias minimised?	NR	NR	NR	NR	NR	NR
5) Was the selection of explanatory variables based on a sound theoretical basis?	+	-	+	-	+	+
6) Was the contamination acceptably low?	NA	NA	NA	NA	NR	NA
7) How well were likely confounding factors identified and controlled?	+	NR	NR	NR	NR	-
8) Outcome measures and procedures reliable?	+	-	+	-	+	-
9) Were the outcome measurements complete?	+	+	+	+	++	+

For peer review only - http://bmjopen.bmj.com/site/about/guidelines.xhtml

10) Were all the important outcomes assessed?	-	+	+	+	+	++
11) Was there a similar follow-up time in exposure and comparison groups?	+	-	++	+	++	+
12) Was follow-up time meaningful?	+	+	++	+	++	+
13) Was the study sufficiently powered to detect an exposure effect (if one exists)	NA	NA	NA	NA	NR	NA
14) Were multiple explanatory variables considered in analyses?	NR	NR	NR	NR	NR	NR
15) Were the analytical methods appropriate?	+	-	-	+	-	+
16) Was the precision of association given or calculable?	+	+	+	+	+	+
**17) Overall assessment of internal validity[a]**	**-**	**-**	**-**	**+**	**-**	**+**
**18) Overall assessment of external validity[a]**	**-**	**+**	**+**	**-**	**-**	**-**

Abbreviations: ++, yes; +, mostly; -, no; NR, not reported; NA, not applicable.

[a] ++, all or most of checklist criteria fulfilled; + some of checklist criteria fulfilled; - few or no checklist criteria fulfilled.

Supporting information

For peer review only - http://bmjopen.bmj.com/site/about/guidelines.xhtml

Table S7 Summary of assessment of funnel plot asymmetry, for outcomes reported in 10 or more studies

Organism	Outcome	Egger test (95% CI)*	P value
M. hominis	PTB	0.56 (-0.08, 1.2)	0.09
	PROM	0.05 (-1.07, 1.17)	0.92
	SA	-0.28 (-3.20, 2.64)	0.83
U. urealyticum	PTB	0.89 (-0.15, 1.93)	0.09
	PROM	1.2 (-1.7, 4.09)	0.37
U. parvum	PTB	0.53 (-1.27, 2.34)	0.52

Abbreviations: CI, confidence interval; PND: perinatal death; PTB: preterm birth; PROM: premature rupture of membrane; S: spontaneous abortion

* Egger test for small-study effects

For peer review only - http://bmjopen.bmj.com/site/about/guidelines.xhtml

Research checklists

A. 1 Preferred reporting items for systematic review and meta-analysis (PRISMA)

Section and Topic	Item #	Checklist item	Location where item is reported
TITLE			
Title	1	Identify the report as a systematic review.	1
ABSTRACT			
Abstract	2	See the PRISMA 2020 for Abstracts checklist.	4-5
INTRODUCTION			
Rationale	3	Describe the rationale for the review in the context of existing knowledge.	6
Objectives	4	Provide an explicit statement of the objective(s) or question(s) the review addresses.	7
METHODS			
Eligibility criteria	5	Specify the inclusion and exclusion criteria for the review and how studies were grouped for the syntheses.	7
Information sources	6	Specify all databases, registers, websites, organisations, reference lists and other sources searched or consulted to identify studies. Specify the date when each source was last searched or consulted.	8
Search strategy	7	Present the full search strategies for all databases, registers and websites, including any filters and limits used.	Appendix S1
Selection process	8	Specify the methods used to decide whether a study met the inclusion criteria of the review, including how many reviewers screened each record and each report	8

For peer review only - http://bmjopen.bmj.com/site/about/guidelines.xhtml

Research checklists

Section and Topic	Item #	Checklist item	Location where item is reported
		retrieved, whether they worked independently, and if applicable, details of automation tools used in the process.	
Data collection process	9	Specify the methods used to collect data from reports, including how many reviewers collected data from each report, whether they worked independently, any processes for obtaining or confirming data from study investigators, and if applicable, details of automation tools used in the process.	8-9
Data items	10a	List and define all outcomes for which data were sought. Specify whether all results that were compatible with each outcome domain in each study were sought (e.g. for all measures, time points, analyses), and if not, the methods used to decide which results to collect.	6
	10b	List and define all other variables for which data were sought (e.g. participant and intervention characteristics, funding sources). Describe any assumptions made about any missing or unclear information.	8-9
Study risk of bias assessment	11	Specify the methods used to assess risk of bias in the included studies, including details of the tool(s) used, how many reviewers assessed each study and whether they worked independently, and if applicable, details of automation tools used in the process.	9
Effect measures	12	Specify for each outcome the effect measure(s) (e.g. risk ratio, mean difference) used in the synthesis or presentation of results.	9
Synthesis methods	13a	Describe the processes used to decide which studies were eligible for each synthesis (e.g. tabulating the study intervention characteristics and comparing against the planned groups for each synthesis (item #5)).	9

For peer review only

Research checklists

Section and Topic	Item #	Checklist item	Location where item is reported
	13b	Describe any methods required to prepare the data for presentation or synthesis, such as handling of missing summary statistics, or data conversions.	9
	13c	Describe any methods used to tabulate or visually display results of individual studies and syntheses.	9
	13d	Describe any methods used to synthesize results and provide a rationale for the choice(s). If meta-analysis was performed, describe the model(s), method(s) to identify the presence and extent of statistical heterogeneity, and software package(s) used.	9
	13e	Describe any methods used to explore possible causes of heterogeneity among study results (e.g. subgroup analysis, meta-regression).	9
	13f	Describe any sensitivity analyses conducted to assess robustness of the synthesized results.	None
Reporting bias assessment	14	Describe any methods used to assess risk of bias due to missing results in a synthesis (arising from reporting biases).	9
Certainty assessment	15	Describe any methods used to assess certainty (or confidence) in the body of evidence for an outcome.	9
RESULTS			
Study selection	16a	Describe the results of the search and selection process, from the number of records identified in the search to the number of studies included in the review, ideally using a flow diagram.	Figure S1

For peer review only

Research checklists

Section and Topic	Item #	Checklist item	Location where item is reported
	16b	Cite studies that might appear to meet the inclusion criteria, but which were excluded, and explain why they were excluded.	Figure S1
Study characteristics	17	Cite each included study and present its characteristics.	Table S1, reference list
Risk of bias in studies	18	Present assessments of risk of bias for each included study.	11, supporting information
Results of individual studies	19	For all outcomes, present, for each study: (a) summary statistics for each group (where appropriate) and (b) an effect estimate and its precision (e.g. confidence/credible interval), ideally using structured tables or plots.	Table 1, Fig 1, Fig 2, Fig 3, supporting information
Results of syntheses	20a	For each synthesis, briefly summarise the characteristics and risk of bias among contributing studies.	11, supporting information
	20b	Present results of all statistical syntheses conducted. If meta-analysis was done, present for each the summary estimate and its precision (e.g. confidence/credible interval) and measures of statistical heterogeneity. If comparing groups, describe the direction of the effect.	11-14
	20c	Present results of all investigations of possible causes of heterogeneity among study results.	Supporting information
	20d	Present results of all sensitivity analyses conducted to assess the robustness of the synthesized results.	None
Reporting biases	21	Present assessments of risk of bias due to missing results (arising from reporting biases) for each synthesis assessed.	None

For peer review only - http://bmjopen.bmj.com/site/about/guidelines.xhtml

Research checklists

Section and Topic	Item #	Checklist item	Location where item is reported
Certainty of evidence	22	Present assessments of certainty (or confidence) in the body of evidence for each outcome assessed.	Fig 1, Fig 2, Fig 3, supporting information
DISCUSSION			
Discussion	23a	Provide a general interpretation of the results in the context of other evidence.	15
	23b	Discuss any limitations of the evidence included in the review.	16
	23c	Discuss any limitations of the review processes used.	15
	23d	Discuss implications of the results for practice, policy, and future research.	17-18
OTHER INFORMATION			
Registration and protocol	24a	Provide registration information for the review, including register name and registration number, or state that the review was not registered.	1
	24b	Indicate where the review protocol can be accessed, or state that a protocol was not prepared.	1
	24c	Describe and explain any amendments to information provided at registration or in the protocol.	None
Support	25	Describe sources of financial or non-financial support for the review, and the role of the funders or sponsors in the review.	18
Competing interests	26	Declare any competing interests of review authors.	19

For peer review only

Research checklists

Section and Topic	Item #	Checklist item	Location where item is reported
Availability of data, code and other materials	27	Report which of the following are publicly available and where they can be found: template data collection forms; data extracted from included studies; data used for all analyses; analytic code; any other materials used in the review.	Supporting information

For peer review only

Research checklists

A.2 Preferred reporting checklist of Meta-analysis (MOOSE)[1]

**Checklist item**	**Page #**
**Reporting of background should include**	
Problem definition	6
Hypothesis statement	No
Description of study outcome(s)	6
Type of exposure or intervention used	6-7
Type of study designs used	7
Study population	7
**Reporting of search strategy should include**	
Qualifications of searchers (eg. Librarians and investigators)	1
Search strategy, including time period included in the synthesis and keywords	7-8, Appendix S1
Effort to include all available studies, including contact with authors	No
Databases and registries searched	8
Search software used, name and version, including special features used (eg, explosion)	No
Use of hand searching (eg, reference lists of obtained articles	8
List of citations located and those excluded, including justification	Fig 1,<br>Table S1, excluded studies not listed

For peer review only

Research checklists

Method of addressing articles published in languages other than English	8
Method of handling abstracts and unpublished studies	Excluded
Description of any contact with authors	We did not contact authors
**Reporting of methods should include**	
Description of relevance or appropriateness of studies assembled for assessing the hypothesis to be tested	7-8
Rationale for the selection and coding of data (eg, multiple raters, blinding and interrater reliability)	7-8
Assessment of confounding (eg, comparability of cases and controls in studies where appropriate)	7
Assessment of study quality, including blinding of quality assessors; stratification or regression on possible predictors of study results	9
Assessment of heterogeneity	9
Description of statistical methods (eg, complete description of fixed or random effects models, justification of whether the chosen models account for predictors of study results, dose-response models, cumulative meta-analysis) in sufficient detail to be replicated	9
Provision of appropriate tables and graphs	Main text and supporting information
**Reporting of results should include**	
Graphic summarizing individual study estimates and overall estimate	Main text and forest plots in supporting information

For peer review only

Research checklists

Table giving descriptive information for each study included	Table S1
Results of sensitivity testing (eg, subgroup analysis)	None
Indication of statistical uncertainty of findings	Main text and forest plots in supporting information
**Reporting of discussion should include**	
Quantitative assessment of bias (eg, publication bias)	15-16
Justification of exclusion (eg. Exclusion of non-English-language citations)	No (provided pg 8 on methods)
Assessment of Quality of included studies	15-17
**Reporting of conclusions should include**	
Consideration of alternative explanations of observations	17-18
Generalization of conclusions (ie, appropriate for the data presented and within the domain of the literature review)	17-18
Guidelines for future research	17-18
Disclosure of funding source	18

[1] Stroup DF, Berlin JA, Morton SC, Olkin I, Williamson GD, Rennie D, et al. Meta-analysis of observational studies in epidemiology: a proposal for reporting. **Jama**. 2000 Apr 19;283(15):2008-12.

For peer review only
